# Teclistamab-based induction treatment in transplant-eligible, newly diagnosed multiple myeloma: a phase 2 trial

**DOI:** 10.1038/s41591-026-04471-x

**Published:** 2026-06-25

**Authors:** Marc S. Raab, Niels Weinhold, K. Martin Kortüm, Jan Krönke, Roland Fenk, Katja Weisel, Lilli Podola, Uta Bertsch, Alexander Brobeil, Julia Mersi, Stefanie Huhn, Ryan Arlinghaus, Michael Hundemer, Stephan R. Bohl, Elias K. Mai, Natalie Schub, Johannes Waldschmidt, Florian Bassermann, Carsten Müller-Tidow, Christoph Heuck, Caline Sakabedoyan, Josephine Khan, Elena Ershova, Bas D. Koster, Monika Engelhardt, Mathias Hänel, Hans Salwender, Raphael Teipel, Hartmut Goldschmidt, Hermann Einsele, Leo Rasche

**Affiliations:** 1 Heidelberg Myeloma Center and GMMG Study Group, Department of Medicine V, Heidelberg University Hospital and Medical Faculty Heidelberg, Heidelberg, Germany; 2 Department of Internal Medicine II, University Hospital of Würzburg, Würzburg, Germany; 3 Department of Hematology, Oncology, and Tumor Immunology, Charité – Universitätsmedizin Berlin, corporate member of Freie Universität Berlin and Humboldt-Universität zu Berlin, Berlin, Germany; 4 Deutsches Konsortium für Translationale Krebsforschung (DKTK), Partner Site Berlin, Berlin, Germany; 5 Department of Hematology, Oncology and Clinical Immunology, University Hospital Düsseldorf, Medical Faculty, Heinrich Heine University Düsseldorf, Düsseldorf, Germany; 6 University Medical Center Hamburg-Eppendorf, Hamburg, Germany; 7 Institute of Pathology, University Hospital Heidelberg, Heidelberg, Germany; 8 Coordination Centre for Clinical Trials (KKS), University Hospital Heidelberg, Heidelberg, Germany; 9 Department of Internal Medicine II, Division of Stem Cell Transplantation and Immunotherapy, Universitätsklinikum Schleswig-Holstein (UKSH) Campus Kiel, Kiel, Germany; 10 Department of Medicine III, TUM Klinikum, Technische Universität München, München, Germany; 11 Deutsches Konsortium für Translationale Krebsforschung (DKTK), Deutsches Krebsforschungszentrum (DKFZ), Heidelberg, Germany; 12 Bayerisches Zentrum für Krebsforschung (BZKF), Munich, Germany; 13 Johnson & Johnson, Spring House, PA USA; 14 Johnson & Johnson, Paris, France; 15 Johnson & Johnson, High Wycombe, UK; 16 Johnson & Johnson, Beerse, Belgium; 17 Johnson & Johnson, Leiden, The Netherlands; 18 Department of Hematology, Oncology and Stem Cell Transplantation, Medical Centre - University of Freiburg, Faculty of Medicine, University of Freiburg, Freiburg, Germany; 19 Department of Internal Medicine III, Klinikum Chemnitz, Chemnitz, Germany; 20 Asklepios Tumorzentrum Hamburg, Asklepios Klinik Altona and Asklepios Klinik St. Georg, Hamburg, Germany; 21 Medizinische Klinik und Poliklinik I Universitätsklinikum Carl Gustav Carus an der Technische Universität Dresden, Dresden, Germany

**Keywords:** Myeloma, Cancer immunotherapy

## Abstract

Advancements in frontline therapies have substantially improved outcomes in newly diagnosed multiple myeloma (NDMM); however, many patients will not achieve deep responses and will relapse. Teclistamab, a BCMA×CD3 bispecific antibody, in combination with daratumumab, has demonstrated strong efficacy in relapsed/refractory multiple myeloma versus standard of care as early as first relapse. This ongoing phase 2 GMMG-HD10/DSMM-XX (MajesTEC-5) study evaluates teclistamab-based regimens in transplant-eligible NDMM. In this prespecified pooled analysis of three cohorts, 49 patients received teclistamab/daratumumab/lenalidomide (Tec-DR; arms A and A1) or Tec-DR with bortezomib (Tec-DVR; arm B). Primary endpoints were incidence and severity of adverse events (AEs) and serious AEs; secondary endpoints included overall response rate (ORR), minimal residual disease (MRD) negativity and MRD-negative complete response (CR). The current analysis spans the induction and autologous stem cell transplantation phases until the premaintenance timepoint. Grade 3 or 4 treatment-emergent AEs (TEAEs) occurred in 91.8% (45/49); most were hematologic (lymphopenia (59.2%; 29/49), neutropenia (59.2%; 29/49) and leukopenia (18.4%; 9/49)). No grade 5 TEAEs were reported. Serious AEs occurred in 55.1% (27/49); pyrexia (12.2% (6/49)) was most common. Any-grade and grade 3 or 4 infections occurred in 81.6% (40/49) and 36.7% (18/49), respectively, the most common grade 3 or 4 infections being COVID-19 and pneumonia (6.1% (3/49) each). Cytokine release syndrome occurred in 67.3% (33/49); all were grade 1 or 2, all resolved and none led to discontinuation of any study treatment. No treatment-related immune effector cell-associated neurotoxicity syndrome (ICANS) events occurred. Across arms, the MRD-negative CR rate was 91.8% (45/49) by the premaintenance timepoint; the MRD negativity rate was 100% in evaluable samples at postinduction cycle 3 (1 × 10^−5^ (46/46)), cycle 6 (1 × 10^−5^ (46/46) and 1 × 10^−6^ (46/46)) and premaintenance (1 × 10^−5^ (40/40)); the ORR was 100% (49/49). Total median stem cell yield was 8.1 × 10^6^ per kg. Data support the feasibility of Tec-D(V)R induction in transplant-eligible NDMM, with a consistent safety profile compared with individual regimen components and notable early MRD negativity rates. ClinicalTrials.gov identifier: NCT05695508.

## Abstract (web summary)

In the ongoing phase 2 GMMG-HD10/DSMM-XX (MajesTEC-5) trial in patients with transplant-eligible, newly diagnosed multiple myeloma, induction with the BCMA×CD3 bispecific engager teclistamab in combination with daratumumab plus lenalidomide, with or without bortezomib, had a similar toxicity profile to other bispecific regimens with an encouraging and deep response rate.

## Main

Treatment advancements in newly diagnosed multiple myeloma (NDMM) have led to notable efficacy and extended survival. For instance, daratumumab-based frontline regimens have been shown to elicit deep responses, as demonstrated in PERSEUS, with a cumulative postconsolidation minimal residual disease (MRD)-negative (1 × 10^−5^) complete response or better (≥CR) rate of 57.5% (ref. ^1^), continuing to increase over time and leading to a sustained (≥12-month) deep response in 64.8% of patients^2^. However, because MRD negativity strongly predicts long-term outcomes^3^, yet is still not achieved in all patients, novel therapies should be explored to deepen and prolong responses, in addition to improving progression-free and overall survival.

Teclistamab is a first-in-class bispecific antibody targeting CD3 on the surface of T cells and B cell maturation antigen (BCMA) on the surface of myeloma cells^4,5^. In the phase 1/2 MajesTEC-1 study, through 30.4 months of follow-up, teclistamab monotherapy induced a ≥CR in 46.1% of patients with triple-class-exposed relapsed refractory disease^6^. These promising response rates in these late-line patients, along with the known advantage of immune fitness for long-term clinical benefit^7^, make teclistamab a promising candidate for use in the frontline setting. As effective eradication of all subclones has been shown to require a multifaceted therapeutic approach^8^, we aimed to evaluate teclistamab in combination with other standard-of-care regimens.

The combination of teclistamab with daratumumab has been shown preclinically to enhance antimyeloma effects, with the immunomodulatory action of daratumumab creating a more sensitive immune microenvironment to augment the cytotoxic action of teclistamab^9^, thus resulting in durable and sustained antimyeloma activity. Likewise, pairing a bispecific antibody with an immunomodulatory drug (IMiD) such as lenalidomide offers a compelling approach for increasing antimyeloma activity, given that lenalidomide has been shown to enhance tumor cell killing induced by CD3 bispecifics and increase beneficial T cell function and expansion^10^. As such, efficacy of a teclistamab–daratumumab combination may be further potentiated by the addition of lenalidomide, which, besides its direct effect on multiple myeloma cells, may boost cytotoxic T cell activity. The addition of a proteasome inhibitor may also augment efficacy, given the critical role of the proteasome in myeloma pathogenesis and its potential to improve outcomes in patients with certain high-risk aberrations, as demonstrated by bortezomib^11^. Teclistamab in combination with daratumumab was recently evaluated in the global phase 3 MajesTEC-3 study and demonstrated significant improvements in progression-free and overall survival versus standard of care as early as second-line relapsed/refractory multiple myeloma, with a safety profile that was consistent with that known for each individual component^12^. In light of these results, this fully immunotherapy-based combination was recently approved by the US Food and Drug Administration for the treatment of relapsed/refractory multiple myeloma in patients with at least one prior line of therapy, including a proteasome inhibitor and an IMiD^4^.

GMMG-HD10/DSMM-XX (MajesTEC-5; NCT05695508) is, to our knowledge, the first multicohort study to evaluate the feasibility of teclistamab in combination with daratumumab-based standard-of-care regimens for transplant-eligible patients with NDMM. Here we report outcomes of a pooled analysis (permitted, including modifications, per the statistical analysis plan) of three specific induction cohorts in MajesTEC-5 after the completion of induction therapy with teclistamab in combination with daratumumab and lenalidomide (Tec-DR) alone (arms A and A1) or in combination with bortezomib (Tec-DVR; arm B) and high-dosage melphalan with autologous stem cell transplantation (ASCT), up to the last visit prior to initiation of maintenance therapy (hereby referred to as ‘premaintenance treatment timepoint’).

## Results

This is an ongoing, multicenter, open-label, nonrandomized, multicohort phase 2 study. Eligible patients were 18–70 years of age, had NDMM per International Myeloma Working Group (IMWG) diagnostic criteria^13^ and were eligible for high-dose therapy and ASCT. This pooled data analysis of arms A, A1 and B was permitted (inclusive of modifications) per the statistical analysis plan. The primary endpoint was the incidence and severity of adverse events and serious adverse events. Prespecified secondary efficacy endpoints reported here include MRD-negative CR rate, MRD negativity rates, ORR (partial response or better), very good partial response or better (≥VGPR), ≥CR (response assessed by investigators per IMWG consensus criteria)^13–15^ and stem cell yield and days to engraftment. Additional secondary endpoints not reported include duration of response, sustained (≥12-month) MRD-negative CR rate, progression-free survival, pharmacokinetics and immunogenicity.

### Patients

**Fig. 1:**
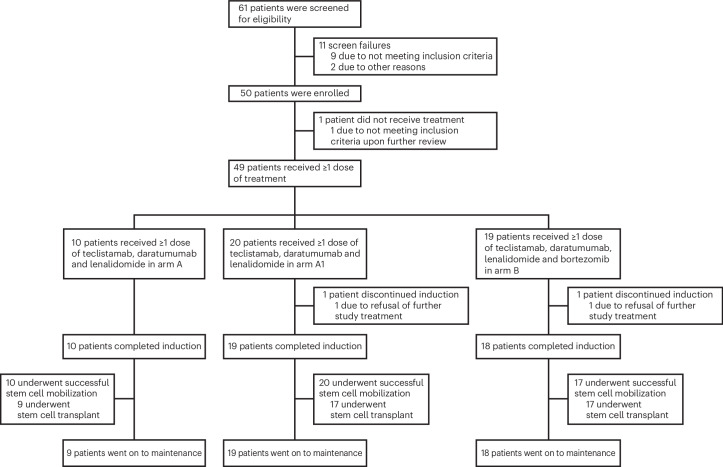
CONSORT diagram for arms A, A1 and B. Sixty-one patients were screened; 11 failed screening due to not meeting inclusion criteria (*n* = 9) and ‘other’ reasons (no request made, *n* = 2). Fifty patients were enrolled; however, upon further review, it was determined that one patient in arm B did not meet inclusion criteria, and, thus, the patient did not receive study treatment. A total of 49 patients received at least 1 dose of study treatment: arm A (teclistamab (1.5 mg kg^−1^ weekly) with daratumumab and lenalidomide), *n* = 10; arm A1 (teclistamab (3.0 mg kg^−1^ monthly) with daratumumab and lenalidomide), *n* = 20; and arm B (teclistamab (3.0 mg kg^−1^ monthly) with daratumumab, lenalidomide and bortezomib), *n* = 19.

A total of 50 patients were enrolled, of whom 49 received at least one dose of study treatment (arm A, *n* = 10; arm A1, *n* = 20; arm B, *n* = 19; Fig. 1). Demographic and clinical characteristics of patients are summarized in Table 1. Among all patients, the median age was 58.0 years (range, 30.0–68.0), and six patients (12.2%) had International Staging System stage III disease. A total of 10 patients (20.4%) had high cytogenetic risk, defined as the presence of at least one of del(17p), t(4;14) or t(14;16), the most common abnormalities being t(4;14) (*n* = 5 (10.2%)) and del(17p) (*n* = 4 (8.2%)). No patient had extramedullary soft tissue plasmacytomas at baseline.

**Table 1:** Summary of baseline demographic and patient characteristics

Characteristic	Arm A^a^ (*n* = 10)	Arm A1^b^ (*n* = 20)	Arm B^c^ (*n* = 19)	Total (*n* = 49)
Age				
Median (range), years	63.0 (54.0–66.0)	57.5 (36.0–65.0)	56.0 (30.0–68.0)	58.0 (30.0–68.0)
Sex, *n* (%)				
Male	6 (60.0)	13 (65.0)	12 (63.2)	31 (63.3)
Female	4 (40.0)	7 (35.0)	7 (36.8)	18 (36.7)
Ethnicity, *n* (%)				
Caucasian	10 (100)	20 (100)	19 (100)	49 (100)
ECOG performance status score^d^, *n* (%)				
0	7 (70.0)	13 (65.0)	6 (31.6)	26 (53.1)
1	2 (20.0)	7 (35.0)	12 (63.2)	21 (42.9)
2	1 (10.0)	0	1 (5.3)	2 (4.1)
International Staging System stage^e^, *n* (%)				
I	8 (80.0)	10 (50.0)	10 (52.6)	28 (57.1)
II	1 (10.0)	7 (35.0)	7 (36.8)	15 (30.6)
III	1 (10.0)	3 (15.0)	2 (10.5)	6 (12.2)
≥60% plasma cells in bone marrow^f^, *n* (%)	4 (40.0)	10 (50.0)	8 (42.1)	22 (44.9)
≥1 paraskeletal soft tissue plasmacytoma^g^, *n* (%)	0	5 (25.0)	4 (21.1)	9 (18.4)
Cytogenetic risk^h^, *n* (%)				
Standard risk	9 (90.0)	13 (65.0)	12 (63.2)	34 (69.4)
High risk	1 (10.0)	5 (25.0)	4 (21.1)	10 (20.4)
Indeterminate risk	0	2 (10.0)	3 (15.8)	5 (10.2)

^a^Teclistamab (1.5 mg kg^−1^ weekly), daratumumab and lenalidomide treatment group.

^b^Teclistamab (3.0 mg kg^−1^ monthly), daratumumab and lenalidomide treatment group.

^c^Teclistamab (3.0 mg kg^−1^ monthly), daratumumab, lenalidomide and bortezomib treatment group.

^d^ECOG performance status scores range from 0 to 5, with a higher score indicating greater disability.

^e^International Staging System stage is derived based on serum β-2-microglobulin and albumin.

^f^Maximum value from bone marrow biopsy or bone marrow aspirate was selected if both of the results were available.

^g^All soft tissue plasmacytomas reported were paraskeletal in nature, and no extramedullary soft tissue plasmacytomas were reported.

^h^Cytogenetic risk was based on central fluorescence in situ hybridization or local fluorescence in situ hybridization if central analysis was unavailable. High cytogenetic risk was defined as the presence of at least one of the following abnormalities: del(17p), t(4;14) or t(14;16).

Forty-six patients (93.9%) completed all six cycles of induction treatment, with a median induction treatment duration of 7.0 months (range, 2.5–13.2). Dose changes and discontinuations are shown in Extended Data Table 1. Dose reductions of lenalidomide were most commonly due to onset of neutropenia; permanent discontinuations of lenalidomide were primarily due to adverse events (Extended Data Table 1). Median overall relative dose intensity, relative to per-protocol defined doses, by the premaintenance treatment timepoint was 92.6% for teclistamab (including teclistamab step-up and repeat step-up doses), 90.0% for daratumumab, 72.4% for lenalidomide, 93.3% for dexamethasone and 90.2% for bortezomib. The median follow-up by the premaintenance treatment timepoint (time from the first dose of study treatment to the last visit prior to the start of maintenance) was 12.3 months (arm A, 11.9 months; arm A1, 12.5 months; arm B, 12.2 months).

### Safety

All patients reported a treatment-emergent adverse event (TEAE), which was grade 3 or 4 in 45 patients (91.8%). No grade 5 events were reported. Table 2 shows the most common TEAEs of any grade (occurring in ≥25% of patients in either arm) and respective grade 3 or 4 events. Most grade 3 or 4 TEAEs were hematologic, including lymphopenia (29 patients (59.2%)), neutropenia (29 (59.2%)) and leukopenia (9 (18.4%)). Any-grade infections occurred in 40 patients (81.6%); 18 patients (36.7%) had grade 3 or 4 infections (Table 3). No grade 5 infections were reported in any cohort. Cytomegalovirus reactivation was the only opportunistic infection reported, occurring in three patients (6.1%) and resolving in all instances. For all three patients, only viremia without organ affection was present. By the premaintenance treatment timepoint, 45 patients (91.8%) reported at least one hypogammaglobulinemia TEAE or postbaseline immunoglobulin G value of <400 mg dl^−1^; 44 patients (89.8%) received at least one dose of intravenous immunoglobulin. Of note, prior to treatment administration, 49 patients (100%) received acyclovir for herpes zoster prevention, and 46 patients (93.9%) received a combination of sulfamethoxazole and trimethoprim for *Pneumocystis jirovecii* pneumonia prophylaxis.

**Table 2:** Summary of the most common^a^ TEAEs

Event, *n* (%)	Arm A^b^ (*n* = 10)		Arm A1^c^ (*n* = 20)		Arm B^d^ (*n* = 19)		Total (*n* = 49)	
	Any grade	Grade 3 or 4	Any grade	Grade 3 or 4	Any grade	Grade 3 or 4	Any grade	Grade 3 or 4
Any adverse event	10 (100)	9 (90.0)	20 (100)	20 (100)	19 (100)	16 (84.2)	49 (100)	45 (91.8)
Hematologic								
Neutropenia	4 (40.0)	3 (30.0)	13 (65.0)	13 (65.0)	15 (78.9)	13 (68.4)	32 (65.3)	29 (59.2)
Lymphopenia	9 (90.0)	8 (80.0)	9 (45.0)	9 (45.0)	12 (63.2)	12 (63.2)	30 (61.2)	29 (59.2)
Anemia	5 (50.0)	0	8 (40.0)	5 (25.0)	6 (31.6)	1 (5.3)	19 (38.8)	6 (12.2)
Thrombocytopenia	3 (30.0)	1 (10.0)	7 (35.0)	2 (10.0)	8 (42.1)	2 (10.5)	18 (36.7)	5 (10.2)
Leukopenia	5 (50.0)	2 (20.0)	3 (15.0)	2 (10.0)	6 (31.6)	5 (26.3)	14 (28.6)	9 (18.4)
Non-hematologic								
CRS	6 (60.0)	0	14 (70.0)	0	13 (68.4)	0	33 (67.3)	0
Hypogammaglobulinemia	10 (100)	1 (10.0)	10 (50.0)	1 (5.0)	7 (36.8)	0	27 (55.1)	2 (4.1)
Pyrexia	7 (70.0)	1 (10.0)	10 (50.0)	2 (10.0)	7 (36.8)	0	24 (49.0)	3 (6.1)
Upper respiratory tract infection	6 (60.0)	0	8 (40.0)	1 (5.0)	6 (31.6)	0	20 (40.8)	1 (2.0)
Rash	5 (50.0)	2 (20.0)	5 (25.0)	0	8 (42.1)	0	18 (36.7)	2 (4.1)
Gamma-glutamyl transferase increased	3 (30.0)	0	6 (30.0)	3 (15.0)	5 (26.3)	4 (21.1)	14 (28.6)	7 (14.3)
Hypokalemia	1 (10.0)	0	9 (45.0)	2 (10.0)	5 (26.3)	0	15 (30.6)	2 (4.1)
Diarrhea	6 (60.0)	0	4 (20.0)	1 (5.0)	6 (31.6)	1 (5.3)	16 (32.7)	2 (4.1)
Nausea	1 (10.0)	0	4 (20.0)	0	9 (47.4)	1 (5.3)	14 (28.6)	1 (2.0)
Peripheral sensory neuropathy	1 (10.0)	0	5 (25.0)	0	4 (21.1)	0	10 (20.4)	0
Blood alkaline phosphatase increased	4 (40.0)	0	1 (5.0)	0	3 (15.8)	1 (5.3)	8 (16.3)	1 (2.0)
Lipase increased	1 (10.0)	1 (10.0)	5 (25.0)	3 (15.0)	1 (5.3)	1 (5.3)	7 (14.3)	5 (10.2)
Alanine aminotransferase increased	3 (30.0)	0	2 (10.0)	1 (5.0)	2 (10.5)	2 (10.5)	7 (14.3)	3 (6.1)
Nasopharyngitis	3 (30.0)	0	2 (10.0)	0	2 (10.5)	0	7 (14.3)	0
Hyperglycemia	3 (30.0)	0	3 (15.0)	1 (5.0)	0	0	6 (12.2)	1 (2.0)
Constipation	0	0	1 (5.0)	0	5 (26.3)	0	6 (12.2)	0

^a^Most common defined as incidence of ≥25% in any treatment arm.

^b^Teclistamab (1.5 mg kg^−1^ weekly), daratumumab and lenalidomide treatment group.

^c^Teclistamab (3.0 mg kg^−1^ monthly), daratumumab and lenalidomide treatment group.

^d^Teclistamab (3.0 mg kg^−1^ monthly), daratumumab, lenalidomide and bortezomib treatment group.

**Table 3:** Summary of the most common^a^ treatment-emergent infections

Event, *n* (%)	Arm A^b^ (*n* = 10)		Arm A1^c^ (*n* = 20)		Arm B^d^ (*n* = 19)		Total (*n* = 49)	
	Any grade	Grade 3 or 4	Any grade	Grade 3 or 4	Any grade	Grade 3 or 4	Any grade	Grade 3 or 4
Any treatment-emergent infection	10 (100)	4 (40.0)	18 (90.0)	10 (50.0)	12 (63.2)	4 (21.1)^e^	40 (81.6)	18 (36.7)^e^
Most common infections								
Upper respiratory tract infection	6 (60.0)	0	8 (40.0)	1 (5.0)	6 (31.6)	0	20 (40.8)	1 (2.0)
COVID-19	2 (20.0)	0	4 (20.0)	1 (5.0)	4 (21.1)	2 (10.5)	10 (20.4)	3 (6.1)
Nasopharyngitis	3 (30.0)	0	2 (10.0)	0	2 (10.5)	0	7 (14.3)	0
Pneumonia	1 (10.0)	1 (10.0)	0	0	3 (15.8)	2 (10.5)	4 (8.2)	3 (6.1)
Viral upper respiratory tract infection	0	0	3 (15.0)	1 (5.0)	1 (5.3)	0	4 (8.2)	1 (2.0)
Respiratory tract infection	0	0	1 (5.0)	0	2 (10.5)	0	3 (6.1)	0
Infection	0	0	1 (5.0)	1 (5.0)	2 (10.5)	1 (5.3)	3 (6.1)	2 (4.1)
Bronchitis	2 (20.0)	0	0	0	0	0	2 (4.1)	0

^a^Most common defined as incidence of >10% in any treatment arm.

^b^Teclistamab (1.5 mg kg^−1^ weekly), daratumumab and lenalidomide treatment group.

^c^Teclistamab (3.0 mg kg^−1^ monthly), daratumumab and lenalidomide treatment group.

^d^Teclistamab (3.0 mg kg^−1^ monthly), daratumumab, lenalidomide and bortezomib treatment group.

^e^One patient had a grade 3 ‘unknown’ infection that was reported under the ‘uncoded’ category.

Cytokine release syndrome (CRS) occurred in 33 patients (67.3%; Extended Data Table 2), with most events occurring during the step-up dose schedule. Per the American Society for Transplantation and Cellular Therapy (ASTCT) CRS revised grading system^16^, all CRS events were grade 1 or 2, all resolved and none led to discontinuation of study treatment. Supportive measures for treatment of CRS are summarized in Extended Data Table 3. Thirty patients (61.2%) reported at least one treatment-emergent neurologic adverse event of any grade, most of which were low severity (maximum toxicity grade 1 or 2, 57.1%; grade 3, 4.1%). The most common was peripheral sensory neuropathy (10 patients (20.4%); total of 12 events), all of which were grade 1 or 2. Across individual arms, peripheral sensory neuropathy was reported in one of 10 patients (10.0%; maximum toxicity grade 1) in arm A, in five of 20 patients (25.0%; maximum toxicity grade 1, *n* = 5) in arm A1 and in four of 19 patients (21.1%; maximum toxicity grade 1, *n* = 1; grade 2, *n* = 3) in arm B. No treatment-related ICANS events occurred. Furthermore, no events of cranial nerve palsies, acute demyelinating neuropathies, transverse myelitis or movement/neurocognitive toxicities were reported.

Serious TEAEs occurred in 27 patients (55.1%; Extended Data Table 4). Serious infections occurred in 16 patients (32.7%). For the individual treatment components, by the premaintenance treatment timepoint, one patient had discontinued teclistamab (arm A1), four patients (arm A1, *n* = 1; arm B, *n* = 3) had discontinued lenalidomide and three patients (all in arm B) had discontinued bortezomib due to onset of TEAEs; however, no TEAEs led to the discontinuation of all study treatments in the complete regimen (that is, Tec-DR or Tec-DVR).

### Efficacy

The efficacy analysis set comprised 10, 20 and 19 patients in arms A, A1 and B, respectively. Sample collection for MRD testing was scheduled after induction cycles 3 and 6 and premaintenance using next-generation flow cytometry (NGF, 1 × 10^−5^) and, in addition, after cycle 6 using next-generation sequencing (NGS, 1 × 10^−6^). When combining all patients (*n* = 49), the cumulative MRD-negative CR rate according to IMWG criteria was 91.8% (arm A, *n* = 10/10; arm A1, *n* = 18/20; arm B, *n* = 17/19). Samples for MRD testing were available for 48 of 49 patients (arm A, *n* = 10/10; arm A1, *n* = 20/20; arm B, *n* = 18/19), all of which were MRD negative by NGF, resulting in a cumulative MRD negativity rate of 98.0%. One patient (arm B) was not evaluable for MRD at any timepoint due to discontinuation before the end of cycle 3.

**Fig. 2:**
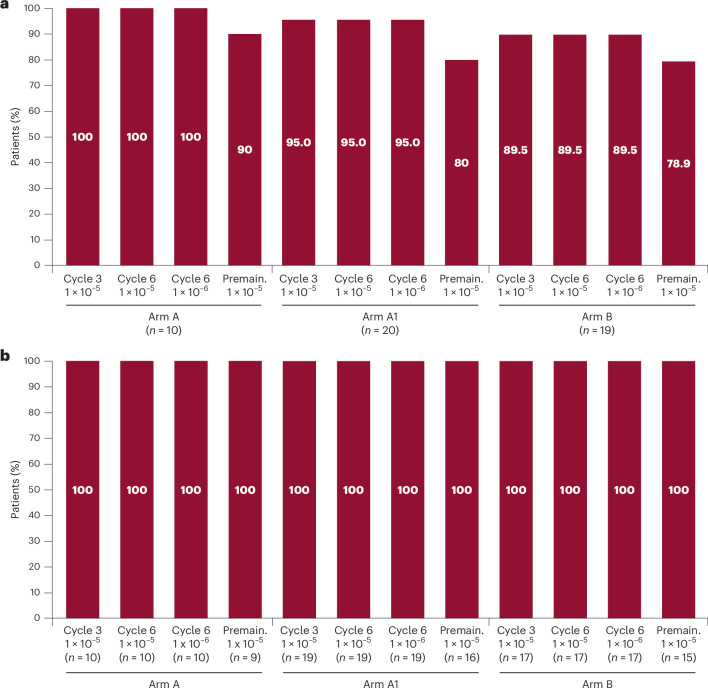
Summary of overall MRD negativity rate in the efficacy analysis set and the MRD-evaluable analysis set, respectively, by the premaintenance visit. Patients in arm A and arm A1 received teclistamab (1.5 mg kg^−1^ weekly or 3.0 mg kg^−1^ monthly, respectively) with daratumumab and lenalidomide; patients in arm B received teclistamab (3.0 mg kg^−1^ monthly) with daratumumab, lenalidomide and bortezomib. Panel **a** shows MRD negativity at the 1 × 10^−5^ threshold and the 1 × 10^−6^ threshold, assessed by NGF and NGS, respectively, in the efficacy analysis set (defined as all enrolled patients who received at least one dose of study treatment). After cycle 3, three patients were not tested for MRD at the 1 × 10^−5^ threshold with NGF (arm A1, *n* = 1; arm B, *n* = 2). After cycle 6, two patients were not tested for MRD at the 1 × 10^−5^ threshold with NGF (arm A1, *n* = 1; arm B, *n* = 1), and one patient (arm B) had an insufficient number of cells to be assessed by NGF. For MRD at the 1 × 10^−6^ threshold with NGS after cycle 6, two patients were not tested (arm A1, *n* = 1; arm B, *n* = 1), and one patient (arm B) had no baseline clone detected. Of note, patients may have been evaluable for MRD by NGF and/or NGS. For premaintenance, eight patients were not tested for MRD at the 1 × 10^−5^ threshold (arm A, *n* = 1; arm A1, *n* = 3; arm B, *n* = 4), and one patient (arm A1) was not evaluable. Panel **b** shows MRD negativity at the 1 × 10^−5^ threshold and the 1 × 10^−6^ threshold, assessed by NGF and NGS, respectively, in the MRD-evaluable analysis set (defined as those patients with an available MRD test with a positive or negative result). Of note, patients may have been evaluable for MRD by NGF and/or NGS. Premain., premaintenance.

Based on the efficacy analysis set (that is, intent-to-treat), MRD negativity rates after induction cycle 3 were 100%, 95.0% and 89.5% in arms A, A1 and B, respectively (Fig. 2a and Extended Data Table 5), with all tested samples being MRD negative (Fig. 2b and Extended Data Table 6). Three patients (arm A1, *n* = 1; arm B, *n* = 2) were not tested (see Supplementary Table 1 for comprehensive overview). These findings were confirmed after induction cycle 6 (Fig. 2); two patients (arm A1, *n* = 1; arm B, *n* = 1) were not tested, and one patient (arm B) was indeterminate (Extended Data Table 5 and Supplementary Table 1). Notably, NGS further confirmed MRD negativity after induction cycle 6 at the 1 × 10^−6^ threshold (Fig. 2); two patients (arm A1, *n* = 1; arm B, *n* = 1) were not tested by NGS, and one patient (arm B) had no baseline clone detected (Extended Data Table 5 and Supplementary Table 1). No MRD conversion events were seen after ASCT prior to maintenance, with MRD negativity rates (1 × 10^−5^) of 90.0%, 80.0% and 78.9% in arms A, A1 and B, respectively (Fig. 2a and Extended Data Table 5). At this timepoint, eight patients were not tested (arm A, *n* = 1; arm A1, *n* = 3; arm B, *n* = 4), and one patient (arm A1) was indeterminate (Extended Data Table 5 and Supplementary Table 1).

**Fig. 3:**
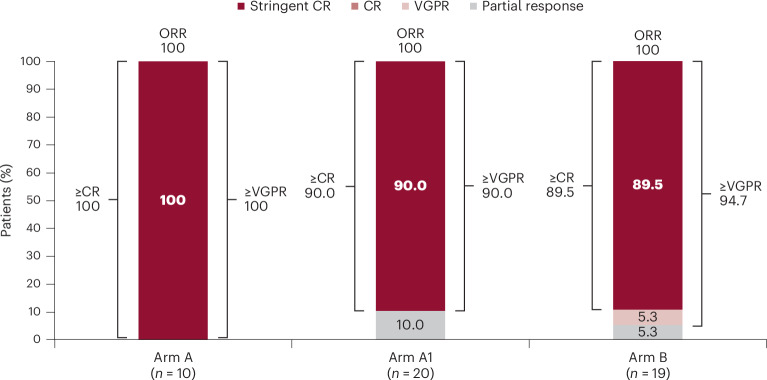
Overall response by the premaintenance visit. ORR was assessed as a stringent CR, CR, VGPR or partial response. Patients in arms A and A1 received teclistamab (1.5 mg kg^−1^ weekly or 3.0 mg kg^−1^ monthly, respectively) with daratumumab and lenalidomide; patients in arm B received teclistamab (3.0 mg kg^−1^ monthly) with daratumumab, lenalidomide and bortezomib. Two patients in arm A1 had a partial response at the end of induction, as the sum of products of the perpendicular diameters of the soft tissue plasmacytoma did not decrease by more than 90% compared with baseline; one patient in arm B had a partial response at cycle 3 and then withdrew consent before completing cycle 3. One patient in arm B had a VGPR due to the presence of M-protein at the end of induction (positive immunofixation). Note that some values may not add to 100% due to rounding.

ORR was 100% across all arms (arm A, *n* = 10/10; arm A1, *n* = 20/20; arm B, *n* = 19/19; Fig. 3 and Extended Data Table 7). A ≥CR was achieved by 10 of 10 patients (100%), by 18 of 20 patients (90.0%) and by 17 of 19 patients (89.5%) in arms A, A1 and B, respectively (Extended Data Table 7). Among the four patients who did not achieve ≥CR, VGPR was observed in one patient (arm B) due to persistent M-protein by immunofixation (immunoglobulin G type) at the end of induction, and partial response was observed in three patients (arm A1, *n* = 2 due to insufficient reduction of paraskeletal soft tissue plasmacytomas; arm B, *n* = 1 partial response at cycle 3 followed by refusal of further study treatment and follow-up).

With a median follow-up of 12.3 months (range, 3.1–14.5) for the combined arms, no progression events have been observed.

Of all 49 patients who received at least one study treatment dose, 47 (95.9%) underwent successful stem cell mobilization, among whom 45 (95.7%) received a combination of cyclophosphamide and granulocyte colony-stimulating factor (G-CSF) and 20 (42.6%) received plerixafor for mobilization. One patient did not undergo mobilization due to withdrawal of consent after cycle 3, and one patient did not proceed to mobilization due to cytopenia and insufficient circulation of CD34^+^ cells. Overall total median CD34^+^ stem cell yield was 8.1 × 10^6^ per kg (range, 2.6–15.9), surpassing the per-protocol minimal and ideal target numbers (2.5 × 10^6^ per kg and 5 × 10^6^ per kg CD34^+^ cells, respectively). Of the 47 patients who underwent successful stem cell mobilization, 43 (87.8%) underwent ASCT. Harvested stem cells led to a recovery after high-dose therapy and ASCT, with an overall median time to neutrophil and platelet engraftment of 12 days and 14 days, respectively.

## Discussion

Advancements in frontline multiple myeloma treatment have led to substantial improvements in patient outcomes; however, there remains opportunity for further improvement. In this analysis of GMMG-HD10/DSMM XX (MajesTEC-5) by the premaintenance treatment period, Tec-DR, with or without bortezomib, demonstrated a safety profile consistent with what is known for each individual regimen component that can be managed with established protocols. Furthermore, we observed notable early clinical efficacy, with an overall response of 100% across all arms, 91.8% of all patients achieving MRD-negative CR and 100% of all evaluable patients achieving MRD-negative status at 1 × 10^−5^ (postinduction cycles 3 and 6 and premaintenance) and the more stringent 1 × 10^−6^ threshold (postinduction cycle 6).

The Tec-DR-based induction regimens explored here were feasible, with no discontinuations of all study treatments in the regimen due to the onset of any TEAEs; dose reductions and permanent discontinuations of lenalidomide, however, were reported, mainly due to adverse events. Overall, observed adverse events were primarily hematologic, with neutropenia being among the most common, consistent with the known safety profile of teclistamab-based, daratumumab-based and lenalidomide-based therapies^2,6,17^. The rate of grade 3 or 4 infections observed with Tec-DR, with or without bortezomib, was higher than that reported in early GRIFFIN study analyses with daratumumab plus lenalidomide/bortezomib/dexamethasone (D-RVd) as induction treatment (36.7% versus 23.2% (median follow-up, 22.1 months), respectively)^18^. However, differing patient cohorts, sample sizes and treatment schedules, and the relatively short follow-up period of the present study should be considered when making cross-trial comparisons. Nonetheless, the infection profile was more favorable than that observed in later lines^12,19^. Notably, no grade 5 infections were reported in MajesTEC-5. Overall, 44 patients (89.8%) received at least one dose of intravenous immunoglobulin treatment; however, differentiation between prophylactic use and continuous use to maintain immunoglobulin levels was not captured. Similar to that observed in other trials of bispecific antibodies, results emphasize the importance of early infection prevention and treatment protocols in patients receiving bispecific antibody treatment for multiple myeloma, including close patient monitoring, broad antimicrobial prophylaxis and the prophylactic use of immunoglobulin replacement per IMWG guidelines^20^. Such prophylactic strategies have been shown in real-world practice to reduce the frequency of high-grade infections with BCMA-targeting bispecific antibodies in patients with multiple myeloma^21^.

CRS, also commonly observed with T-cell-redirecting therapies, occurred in 67.3% of patients, predominantly during the step-up dosing schedule. All CRS events observed here were of low severity (grade 1 or 2) and were similar to those reported early in MajesTEC-1 (70%)^22^ and real-world clinical practice (54–64%)^23–25^. This absence of high-grade CRS events, observed even with the limited use of corticosteroids and tocilizumab, may have been due to effective mitigation strategies such as step-up dosing of teclistamab, premedication and dosing strategies such as the sparing of lenalidomide in the first cycle and introduction at cycle 2.

Notably, this safety profile was observed without the use of continuous dexamethasone treatment, which, given steroid-related toxicities, may help improve patient quality of life^26^. Furthermore, although the addition of bortezomib did not lead to any additional toxicity, its additional efficacy effects remain unclear at this timepoint because overall responses were equally deep across all arms. However, patients with a very high tumor burden, high-risk factors and potentially high soluble BCMA levels may benefit from the addition of bortezomib^11,27^. Longer follow-up is needed to gain further insight into the role of bortezomib in novel frontline combinations.

As reported here, Tec-DR, with or without bortezomib, and high-dosage melphalan ASCT resulted in a ≥CR rate of 91.8% (45 of 49 patients collectively), all of which met stringent CR criteria. At the most appropriate comparative treatment timepoints (after four induction cycles, transplant and two consolidation cycles) in the PERSEUS and GRIFFIN studies of daratumumab-based therapies for transplant-eligible disease, ≥CR rates were 44.5% and 51.5%, respectively^1,18^. Response rates shown here were also greater than those reported in the GMMG-HD7 study in a similar patient population (CR rate, 43.5%) after three 6-week induction cycles of an isatuximab-based therapy and ASCT^28^. Moreover, 100% of the MRD-evaluable population in MajesTEC-5 achieved MRD negativity per NGF (1 × 10^−5^) and NGS (1 × 10^−6^) across all arms, with 91.8% of all patients achieving MRD-negative CR. These MRD negativity rates surpass those observed after consolidation in PERSEUS (per NGS with ≥CR: 1 × 10^−5^, 57.5%; 1 × 10^−6^, 34.4%)^1^ and GRIFFIN (per NGS 1 × 10^−5^, 47.1%)^18^, albeit rates increased with time in these studies and after ASCT in GMMG-HD7 (per NGF 1 × 10^−5^, 66.2%; 1 × 10^−5^ with CR, 38.1%)^28^. Of note, cross-trial comparisons should be made with caution given differences in patient characteristics, study treatments, timepoints of comparison and MRD testing and duration of follow-up. Nevertheless, given the association between achieving deep and early MRD negativity and long-term survival outcomes^29^, the MRD negativity rates observed here are strong and support the feasibility of Tec-DR, with or without bortezomib, as induction therapy. Furthermore, if these results were to translate into sustained long-term outcomes, such as sustained (≥24-month) MRD negativity, there may be potential to reassess the duration of treatment or role of transplant in NDMM, thus informing future frontline treatment strategies^30^. Sustained MRD negativity was not evaluable at the premaintenance timepoint due to insufficient follow-up, and, as such, longer observation is required.

These promising results observed with the combination of teclistamab and daratumumab may be attributed to their potentially synergistic mechanisms of action^9,31^. In addition to its direct on-tumor activity, the immunomodulatory action of daratumumab depletes immunosuppressive CD38^+^ cells and induces helper and cytotoxic T cell expansion^32^. Teclistamab engages and activates T cells via its CD3-binding domain while targeting BCMA on myeloma cells, facilitating potent T-cell-mediated cytotoxicity^9^. Therefore, as shown preclinically^9^, daratumumab creates a more sensitive immune microenvironment, augmenting the cytotoxic activity of teclistamab, thus resulting in durable and sustained antimyeloma activity. Lenalidomide, a primary component of first-line standard-of-care NDMM therapy^33^ with T-cell-activating immunomodulatory effects, has demonstrated favorable efficacy and safety when combined with other antimyeloma agents^2,28,34^. Thus, combining teclistamab, daratumumab and lenalidomide, as explored here, may serve as a promising approach to further enhance and optimize the targeting and eradication of myeloma cells.

Several limitations should be taken into consideration. As this is a phase 2 study, the lack of a comparator arm limits the ability to compare treatment effect to that of non-Tec-containing combinations. Furthermore, although safety analyses are important to identify initial safety considerations, follow-up time was relatively short, thus emphasizing the need for continued follow-up. Additionally, our study population was predominantly White, limiting racial generalizability. Enrolled patients were also relatively fit, with no patients presenting with true extramedullary soft tissue plasmacytomas, a subgroup characterized by reduced efficacy and poorer long-term outcomes, albeit relatively rare (0.5–4.8%) in patients with NDMM^35^. Teclistamab-based combinations, however, have shown promise in patients with high-risk features, including extramedullary disease, as shown in the recent MajesTEC-3 study in which Tec-Dara resulted in progression-free and overall survival benefits across all high-risk subgroups in early-line relapsed refractory multiple myeloma^12,36^. Of note, despite limitations, MajesTEC-5 is, to our knowledge, the first study to evaluate the Tec-DR combination, with or without bortezomib, in NDMM. The observed favorable efficacy with deep MRD responses in all patients, including those with high-risk features, may increase the opportunity for patients to achieve long-term clinical benefit.

Collectively, the positive early clinical efficacy and manageable safety profile observed here in this premaintenance analysis of the ongoing GMMG-HD10/DSMM-XX (MajesTEC-5) study supports the feasibility of the innovative Tec-DR combination, with or without bortezomib, as a promising immune-based, steroid-sparing frontline induction treatment for NDMM.

## Methods

### Study design and oversight

This is an ongoing, multicenter, open-label, nonrandomized, multicohort phase 2 study (NCT05695508). The study was conducted in accordance with the principles of the Declaration of Helsinki, the International Council for Harmonisation guidelines for Good Clinical Practice and any country-specific regulations. The study protocol and relevant documents were approved at each site by an independent ethics committee (Ethikkommission der Medizinischen Fakultät Heidelberg (Ethics Committee of the Medical Faculty Heidelberg)) and institutional review board (Paul Ehrlich Institut, Bundesinstitut für Impfstoffe und biomedizinische Arzneimittel (Federal Institute for Vaccines and Biomedicinal Drugs)). All protocol amendments were similarly approved by the independent ethics committee or institutional review board at each study site and are outlined online in the protocol, which is provided along with the statistical analysis plan in Supplementary Information. All patients provided written informed consent. A study safety committee was established to monitor safety, inclusive of two independent external medical experts in the relevant therapeutic area.

The study was sponsored by the Heidelberg University Hospital, conducted in collaboration with the German-Speaking Myeloma Multicenter Group (GMMG), Deutsche Studiengruppe Multiples Myelom (DSMM) and Johnson & Johnson. The sponsor and investigators designed the study and compiled, maintained and analyzed the data that were collected by investigators. The sponsor and all authors vouch for the integrity of the data and that the study was conducted per study protocol. All authors reviewed, revised and approved the paper before submission.

### Patients

Patients were enrolled from 11 sites in Germany between 13 December 2022 and 6 March 2023, in arm A, and between 17/24 October 2023 and 14 March 2024, in arm A1/arm B, respectively. Enrolled patients were 18–70 years of age, had documented NDMM per IMWG diagnostic criteria^13^, were eligible for high-dose therapy and ASCT and had an Eastern Cooperative Oncology Group (ECOG) performance status score of 0–2. Sex was collected and reported in the study; sex was reported by the patient. Total number and percent of male and female patients recruited across arms are provided. A disaggregated analysis by sex was not prespecified in the trial protocol or statistical analysis plan and was not performed as sex was not considered a major confounder in the NDMM field.

Patients had a hemoglobin level of ≥7.5 g dl^−1^ (≥4.65 mmol l^−1^, without prior red blood cell transfusion within 7 days before the laboratory test), a platelet count of ≥75 × 10^9^ per liter (if <50% of bone marrow nucleated cells were plasma cells; otherwise, the platelet count was ≥50 × 10^9^ per liter) and an absolute neutrophil count of ≥1.0 × 10^9^ per liter (prior growth factor support was permitted but must have been without G-CSF or granulocyte–macrophage colony‑stimulating factor (GM-CSF) support for 7 days or without pegylated-G-CSF for 14 days). In addition, patients had aspartate and alanine aminotransferase levels of ≤2.5 times the upper limit of normal, an estimated glomerular filtration rate of ≥30 ml min^−1^ (based on Cockroft–Gault formula or 24-hour urine collection), a total bilirubin level ≤2.0 times the upper limit of normal, and a corrected serum calcium level of ≤14 mg dl^−1^ (≤3.5 mmol l^−1^) or free ionized calcium of ≤6.5 mg dl^−1^ (≤1.6 mmol l^−1^). Excluded were patients with prior or current systemic therapy or stem cell transplant (exception of a short course of corticosteroids); prior radiotherapy or focal radiation (within 14 days or 7 days, respectively, of enrollment); prior plasmapheresis (within 28 days of enrollment); prior or concurrent invasive malignancy (other than multiple myeloma); central nervous system involvement or clinical signs of meningeal involvement of multiple myeloma; seropositive for HIV, hepatitis B infection or hepatitis C infection (or received a live, attenuated vaccine within 28 days of first study dose); chronic obstructive pulmonary disease with a forced expiratory volume in 1 second <50% of predicted normal; moderate or severe persistent asthma within the past 2 years or currently uncontrolled asthma; grade 2 or higher peripheral neuropathy or neuropathic pain; concurrent medical or psychiatric condition or disease (likely to interfere with study procedures); cardiac conditions; contraindications or life-threatening allergies; hypersensitivity or intolerance to any study drug or its excipients; prior traumatic injury or surgery (within 14 days of enrollment); received an investigational drug or investigational medical device; or gastrointestinal disease (impacting absorption of oral drugs).

### Study treatments

This was an unblinded, nonrandomized study. Arm A was the first cohort to open, and, after completing enrollment, patients were assigned to arm A1 or arm B in parallel. Induction treatment consisted of six 28-day cycles. During cycle 1, all patients in arms A, A1 and B received subcutaneous teclistamab at the recommended phase 2 dose of 1.5 mg kg^−1^ on days 8 and 15, preceded by step-up doses of 0.06 mg kg^−1^ and 0.3 mg kg^−1^; patients in arm A received an additional dose of teclistamab at 1.5 mg kg^−1^ on day 22 (refs. ^4,5^). In cycles 2 through 6, teclistamab was given at a dose of 1.5 mg kg^−1^ weekly in arm A and at a dose of 3.0 mg kg^−1^ every 4 weeks in arms A1 and B. Additionally, all patients received subcutaneous daratumumab (1,800 mg) weekly in cycles 1 and 2 and every 2 weeks in cycles 3 through 6 and oral lenalidomide (25 mg on days 1–21 of each cycle from cycle 2 onwards). Patients in arm B received, in addition, subcutaneous bortezomib at a dose of 1.3 mg m^−^^2^ weekly in cycles 1 through 6. Oral or intravenous dexamethasone at a dose of 20 mg was administered in cycles 1 and 2 in arms A1 and B and in cycles 1 through 4 in arm A only.

After completion of induction cycle 3, stem cell mobilization was performed per local standard of care, with use of plerixafor and other standard agents, such as cyclophosphamide and G-CSF, permitted to ensure adequate mobilization. Of note, the use of cyclophosphamide for mobilization remains standard practice in Germany, in which all study sites reside at the time of this analysis. Conditioning therapy with melphalan (200 mg m^−^^2^) was recommended 24–48 hours prior to ASCT.

Patients who completed induction and received transplant went on to receive teclistamab–daratumumab maintenance therapy. Although maintenance phase results are out of scope for this current analysis, additional dosing information regarding this phase is provided in the protocol.

Any patient who experienced a grade 2 or higher CRS deemed related to study drug received dexamethasone 20 mg, diphenhydramine 25–50 mg or equivalent and acetaminophen 650–1,000 mg or equivalent as pretreatment medication for at least the subsequent dose of the study drug to which the event was related. Prophylactic tocilizumab was not recommended per protocol; however, tocilizumab was considered for grade 1 and recommended for grade 2 or higher CRS events.

### Endpoints and assessments

The analysis period reported in this paper is from the first dose of study treatment to the last visit prior to the start of maintenance (premaintenance treatment period). The primary endpoint was the incidence and severity of adverse events and serious adverse events over the entire treatment phase (premaintenance treatment period). Prespecified secondary efficacy endpoints reported here include MRD-negative CR rate, MRD negativity rates (at 1 × 10^−5^ and 1 × 10^−6^ sensitivity thresholds), ORR (partial response or better), ≥VGPR, ≥CR and stem cell yield and days to engraftment. MRD negativity was defined, regardless of response, as achieving MRD-negative status by NGF with a sensitivity of 1 × 10^−5^ after the date of first dose of the study medication and prior to progressive disease, subsequent therapy or both. MRD-negative status was also assessed by NGS with a sensitivity of 1 × 10^−6^. MRD-negative CR was defined as achieving MRD negativity (per NGF at 1 × 10^−5^) and ≥CR at any time after the date of first dose of the study medication and prior to progressive disease, subsequent therapy or both. ORR, ≥CR and ≥VGPR were defined as the proportion of patients who achieved the respective response per IMWG criteria by the premaintenance treatment period.

Additional secondary endpoints that are not reported at this time include duration of response, sustained (≥12-month) MRD-negative CR rate (1 × 10^−5^), progression-free survival, pharmacokinetic parameters and immunogenicity.

All disease evaluations were performed at a central laboratory on day 1 of every treatment cycle and at the end of induction and premaintenance visits. Tumor response was assessed by investigators based on IMWG consensus criteria for response^13–15^. Bone marrow aspirates after induction cycles 3 and 6 and premaintenance were assessed centrally for MRD by NGF (EuroFlow) at the 1 × 10^−5^ sensitivity threshold. Additionally, aspirates collected after cycle 6 were assessed by NGS (clonoSEQ assay with a sensitivity of 1 × 10^−6^, version 2.0; Adaptive Biotechnologies). Due to the extended half-life of immunoglobulin heavy chains, MRD diagnostics were performed with NGF (at 1 × 10^−5^) and NGS (at 1 × 10^−6^) irrespective of response and the presence of M-protein. Bone marrow aspirates were also used to assess cytogenetic risk by central fluorescence in situ hybridization or local fluorescence in situ hybridization (if central analysis was unavailable). High cytogenetic risk was defined as the presence of one or more of the following abnormalities: del(17p), t(4;14) or t(14;16).

All adverse events were monitored and reported from the time a signed and dated informed consent form was obtained until 30 days after the last dose of study treatment or start of subsequent antimyeloma therapy, whichever occurred first. In patients presenting with persistent fever with undetermined cause (symptomatic cases), opportunistic infections including new onset or reactivation of viral infections, such as herpesviruses (that is, cytomegalovirus), parvovirus B19 and adenovirus, were assessed and reported. Routine testing for opportunistic infections was otherwise not mandated by protocol. Adverse events were graded according to National Cancer Institute Common Terminology Criteria for Adverse Events, version 5.0, except for CRS and ICANS, which were graded according to ASTCT guidelines^16^.

### Statistical analysis

This pooled data analysis of arms A, A1 and B was conducted to characterize safety and efficacy prior to the maintenance treatment period, because all patients have completed both the induction treatment phase and ASCT, specifically. Pooled arm reporting was permitted by the statistical analysis plan, with modifications permitted to make reporting more informative (that is, for interpreting adverse event patterns), to account for data availability and arms open for enrollment at different times and to evaluate additional combinations for exploratory purposes.

For arm A, approximately 10 patients were planned to be enrolled. For arms A1 and B, an initial enrollment of approximately 20 patients in each arm was planned, with the potential for expansion up to a total of 80 patients (arms A1 and B combined). No formal statistical hypothesis testing is planned in this study; all data are presented descriptively. Both the safety set and the efficacy analysis set consist of all patients who received at least one dose of study treatment. The MRD-evaluable analysis set consisted of all treated patients with an available sample per testing modality (NGF or NGS) that was either positive or negative, excluding those who were not tested, tested but sample is indeterminate, had no baseline clone detected (as it pertains to NGS) or with a missing sample. Response rates and MRD negativity rates, along with associated Clopper–Pearson exact 95% confidence intervals, were calculated. MRD negativity rates were calculated at the end of induction cycle 3, at the end of induction cycle 6 and at premaintenance treatment period. Outputs were generated using SAS software, version 9.4.

### Reporting summary

Further information on research design is available in the Nature Portfolio Reporting Summary linked to this article.

## Online content

Any methods, additional references, Nature Portfolio reporting summaries, source data, extended data, supplementary information, acknowledgements, peer review information; details of author contributions and competing interests; and statements of data and code availability are available at 10.1038/s41591-026-04471-x.

## Supplementary information

Supplementary Information List of investigators, Supplementary Table 1, CONSORT checklist, redacted protocol and redacted statistical analysis plan.

Reporting Summary

## Figures and Tables

**Extended Data Table 1:**
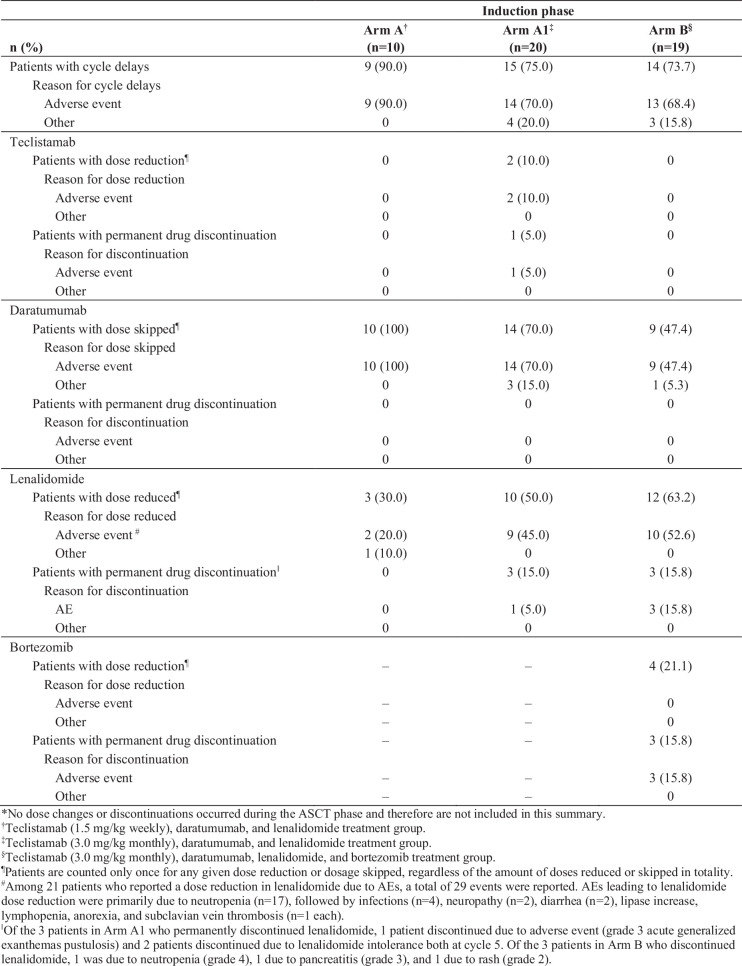
Summary of Dose Changes and Discontinuations During Induction Phase Only*

AE, adverse event.

*No dose changes or discontinuations occurred during the ASCT phase and therefore are not included in this summary.

^†^Teclistamab (1.5 mg kg^−1^ weekly), daratumumab and lenalidomide treatment group.

^‡^Teclistamab (3.0 mg kg^−1^ monthly), daratumumab and lenalidomide treatment group.

^§^Teclistamab (3.0 mg kg^−1^ monthly), daratumumab, lenalidomide and bortezomib treatment group.

^¶^Patients are counted only once for any given dose reduction or dosage skipped, regardless of the amount of doses reduced or skipped in totality.

^#^Among 21 patients who reported a dose reduction in lenalidomide due to AEs, a total of 29 events were reported. AEs leading to lenalidomide dose reduction were primarily due to neutropenia (*n* = 17), followed by infections (*n* = 4), neuropathy (*n* = 2), diarrhea (*n* = 2), lipase increase, lymphopenia, anorexia and subclavian vein thrombosis (*n* = 1 each).

^‖^Of the 3 patients in arm A1 who permanently discontinued lenalidomide, 1 patient discontinued due to adverse event (grade 3 acute generalized exanthemas pustulosis) and 2 patients discontinued due to lenalidomide intolerance both at cycle 5. Of the 3 patients in arm B who discontinued lenalidomide, 1 was due to neutropenia (grade 4), 1 due to pancreatitis (grade 3) and 1 due to rash (grade 2).

**Extended Data Table 2:**
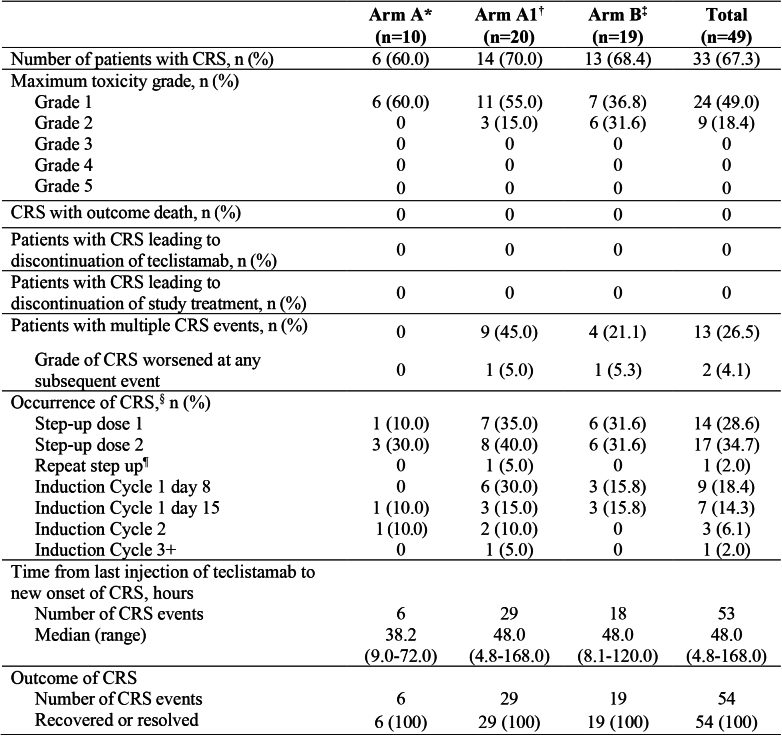
Summary of Treatment-Emergent CRS by the Pre-Maintenance Visit

CRS denotes cytokine release syndrome.

*Teclistamab (1.5 mg kg^−1^ weekly), daratumumab and lenalidomide treatment group.

^†^Teclistamab (3.0 mg kg^−1^ monthly), daratumumab and lenalidomide treatment group.

^‡^Teclistamab (3.0 mg kg^−1^ monthly), daratumumab, lenalidomide and bortezomib treatment group.

^§^Patients may appear in >1 category. Occurrence was based on the last treatment visit on or prior to the day in which the treatment-emergent adverse event occurred.

^¶^Prior to start of treatment.

**Extended Data Table 3:**
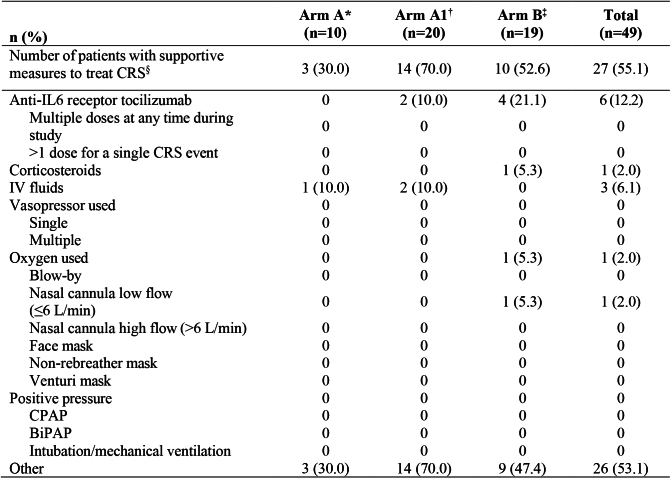
Summary of Supportive Treatments for CRS

BiPAP denotes bilevel positive airway pressure, CPAP continuous positive airway pressure, CRS cytokine release syndrome, IL interleukin and IV intravenous.

*Teclistamab (1.5 mg kg^−1^ weekly), daratumumab and lenalidomide treatment group.

^†^Teclistamab (3.0 mg kg^−1^ monthly), daratumumab and lenalidomide treatment group.

^‡^Teclistamab (3.0 mg kg^−1^ monthly), daratumumab, lenalidomide and bortezomib treatment group.

^§^Supportive measures to treat CRS and CRS symptoms are included.

**Extended Data Table 4:**
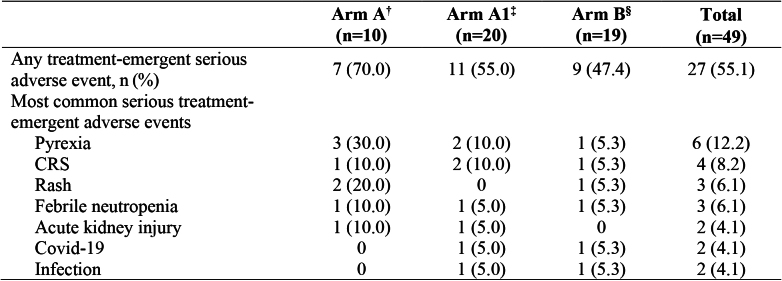
Summary of Most Common* Serious Treatment-Emergent Adverse Events by the Pre-Maintenance Visit

CRS denotes cytokine release syndrome.

*Most common defined as ≥2 patients in the combined arms A, A1 and B (total).

^†^Teclistamab (1.5 mg kg^−1^ weekly), daratumumab and lenalidomide treatment group.

^‡^Teclistamab (3.0 mg kg^−1^ monthly), daratumumab and lenalidomide treatment group.

^§^Teclistamab (3.0 mg kg^−1^ monthly), daratumumab, lenalidomide and bortezomib treatment group.

**Extended Data Table 5:**
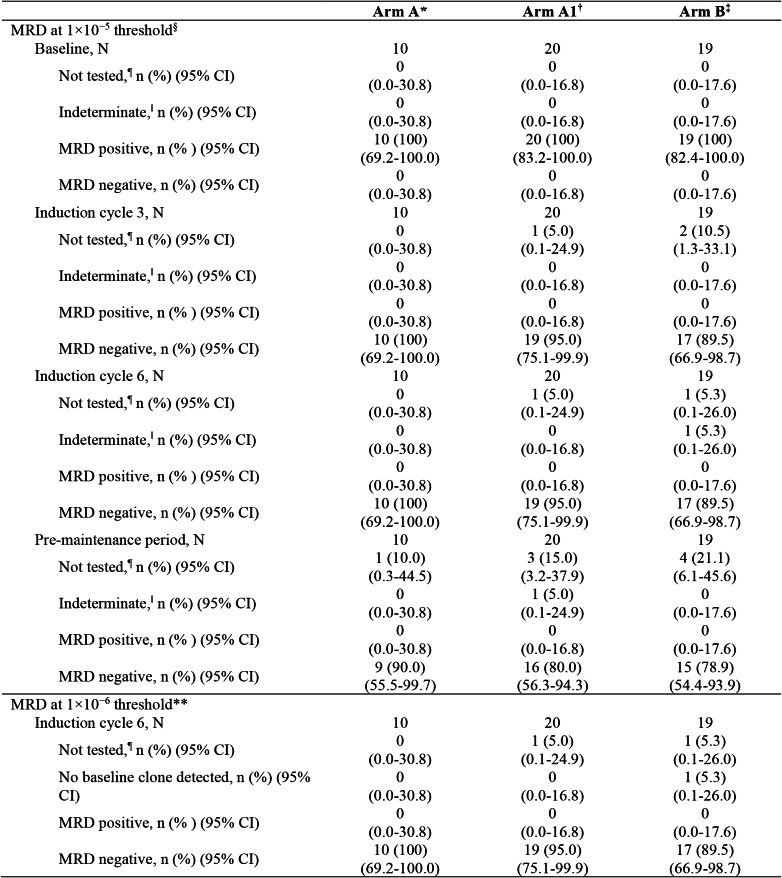
Summary of MRD Negativity in the Efficacy Analysis Set

CI denotes confidence interval, MRD minimal residual disease, NE not evaluable, NGF next-generation flow and NGS next-generation sequencing.

*Teclistamab (1.5 mg kg^−1^ weekly), daratumumab and lenalidomide treatment group.

^†^Teclistamab (3.0 mg kg^−1^ monthly), daratumumab and lenalidomide treatment group.

^‡^Teclistamab (3.0 mg kg^−1^ monthly), daratumumab, lenalidomide and bortezomib treatment group.

^§^MRD at the 1 × 10^−5^ threshold was assessed by NGF.

^¶^No MRD test taken in the given window.

^ǁ^MRD test with an indeterminate result.

**MRD at the 1 × 10^−6^ threshold was assessed by NGS.

**Extended Data Table 6:**
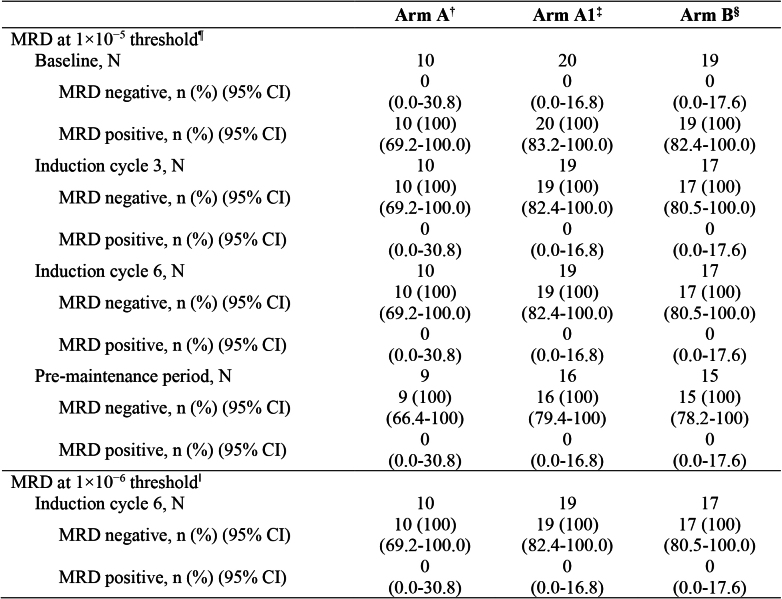
Summary of MRD Negativity in the MRD-Evaluable Analysis Set*

CI denotes confidence interval, MRD minimal residual disease, NE not evaluable, NGF next-generation flow and NGS next-generation sequencing.

*MRD-evaluable analysis set was defined as patients with an available MRD test with a positive or negative result (excluding those who were not tested, were indeterminate or had no baseline clone detected [NGS]).

^†^Teclistamab (1.5 mg kg^−1^ weekly), daratumumab and lenalidomide treatment group.

^‡^Teclistamab (3.0 mg kg^−1^ monthly), daratumumab and lenalidomide treatment group.

^§^Teclistamab (3.0 mg kg^−1^ monthly), daratumumab, lenalidomide and bortezomib treatment group.

^¶^MRD at the 1 × 10^−5^ threshold was assessed by NGF.

^ǁ^MRD at the 1 × 10^−6^ threshold was assessed by NGS.

**Extended Data Table 7:**
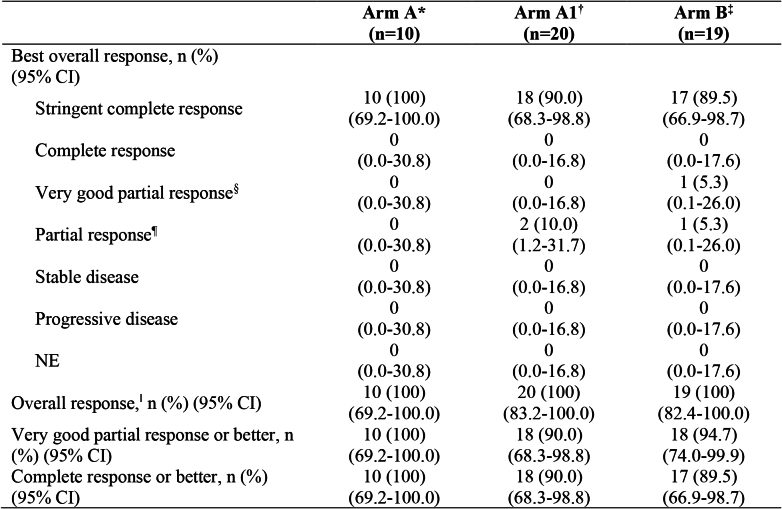
Summary of Best Overall Response By the Pre-Maintenance Visit

CI denotes confidence interval and NE not evaluable.

*Teclistamab (1.5 mg kg^−1^ weekly), daratumumab and lenalidomide treatment group.

^†^Teclistamab (3.0 mg kg^−1^ monthly), daratumumab and lenalidomide treatment group.

^‡^Teclistamab (3.0 mg kg^−1^ monthly), daratumumab, lenalidomide and bortezomib treatment group.

^§^One patient in arm B had a very good partial response due to the presence of M-protein at the end of induction (positive immunofixation).

^¶^Two patients in arm A1 had a partial response, both had a partial response by the pre-maintenance visit as the sum of products of the perpendicular diameters of the soft tissue plasmacytoma did not decrease by >90% compared with baseline; 1 patient in arm B had partial response at cycle 3 and then withdrew consent before completing cycle 3.

^‖^Overall response was defined as achieving a stringent complete response, complete response, very good partial response or partial response. Response was assessed by investigators based on International Myeloma Working Group criteria, encompassing timepoints up to the pre-maintenance treatment period.

## Data Availability

The Heidelberg University Hospital, in partnership with the GMMG, DSMM and Johnson & Johnson, will make the data available according to the data-sharing policy of Johnson & Johnson, which is available at https://innovativemedicine.jnj.com/our-innovation/clinical-trials/transparency. As noted on this site, requests for access to the study data can be submitted through the Yale Open Data Access (YODA) Project site at http://yoda.yale.edu.
