# Author Correction: Teclistamab-based induction treatment in transplant-eligible, newly diagnosed multiple myeloma: a phase 2 trial

**DOI:** 10.1038/s41591-026-04551-y

**Published:** 2026-07-06

**Authors:** Marc S. Raab, Niels Weinhold, K. Martin Kortüm, Jan Krönke, Roland Fenk, Katja Weisel, Lilli Podola, Uta Bertsch, Alexander Brobeil, Julia Mersi, Stefanie Huhn, Ryan Arlinghaus, Michael Hundemer, Stephan R. Bohl, Elias K. Mai, Natalie Schub, Johannes Waldschmidt, Florian Bassermann, Carsten Müller-Tidow, Christoph Heuck, Caline Sakabedoyan, Josephine Khan, Elena Ershova, Bas D. Koster, Monika Engelhardt, Mathias Hänel, Hans Salwender, Raphael Teipel, Hartmut Goldschmidt, Hermann Einsele, Leo Rasche

**Affiliations:** 1 Heidelberg Myeloma Center and GMMG Study Group, Department of Medicine V, Heidelberg University Hospital and Medical Faculty Heidelberg, Heidelberg, Germany; 2 Department of Internal Medicine II, University Hospital of Würzburg, Würzburg, Germany; 3 Department of Hematology, Oncology, and Tumor Immunology, Charité – Universitätsmedizin Berlin, corporate member of Freie Universität Berlin and Humboldt-Universität zu Berlin, Berlin, Germany; 4 Deutsches Konsortium für Translationale Krebsforschung (DKTK), Partner Site Berlin, Berlin, Germany; 5 Department of Hematology, Oncology and Clinical Immunology, University Hospital Düsseldorf, Medical Faculty, Heinrich Heine University Düsseldorf, Düsseldorf, Germany; 6 University Medical Center Hamburg-Eppendorf, Hamburg, Germany; 7 Institute of Pathology, University Hospital Heidelberg, Heidelberg, Germany; 8 Coordination Centre for Clinical Trials (KKS), University Hospital Heidelberg, Heidelberg, Germany; 9 Department of Internal Medicine II, Division of Stem Cell Transplantation and Immunotherapy, Universitätsklinikum Schleswig-Holstein (UKSH) Campus Kiel, Kiel, Germany; 10 Department of Medicine III, TUM Klinikum, Technische Universität München, München, Germany; 11 Deutsches Konsortium für Translationale Krebsforschung (DKTK), Deutsches Krebsforschungszentrum (DKFZ), Heidelberg, Germany; 12 Bayerisches Zentrum für Krebsforschung (BZKF), Munich, Germany; 13 Johnson & Johnson, Spring House, PA USA; 14 Johnson & Johnson, Paris, France; 15 Johnson & Johnson, High Wycombe, UK; 16 Johnson & Johnson, Beerse, Belgium; 17 Johnson & Johnson, Leiden, The Netherlands; 18 Department of Hematology, Oncology and Stem Cell Transplantation, Medical Centre - University of Freiburg, Faculty of Medicine, University of Freiburg, Freiburg, Germany; 19 Department of Internal Medicine III, Klinikum Chemnitz, Chemnitz, Germany; 20 Asklepios Tumorzentrum Hamburg, Asklepios Klinik Altona and Asklepios Klinik St. Georg, Hamburg, Germany; 21 Medizinische Klinik und Poliklinik I Universitätsklinikum Carl Gustav Carus an der Technische Universität Dresden, Dresden, Germany

**Keywords:** Myeloma, Cancer immunotherapy

Correction to: *Nature Medicine* 10.1038/s41591-026-04471-x, published online 25 June 2026.

In the version of this article initially published, there were typographical errors in Table 2, row 1, column 5, where “20 (100)” appeared originally as “19 (95.0),” and row 1, column 8, where “45 (98.1)” appeared originally as “44 (89.8),” and errors in Table 3, row 1, column 6, where “12 (63.2) appeared originally as “11 (57.9)” and in row 1, column 8, where “40 (81.6)” appeared originally as “39 (79.6).” The tables are now amended in the HTML and PDF versions of the article.

