# Supplementary material for: Teclistamab-based induction treatment in transplant-eligible, newly diagnosed multiple myeloma: a phase 2 trial
Source: Nat Med. 2026 Jun 25;32(7):2440–8. doi: 10.1038/s41591-026-04471-x (PMC13375549; doi:10.1038/s41591-026-04471-x)
Supplement: Supplementary file 1 — List of investigators, Supplementary Table 1, CONSORT checklist, redacted protocol and redacted statistical analysis plan. [file 41591_2026_4471_MOESM1_ESM.pdf]

nature medicine

Supplementary information https://doi.org/10.1038/s41591-026-04471-x

Teclistamab-based induction treatment in transplant-eligible, newly diagnosed multiple myeloma: a phase 2 trial

In the format provided by the authors and unedited

Table of Contents

List of Investigators 2

Supplementary Information Table S1. Summary of MRD Assessments and Best Responses Per Investigator Assessment by the Pre-Maintenance Visit 4

CONSORT Checklist 7

Study Protocol 12

Statistical Analysis Plan 277

List of GMMG-HD10/DSMM-XX (MajesTEC-5) Study Investigators

Marc S. Raab,[1] Niels Weinhold,[1] K. Martin Mortüm,[2] Jan Krönke,[3,4] Roland Fenk,[5] Katja Weisel,[6] Gunhild Mechtersheimer,[7] Julia Mersi,[2] Stephan R. Bohl,[3] Elias K. Mai,[1] Natalie Schub,[9] Johannes Waldschmidt,[2] Florian Bassermann,[10,11,12] Monika Engelhardt,[18] Mathias Hänel,[19] Hans Salwender,[20] Raphael Teipel,[21] Hartmut Goldschmidt,[1] Hermann Einsele,[2] Leo Rasche,[2] Jan Frenking,[1,22] Christine Riedhammer,[2] Franziska Panther,[2] Romans Zukovs,[5] Marion Högner,[10] Ralph Wäsch,[18] Dominik Zolnowski,[19] Christoph Schäfers,[23] Karolin Trautmann-Grill,[24] Hendrick Brockhoff,[25] Igor Blau,[26] Anna Jauch,[27]

[1]Heidelberg Myeloma Center and GMMG Study Group, Department of Medicine V, Heidelberg University Hospital and Medical Faculty Heidelberg, Heidelberg, Germany;

[2]Department of Internal Medicine II, University Hospital of Würzburg, Würzburg, Germany;

[3]Department of Hematology, Oncology, and Tumor Immunology, Charité – Universitätsmedizin Berlin, corporate member of Freie Universität Berlin and Humboldt-Universität zu Berlin, Berlin, Germany;

[4]Deutsches Konsortium für Translationale Krebsforschung (DKTK), Partner Site Berlin, Berlin, Germany;

[5]Department of Hematology, Oncology and Clinical Immunology, University Hospital Düsseldorf, Medical Faculty, Heinrich Heine University Düsseldorf, Düsseldorf, Germany;

[6]University Medical Center Hamburg-Eppendorf, Hamburg, Germany;

[7]Institute of Pathology, University Hospital Heidelberg, Heidelberg, Germany;

[9]Department of Internal Medicine II, Division of Stem Cell Transplantation and Immunotherapy, Universitätsklinikum Schleswig-Holstein (UKSH) Campus Kiel, Kiel, Germany;

[10]Department of Medicine III, TUM Klinikum, Technische Universität München, München, Germany;

[11]Deutsches Konsortium für Translationale Krebsforschung (DKTK), Deutsches Krebsforschungszentrum (DKFZ), Heidelberg, Germany;

[12]Bayerisches Zentrum für Krebsforschung (BZKF), Munich, Germany;

[18]Department of Hematology, Oncology and Stem Cell Transplantation, Medical Centre - University of Freiburg, Faculty of Medicine, University of Freiburg, Freiburg, Germany;

[19]Department of Internal Medicine III, Klinikum Chemnitz, Chemnitz, Germany;

[20]Asklepios Tumorzentrum Hamburg, Asklepios Klinik Altona and Asklepios Klinik St Georg, Hamburg, Germany;

[21]Medizinische Klinik und Poliklinik I Universitätsklinikum Carl Gustav Carus an der Technische Universität Dresden, Dresden, Germany;

[22]Clinical Cooperation Unit Molecular Hematology/Oncology, German Cancer Research Center (DKFZ), Heidelberg, Germany;

[23]Department of Oncology, Hematology and Bone Marrow Transplantation, Division of Pneumology, University Medical Center Hamburg-Eppendorf, Hamburg, Germany;

[24]Department of Internal Medicine I, University Hospital Dresden, Germany;

[25]Asklepios Tumorzentrum Hamburg, Asklepios Klinik Altona, Hamburg, Germany;

[26]Medical Clinic, Charité University Medicine Berlin, Germany;

[27]Institute of Human Genetics, University of Heidelberg, Heidelberg, Germany.

Supplementary Information Table S1. Summary of MRD Assessments and Best Response Per Investigator Assessment by the Pre-Maintenance Visit.

	Arm*	NGF (1×10^{-5})			NGS (1×10^{-6})	Best response per investigator assessment	Comments
		Cycle 3	Cycle 6	Pre-Main.	Cycle 6		
Pt 1	A	Negative	Negative	Not tested	Negative	sCR	Not tested for MRD at pre-main. by NGF due to refusal of bone marrow procedure
Pt 2	A	Negative	Negative	Negative	Negative	sCR	
Pt 3	A	Negative	Negative	Negative	Negative	sCR	
Pt 4	A	Negative	Negative	Negative	Negative	sCR	
Pt 5	A	Negative	Negative	Negative	Negative	sCR	
Pt 6	A	Negative	Negative	Negative	Negative	sCR	
Pt 7	A	Negative	Negative	Negative	Negative	sCR	
Pt 8	A	Negative	Negative	Negative	Negative	sCR	
Pt 9	A	Negative	Negative	Negative	Negative	sCR	
Pt 10	A	Negative	Negative	Negative	Negative	sCR	
Pt 11	A1	Negative	Negative	Negative	Negative	sCR	
Pt 12	A1	Negative	Negative	Negative	Negative	sCR	
Pt 13	A1	Negative	Negative	Negative	Negative	sCR	
Pt 14	A1	Negative	Negative	Negative	Negative	sCR	
Pt 15	A1	Negative	Negative	Negative	Negative	sCR	
Pt 16	A1	Negative	Negative	Negative	Negative	PR	PR at the end of induction as the sum of products of the perpendicular diameters of the soft tissue plasmacytoma did not decrease by >90% compared with baseline
Pt 17	A1	Negative	Not tested	Not tested	Not tested	sCR	Patient discontinued study participation after completing cycle 3 and thus was not tested for MRD thereafter
Pt 18	A1	Negative	Negative	Negative	Negative	sCR	
Pt 19	A1	Negative	Negative	Negative	Negative	sCR	
Pt 20	A1	Negative	Negative	Not tested	Negative	sCR	Bone marrow assessment was not conducted at pre-main. for MRD by NGF as patient became transplant

							ineligible due to multiple adverse events and skipped the ASCT phase
Pt 21	A1	Negative	Negative	Negative	Negative	sCR	
Pt 22	A1	Negative	Negative	Negative	Negative	sCR	
Pt 23	A1	Negative	Negative	Negative	Negative	sCR	
Pt 24	A1	Negative	Negative	Negative	Negative	sCR	
Pt 25	A1	Negative	Negative	Negative	Negative	PR	PR at the end of induction as the sum of products of the perpendicular diameters of the soft tissue plasmacytoma did not decrease by >90% compared with baseline
Pt 26	A1	Not tested	Negative	Negative	Negative	sCR	Not tested after cycle 3 due to site mistake
Pt 27	A1	Negative	Negative	Not evaluable	Negative	sCR	Patient sample was indeterminate at pre-main. by NGF
Pt 28	A1	Negative	Negative	Negative	Negative	sCR	
Pt 29	A1	Negative	Negative	Negative	Negative	sCR	
Pt 30	A1	Negative	Negative	Not tested	Negative	sCR	Bone marrow assessment at pre-main. was not conducted because patient refused from transplant and proceeded to maintenance without ASCT phase
Pt 31	B	Negative	Negative	Not tested	Negative	sCR	Patient had MRD performed on the next day after the start of maintenance, thus was not counted as part of the pre-maint. period
Pt 32	B	Negative	Negative	Negative	Negative	VGPR	VGPR due to the presence of M-protein at the end of induction (positive immunofixation). Note that this patient has missing assessments at the premaintenance visit in the data base, and per algorithm, response was sCR
Pt 33	B	Negative	Negative	Negative	Negative	sCR	
Pt 34	B	Negative	Negative	Negative	Not evaluable	sCR	Patient had no baseline clone detected for MRD by NGS
Pt 35	B	Negative	Negative	Negative	Negative	sCR	

Pt 36	B	Negative	Negative	Not tested	Negative	sCR	Not tested for MRD at pre-main. by NGF due to refusal of bone marrow procedure
Pt 37	B	Negative	Not evaluable	Negative	Negative	sCR	Patient was not evaluable (indeterminate) due to an insufficient number of cell numbers (negative at sensitivity of 1.4×10^{-5}) for NGF after cycle 6
Pt 38	B	Negative	Negative	Negative	Negative	sCR	
Pt 39	B	Negative	Negative	Negative	Negative	sCR	
Pt 40	B	Negative	Negative	Negative	Negative	sCR	
Pt 41	B	Negative	Negative	Negative	Negative	sCR	
Pt 42	B	Negative	Negative	Negative	Negative	sCR	
Pt 43	B	Negative	Negative	Not tested	Negative	sCR	Due to failed mobilization, ASCT phase was skipped thus bone marrow assessment was not conducted at pre-main.
Pt 44	B	Negative	Negative	Negative	Negative	sCR	
Pt 45	B	Not tested	Negative	Negative	Negative	sCR	Not tested after cycle 3 due site mistake
Pt 46	B	Negative	Negative	Negative	Negative	sCR	
Pt 47	B	Negative	Negative	Negative	Negative	sCR	
Pt 48	B	Not tested	Not tested	Not tested	Not tested	PR	Patient discontinued study participation before completing cycle 3, thus MRD was not evaluated; patient had PR at cycle 3 and then withdrew consent before completing cycle 3
Pt 49	B	Negative	Negative	Negative	Negative	sCR	

MRD denotes minimal residual disease, NGF next-generation flow, NGS next-generation sequencing, PR, partial response, sCR stringent complete response, and VGPR very good partial response.

*Patients in Arm A received teclistamab (1.5 mg/kg weekly), daratumumab, and lenalidomide treatment; patients in Arm A1 received teclistamab (3.0 mg/kg monthly), daratumumab, and lenalidomide treatment; patients in Arm B received teclistamab (3.0 mg/kg monthly), daratumumab, lenalidomide, and bortezomib treatment.

CONSORT 2025 checklist of information to include when reporting a randomised trial*

Disclaimer: Page numbers reflect the original manuscript submitted to *Nature Medicine* and may not align with the finalized, published (online or print) PDF layout

Section / Topic	No	CONSORT 2025 checklist item description	Reported on page no.
**Title and abstract**			
Title and structured abstract	1a	Identification as a randomised trial	N/A (non-randomized phase 2 study)
	1b	Structured summary of the trial design, methods, results, and conclusions	Page 5-6
**Open science**			
Trial registration	2	Name of trial registry, identifying number (with URL) and date of registration	Page 6, Page 8, Page 38
Protocol and statistical analysis plan	3	Where the trial protocol and statistical analysis plan can be accessed	Page 38
Data sharing	4	Where and how the individual de-identified participant data (including data dictionary), statistical code and any other materials can be accessed	N/A
Funding and conflicts of interest	5a	Sources of funding and other support (e.g., supply of drugs), and role of funders in the design, conduct, analysis and reporting of the trial	Page 19, Page 38
	5b	Financial and other conflicts of interest of the manuscript authors	Page 20-24
**Introduction**			
Background and rationale	6	Scientific background and rationale	Page 7-9

Objectives	7	Specific objectives related to benefits and harms	Page 8-9
**Methods**			
Patient and public involvement	8	Details of patient or public involvement in the design, conduct and reporting of the trial	N/A
Trial design	9	Description of trial design including type of trial (e.g., parallel group, crossover), allocation ratio, and framework (e.g., superiority, equivalence, non-inferiority, exploratory)	Page 38
Changes to trial protocol	10	Important changes to the trial after it commenced including any outcomes or analyses that were not prespecified, with reason	Page 38
Trial setting	11	Settings (e.g., community, hospital) and locations (e.g., countries, sites) where the trial was conducted	Page 39
Eligibility criteria	12a	Eligibility criteria for participants	Page 39-40
	12b	If applicable, eligibility criteria for sites and for individuals delivering the interventions (e.g., surgeons, physiotherapists)	N/A
Intervention and comparator	13	Intervention and comparator with sufficient details to allow replication. If relevant, where additional materials describing the intervention and comparator (e.g., intervention manual) can be accessed	Page 40-42
Outcomes	14	Pre-specified primary and secondary outcomes, including the specific measurement variable (e.g., systolic blood pressure), analysis metric (e.g., change from baseline, final value, time to event), method of aggregation (e.g., median, proportion), and time point for each outcome	Page 42-44
Harms	15	How harms were defined and assessed (e.g., systematically, non-systematically)	N/A
Sample size	16a	How sample size was determined, including all assumptions supporting the sample size calculation	Page 44
	16b	Explanation of any interim analyses and stopping guidelines	N/A
Randomisation:			
Sequence generation	17a	Who generated the random allocation sequence and the method used	N/A (non-randomized)

	17b	Type of randomisation and details of any restriction (e.g., stratification, blocking and block size)	N/A (non-randomized)
Allocation concealment mechanism	18	Mechanism used to implement the random allocation sequence (e.g., central computer/telephone; sequentially numbered, opaque, sealed containers), describing any steps to conceal the sequence until interventions were assigned	N/A (non-randomized)
Implementation	19	Whether the personnel who enrolled and those who assigned participants to the interventions had access to the random allocation sequence	N/A (non-randomized)
Blinding	20a	Who was blinded after assignment to interventions (e.g., participants, care providers, outcome assessors, data analysts)	N/A (non-blinded)
	20b	If blinded, how blinding was achieved and description of the similarity of interventions	N/A (non-blinded)
Statistical methods	21a	Statistical methods used to compare groups for primary and secondary outcomes, including harms	N/A (no formal statistical analysis [Page 44])
	21b	Definition of who is included in each analysis (e.g., all randomised participants), and in which group	Page 44
	21c	How missing data were handled in the analysis	N/A
	21d	Methods for any additional analyses (e.g., subgroup and sensitivity analyses), distinguishing prespecified from post-hoc	N/A
**Results**			
Participant flow, including flow diagram	22a	For each group, the numbers of participants who were randomly assigned, received intended intervention, and were analysed for the primary outcome	Page 9, Figure 1
	22b	For each group, losses and exclusions after randomisation, together with reasons	Figure 1
Recruitment	23a	Dates defining the periods of recruitment and follow-up for outcomes of benefits and harms	Page 39

	23b	If relevant, why the trial ended or was stopped	N/A (ongoing)
Intervention and comparator delivery	24a	Intervention and comparator as they were actually administered (e.g., where appropriate, who delivered the intervention/comparator, how participants adhered, whether they were delivered as intended [fidelity])	Page 40-42
	24b	Concomitant care received during the trial for each group	Page 41-42
Baseline data	25	A table showing baseline demographic and clinical characteristics for each group	Table 1
Numbers analysed,<br>outcomes and estimation	26	For each primary and secondary outcome, by group:<br>● the number of participants included in the analysis<br>● the number of participants with available data at the outcome time point<br>● result for each group, and the estimated effect size and its precision (such as 95% confidence interval)<br>● for binary outcomes, presentation of both absolute and relative effect size	Page 9, all data tables (including Extended Data Tables), effect size N/A (no formal statistical analyses)
Harms	27	All harms or unintended events in each group	Page 10-12; Table 2-3, Extended Data Table 2, Extended Data Table 4
Ancillary analyses	28	Any other analyses performed, including subgroup and sensitivity analyses, distinguishing pre-specified from post-hoc	N/A
**Discussion**			
Interpretation	29	Interpretation consistent with results, balancing benefits and harms, and considering other relevant evidence	Page 14-19

Limitations	30	Trial limitations, addressing sources of potential bias, imprecision, generalisability, and, if relevant, multiplicity of analyses	Page 18

*We strongly recommend reading this statement in conjunction with the CONSORT 2025 Explanation and Elaboration and/or the CONSORT 2025 Expanded Checklist for important clarifications on all the items. We also recommend reading relevant CONSORT extensions. See www.consort-spirit.org.

Citation: Hopewell S, Chan AW, Collins GS, Hróbjartsson A, Moher D, Schulz KF, et al. CONSORT 2025 Statement: updated guideline for reporting randomised trials. BMJ. 2025; 388:e081123. https://dx.doi.org/10.1136/bmj-2024-081123.

© 2025 Hopewell et al. This is an Open Access article distributed under the terms of the Creative Commons Attribution License (https://creativecommons.org/licenses/by/4.0/), which permits unrestricted use, distribution, and reproduction in any medium, provided the original work is properly cited.

Clinical Protocol

A Phase 2 Study to Evaluate Safety and Efficacy of Teclistamab- and Talquetamab-based Combination Regimens in Participants with Newly Diagnosed Transplant Eligible Multiple Myeloma

MajesTEC-5

Protocol GMMG-HD10/DSMM XX/64007957MMY2003 Amendment 5; Phase 2

JNJ-64007957; JNJ-64407564 (teclistamab; talquetamab)
Amendment 5

The term "sponsor" is used throughout the protocol. The sponsor is identified on the Contact Information page that accompanies the protocol.

Studies conducted at sites in the European Economic Area (EEA) will be conducted under Regulation [EU] No 536/2014.

EU TRIAL NUMBER: 2024-517382-17-00

Sponsor: Ruprecht-Karls-University Heidelberg, Medical Faculty represented by University Hospital Heidelberg
Sponsor representative: Commercial Managing Director PPD, executing lead investigator Prof. Dr. PPD

**Status:**	Approved
**Date:**	19 December 2024
**Prepared by:**	Janssen Research & Development, LLC
**EDMS number:**	EDMS-RIM-350759, 10.0

GCP Compliance: This study will be conducted in compliance with Good Clinical Practice, and applicable regulatory requirements.

Confidentiality Statement
The information provided herein contains Company trade secrets, commercial or financial information that the Company customarily holds close and treats as confidential. The information is being provided under the assurance that the recipient will maintain the confidentiality of the information under applicable statutes, regulations, rules, protective orders or otherwise.

PROTOCOL AMENDMENT SUMMARY OF CHANGES TABLE

DOCUMENT HISTORY	
**Document**	**Date**
Amendment 5	19 December 2024
Amendment 4	13 March 2024
Amendment 3	06 July 2023
Amendment 2	17 October 2022
Amendment 1	07 October 2022
Original Protocol	12 April 2022

Amendment 5 (19 December 2024)

Overall Rationale for the Amendment: The overall rationale for this amendment is to add information on [redacted] and to add management guidelines for neurological AE per a clinical development decision by the Sponsor following an urgent safety measure based on data from participants receiving talquetamab. In addition, updates were made to align with EU-CTR requirements and teclistamab program level language.

The changes made to the clinical protocol 64007957MMY2003 as part of Protocol Amendment 5 are listed below, including the rationale of each change and a list of all applicable sections. Changes made in previous protocol amendments are listed in Section 10.23 Appendix 23: Protocol Amendment History.

Section Number and Name	Description of Change	Brief Rationale
Title Page	Addition of EU CTR number. Removal of EudraCT number.	To comply with EU-CTR requirements.
1.1 Synopsis	Addition of EU CTR number.	To comply with EU-CTR requirements.
2.4.1.1. Risks Associated with Teclistamab and Talquetamab; 6.5.2. ICANS and Other Neurotoxicity; 6.5.2.2. Management Guidelines for [redacted]; 6.9.4.1. Teclistamab, Talquetamab and Daratumumab SC; 8.3.7. Neurologic Examinations	[redacted] disorder was added to the risk table.<br>Text added for clarification for management of other neurotoxicity.<br>[redacted].<br>Text added to provide guidance on talquetamab dose delays for other neurotoxicities.<br>[redacted].	These updates were made to address an urgent safety measure based on data from participants receiving talquetamab. The sponsor is updating the protocol to further characterize this risk [redacted]
6.1. Study Treatment Administered	For each study treatment, added if use is in accordance or not with Marketing Authorization in the EU/EEA.	To comply with EU-CTR requirements.
6.9.1.3. Skipping of Study Drugs	Recommendations for teclistamab dosing after delays in treatment (Table 48) revised	To align with teclistamab program level protocol language.
10.7. Appendix 7: Cockcroft-Gault Formula	Minor revision to footnote of the Cockcroft-Gault formula.	To align with teclistamab program level protocol language.

TABLE OF CONTENTS

PROTOCOL AMENDMENT SUMMARY OF CHANGES TABLE 2

TABLE OF CONTENTS 3

LIST OF IN-TEXT TABLES AND FIGURES 9

1. PROTOCOL SUMMARY 12
1.1. Synopsis 12
1.2. Schema 16
1.3. Schedule of Activities 20

2. INTRODUCTION 37
2.1. Study Rationale 37
2.1.1. Multiple Myeloma 37
2.1.2. Treatment Options for Patients with Newly Diagnosed Multiple Myeloma 38
2.1.3. Bispecific Antibodies to Treat B Cell Malignancies 40
2.1.4. Teclistamab 41
2.1.4.1. Summary of Clinical Studies 41
2.1.4.1.1. Study 64007957MMY1001 (MajesTEC-1) 42
2.1.4.1.2. Study 64407564MMY1002 (TriMM-2) 42
2.1.4.1.3. Study 64007957MMY1004 (MajesTEC-2) 43
2.1.4.1.4. Study 64007957MMY3003 (MajesTEC-4) 46
2.1.4.1.5. Study 64007957MMY3005 (MajesTEC-7) 46
2.1.5. Talquetamab 46
2.1.5.1. Summary of Clinical Studies 46
2.1.5.1.1. Study 64407564MMY1001 (MonumenTAL-1) 47
2.1.5.1.2. Study 64407564MMY1002 (TriMM-2) 48
2.1.5.1.3. Study 64407564MMY1004 (MonumenTAL-2) 48
2.1.5.2. Teclistamab/Talquetamab Combination Studies 51
2.1.5.2.1. Study 64007957MMY1003 (RedirecTT-1) 51
2.2. Rationale for Bispecific Antibody-based Combination Regimens 53
2.2.1. Rationale for Bispecific Antibody Therapy Combined with Daratumumab 53
2.2.2. Rationale for Bispecific Antibody Therapy Combined with Daratumumab and an IMiD 54
2.2.3. Rationale for Bispecific Antibody Therapy Combined with Daratumumab, an IMiD, and Bortezomib 55
2.2.4. Rationale for Combining Teclistamab and Talquetamab as Replacement for HDT and ASCT 56
2.3. Background 57
2.4. Benefit-Risk Assessment 57
2.4.1. Risks for Study Participation 57
2.4.1.1. Risks Associated with Teclistamab and Talquetamab 57
2.4.1.2. Risks Associated with Daratumumab SC 60
2.4.1.3. Risks Associated with Lenalidomide 60
2.4.1.4. Risks Associated with Bortezomib 60
2.4.1.5. Risks of Overlapping Toxicities 61
2.4.1.5.1. General 61
2.4.1.5.2. Risk of Overlapping Toxicities for CCI [redacted] 61
2.4.2. Benefits for Study Participation 61
2.4.2.1. Benefits for Teclistamab or Talquetamab with Daratumumab, Lenalidomide, and Dexamethasone, with or without Bortezomib, as Induction 62
2.4.2.2. Benefits for Teclistamab or Talquetamab with Daratumumab, with or without Lenalidomide, as Maintenance 62
2.4.2.3. Benefits for CCI [redacted] 62
2.4.3. Benefit-Risk Assessment for Study Participation 63

3. OBJECTIVES AND ENDPOINTS 65

4. STUDY DESIGN 67
4.1. Overall Design 67
4.1.1. Initiation and Expansion Rules for All Arms 70
4.1.2. Scientific Rationale for Study Design 71
4.1.3. Study-Specific Ethical Design Considerations 71
4.1.4. Rationale for Maintenance Treatment Duration 72
4.2. Justification for Dose 73
4.2.1. Treatment Dose and Schedule for Teclistamab 73
4.2.2. Treatment Dose and Schedule for Talquetamab 73
4.2.3. Treatment Dose and Schedule for [redacted] 74
4.2.4. Treatment Dose and Route of Administration for Other Study Drugs 75
4.3. End of Study Definition 75

5. STUDY POPULATION 75
5.1. Inclusion Criteria 76
5.2. Exclusion Criteria 79
5.3. Lifestyle Considerations 84
5.3.1. Daratumumab Interference With Indirect Antiglobulin Test Results 85
5.4. Screen Failures 85

6. STUDY TREATMENT AND CONCOMITANT THERAPY 86
6.1. Study Treatment Administered 86
6.1.1. Study Treatment Administered – Tec-DRd (Arms A, A1, or [redacted]) and Tec-DVRd (Arm B) Induction 91
6.1.3. Study Treatment Administered – [redacted] (Arms [redacted] and [redacted]) and [redacted] (Arms [redacted] and [redacted]) Induction 96
6.1.5. Study Treatment Administration – Arm [redacted] – [redacted] Following Induction 100
6.1.6. Study Treatment Administration – Arms A and C - Tec-DR Maintenance 102
6.1.7. Study Treatment Administration – Arm [redacted] – [redacted] Maintenance 105
6.1.8. Study Treatment Administration – Arms A, A1, B, C, C1, [redacted], and [redacted] – Tec-D Maintenance 108
6.1.9. Study Treatment Administration – Arms [redacted] and [redacted] – [redacted] Maintenance 110
6.2. Pretreatment Medications 112
6.2.1. Event-driven Pretreatment Medications 112
6.3. Posttreatment Medication for Daratumumab SC for Participants with Higher Risk of Respiratory Complications 112
6.4. Required Safety Monitoring 113
6.4.1. Safety Monitoring for Teclistamab and Talquetamab Dosing Through the First Three Doses 113
6.4.1.1. Guidance for Outpatient Administration of First Three Doses of Teclistamab or Talquetamab (only after consultation with the sponsor) 113
6.4.1.2. Required Hospitalization for Teclistamab or Talquetamab Dosing Following Specified Adverse Events 113
6.4.1.3. Hospitalization Discharge Criteria 114
6.4.2. Outpatient Monitoring After Daratumumab SC Administration 114
6.5. Management Guidelines for Potential Toxicities 114
6.5.1. CRS 114
6.5.1.1. Management Guidelines for CRS 115
6.5.2. ICANS and Other Neurotoxicity 116
6.5.2.1. Management Guidelines for ICANS 117
6.5.2.2. Management Guidelines for [redacted] 119
6.5.3. Hypogammaglobulinemia 119
6.5.4. Infection 119
6.5.4.1. Infection Prophylaxis 119
6.5.4.2. Vaccinations 120
6.5.4.3. Infection Management 120
6.5.4.4. COVID-19 Infection 121

6.5.4.5. HBV Reactivation 121
6.5.4.6. HCV Reactivation 121
6.5.5. sARRs 122
6.5.5.1. Management of sARRs Related to Daratumumab SC 123
6.5.6. Injection-site Reactions 124
6.5.6.1. Injection-site Reactions Related to Bortezomib 124
6.5.7. Tumor Lysis Syndrome 125
6.5.8. Immune-related AEs 125
6.5.9. Rash (Participants Receiving Talquetamab) 125
6.5.10. Nail Dysfunction (Participants Receiving Talquetamab) 125
6.5.11. Oral Toxicity (Participants Receiving Talquetamab) 126
6.6. Preparation/Handling/Storage/Accountability 126
6.6.1. Teclistamab and Talquetamab Preparation/Handling/Storage 126
6.6.2. Preparation/Handling/Storage for Other Combination Agents 126
6.6.3. Accountability 126
6.7. Measures to Minimize Bias: Randomization and Blinding 127
6.8. Study Treatment Compliance 128
6.9. Dosing Interruption and Dose Reduction 128
6.9.1. Guidance for Dosing Interruption and Skipping (All Study Drugs) 130
6.9.1.1. Cycle Delays 130
6.9.1.2. Dosing Interruption of a Study Drug During a Cycle 130
6.9.1.3. Skipping of Study Drugs 130
6.9.2. Guidance for Dose Reduction 135
6.9.3. Hematologic Adverse Events 136
6.9.4. Non-hematologic Adverse Events 138
6.9.4.1. Teclistamab, Talquetamab, and Daratumumab SC 138
6.9.4.2. Lenalidomide 139
6.9.4.3. Bortezomib 140
6.9.4.4. Dexamethasone 141
6.10. Continued Access to Study Treatment After the End of the Study 142
6.11. Treatment of Overdose 142
6.12. Concomitant Therapy 143
6.12.1. Permitted Therapies 143
6.12.2. Recommended Therapies 144
6.12.2.1. Infection Prophylaxis 144
6.12.2.2. Management of HBV Reactivation 144
6.12.2.3. Drugs Affecting Bone Structure and Mineralization 145
6.12.2.4. Prevention of Steroid-induced Gastritis 145
6.12.2.5. Prevention of Deep Vein Thrombosis and Pulmonary Embolism 145
6.12.2.6. Mitigation Measures in Case of Positive Testing for COVID-19 145
6.12.3. Prohibited or Restricted Therapies 146
6.12.3.1. Prohibited and Restricted Therapies Specific to Teclistamab and Talquetamab 147
6.12.3.2. Prohibited and Restricted Therapies Specific to Lenalidomide 147
6.12.3.3. Prohibited and Restricted Therapies Specific to Bortezomib (Arms B, [redacted], and [redacted]) 147
6.13. Subsequent Antimyeloma Therapy 148

7. DISCONTINUATION OF STUDY TREATMENT AND PARTICIPANT DISCONTINUATION/WITHDRAWAL 148
7.1. Discontinuation of Study Treatment 148
7.1.1. Discontinuation of All Study Treatment 148
7.1.2. Discontinuation of Teclistamab and Talquetamab 149
7.1.3. Discontinuation of Daratumumab SC 149
7.1.4. Discontinuation of Lenalidomide 150
7.1.5. Discontinuation of Bortezomib 150
7.1.6. Discontinuation of Dexamethasone 150
7.2. Participant Discontinuation/Withdrawal From the Study 150
7.2.1. Withdrawal From the Use of Study Samples 151

7.3. Lost to Follow-up 151

8. STUDY ASSESSMENTS AND PROCEDURES 152
8.1. Study Procedures 152
8.1.1. Overview 152
8.1.1.1. Screening Phase 152
8.1.1.2. Treatment Phase 153
8.1.1.2.1. Induction Treatment (Arms A, A1, B, [redacted], and [redacted] Only) 153
8.1.1.2.1.1. End of Induction Treatment 153
8.1.1.3. Stem Cell Collection, HDT, and ASCT (as per SoC) 153
8.1.1.3.1. Mobilization and Harvesting of Stem Cells 153
8.1.1.3.2. Conditioning (Melphalan) 154
8.1.1.3.3. Transplant 154
8.1.1.3.4. Engraftment/Recovery 154
8.1.1.4. [redacted] 154
8.1.1.5. Maintenance Treatment 154
8.1.1.5.1. EOT Visit 155
8.1.1.6. Follow-up Phase 155
8.1.1.6.1. SoC Maintenance 156
8.1.2. Local Laboratory Assessments 156
8.1.3. Sample Collection and Handling 156
8.1.4. Study-Specific Materials 156
8.1.5. Home Health Care and Telehealth Visits 157
8.2. Efficacy Assessments 157
8.2.1. M-protein Measurements in Serum and Urine 158
8.2.1.1. Potential for Daratumumab Interference with Disease Evaluations 158
8.2.2. Bone Marrow Examination 159
8.2.3. Minimal Residual Disease Evaluations 161
8.2.4. Imaging for Disease Evaluation 161
8.2.4.1. Imaging at Time of MRD Assessments 161
8.2.5. Documentation of Soft-Tissue Plasmacytomas 162
8.2.6. Patient-reported Outcomes 162
8.2.6.1. [redacted] 163
8.2.6.2. [redacted] 163
8.2.6.3. [redacted] 163
8.3. Safety Assessments 163
8.3.1. Physical Examinations 164
8.3.2. Vital Signs 164
8.3.3. Electrocardiograms 164
8.3.4. Clinical Safety Laboratory Assessments 164
8.3.5. Hepatitis Testing 164
8.3.5.1. Hepatitis B Virus Testing 164
8.3.5.2. Hepatitis C Virus Testing 165
8.3.5.3. Ongoing Hepatitis B Virus and Hepatitis C Virus Testing 165
8.3.6. Pregnancy Testing 166
8.3.7. Neurological Examination 166
8.3.8. ECOG Performance Status 166
8.4. Adverse Events, Serious Adverse Events, and Other Safety Reporting 166
8.4.1. Time Period and Frequency for Collecting Adverse Event and Serious Adverse Event Information 167
8.4.2. Method of Detecting Adverse Events and Serious Adverse Events 168
8.4.3. Follow-up of Adverse Events and Serious Adverse Events 168
8.4.4. Regulatory Reporting Requirements for Serious Adverse Events 168
8.4.5. Pregnancy 169
8.4.6. Disease-Related Events and Disease-Related Outcomes Not Qualifying as Adverse Events or Serious Adverse Events 169
8.4.7. Adverse Events of Special Interest 170

8.5. Pharmacokinetics and Immunogenicity ... 170
8.5.1. Evaluations ... 170
8.5.2. Analytical Procedures ... 170
8.5.3. Pharmacokinetic Parameters and Evaluations ... 171
8.5.4. Pharmacokinetic/Pharmacodynamic Evaluations ... 171
8.5.5. Immunogenicity Parameters and Evaluations ... 171
8.6. Genetics ... 171
8.7. Biomarkers ... 171
8.7.1. Pharmacodynamics and Exploratory Biomarker Studies ... 171
8.7.2. Minimal Residual Disease ... 172
8.7.3. Additional Collections ... 172

9. STATISTICAL CONSIDERATIONS ... 173
9.1. Statistical Hypotheses ... 173
9.2. Sample Size Determination ... 173
9.3. Populations for Analysis Sets ... 173
9.4. Statistical Analyses ... 174
9.4.1. General Considerations ... 174
9.4.2. Primary Endpoints ... 175
9.4.3. Secondary Endpoints ... 175
9.4.4. Safety Analyses ... 176
9.4.5. Other Analyses ... 177
9.4.5.1. Pharmacokinetic Analyses ... 177
9.4.5.2. Biomarkers Analyses ... 177
9.4.5.3. Immunogenicity Analyses ... 177
9.4.5.4. Pharmacokinetic/Pharmacodynamic Analyses ... 178
9.4.5.5. PRO Analyses ... 178
9.5. Interim Analyses ... 178

10. SUPPORTING DOCUMENTATION AND OPERATIONAL CONSIDERATIONS ... 179
10.1. Appendix 1: Abbreviations and Definitions ... 179
10.2. Appendix 2: Regulatory, Ethical, and Study Oversight Considerations ... 183
10.2.1. Regulatory and Ethical Considerations ... 183
10.2.2. Financial Disclosure ... 186
10.2.3. Informed Consent Process ... 186
10.2.4. Recruitment Strategy ... 187
10.2.5. Data Protection ... 187
10.2.6. Storage, Use, Transfer, and Retention of Data and Samples ... 188
10.2.7. Committees Structure ... 188
10.2.8. Use of Information and Publication ... 188
10.2.9. Data Quality Assurance ... 189
10.2.10. Case Report Form Completion ... 189
10.2.11. Source Documents ... 190
10.2.12. Monitoring ... 191
10.2.13. On-Site Audits ... 191
10.2.14. Record Retention ... 192
10.2.15. Study and Site Start and Closure ... 192
10.3. Appendix 3: Adverse Events, Serious Adverse Events, Product Quality Complaints, and Other Safety Reporting: Definitions and Procedures for Recording, Evaluating, Follow-up, and Reporting ... 194
10.3.1. Adverse Event Definitions and Classifications ... 194
10.3.2. Attribution Definitions ... 195
10.3.3. NCI-CTCAE Grading of Adverse Event Severity ... 196
10.3.4. Special Reporting Situations ... 197
10.3.5. Adverse Event Reporting Procedures ... 197
10.3.6. Product Quality Complaint Handling ... 199
10.3.7. Contacting Sponsor Regarding Safety, Including Product Quality ... 199

10.4. Appendix 4: IMWG Diagnostic Criteria ... 200
10.5. Appendix 5: IMWG Response Criteria ... 201
10.6. Appendix 6: ECOG Performance Status ... 203
10.7. Appendix 7: Cockcroft-Gault Formula ... 204
10.8. Appendix 8: Serum Calcium Corrected for Albumin ... 205
10.9. Appendix 9: Contraceptive Guidance and Lenalidomide Global/Local PPP Programs ... 206
10.10. Appendix 10: Conversion Table for Steroids ... 209
10.11. Appendix 11: Asthma Guidelines (Adults) ... 210
10.12. Appendix 12: Body Surface Area Calculation ... 212
10.13. Appendix 13: Considerations for Outpatient Teclistamab CCI [redacted] Administration Through the First Treatment Dose ... 213
10.14. Appendix 14: Severity Grading for CRS (ASTCT) ... 215
10.15. Appendix 15: ICE Tool ... 216
10.16. Appendix 16: Severity Grading for ICANS (ASTCT) ... 217
10.17. Appendix 17: Prophylaxis of Venous Thromboembolism ... 218
10.18. Appendix 18: Clinical Laboratory Tests ... 219
10.19. Appendix 19: Study Conduct During COVID-19 Pandemic ... 220
10.20. Appendix 20: CYP3A4 Inducers ... 223
10.21. Appendix 21: New York Heart Association Functional Classification ... 224
10.22. Appendix 22: Previous Schedule of Activities and Dose Regimens for Arm A and Arm C (Prior to Protocol Amendment 3) ... 225
10.22.1. Schedule of Activities ... 226
10.22.2. Justification for Treatment Dose ... 234
10.22.2.1. Treatment Dose and Schedule for Teclistamab ... 234
10.22.3. Study Treatment Administered ... 237
10.22.4. Skipping of Study Drugs ... 241
10.23. Appendix 23: Protocol Amendment History ... 242

11. REFERENCES ... 259

INVESTIGATOR AGREEMENT ... 265

LIST OF IN-TEXT TABLES AND FIGURES

TABLES

Table 1: Guide to Schedules of Activities and Dosing 21
Table 2: Schedule of Activities for Screening – All Participants 22
Table 3: Arms A*, A1, [redacted] (Tec-DRd Induction), [redacted] Induction) – SoA for Treatment Phase, EOT Visit, and Posttreatment Follow-up Phase 23
Table 4: Arms B (Tec-DVRd Induction), [redacted] and [redacted] ([redacted] Induction) – SoA for Treatment Phase, EOT Visit, and Posttreatment Follow-up Phase 26
Table 5: Arm [redacted] ([redacted] Following Induction) – SoA for Treatment Phase, EOT Visit, and Posttreatment Follow-up Phase 29
Table 6: Arms A and C* (Tec-DR or Tec-D Maintenance), Arms A1, B, C1, [redacted] (Tec-D Maintenance), Arm [redacted] Maintenance), and Arms [redacted] and [redacted] Maintenance) – SoA for Treatment Phase, EOT Visit, and Posttreatment Follow-up Phase 31
Table 7: Arms A*, A1, and [redacted] (Tec-DRd Induction), Arm B (Tec-DVRd Induction), Arms [redacted] and [redacted] Induction), and Arms [redacted] and [redacted] Induction) – Schedule of PK, Immunogenicity, and Biomarker Sample Collection 34
Table 8: Arm [redacted] Following Induction) – Schedule of PK, Immunogenicity, and Biomarker Sample Collection 35
Table 9: Arms A and C* (Tec-DR or Tec-D Maintenance), Arms A1, B, C1, [redacted] and [redacted], (Tec-D Maintenance), Arms [redacted] and [redacted] ([redacted] Maintenance), and Arm [redacted] Maintenance) – Schedule of PK, Immunogenicity, and Biomarker Sample Collection 36
Table 10: Overview of Relevant Study Treatment Regimens in MajesTEC-2 43
Table 11: Overview of Relevant Study Treatment Regimens in MonumenTAL-2 49
Table 12: Overview of Tec-Tal Dose Combinations in RedirecTT-1 52
Table 13: Mitigation Strategies for Potential Risks Associated with Teclistamab and Talquetamab 58
Table 14: Study Treatment Administered – Overview – Arms A*, A1, and [redacted] (Induction) 87
Table 15: Study Treatment Administered – Overview – Arm B (Induction) 88
Table 16: Study Treatment Administered – Overview – Arm [redacted] Treatment Following Induction) 88
Table 17: Study Treatment Administered – Overview – Arms [redacted] and [redacted] (Induction) 88
Table 18: Study Treatment Administered – Overview – Arms [redacted] and [redacted] (Induction) 89
Table 19: Study Treatment Administered – Overview – Arms A and C* (Maintenance) 89
Table 20: Study Treatment Administered – Overview – Arms A, A1, B, C, C1, [redacted], and [redacted] (Maintenance) 89
Table 21: Study Treatment Administered – Overview – Arm [redacted] (Maintenance) 90
Table 22: Study Treatment Administered – Overview – Arms [redacted] and [redacted] (Maintenance) 90
Table 23: Dose Schedule and Pretreatment Medications for Arms A*, A1, or [redacted] (Tec-DRd), and Arm B (Tec-DVRd) Induction 91
Table 24: Study Treatment Administration Instruction – Arms A, A1, or [redacted] (Tec-DRd), and Arm B (Tec-DVRd) Induction 93
Table 25: Dose Schedule and Pretreatment Medications for Arms [redacted] and [redacted] and Arms [redacted] and [redacted]) Induction 96
Table 26: Study Treatment Administration Instruction – Arms [redacted] and [redacted] ([redacted]), and Arms F and [redacted]) Induction 97
Table 27: Dose Schedule and Pretreatment Medications for Arm [redacted] 100
Table 28: Study Treatment Administration Instructions – Arm [redacted] 101
Table 29: Dose Schedule and Pretreatment Medications for Arms A and C* - Tec-DR Maintenance 102
Table 30: Study Treatment Administration Instructions – Arms A and C - Tec-DR Maintenance 103
Table 31: Dose Schedule and Pretreatment Medications for Arm [redacted] Maintenance 105
Table 32 Study Treatment Administration Instructions for Arm [redacted] Maintenance 106
Table 33: Dose Schedule and Pretreatment Medications for Arms A, A1, B, C, C1, [redacted] and [redacted] – Tec-D Maintenance 108

Table 34: Study Treatment Administration Instructions for Arms A, A1, B, C, C1, [redacted], and [redacted] – Tec-D Maintenance ... 109
Table 35: Dose Schedule and Pretreatment Medications for Arms [redacted] and [redacted] – [redacted] Maintenance ... 110
Table 36: Study Treatment Administration Instructions for Arms [redacted] and [redacted] – [redacted] Maintenance ... 111
Table 37: Adverse Events Requiring Hospitalization for Subsequent Administration of Teclistamab or Talquetamab ... 114
Table 38: Recommended Management of CRS ... 116
Table 39: Recommended Management of ICANS ... 117
Table 40: Guidelines for the Management of Raised ICP/Cerebral Edema ... 118
Table 41: Guidelines for the Management of sARRs ... 123
Table 42: Guide to Dosing Interruption and Dose Reduction of Study Treatment for Induction Regimens ... 129
Table 43: Guide to Dosing Interruption and Dose Reduction of Study Treatment for Arm [redacted] for the [redacted] Regimen ... 129
Table 44: Guide to Dosing Interruption and Dose Reduction of Study Treatment for Maintenance Regimens ... 129
Table 45: Teclistamab, Talquetamab, and Daratumumab SC Dose Skips during Tec-DRd, Tec-DVRd, [redacted], or [redacted] Induction ... 131
Table 46: Teclistamab and Talquetamab Dose Skips During [redacted] Treatment ... 131
Table 47: Teclistamab, Talquetamab, and Daratumumab SC Dose Skips During Tec-DR, Tec-D, [redacted], or [redacted] Maintenance* ... 132
Table 48 Recommendations For Teclistamab Dosing After Delays in Treatment ... 133
Table 49: Recommendations for [redacted] Dosing After Delays in Treatment ... 134
Table 50: Dose Reduction Guidance for Lenalidomide Adverse Events for Arms A, A1, and [redacted] (Tec-DRd), Arm B (Tec-DVRd), Arms [redacted] and [redacted] and Arms [redacted] and [redacted] Induction Treatment ... 135
Table 51: Dose Reduction Guidance for Lenalidomide Adverse Events for Arms A and C (Tec-DR), and Arm [redacted] Maintenance Treatment ... 135
Table 52: Dose Reduction Guidance for Bortezomib Adverse Events for Arm B (Tec-DVRd) and Arms [redacted] and [redacted]) Induction Treatment ... 135
Table 53: Dose Reduction Guidance for Dexamethasone Adverse Events for Arms A, A1, and D (Tec-DRd), Arm B (Tec-DVRd), Arms [redacted] and [redacted] and Arms [redacted] and [redacted]) Induction Treatment ... 136
Table 54: Study Drug Dosing Interruption or Dose Reduction (Lenalidomide or Bortezomib Only) Due to Hematologic Adverse Events ... 137
Table 55: Teclistamab, Talquetamab, and Daratumumab SC Dosing Interruption for Non-hematologic Adverse Events ... 138
Table 56: Lenalidomide Dose Modifications Due to Renal Impairment ... 139
Table 57: Lenalidomide Dosing Interruption or Dose Reduction for Non-hematologic Adverse Events ... 139
Table 58: Treatment Guidelines for Bortezomib-related Neuropathy ... 140
Table 59: Treatment Guidelines for Dexamethasone-related Adverse Events ... 141
Table 60: Bone Marrow Testing – All Arms ... 160
Table 61: Eligibility Related to Hepatitis B Test Results ... 165
Table 62: Length of Widest 95% Confidence Interval Around AE Rates ... 173
Table 63: Guide to Previous Schedule of Activities and Dosing for Arm A and Arm C ... 225
Table 64: Schedule of Activities for Treatment Phase, EOT Visit, and Posttreatment Follow-up Phase – Arm A - Tec-DRd Induction ... 226
Table 65: Schedule of Activities for Treatment Phase, EOT Visit, and Posttreatment Follow-up Phase – Arm A, Arm B, and Arm C - Tec-DR Maintenance ... 229
Table 66: Schedule of PK, Immunogenicity, and Biomarker Sample Collection for Arm A Tec-DRd Induction ... 232
Table 67: Schedule of PK, Immunogenicity, and Biomarker Sample Collection for Arm C - Tec-DR Maintenance ... 233
Table 68: Model-predicted Median (5th-95th Percentiles) Steady-state For the Two Dose Schedules ... 236

Table 69: Study Treatment Administered – Overview – Arm A 237
Table 70: Study Treatment Administered – Overview – Arm C 238
Table 71: Dose Schedule and Pretreatment Medications for Arm A Tec-DRd Induction 239
Table 72: Dose Schedule and Pretreatment Medications for Arm C Tec-DR Maintenance 240
Table 73: Teclistamab and Daratumumab SC Dose Skips During Arm A Tec-DRd and Arm B Tec-DVRd Induction 241
Table 74: Teclistamab and Daratumumab SC Dose Skips During Arm A, Arm B, and Arm C Tec-DR Maintenance 241

FIGURES

Figure 1: Schematic Overview of the Study Phases 16
Figure 2: Schematic Overview of Safety Data Snapshot (Arms A1 and B) 18
Figure 3: Schematic of Dose Schedule – Induction (Tec-DRd) – Arm A*, Arm A1, and Arm [redacted] 18
Figure 4: Schematic of Dose Schedule – Induction (Tec-DVRd) – Arm B 18
Figure 5: Schematic of Dose Schedule – Induction ([redacted]) – Arm [redacted] and Arm [redacted] 18
Figure 6: Schematic of Dose Schedule – Induction ([redacted]) – Arm [redacted] and Arm [redacted] 19
Figure 7: Schematic of Dose Schedule – [redacted] Following Induction – Arm [redacted]* 19
Figure 8: Schematic of Dose Schedule – Maintenance (Tec-DR*) – Arm A and Arm C** 19
Figure 9: Schematic of Dose Schedule – Maintenance (Tec-D*) – Arm A**, Arm A1, Arm B, Arm C**, Arm C1, Arm [redacted], and Arm [redacted] 19
Figure 10: Schematic of Dose Schedule – Maintenance ([redacted]) – Arm [redacted] 20
Figure 11: Schematic of Dose Schedule – Maintenance ([redacted]) – Arm [redacted] and Arm [redacted] 20
Figure 12: Schematic Overview of the Mechanisms of Action of Teclistamab and Talquetamab 41
Figure 13: Predicted Teclistamab Concentration-time Profiles Following Teclistamab SC Dose of 1.5 mg/kg Weekly (C1-C2) Followed by 3 mg/kg Q2W in Cycle 3+ Versus Teclistamab SC Dose of 1.5 mg/kg Weekly 235
Figure 14: Predicted Teclistamab Steady-state PK Parameters for 3 mg/kg Q2W SC Dosing (Cycle 3+) Versus 1.5 mg/kg Weekly SC Dosing 236
Figure 15: Schematic of Dose Schedule – Arm A 237
Figure 16: Schematic of Dose Schedule – Arm C 238

1. PROTOCOL SUMMARY

1.1. Synopsis

A Phase 2 Study to Evaluate Safety and Efficacy of Teclistamab- and Talquetamab-based Combination Regimens in Participants with Newly Diagnosed Transplant Eligible Multiple Myeloma

EU TRIAL NUMBER: 2024-517382-17-00

BENEFIT-RISK ASSESSMENT

Teclistamab and talquetamab have been studied as monotherapies and in combinations with standard of care agents in treating multiple myeloma and have demonstrated deep and durable responses. The addition of teclistamab or talquetamab to daratumumab, DR, DRd, or DVRd is expected to combine the individual cytotoxic and immunomodulatory aspects of each drug and lead to enhanced efficacy against multiple myeloma. Additionally, the combination of teclistamab and talquetamab has demonstrated high rates of deep and robust responses in heavily pretreated patients with relapsed/refractory multiple myeloma, and as such is hypothesized to be an effective alternative to HDT+ASCT in patients with ND-TEMM.

Safety risks associated with teclistamab and talquetamab include CRS, neurotoxicity including ICANS, hypogammaglobulinemia, cytopenias, serious infections, and injection-site reactions. Additionally, skin and nail, and oral toxicity are safety risks for talquetamab. The primary acute toxicity for SC daratumumab is sARRs. The most commonly reported adverse reactions associated with IMiDs (eg, lenalidomide) in pivotal studies have been hematologic toxicities. With combination treatment, there is a potential risk for overlapping toxicities (eg, cytopenias, serious infections, and sARRs; and for [redacted] CRS, ICANS, infection, and hypogammaglobulinemia). The current study design accounts for potential risks by administration of step-up doses of teclistamab or talquetamab, staggered initiation of daratumumab SC and teclistamab or talquetamab therapy, initiation of lenalidomide administration on Cycle 2 Day 1, and the use of pretreatment medications. The planned mitigation measures aim to reduce potential risks in conjunction with robust dose modification guidelines and management strategies for hematologic and non-hematologic AEs, including extensive protocol guidance on monitoring, prophylaxis, and management of infections.

OBJECTIVES AND ENDPOINTS

The primary objective is to evaluate the safety and tolerability of teclistamab- and talquetamab-based combination regimens over the entire treatment phase for each arm, in participants with ND-TEMM.

Hypothesis

The clinical hypothesis of this study is that teclistamab- and talquetamab-based combination regimens are safe and well tolerated treatments in participants with ND-TEMM.

OVERALL DESIGN

Treatment Phase:

Arms A, A1, B, [redacted] and [redacted]

After Screening, the Treatment Phase begins with the administration of study treatment: induction with Tec-DRd (Arms A, A1, and [redacted]), Tec-DVRd (Arm B), or [redacted] (Arms [redacted] and [redacted]). Based on emerging data from Arms A1, B, [redacted] and [redacted], the sponsor may also open Arms [redacted] and [redacted] where participants receive [redacted] induction. The Treatment Phase continues until the completion of the EOT Visit. Periodic safety evaluations will be conducted to ensure that treatment is safe and tolerable.

Participants will receive six 28-day cycles of Tec-DRd, Tec-DVRd, [redacted] or [redacted] induction therapy, followed by HDT and a single ASCT according to local SoC (Arms A, A1, B[redacted] and [redacted]) or [redacted] treatment for a maximum of eighteen 28-day cycles or until confirmed progressive disease, death, intolerable toxicity, loss to follow-up, or consent withdrawal, whichever comes first (Arm [redacted]. Stem cell collection is recommended to be done after Induction Cycle 3 according to local SoC.

After ASCT, per Amendment 4, participants in Arm A initially assigned to receive Tec-DR maintenance may receive Tec-D maintenance per investigator's choice (participants who have started Tec-DR may discontinue lenalidomide to receive Tec-D per investigator's choice). Participants in Arms A1, B, [redacted], and (if opened) [redacted] will receive study maintenance treatment with Tec-D, and participants in Arms [redacted] and (if opened) [redacted] will receive study maintenance treatment with [redacted] Study maintenance treatment will be for a maximum of 18 cycles or until confirmed progressive disease, death, intolerable toxicity, loss to follow-up, or consent withdrawal, whichever comes first. Tec-DR, Tec-D, or [redacted] study maintenance treatment or [redacted] study treatment can be discontinued when 12 months of sustained MRD negativity has been observed during the study.

Arms C, C1, and [redacted]

Participants enter the Screening Phase after induction, HDT, and ASCT according to local SoC (outside of the study). The Treatment Phase begins with the administration of study maintenance treatment (per Amendment 4, participants in Arm C initially assigned to receive Tec-DR may receive Tec-D per investigator's choice, and participants in Arm [redacted] will receive [redacted]) and continues until the completion of the EOT Visit. Based on evolving data, the sponsor may also open Arm C1 where participants receive Tec-D maintenance treatment with teclistamab administered monthly as of Cycle 1. Participants will receive study maintenance treatment with Tec-DR, Tec-D, or [redacted] for a maximum of 18 cycles or until confirmed progressive disease, death, intolerable toxicity, loss to follow-up, or consent withdrawal, whichever comes first. Tec-DR, Tec-D, or [redacted] study maintenance therapy can be discontinued when 12 months of sustained MRD negativity has been observed during the study. Periodic safety evaluations will be conducted to ensure that treatment is safe and tolerable.

Follow-up Phase:

All Arms

Upon treatment discontinuation, an EOT Visit will be conducted. Thereafter, participants will continue in the Follow-up Phase until death, withdrawal of consent, loss to follow-up, or end of the study, whichever occurs first.

Following study maintenance therapy, additional SoC maintenance treatment per institutional standard and local investigator decision, is permitted. This additional maintenance therapy, without intercurrent progressive disease, is not considered subsequent therapy and is outside of the study.

In the Follow-up Phase, participants who discontinue treatment before disease progression must continue to have disease evaluations and should not initiate any subsequent antimyeloma treatment until confirmed disease progression. After disease progression is documented, follow-up will continue and subsequent antimyeloma treatment, disease progression data (per investigator assessment) on the first line of subsequent therapy, second primary malignancies, and survival status will also be recorded.

NUMBER OF PARTICIPANTS

This study anticipates enrolling up to a maximum of approximately 320 participants. Arm A will enroll approximately 10 participants. Arms A1 and B will initially enroll approximately 20 participants each and may be expanded up to a total of 80 participants (Arms A1 and B combined). Arm C will enroll approximately 10 participants and based on evolving data, Arm C1 may also be opened to enroll approximately 10 participants. Arm [redacted] will initially enroll approximately 10 participants and may enroll up to a total of 30 participants. Arm [redacted] will initially enroll approximately 20 participants and may enroll up to 60 participants. Arms [redacted] and [redacted] will initially enroll approximately 10 participants each and may enroll up to 30 participants each. Based on emerging data from Arms A1, B, [redacted], and [redacted], Arms [redacted] and [redacted] may also be opened to enroll up to 30 participants each.

DESCRIPTION OF STUDY TREATMENT

Study treatment will be administered in 28-day cycles.

Induction Treatment – 6 cycles:

Arms A, A1, and [redacted] (Tec-DRd) or Arm B (Tec-DVRd)

- Teclistamab SC
  - Cycle 1: 2 step-up doses (0.06 and 0.3 mg/kg) on Days 2 and 4 followed by treatment doses of 1.5 mg/kg on Days 8 and 15
  - Cycles 2-6: treatment dose of 3 mg/kg Q4W on Day 1 of each cycle
- Daratumumab SC 1800 mg – Cycles 1-2: weekly on Days 1, 8, 15, and 22, and Cycles 3 to 6: Q2W on Days 1 and 15
- Lenalidomide oral 25 mg – Cycles 2 to 6: Days 1-21
- Dexamethasone oral/IV 20 mg – Cycles 1-2: Days 1-2, 8-9, 15-16, and 22-23
- ***For Arm B only*****:** Bortezomib SC 1.3 mg/m^2 – Cycles 1-6: weekly on Days 1, 8, 15 and 22 (in addition to the above)

[redacted]

- Daratumumab, lenalidomide, and dexamethasone (same as for induction in Arms A, A1, B, and [redacted])
- ***For Arms F and F1 only*****:** Bortezomib (same as for Arm B)

[redacted]

Maintenance Treatment – maximum of 18 cycles:

Arms A and C* (Tec-DR or Tec-D), Arms A1, B, [redacted] and if opened, Arms C1 and [redacted] (Tec-D)

- Teclistamab SC
  - Cycle 1: 2 step-up doses (0.06 and 0.3 mg/kg) on Days 2 and 4 followed by 1.5 mg/kg treatment doses on Days 8 and 15
  - Cycles 2-18: treatment dose of 3 mg/kg Q4W on Day 1 of each cycle
- Daratumumab SC 1800 mg – Cycles 1 to 18: Q4W on Day 1 of each cycle

- ***For Arms A and C only***: Lenalidomide oral 10 mg – Cycles 2 to 18: daily

* Per Amendment 4, participants in Arms A or C initially assigned to receive Tec-DR maintenance may receive Tec-D maintenance per investigator's choice.

[redacted]

- [redacted]aratumumab (same as for maintenance in Arms A, A1, B, C, and [redacted]

Arm [redacted] ([redacted])

- [redacted] (same as for maintenance in Arm [redacted])
- Daratumumab, lenalidomide (same as for maintenance in Arms A and C)

EFFICACY EVALUATIONS

Efficacy assessments will occur per IMWG criteria (2016), using data from serum, urine, bone marrow, and imaging.

PHARMACOKINETIC AND IMMUNOGENICITY EVALUATIONS

Sparse blood samples will be collected for the measurement of serum concentrations of teclistamab, talquetamab, and daratumumab for PK analyses. Population PK analyses will be performed, and the results will be reported separately. The detection and characterization of ADAs to teclistamab, talquetamab, and daratumumab will be performed using validated assay methods.

PHARMACODYNAMIC AND EXPLORATORY BIOMARKER EVALUATIONS

Peripheral blood and bone marrow aspirate will be collected from participants in all arms. Additionally, the available apheresis product may be assessed for the presence of clonal plasma cells in the autograft. Biomarker assessments aim to: 1) evaluate pharmacodynamic biomarkers indicative of antimyeloma and immune activity of study treatment; 2) determine ability of study treatment to induce and sustain MRD negativity; 3) investigate biomarkers of response/resistance to study treatment; 4) explore prognostic and disease markers in relation to efficacy parameters; 5) determine the proportion of participants with clonal plasma cell-negative autograft after 3 cycles of study induction treatment.

PRO EVALUATIONS

[redacted], participants' symptoms, functioning, and general well-being will be captured using the [redacted] and [redacted] PRO instruments. The [redacted] will capture data on participants' oral health to further quantify [redacted] associated with [redacted] (Arms [redacted], [redacted]

SAFETY EVALUATIONS

Safety will be assessed by physical examinations, neurologic examinations, ECOG performance status, clinical laboratory tests, vital signs, and AE monitoring. The severity of AEs will be assessed using NCI-CTCAE Version 5.0, except for grading of CRS and ICANS, which will be assessed based on ASTCT guidelines. Concomitant medication use will be recorded.

STATISTICAL METHODS

The primary endpoints are AE and SAE rate and severity during treatment with teclistamab- and talquetamab-based combination regimens for each arm. No formal statistical hypothesis testing will be conducted. Descriptive statistics will be provided. In addition to safety, ability to mobilize and collect stem cells and efficacy data will be summarized using descriptive statistics.

1.2. Schema

Figure 1: Schematic Overview of the Study Phases

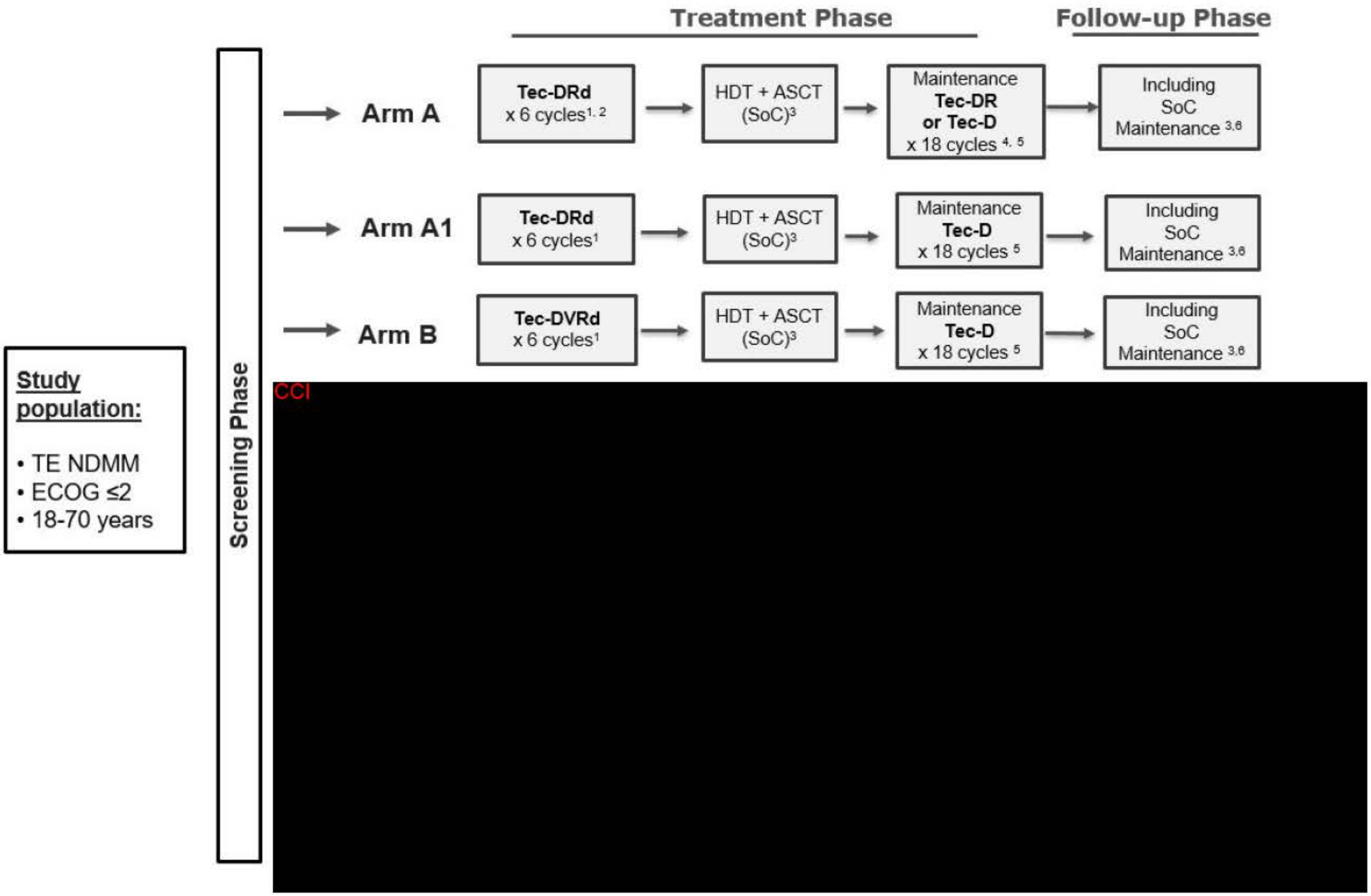

Figure 1: Schematic Overview of the Study Phases (Continued)

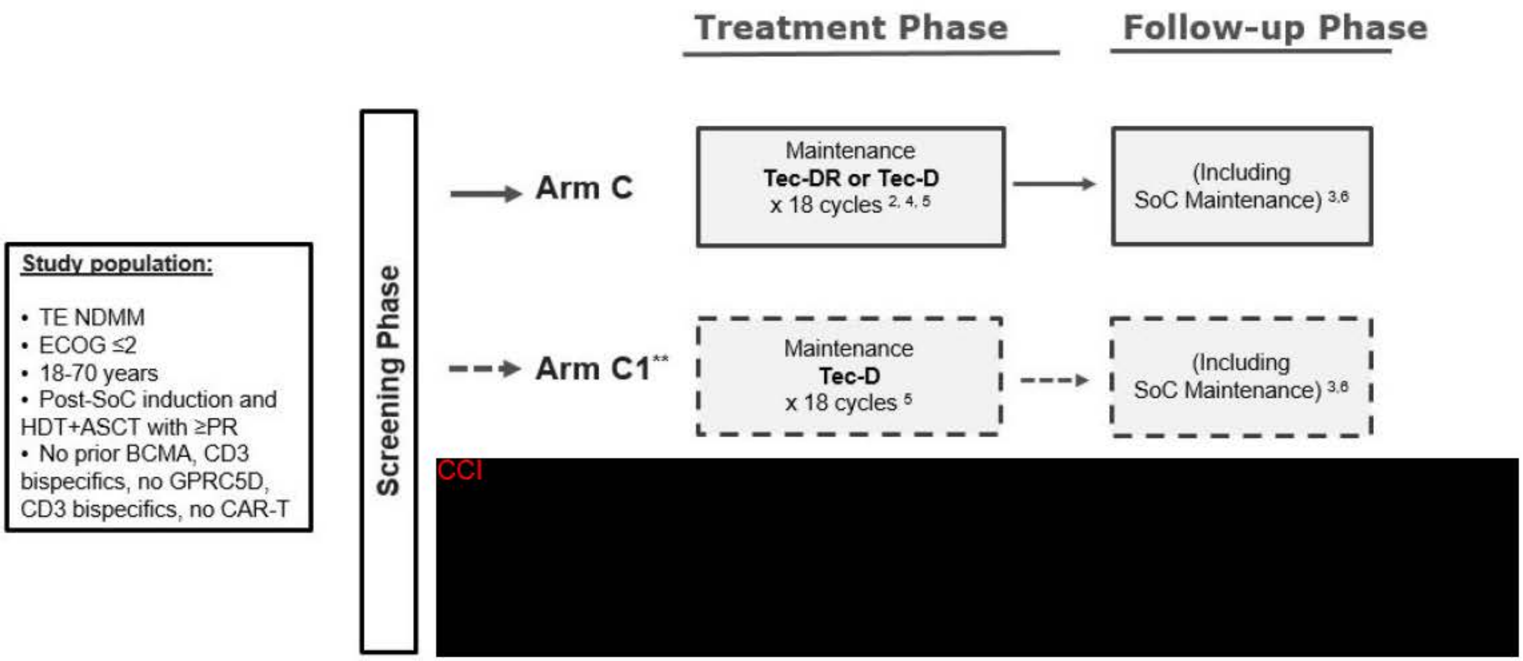

1. Including stem cell collection as described in Section 8.1.1.3.
2. Arm A and Arm C participants initially treated with previous teclistamab dosing schedules have transitioned to teclistamab monthly dosing. See Section 4.1.1 and Section 6.1 for details.
3. SoC treatments are not considered study treatments.
4. Per Amendment 4, participants in Arm A and Arm C initially assigned to receive Tec-DR maintenance may receive Tec-D maintenance per investigator's choice (participants who have started Tec-DR may discontinue lenalidomide to receive Tec-D per investigator's choice).
5. [redacted] therapy following induction, or study maintenance therapy with Tec-DR, Tec-D, [redacted] to be administered for a maximum of [redacted] and can be discontinued when 12 months of sustained MRD negativity has been observed during the study (See Section 4.1).
6. Disease evaluation before progressive disease every 12 weeks (±1 week) until confirmed PD or start of SST (whichever occurs earlier).

* Based on emerging data from Arms A1, B[redacted] and [redacted] the sponsor may open Arms [redacted]

** Based on evolving data, the sponsor may also open Arm C1 where participants receive Tec-DR maintenance treatment with teclistamab administered monthly as of Cycle 1.

Figure 2: Schematic Overview of Safety Data Snapshot (Arms A1 and B)

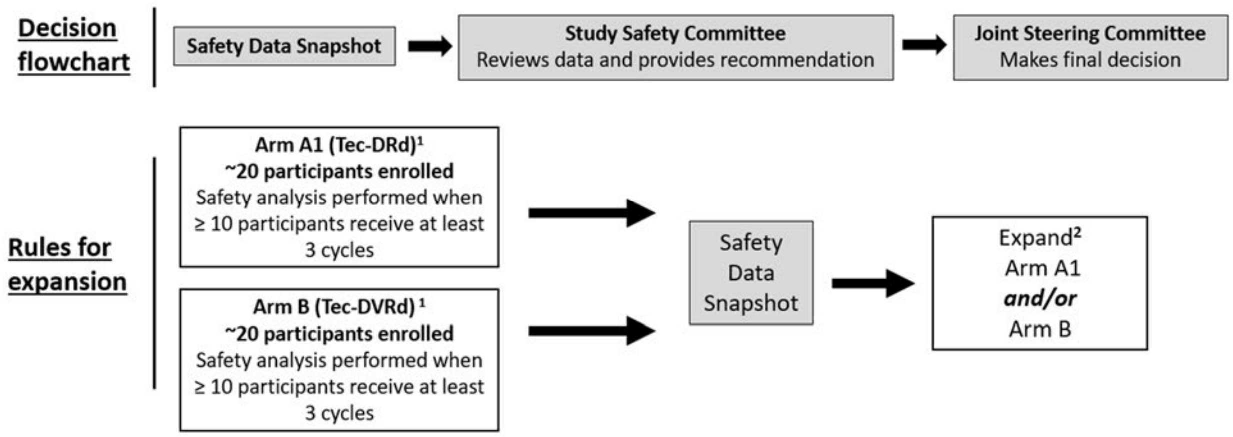

1. Arm A1 and Arm B will initially enroll approximately 20 participants each.
2. Based on the safety snapshot and the totality of data, Arm A1 *and/or* Arm B may be expanded up to a total of 80 participants (Arm A1 and B combined).

Figure 3: Schematic of Dose Schedule – Induction (Tec-DRd) – Arm A*, Arm A1, and Arm [redacted]

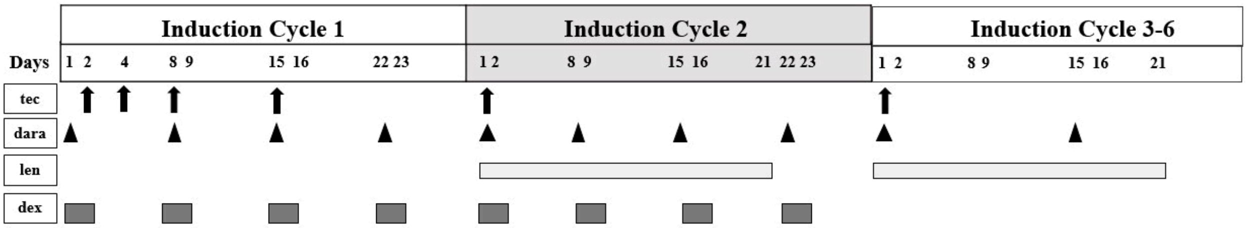

*Refer to Appendix 22 (Figure 15) for schematic dose schedule of Arm A in previous teclistamab weekly dosing schedule.

Figure 4: Schematic of Dose Schedule – Induction (Tec-DVRd) – Arm B

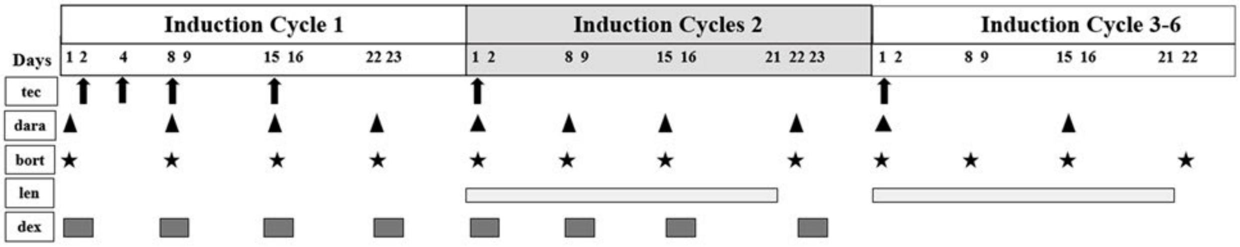

Figure 5: Schematic of Dose Schedule – Induction ([redacted]) – Arm [redacted] and Arm [redacted]

[redacted]

Figure 6: Schematic of Dose Schedule – Induction ([redacted]) – Arm [redacted] and Arm [redacted]

[redacted]

Figure 7: Schematic of Dose Schedule – [redacted] Following Induction – Arm [redacted]*

[redacted]

Figure 8: Schematic of Dose Schedule – Maintenance (Tec-DR*) – Arm A and Arm C**

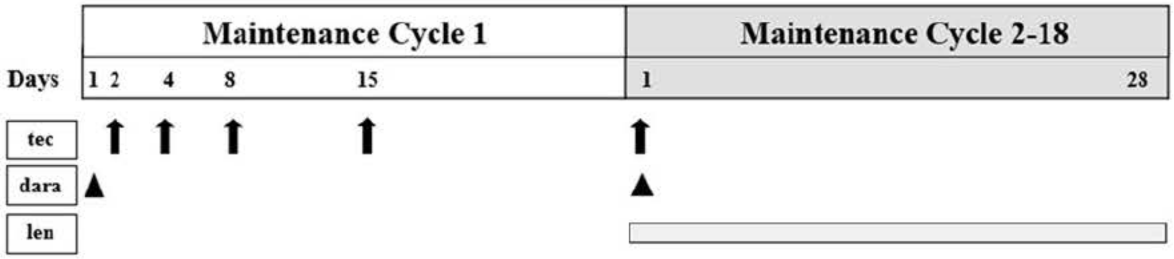

*Per Amendment 4, participants in Arms A or C initially assigned to receive Tec-DR maintenance may receive Tec-D maintenance per investigator's choice (participants who have started Tec-DR may discontinue lenalidomide to receive Tec-D per investigator's choice).
**Refer to Appendix 22 (Figure 15 and Figure 16) for schematic dose schedule of Arms A and C in previous biweekly dosing schedule.

Figure 9: Schematic of Dose Schedule – Maintenance (Tec-D*) – Arm A, Arm A1, Arm B, Arm C**, Arm C1, Arm [redacted], and Arm [redacted]**

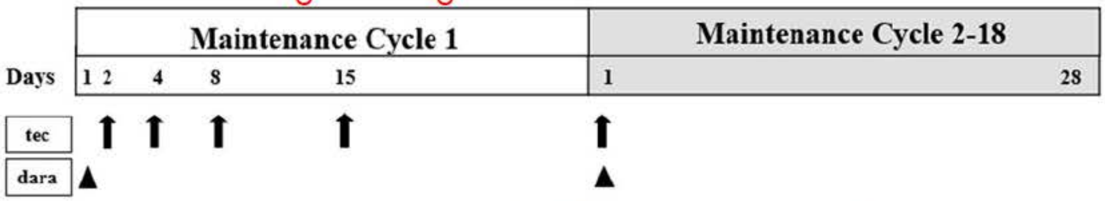

* Per Amendment 4, participants in Arms A or C initially assigned to receive Tec-DR maintenance may receive Tec-D maintenance per investigator's choice (participants who have started Tec-DR may discontinue lenalidomide to receive Tec-D per investigator's choice).
**Refer to Appendix 22 (Figure 15 and Figure 16) for schematic dose schedule of Arms A and C in previous biweekly dosing schedule.

Figure 10: Schematic of Dose Schedule – Maintenance [redacted] – Arm [redacted]

[redacted]

Figure 11: Schematic of Dose Schedule – Maintenance [redacted] – Arm [redacted] and Arm [redacted]

[redacted]

1.3. Schedule of Activities

Table 1 indicates which Schedules of Activities apply to each arm included in the current study. Dose schedules for study drugs and pretreatment medications are presented in Section 6.1 (applicable tables noted in Table 1). Except as noted, all assessments will occur predose on the dosing day. Evaluations for eligibility determination performed outside the screening window may need to be repeated unless specified otherwise.

Refer to Appendix 22 for Schedule of Activities (Table 64 and Table 65), Schedule of PK, Immunogenicity, and Biomarker Sample Collection (Table 66 and Table 67), dose schedule and pretreatment medications (Table 71 and Table 72) applicable to Arms A and C, respectively, for teclistamab weekly dosing schedule.

Table 1: Guide to Schedules of Activities and Dosing

	Arm A	Arm A1	Arm B	Arm C	Arm C1	Arm [redacted]	Arm [redacted]	Arm [redacted]	Arm [redacted]	Arm [redacted]	Arm [redacted]
Screening	Table 2										
Schedule of Activities	Induction: Table 3 Maint.: Table 6	Induction: Table 3 Maint.: Table 6	Induction: Table 4 Maint.: Table 6	Maint.: Table 6	Maint.: Table 6	Maint.: Table 6	Induction: Table 3 [redacted] Following Induction.: Table 5	Induction: Table 3 Maint.: Table 6	Induction: Table 3 Maint.: Table 6	Induction: Table 4 Maint.: Table 6	Induction: Table 4 Maint.: Table 6
PK, immunogenicity and biomarker sampling	Induction: Table 7 Maint.: Table 9	Induction: Table 7 Maint.: Table 9	Induction: Table 7 Maint.: Table 9	Maint.: Table 9	Maint.: Table 9	Maint.: Table 9	Induction: Table 7 [redacted] Following Induction.: Table 8	Induction: Table 7 Maint.: Table 9	Induction: Table 7 Maint.: Table 9	Induction: Table 7 Maint.: Table 9	Induction: Table 7 Maint.: Table 9
Dose schedule and pretreatment medications	Induction: Table 23 Maint.: Table 29 or Table 33	Induction: Table 23 Maint.: Table 33	Induction: Table 23 Maint.: Table 33	Maint.: Table 29 or Table 33	Maint.: Table 33	Maint.: Table 31	Induction: Table 23 [redacted] Following Induction.: Table 27	Induction: Table 25 Maint.: Table 33	Induction: Table 25 Maint.: Table 35	Induction: Table 25 Maint.: Table 33	Induction: Table 25 Maint.: Table 35
Bone marrow testing	Table 60										

Maint. = maintenance

Table 2: Schedule of Activities for Screening – All Participants

Assessments (within ≤28 d before enrollment)	Notes
**STUDY PROCEDURES**	
Informed consent	
Eligibility criteria	
Demography, medical history and prior multiple myeloma therapies	
Disease characteristics	See Section 8.1.1.1
Physical examination	Complete examination (including height, weight, ECOG performance status, and neurological examination, including ICE tool)
Vital signs including oxygen saturation	
12-lead ECG	
Chest X-ray or low-dose chest CT scan	If a whole body CT was obtained within 42 days before study inclusion, chest x-ray or low-dose chest CT scan is not needed.
Spirometry test	For participants with known or suspected COPD or asthma only
**LABORATORY ASSESSMENTS: See Appendix 18**	
Blood type, Indirect Antiglobulin Test, and wallet card	Wallet card for study participant identification and results of IAT
Hematology and Chemistry	Local laboratory. Assessments obtained as part of standard of care prior to signing of ICF can be used if obtained within 28 days of starting study drug
HIV, HBV, and HCV screening	Local testing is required if not performed as part of standard of care within 3 months before Cycle 1 Day1; see Section 8.3.5 for additional information
Coagulation	Local laboratory
Serum pregnancy test	For participants of childbearing potential: see Appendix 9
**DISEASE EVALUATIONS: See Section 8.2**	
Albumin	
Serum β2-microglobulin, Quantitative immunoglobulins, SPEP, 24-hour UPEP, Serum FLC and SIFE/UIFE	• Central laboratory. In extenuating circumstances and with approval by the sponsor, local laboratory assessments may be used to establish measurable disease at screening (see Section 5.1).<br>• If the 24-hour urine collection (UPEP) begins the day before informed consent is obtained as part of standard of care, the sample can be used for this study as long as it is sent to the central lab for analysis after the informed consent was obtained.
Fresh bone marrow aspirate samples and bone marrow core biopsy	Disease characterization (morphology and either IHC or flow cytometry for plasma cell clonality) see Table 60<br>**Arms A, A1, B,** [redacted]<br>If a biopsy was done within 42 days before enrollment, no need to repeat for morphology; however, fresh aspirate samples need to be obtained and sent to central laboratory for MRD, cytogenetics, immunophenotype, and/or molecular markers (see Section 8.2.2).<br>**Arms C, C1, and** [redacted]**:**<br>Part of the fresh sample will also be sent to the central laboratory for MRD assessment and/or molecular markers (see Section 8.2.2).
Imaging for disease evaluation	See Section 8.2.4 for acceptable modalities. If performed within 42 days before study inclusion, does not need to be repeated at screening.
Imaging for MRD assessment	All arms: optional for sites where available, DW-MRI (see Section 8.2.4.1)
Assessment of soft-tissue plasmacytomas	See Section 8.2.5 for acceptable modalities and instructions regarding biopsies. If performed within 42 days before study inclusion, does not need to be repeated at screening.
**BIOMARKER SAMPLING (WHOLE BLOOD)**	
Immunophenotyping	
Molecular markers	
**ONGOING REVIEW**	
AEs	Continuous from the time of signing of ICF
Second primary malignancy	
Concomitant therapy (see Section 6.12)	

NOTE: Screening occurs prior to induction treatment for Arms A, A1, B, [redacted], and after induction, HDT, and ASCT for Arms C, C1 and [redacted].

Table 3: Arms A*, A1, [redacted] (Tec-DRd Induction), [redacted] ([redacted] Induction) – SoA for Treatment Phase, EOT Visit, and Posttreatment Follow-up Phase

Assessment	Notes	Treatment Phase (28-day cycle)												Follow-up Phase
Induction Cycle		Cycle 1					Cycles 2-3		Cycles 4-6 (stem cell collection after C3 – Sec 8.1.1.3.1)		End of Induction	HDT + ASCT[a]	EOT (if not continued on maintenance or [for Arm [redacted]] [redacted])	
Day		1	2	4 (+2d)	8 (+2d)	15, 22 (±2d)	1 (±3d)	8, 15, 22 (±3d)	1 (±3d)	15 (±3d)	Within 2wk of completing C6 and [for Arm [redacted]] prior to C1 of [redacted]	Limited data collection Sec 8.1.1.3.3	≤30d after last dose (±7d)	Pre-PD Q12W (±14d) Post-PD Q16W (±28d)
**STUDY PROCEDURES**														
ECOG performance status	See Appendix 6	X					X		X		X			
Physical examination	Symptom-directed	As clinically indicated												
Weight		X					X		X					
Neurologic examination incl. ICE tool	See Section 8.3.7 and Appendix 15 Prior to administration of study drugs.	X	X	As clinically indicated (see Section 6.5.2)										
Vital signs incl. O_2 saturation	See Table 24 and Table 26 for timing	X	X	X	X	X	X	X	X	X	X		X	
12-lead ECG		As clinically indicated												
**LABORATORY ASSESSMENTS: See Appendix 18**														
Hematology	Must be performed ≤72h before dosing. Note: During the step-up phase, laboratory tests must be performed ≤24h prior to the 2nd and 3rd dose. Laboratory values for C1D1 must meet Section 5 criteria. If criteria are not met see Section 5 for guidance or repeated testing requirements.	X	X	X	X	X	X	X	X	X	X		X	
Chemistry		X	X	X	X	X	X	D15	X	X	X		X	
TSH		X							C4					
Creatine kinase	Perform only on participants who are treated with statins and receive concurrent treatment with lenalidomide, from Cycle 2 to Cycle 3, at a minimum.						X	D15	X	X				
TLS-related (uric acid)	Perform ≤72h before dosing See Section 6.5.7 for additional assessments and management of TLS.	X			X	X								
HBV, HCV PCR	• For participants with history of HBV and/or HCV infection. • See Section 8.3.5.3	X							C4				3 and 6 months (±1 month) after last dose	
Coagulation		As clinically indicated (including at CRS onset if applicable)												

Table 3: Arms A*, A1[redacted] (Tec-DRd Induction),[redacted] Induction) – SoA for Treatment Phase, EOT Visit, and Posttreatment Follow-up Phase

Assessment	Notes	Treatment Phase (28-day cycle)												Follow-up Phase
**Induction Cycle**		**Cycle 1**					**Cycles 2-3**		**Cycles 4-6 (stem cell collection after C3 – Sec 8.1.3.1)**		**End of Induction**	**HDT + ASCT[a]**	**EOT (if not continued on maintenance or [for Arm [redacted] [redacted])**	
**Day**		1	2	4 (+2d)	8 (+2d)	15, 22 (±2d)	1 (±3d)	8, 15, 22 (±3d)	1 (±3d)	15 (±3d)	**Within 2wk of completing C6 and [for Arm [redacted]] prior to C1 of [redacted][b]**	Limited data collection Sec 8.1.3.3	**≤30d after last dose (±7d)**	**Pre-PD Q12W (±14d) Post-PD Q16W (±28d)**
Urine Pregnancy Test	• Participants of childbearing potential<br>• Additional testing may be required per the local PPP<br>• Serum pregnancy test is acceptable	• Within 24 hours of C1D1.<br>• 10 to 14 days prior and again within 24 hours prior to 1st dose of lenalidomide.<br>• Weekly for first 4 weeks of lenalidomide treatment, thereafter, Q4W (Q2W for participants with irregular menses) and as clinically indicated.											X	
**STUDY VISITS: See Section 6.4.1 for required safety monitoring through the first 3 doses of teclistamab (0.06, 0.3, and 1.5 mg/kg) or talquetamab (0.01, 0.06, and 0.4 mg/kg) & Appendix 13 for considerations for outpatient administration during this period**														
Study visits	If a visit occurs later than planned, adjust subsequent visit(s) accordingly or skip dose as per Table 45	X	X	X	X	X	X	X	X	X	X		X	X
**DISEASE EVALUATIONS: See Section 8.2 for details regarding evaluations and Section 8.1.1.6 for guidance regarding follow-up requirements.**														
Quantitative immunoglobulins	• Central laboratory (local laboratory assessments may be used under specified circumstances [see Section 8.1.2]). The sample for C1D1 may be collected on or within 3 days prior to C1D1.<br>• **Evaluations will also be done after completion of Cycle 3 (before mobilization procedure)**	X					X		X		X		X (until confirmed PD or SST, whichever occurs earlier)<br>**NOTE:** SPEP and UPEP assessments continue until 30 days after the start of the 1st SST for participants with no PD or unconfirmed PD (ie, single instance of PD by laboratory assessment) while on treatment. For participants with measurable disease by light chain, serum FLC will also continue during this period.	
SPEP		X					X		X		X			
24-hour UPEP		X					X		X		X			
Serum FLC		• For participants with measurable disease by light chain: Day 1 of each cycle<br>• Others: whenever CR or sCR is suspected												
SIFE/UIFE		X	D1 of each cycle and wherever CR or sCR are suspected											
Imaging for disease evaluation	See Section 8.2.4 for acceptable modalities	As clinically indicated to document response or progression												
Assessment of soft-tissue plasmacytoma	See Section 8.2.5 for acceptable modalities and instructions regarding biopsies	• For participants with a history of soft-tissue plasmacytoma<br>-For assessment by physical examination (if applicable), Q4W (±1W) until development of confirmed CR or PD or start of SST<br>-For assessment by radiology, Q12W (±14d) until the plasmacytoma(s) meet CR criteria or confirmed PD or start of SST<br>• As clinically indicated for other participants												
Bone marrow aspirate and core biopsy	See Table 60 (a portion of aspirate should be sent to the central laboratory for biomarker analysis)	• Sampling for MRD will be done after completion of Cycle 3 (before mobilization procedure) and after completion of Cycle 6 in all participants.<br>• Time of suspected CR (including sCR)<br>• Time of PD												
Imaging for MRD assessment (DW-MRI)	Optional for sites where available. See Section 8.2.4.1	• After Cycle 6 of induction treatment												

Table 3: Arms A*, A1, [redacted] (Tec-DRd Induction), [redacted] Induction) – SoA for Treatment Phase, EOT Visit, and Posttreatment Follow-up Phase

Assessment	Notes	Treatment Phase (28-day cycle)												Follow-up Phase
**Induction Cycle**		**Cycle 1**					**Cycles 2-3**		**Cycles 4-6 (stem cell collection after C3 – Sec 8.1.3.1)**		**End of Induction**	**HDT + ASCT[a]**	**EOT (if not continued on maintenance or [for Arm [redacted]] [redacted]**	
**Day**		**1**	**2**	4 (+2d)	8 (+2d)	15, 22 (±2d)	1 (±3d)	8, 15, 22 (±3d)	1 (±3d)	15 (±3d)	**Within 2wk of completing C6 and [for Arm [redacted]] prior to C1 of [redacted][b]**	Limited data collection Sec 8.1.3.3	≤30d after last dose (±7d)	**Pre-PD Q12W (±14d) Post-PD Q16W (±28d)**
**PRO: PRO assessments should be completed before any clinical tests or procedures**														
[redacted]	[redacted]													
**ONGOING REVIEW**														
AEs		• Continue until 30 days after last dose of study treatment or until the start of SST, whichever comes first. Continue to report any (S)AEs related to study treatment until EOS.<br>• Limited (S)AE reporting required during HDT+ASCT, see Section 8.1.3.4												
2nd primary malignancy		Continuous until EOS.												
Concomitant therapy	See Section 6.12	• Continuous until 30 days after last dose of study treatment or until the start of SST, whichever comes first. Continue to report concomitant therapy given for any (S)AEs considered related to study treatment until EOS.<br>• During HDT+ASCT, record only the concomitant treatments used to treat AE specified in Section 8.1.3.4												
SST	See Section 6.13													X (post-PD)
Survival							Continuous							Q 16 wk

* Refer to Appendix 22 for dose schedule of Arm A (Table 64) in previous teclistamab weekly dosing schedule.

a. Not applicable for [redacted]. Participants in [redacted] will receive [redacted] treatment as replacement for HDT+ASCT.

b. If a participant in [redacted] more than 28 days after induction, all End of Induction disease evaluations (except for bone marrow aspirate and imaging for MRD) must be repeated.

c. [redacted] Only if oral toxicity (AEs) is present at EOT and until resolution.

Table 4: Arms B (Tec-DVRd Induction), F[redacted] Induction) – SoA for Treatment Phase, EOT Visit, and Posttreatment Follow-up Phase

Assessment	Notes	Treatment Phase (28-day cycle)										Follow-up Phase
Induction Cycle		Cycle 1					Cycles 2-6 (stem cell collection after C3 per Sec 8.1.1.3.1)		End of Induction	HDT + ASCT	EOT	(if not continued on maintenance)
Day		1	2	4 (+2d)	8 (+2d)	15 (+2d) 22 (+2d)	1 (±3d)	8, 15, 22 (±3d)	Within 2wk of completing C6	Limited data collection; Sec 8.1.1.3.3	≤30d after last dose (+7d)	Pre-PD Q12W (±14d) Post-PD Q16W (±28d)
**STUDY PROCEDURES**												
ECOG performance status	See Appendix 6	X					X		X			
Physical examination	Symptom-directed	As clinically indicated										
Weight		X					X					
Neurologic examination incl. ICE tool	See Section 8.3.7 and Appendix 15<br>Prior to administration of study drugs.	X	X	As clinically indicated (see Section 6.5.2)								
Vital signs incl. O_2 saturation	See Table 24 and Table 26 for timing	X	X	X	X	X	X	X	X		X	
12-lead ECG		As clinically indicated										
**LABORATORY ASSESSMENTS: See Appendix 18**												
Hematology	Must be performed ≤72 hours before dosing.<br>Note: During the step-up phase, laboratory tests must be performed ≤24 hours prior to the 2nd and 3rd dose. Laboratory values for C1D1 must meet Section 5 criteria. If criteria are not met see Section 5 for guidance or repeated testing requirements.	X	X	X	X	X	X	D8, D15, D22	X		X	
Chemistry		X	X	X	X	X	X	D15	X		X	
TSH		X					C4					
Creatine kinase	Perform only on participants who are treated with statins and receive concurrent treatment with lenalidomide, from Cycle 2 to Cycle 3, at a minimum.						X	D15				
TLS-related (uric acid)	Perform ≤72 hours before dosing<br>See Section 6.5.7 for additional assessments and management of TLS.	X			X	X						
HBV, HCV PCR	• For participants with history of HBV and/or HCV infection.<br>• See Section 8.3.5.3	X					C4				3 and 6 months (±1 month) after last dose	
Coagulation		As clinically indicated (including at CRS onset if applicable)										
Urine Pregnancy Test	• Participants of childbearing potential<br>• Additional testing may be required per the local PPP<br>• Serum pregnancy test is acceptable	• Within 24 hours of C1D1.<br>• 10 to 14 days prior and again within 24 hours prior to 1st dose of lenalidomide.<br>• Weekly for first 4 weeks of lenalidomide treatment, thereafter, Q4W (Q2W for participants with irregular menses) and as clinically indicated.									X	

Table 4: Arms B (Tec-DVRd Induction), [redacted] Induction) – SoA for Treatment Phase, EOT Visit, and Posttreatment Follow-up Phase

Assessment	Notes	Treatment Phase (28-day cycle)										Follow-up Phase
Induction Cycle		Cycle 1					Cycles 2-6 (stem cell collection after C3 per Sec 8.1.1.3.1)		End of Induction	HDT + ASCT	EOT (if not continued on maintenance)	
Day		1	2	4 (+2d)	8 (+2d)	15 (+2d) 22 (+2d)	1 (±3d)	8, 15, 22 (±3d)	Within 2wk of completing C6	Limited data collection; Sec 8.1.1.3.3	≤30d after last dose (+7d)	Pre-PD Q12W (±14d) Post-PD Q16W (±28d)
**STUDY VISITS: See Section 6.4.1 for required safety monitoring through the first 3 doses of teclistamab (0.06, 0.3, and 1.5 mg/kg) or talquetamab (0.01, 0.06, and 0.4 mg/kg) & Appendix 13 for considerations for outpatient administration during this period**												
Study visits	If a visit occurs later than planned, adjust subsequent visit(s) accordingly or skip dose per Table 45	X	X	X	X	X	X	X	X		X	X
**DISEASE EVALUATIONS: See Section 8.2 for details regarding evaluations and Section 8.1.1.6 for guidance regarding follow-up requirements.**												
Quantitative immunoglobulins	• Central laboratory (local laboratory assessments may be used under specified circumstances [see Section 8.1.2]). The sample for C1D1 may be collected on or within 3 days prior to C1D1.<br>• **Evaluations will also be done after completion of Cycle 3 (before mobilization procedure).**	X					X		X		X (until confirmed PD or SST, whichever occurs earlier)<br>**NOTE**: SPEP and UPEP assessments will continue until 30 days after the start of the 1st SST for participants with no PD or unconfirmed PD (ie, single instance of PD by laboratory assessment) while on treatment. For participants with measurable disease by light chain, serum FLC will also continue during this period.	
SPEP		X					X		X			
24-hour UPEP		X					X		X			
Serum FLC		• For participants with measurable disease by light chain: Day 1 of each cycle<br>• Others: whenever CR or sCR is suspected										
SIFE/UIFE		X	D1 of each cycle and wherever CR or sCR are suspected									
Imaging for disease evaluation	See Section 8.2.4 for acceptable modalities	As clinically indicated to document response or progression										
Assessment of soft-tissue plasmacytoma	See Section 8.2.5 for acceptable modalities and instructions regarding biopsies	• For participants with a history of soft-tissue plasmacytoma<br>-For assessment by physical examination (if applicable), Q4W (±1W) until development of confirmed CR or PD or start of SST<br>-For assessment by radiology, Q12W (±14d) until the plasmacytoma(s) meet CR criteria or confirmed PD or start of SST<br>• As clinically indicated for other participants										
Bone marrow aspirate and core biopsy	See Table 60 (a portion of aspirate should be sent to the central laboratory for biomarker analysis)	• Sampling for MRD will be done after completion of Cycle 3 (before mobilization procedure and after completion of Cycle 6 in all participants<br>• Time of suspected CR (including sCR)<br>• Time of PD										
Imaging for MRD assessment (DW-MRI)	Optional for sites where available. See Section 8.2.4.1	• After Cycle 6 of induction treatment										
**PRO: PRO assessments should be completed before any clinical tests or procedures**												

[redacted]

Table 4: Arms B (Tec-DVRd Induction), [redacted] Induction) – SoA for Treatment Phase, EOT Visit, and Posttreatment Follow-up Phase

Assessment	Notes	Treatment Phase (28-day cycle)										Follow-up Phase
Induction Cycle		Cycle 1					Cycles 2-6 (stem cell collection after C3 per Sec 8.1.1.3.1)		End of Induction	HDT + ASCT	EOT (if not continued on maintenance)	
Day		1	2	4 (+2d)	8 (+2d)	15 (+2d) 22 (+2d)	1 (±3d)	8, 15, 22 (±3d)	Within 2wk of completing C6	Limited data collection; Sec 8.1.1.3.3	≤30d after last dose (+7d)	Pre-PD Q12W (±14d) Post-PD Q16W (±28d)
**ONGOING REVIEW**												
AEs		• Continue until 30 days after last dose of study treatment or until the start of SST, whichever comes first. Continue to report any (S)AEs related to study treatment until EOS.<br>• Limited (S)AE reporting required during HDT+ASCT, see Section 8.1.1.3.4										
2nd primary malignancy		Continuous until EOS.										
Concomitant therapy	See Section 6.12	• Continuous until 30 days after last dose of study treatment or until the start of subsequent therapy, whichever comes first. Continue to report concomitant therapy given for any (S)AEs considered related to study treatment until EOS.<br>• During HDT+ASCT, record only the concomitant treatments used to treat AE specified in Section 8.1.1.3.4.										
SST	See Section 6.13											X (post-PD)
Survival		Continuous										Q 16 wk

a. [redacted] Only if oral toxicity (AEs) is present at EOT and until resolution.

Table 5: Arm[redacted] ([redacted] Following Induction) – SoA for Treatment Phase, EOT Visit, and Posttreatment Follow-up Phase

Assessments	Notes	[redacted] Treatment Phase (28-day cycle)										Follow-up Phase (Incl. Potential SoC Maintenance)
**Treatment Cycle**		**Cycle 1**					**Cycle 2**		**Cycle 3-18**		**EOT**	
**Day**		**1**	**2**	**4 (+2d)**	**8 (+2d)**	**15 (+2d) 22 (±2d)**	**1 (±3d)**	**8, 15, 22 (±2d)**	**1 (±3d)**	**15 (±3d)**	**≤30d after last dose (+7d)**	**Pre-PD Q12wk (±14d) Post-PD Q16wk (±28d)**
**STUDY PROCEDURES**												
ECOG performance status	See Appendix 6	X					X		X		X	
Physical examination	Symptom-directed	As clinically indicated										
Weight		X					X		X			
Neurologic examination incl. ICE Tool	See Section 8.3.7 and Appendix 15<br>Prior to administration of study drugs.	X	X	As clinically indicated (see Section 6.5.2)								
Vital signs incl. O_2 saturation	See Table 28 for timing in relation to dosing	X	X	X	X	D15	X		X		X	
12-lead ECG		As clinically indicated										
**LABORATORY ASSESSMENT: See Appendix 18**												
Hematology	Must be performed ≤72 hours before dosing. Note: During the step-up phase, laboratory tests must be performed ≤24 hours prior to the 2nd and 3rd dose. Laboratory values for C1D1 dose must meet Section 5.1 criteria. If criteria are not met see Section 5.1 for guidance or repeated testing requirements.	X	X	X	X	X	X	X	X	C3, 4	X	
Chemistry		X	X	X	X	X	X	D15	X	C3, 4: as clinically indicated	X	
HBV, HCV PCR	• For participants with history of HBV and/or HCV infection.<br>• See Section 8.3.5.3	X							C4, 7, 10, 13, 16		3 and 6 months (±1 month) after last dose	
Coagulation		As clinically indicated (including at CRS onset if applicable)										
Urine pregnancy test	• Participants of childbearing potential<br>• Serum pregnancy test is acceptable	• Within 24 hours of C1D1, Q4W during treatment (Q2W for participants with irregular menses), and as clinically indicated.									X	
**STUDY VISITS: See Section 6.4.1 for required safety monitoring through the first 3 doses of talquetamab (0.01, 0.06, and 0.4 mg/kg) & Appendix 13 for considerations for outpatient administration during this period**												
Study visit	If a visit occurs later than planned, adjust subsequent visit(s) accordingly or skip dose as per Table 46	X	X	X	X	X	X	X	X	C3, 4	X	X
**DISEASE EVALUATIONS: See Section 8.2 for details regarding evaluations and Section 8.1.1.6 for guidance regarding follow-up requirements.**												
Quantitative immunoglobulins	Central laboratory (local laboratory assessments may be used under specified circumstances [see Section 8.1.2]). The sample for C1D1 may be collected on or within 3 days prior to C1D1.	X					X		X		X (until confirmed PD or SST, whichever occurs earlier)<br>**NOTE**: continue SPEP and UPEP assessments will continue until 30 days after the start of the 1st SST for	
SPEP		X					X		X			
24-hour UPEP		X					X		X			
Serum FLC		• For participants with measurable disease by light chain: Day 1 of each cycle<br>• Others: Whenever CR or sCR is suspected										
SIFE/UIFE		X	Day 1 of each cycle and whenever CR or sCR are suspected									

Table 5: Arm [redacted] ([redacted] Following Induction) – SoA for Treatment Phase, EOT Visit, and Posttreatment Follow-up Phase

Assessments	Notes	[redacted] Treatment Phase (28-day cycle)										Follow-up Phase (Incl. Potential SoC Maintenance)
Treatment Cycle		Cycle 1					Cycle 2		Cycle 3-18		EOT	
Day		1	2	4 (+2d)	8 (+2d)	15 (+2d) 22 (±2d)	1 (±3d)	8, 15, 22 (±2d)	1 (±3d)	15 (±3d)	≤30d after last dose (+7d)	Pre-PD Q12wk (±14d) Post-PD Q16wk (±28d)
Imaging for disease evaluation	See Section 8.2.4 for acceptable modalities	• As clinically indicated to document response or progression										participants with no PD or unconfirmed PD (ie, single instance of PD by lab assessment) while on treatment. For participants with measurable disease by light chain, serum FLC will also continue during this period.
Assessment of soft-tissue plasmacytomas	See Section 8.2.5 for acceptable modalities and instructions regarding biopsies	• For participants with a history of soft-tissue plasmacytoma<br>-For assessment by physical examination (if applicable), Q4W (±1W) until development of confirmed CR or PD or start of SST<br>-For assessment by radiology, Q12W (±14d) until the plasmacytoma(s) meet CR criteria or confirmed PD or start of SST<br>• As clinically indicated for other participants										
Bone marrow aspirate	See Table 60 (a portion of aspirate should be sent to the central laboratory for MRD assessment)	• For participants who have not progressed, and remain on study, an additional bone marrow aspirate will be obtained at 6, 12, 18, and 24 months (±1 month) post start of [redacted] treatment. For participants who remain on study drug, cycles should be used to guide MRD sampling (ie, 6 months is after Cycle 6)<br>• Time of suspected CR (including sCR)<br>• Time of PD										
Imaging for MRD (DW-MRI)	Optional for sites where available. See Section 8.2.4.1	• After 18 cycles of [redacted] treatment										
**PRO: PRO assessments should be completed before any clinical tests or procedures**												
[redacted]												
**ONGOING REVIEW**												
AEs		Continuous until 30 days after last dose of study treatment or until the start of subsequent therapy, whichever comes first. Continue to report any (S)AEs related to study treatment until EOS.										
2nd primary malignancy		Continuous until EOS										
Concomitant therapy	See Section 6.12	Continuous until 30 days after last dose of study treatment or until the start of SST, whichever comes first. Continue to report concomitant therapy given for any (S)AEs considered related to study treatment until EOS										
SoC Maintenance	Per institutional standard and local investigator decision. See Section 8.1.1.6.1											If applicable, document treatment regimen and reason for discontinuation or changes in SoC maintenance
SST	See Section 6.13. SoC maintenance without PD is not regarded as SST											X (post-PD)
Survival		Continuous										X

a. [redacted] Only if oral toxicity (AEs) is present at EOT and until resolution.

Table 6: Arms A and C* (Tec-DR or Tec-D Maintenance), Arms A1, B, C1, [redacted], and [redacted] (Tec-D Maintenance), Arm [redacted] Maintenance), and Arms [redacted] Maintenance) – SoA for Treatment Phase, EOT Visit, and Posttreatment Follow-up Phase

Assessments	Notes	Pre Mainten-ance	Maintenance Treatment Phase (28-day cycle)										Follow-up Phase (Incl. Potential SoC Maintenance)
Maintenance Cycle			Cycle 1					Cycle 2		Cycle 3-18		EOT	
Day		Within 28d prior to starting maintenance	1	2	4 (+2d)	8 (+2d)	15 (+2d) 22 (±2d)	1 (±3d)	8, 15, 22 (±2d)	1 (±3d)	15 (±3d)	≤30d after last dose (+7d)	Pre-PD Q12wk (±14d) Post-PD Q16wk (±28d)
**STUDY PROCEDURES**													
ECOG performance status	See Appendix 6		X					X		X		X	
Physical examination	Symptom-directed		As clinically indicated										
Weight			X					X		X			
Neurologic examination incl. ICE Tool	See Section 8.3.7 and Appendix 15 Prior to administration of study drugs.		X	X	As clinically indicated (see Section 6.5.2)								
Vital signs incl. O_2 saturation	See Table 30, Table 32, Table 34, and Table 36 for timing in relation to dosing		X	X	X	X	D15	X		X		X	
12-lead ECG			As clinically indicated										
**LABORATORY ASSESSMENT: See Appendix 18**													
Hematology	Must be performed ≤72 hours before dosing. Note: During the step-up phase, laboratory tests must be performed ≤24 hours prior to the 2nd and 3rd dose. Laboratory values for C1D1 dose must meet Section 5.1 criteria. If criteria are not met see Section 5.1 for guidance or repeated testing requirements.		X	X	X	X	X	X	X	X	C3, 4	X	
Chemistry	**For Arms A, C,** [redacted], participants who have not previously received lenalidomide as part of induction or consolidation should have a complete blood cell count, including white blood cell count with differential count, platelet count, hemoglobin, and hematocrit performed at baseline and every week for the first 8 weeks of lenalidomide treatment and Q2W thereafter.		X	X	X	X	X	X	D15	X	C3, 4: as clinically indicated	X	
TSH ARMS A, C, and [redacted] ONLY			X										

Table 6: Arms A and C* (Tec-DR or Tec-D Maintenance), Arms A1, B, C1, [redacted], and [redacted] (Tec-D Maintenance), Arm [redacted] ([redacted] Maintenance), and Arms [redacted] and [redacted] ([redacted] Maintenance) – SoA for Treatment Phase, EOT Visit, and Posttreatment Follow-up Phase

Assessments	Notes	Pre Mainten-ance	Maintenance Treatment Phase (28-day cycle)										Follow-up Phase (Incl. Potential SoC Maintenance)
Maintenance Cycle			Cycle 1					Cycle 2		Cycle 3-18		EOT	
Day		Within 28d prior to starting maintenance	1	2	4 (+2d)	8 (+2d)	15 (+2d) 22 (±2d)	1 (±3d)	8, 15, 22 (±2d)	1 (±3d)	15 (±3d)	≤30d after last dose (+7d)	Pre-PD Q12wk (±14d) Post-PD Q16wk (±28d)
Creatine kinase ARMS A, C, and [redacted] ONLY	Perform only on participants who are treated with statins and receive concurrent treatment with lenalidomide, from Cycle 2 to Cycle 3, at a minimum.							X	D15	X	C3, 4		
HBV, HCV PCR	• For participants with history of HBV and/or HCV infection.<br>• See Section 8.3.5.3		X							C4, 7, 10, 13, 16		3 and 6 months (±1 month) after last dose	
Coagulation			As clinically indicated (including at CRS onset if applicable)										
Urine pregnancy test	• Participants of childbearing potential<br>• Additional testing may be required per the local PPP<br>• Serum pregnancy test is acceptable		• Within 24 hours of C1D1.<br>• 10 to 14 days prior and again within 24 hours prior to first dose of lenalidomide (Arms A, C, [redacted]).<br>• Weekly for first 4 weeks of lenalidomide treatment (Arms A, C, [redacted]), thereafter, Q4W (Q2W for participants with irregular menses), and as clinically indicated.									X	
**STUDY VISITS: See Section 6.4.1 for required safety monitoring through the first 3 doses of teclistamab (0.06, 0.3, and 1.5 mg/kg) or talquetamab (0.01, 0.06, and 0.4 mg/kg) & Appendix 13 for considerations for outpatient administration during this period**													
Study visit	If a visit occurs later than planned, adjust subsequent visit(s) accordingly or skip dose as per Table 47.		X	X	X	X	X	X	X	X	C3, 4	X	X
**DISEASE EVALUATIONS: See Section 8.2 for details regarding evaluations and Section 8.1.1.6 for guidance regarding follow-up requirements.**													
Quantitative immunoglobulins	Central laboratory (local laboratory assessments may be used under specified circumstances [see Section 8.1.2]). The sample for C1D1 may be collected on or within 3 days prior to C1D1.	X	X					X		X		X (until confirmed PD or SST, whichever occurs earlier)<br>**NOTE**: SPEP and UPEP assessments will continue until 30 days after the start of the first SST for participants with no PD or unconfirmed PD (ie, single instance of PD by laboratory assessment) while on treatment. For participants with measurable disease by light chain, serum FLC will also continue during this period.	
SPEP		X	X					X		X			
24-hour UPEP		X	X					X		X			
Serum FLC		X	• For participants with measurable disease by light chain: Day 1 of each cycle<br>• Others: Whenever CR or sCR is suspected										
SIFE/UIFE		X	X	Day 1 of each cycle and whenever CR or sCR are suspected									
Imaging for disease evaluation	See Section 8.2.4 for acceptable modalities		• As clinically indicated to document response or progression										
Assessment of soft-tissue plasmacytomas	See Section 8.2.5 for acceptable modalities and instructions regarding biopsies		• For participants with a history of soft-tissue plasmacytoma<br>-For assessment by physical examination (if applicable), every 4 weeks (±1 week) until development of confirmed CR or PD or start of SST<br>-For assessment by radiology, Q12W (±14 days) until the plasmacytoma(s) meet CR criteria or confirmed PD or start of SST<br>• As clinically indicated for other participants										

Table 6: Arms A and C* (Tec-DR or Tec-D Maintenance), Arms A1, B, C1, [redacted] (Tec-D Maintenance), Arm [redacted] Maintenance), and Arms [redacted] and [redacted] ([redacted] Maintenance) – SoA for Treatment Phase, EOT Visit, and Posttreatment Follow-up Phase

Assessments	Notes	Pre Mainten-ance	Maintenance Treatment Phase (28-day cycle)										Follow-up Phase (Incl. Potential SoC Maintenance)
Maintenance Cycle			Cycle 1					Cycle 2		Cycle 3-18		EOT	
Day		Within 28d prior to starting maintenance	1	2	4 (+2d)	8 (+2d)	15 (+2d) 22 (±2d)	1 (±3d)	8, 15, 22 (±2d)	1 (±3d)	15 (±3d)	≤30d after last dose (+7d)	Pre-PD Q12wk (±14d) Post-PD Q16wk (±28d)
Bone marrow aspirate	See Table 60 (a portion of aspirate should be sent to the central laboratory for MRD assessment)	X (for MRD sampling)	• For participants who have not progressed, and remain on study, an additional bone marrow aspirate will be obtained at 6, 12, 18, and 24 months (±1 month) post start of maintenance. For participants who remain on study drug, cycles should be used to guide MRD sampling (ie, 6 months is after Maintenance Cycle 6)<br>• Time of suspected CR (including sCR)<br>• Time of PD										
Imaging for MRD (DW-MRI)	Optional for sites where available. See Section 8.2.4.1	X	• After 18 cycles of maintenance										
PRO: PRO assessments should be completed before any clinical tests or procedures													
[redacted]													
ONGOING REVIEW													
AEs			Continuous until 30 days after last dose of study treatment or until the start of subsequent therapy, whichever comes first. Continue to report any (S)AEs related to study treatment until EOS.										
2nd primary malignancy			Continuous until EOS										
Concomitant therapy	See Section 6.12		Continuous until 30 days after last dose of study treatment or until the start of SST, whichever comes first. Continue to report concomitant therapy given for any (S)AEs considered related to study treatment until EOS										
SoC Maintenance	Per institutional standard and local investigator decision. See Section 8.1.1.6.1												If applicable, document treatment regimen and reason for discontinuation or changes in SoC maintenance
SST	See Section 6.13. SoC maintenance without PD is not regarded as SST												X (post-PD)
Survival			Continuous										X

* Refer to Appendix 22 for dose schedule of Arm C (Table 65) in previous teclistamab biweekly dosing schedule.

a. [redacted] Only if oral toxicity (AEs) is present at EOT and until resolution

Table 7: Arms A*, A1, and [redacted] (Tec-DRd Induction), Arm B (Tec-DVRd Induction), Arms [redacted] and [redacted] ([redacted] Induction), and Arms [redacted] and [redacted] ([redacted] Induction) – Schedule of PK, Immunogenicity, and Biomarker Sample Collection

Assessment	Time	Induction Treatment Phase (28-day cycle)										Follow-up Phase
Induction Cycle		Cycle 1					Cycles 2-6 (stem cell collection after Cycle 3 as per Section 8.1.1.3.1)		End of Induction	HDT + ASCT[d]	EOT (if not continued on main-tenance or [for Arm [redacted]] [redacted]	(if not continued on maintenance or [for Arm [redacted] [redacted]
Day		1	2	4 (+2d)	8 (+2d)	15 (+2d)	1 (±3d)	8, 15, 22 (±7d)	2 weeks after completing Cycle 6	Limited data collection (Section 8.1.1.3.3)	≤30d after last dose	Pre-PD Q12wk (±14d) Post-PD Q16wk (±28d)
**PHARMACOKINETICS AND IMMUNOGENICITY SAMPLING**												
Teclistamab or talquetamab	Prior to first dose of daratumumab (-2 h)	X										
	Predose (prior to teclistamab or talquetamab dose) on day of dosing[a,b]				X (PK only)	X (PK only)	C3 and C6				X	X[c]
	Suspected sARR Grade ≥2	Collect additional PK/immunogenicity sample as soon as sARR is detected, if feasible										
	Suspected CRS or teclistamab- or talquetamab-related neurotoxicity Grade ≥2	Collect additional PK/immunogenicity sample as soon as CRS or neurotoxicity is detected, if feasible										
**BIOMARKER SAMPLING (BLOOD): See Section 8.7. For bone marrow aspirate, see Table 3, Table 4, and Table 60 for Disease Evaluation**												
Immunophenotyping (whole blood)[a]	Time-based									X	X	
		• In all participants, along with bone marrow collection, after completion of Induction Cycle 3 (before mobilization) and after Induction Cycle 6										
	Response-based	• Time of suspected CR or sCR (unless this occurs within 1 month of another sample)<br>• Time of PD										
Molecular markers (whole blood)[a]	Time-based									X	X	
		• In all participants, along with bone marrow collection, after completion of Induction Cycle 3 (before mobilization and after Induction Cycle 6.										
	Response-based	• Time of suspected CR or sCR (unless this occurs within 1 month of another sample)<br>• Time of PD										
Biopsy of soft-tissue plasmacytomas	As clinically indicated	See Section 8.2.5										

* Refer to Appendix 22 (Table 66) for schedule of PK, immunogenicity, and biomarker sample collection for Arm A in previous teclistamab weekly dosing schedule.

a. Sample collection should occur within 4 hours before teclistamab or talquetamab dosing, if feasible.

b. If predose samples are drawn on the day of planned dosing prior to the determination of a dose delay (eg, due to lab values), PK samples do not need to be recollected.

c. PK sample will only be taken once, at the first follow-up visit.

d. Not applicable for [redacted] Participants in [redacted] treatment as replacement for HDT+ASCT.

Table 8: [redacted] Following Induction) – Schedule of PK, Immunogenicity, and Biomarker Sample Collection

Assessments	Time	[redacted] Treatment Phase (28-day cycle)										Follow-up Phase (Including Potential SoC Maintenance)
Treatment Cycle		Cycle 1					Cycle 2		Cycles 3-18		EOT	
Day		1	2	4 (+2d)	8 (+2d)	15 (+2d),	1 (±3d)	8, 15, 22 (±2d)	1 (±3d)	15 (±7d)	≤30d after last dose	Pre-PD Q12wk (±14d) Post-PD Q16wk (±28d)
**PHARMACOKINETICS AND IMMUNOGENICITY SAMPLING**												
Teclistamab and talquetamab	Predose on day of teclistamab and talquetamab dosing[a,b]	X			X (PK only)	X (PK only)			C3, C6, C12, C18		X	X[c]
	Suspected sARR Grade ≥2	Collect additional PK/immunogenicity sample as soon as sARR is detected, if feasible										
	Suspected CRS or teclistamab- or talquetamab-related neurotoxicity event Grade ≥2	Collect additional PK/immunogenicity sample as soon as CRS or neurotoxicity is detected, if feasible										
**BIOMARKER SAMPLING (BLOOD): See Section 8.7. For bone marrow aspirate, see Table 5 and Table 60 for Disease Evaluation**												
Immunophenotyping (whole blood)[a]	Time-based										X	
	Response-based	• For participants who have not progressed and remain on study, at 6, 12, 18, and 24 months (±1 month) post start of [redacted] treatment.<br>• Time of suspected CR or sCR (unless this occurs within 1 month of another sample)<br>• Time of PD										
Molecular markers (whole blood)[a]	Time-based										X	
	Response-based	• For participants who have not progressed and remain on study, at 6, 12, 18, and 24 months (±1 month) post start of [redacted] treatment.<br>• Time of suspected CR or sCR (unless this occurs within 1 month of another sample)<br>• Time of PD										
Biopsy of soft-tissue plasmacytomas	As clinically indicated	See Section 8.2.5										

a. Sample collection should occur within 4 hours before teclistamab and talquetamab dosing, if feasible.
b. If predose samples are drawn on the day of planned dosing prior to the determination of a dose delay (eg, due to lab values), PK samples do not need to be recollected.
c. PK sample will only be taken once, at the first follow-up visit.

Table 9: Arms A and C* (Tec-DR or Tec-D Maintenance), Arms A1, B, C1, [redacted] and [redacted], (Tec-D Maintenance), Arms [redacted] and [redacted] Maintenance), and Arm [redacted] Maintenance) – Schedule of PK, Immunogenicity, and Biomarker Sample Collection

Assessments	Time	Maintenance Treatment Phase (28-day cycle)										Follow-up Phase (Including Potential SoC Maintenance)
Maintenance Cycle		Cycle 1					Cycle 2		Cycles 3-18		EOT	
Day		1	2	4 (+2d)	8 (+2d)	15 (+2d),	1 (±3d)	8, 15, 22 (±2d)	1 (±3d)	15 (±7d)	≤30d after last dose	Pre-PD Q12wk (±14d) Post-PD Q16wk (±28d)
**PHARMACOKINETICS AND IMMUNOGENICITY SAMPLING**												
Teclistamab or talquetamab	Prior to first dose of daratumumab (-2 h)	X										
	Predose (prior to teclistamab or talquetamab dose) on day of dosing[a,b]				X (PK only)	X (PK only)			C3, C6, C12, C18		X	X[c]
	Suspected sARR Grade ≥2	Collect additional PK/immunogenicity sample as soon as sARR is detected, if feasible										
	Suspected CRS or teclistamab- or talquetamab-related neurotoxicity event Grade ≥2	Collect additional PK/immunogenicity sample as soon as CRS or neurotoxicity is detected, if feasible										
Daratumumab	Predose (prior to daratumumab dose) on day of dosing	X					X		C6			
**BIOMARKER SAMPLING (BLOOD): See Section 8.7. For bone marrow aspirate, see Table 6 and Table 60 for Disease Evaluation**												
Immunophenotyping (whole blood)[a]	Time-based										X	
	Response-based	• For participants who have not progressed and remain on study, at 6, 12, 18, and 24 months (±1 month) post start of maintenance.<br>• Time of suspected CR or sCR (unless this occurs within 1 month of another sample)<br>• Time of PD										
Molecular markers (whole blood)[a]	Time-based										X	
	Response-based	• For participants who have not progressed and remain on study, at 6, 12, 18, and 24 months (±1 month) post start of maintenance.<br>• Time of suspected CR or sCR (unless this occurs within 1 month of another sample)<br>• Time of PD										
Biopsy of soft-tissue plasmacytomas	As clinically indicated	See Section 8.2.5										

* Refer to Appendix 22 (Table 67) for dose schedule PK, immunogenicity, and biomarker sample collection for Arm C in previous teclistamab biweekly dosing schedule.

a. Sample collection should occur within 4 hours before teclistamab or talquetamab dosing, if feasible.

b. If predose samples are drawn on the day of planned dosing prior to the determination of a dose delay (eg, due to lab values), PK samples do not need to be recollected.

c. PK sample will only be taken once, at the first follow-up visit.

2. INTRODUCTION

The term "study treatment" used throughout this document refers to the combination of:

- Teclistamab SC and daratumumab SC, lenalidomide PO, and dexamethasone PO (Tec-DRd) for induction in Arms A, A1, [redacted]
- Teclistamab SC and daratumumab SC, bortezomib SC, lenalidomide PO, and dexamethasone PO (Tec-DVRd) for induction in Arm B;
- [redacted]
- Teclistamab SC, daratumumab SC, and lenalidomide PO (Tec-DR) for maintenance in Arms A and C;
- Teclistamab SC and daratumumab SC (Tec-D) for maintenance in Arms A, A1, B, C, C1, [redacted];
- [redacted]

The term "sponsor" used throughout this document refers to the entities listed in the Contact Information page(s), which will be provided as a separate document.

The term "participant" throughout the protocol refers to the common term "subject."

2.1. Study Rationale

2.1.1. Multiple Myeloma

Multiple myeloma is a mostly incurable, malignant, plasma cell disorder. It is the second most common hematologic malignancy, with 35,842 cases and approximately 23,275 deaths estimated in the 27 European (EU-27) countries in 2020 (ECIS 2020). The estimated crude incidence for the EU-27 countries was 0.80 per 10,000 persons (ECIS 2020). Overall, the estimated 5-year survival rate for patients with multiple myeloma in Europe is approximately 50% (CancerMPact 2018), with the most recent estimates of 51.3% in the Swedish Myeloma Registry in 2014 (Turesson 2018). Survival after diagnosis differs by age, with a recent global review reporting a median survival among patients diagnosed at less than 65 years ranging from <2.75 to 5.42 years and the median survival among patients diagnosed at 65 years and older ranging from 2.17 to 2.67 years (Turesson 2018). A conservative prevalence estimate for multiple myeloma can be calculated using the highest median survival duration of 5.42 years among patients aged less than 65 years, giving an estimated prevalence of 4.34 per 10,000 persons (0.80 per 10,000 * 5.42 years = 4.34 per 10,000).

Multiple myeloma is characterized by proliferation of neoplastic clones of plasma cells derived from B cells. These neoplastic clones grow in the bone marrow, frequently invade adjacent bone, disrupt both bone homeostasis and hematopoiesis, and cause multifocal destructive lesions throughout the skeleton that result in bone pain and fracture (Chung 2017). Common clinical presentations of multiple myeloma are hypercalcemia, renal insufficiency, anemia, bony lesions, bacterial infections, hyperviscosity, and secondary amyloidosis (Orlowski 2013).

Despite multiple therapeutic options, the disease most often recurs and remains incurable. With each successive relapse, symptoms return, quality of life worsens, and the chance and duration of response typically decreases. Therefore, there remains a significant and critical unmet need for new therapeutic options directed at alternative mechanisms of action that can better control the disease, provide deeper, more sustained responses, and yield better long-term outcomes including maintenance of health-related quality of life (Usmani 2016).

2.1.2. Treatment Options for Patients with Newly Diagnosed Multiple Myeloma

Over recent decades, several new drugs have been developed for the treatment of multiple myeloma, including IMiDs (lenalidomide, thalidomide, and pomalidomide), PIs (bortezomib, carfilzomib or ixazomib), anti-CD38 monoclonal antibodies (daratumumab and isatuximab), an ADC targeting BCMA (belantamab mafodotin), BCMA-directed genetically modified autologous T-cell immunotherapy (idecabtagene vicleucel and ciltacabtagene autoleucel), and selective inhibitors of nuclear export (selinexor). These developments have substantially improved outcomes in a significant number of patients; however, treatment remains non-curative and ultimately relapse is inevitable. With each successive relapse, the molecular and genetic complexity increases and the depth and duration of response decreases, resulting in refractory disease. Furthermore, many patients who progress on initial therapy are not eligible to receive second-line therapy. Recent reports suggest that attrition rates between the first and second line of therapy are as high as 50% in patients with multiple myeloma (Fonseca 2020; Venner 2015; Yong 2016) and achieving the longest possible PFS with the first-line of therapy drives overall survival outcomes (Fonseca 2021). Therefore, efforts to improve initial treatment are critical, as the disease is most sensitive to therapy at the time of diagnosis and provides the highest probability of achieving a long disease-free interval. In younger, fit patients, induction with high-dose melphalan with ASCT remains an important treatment strategy and continues to demonstrate a significant improvement in PFS in the era of novel drugs (Dimopoulos 2021a; Hamed 2019; NCCN 2022).

Induction Therapy

Patients with ND-TEMM are treated with an induction regimen to reduce the plasma cell disease burden and improve the depth of response. Response rates to induction therapy have been significantly increased using triple combination chemotherapy. Bortezomib-based induction regimens including VRd, VCd, and VTd improve response and prolong PFS/OS compared with the same regimens without bortezomib.

Daratumumab in combination with therapies commonly used for the treatment of multiple myeloma has demonstrated improved efficacy with a manageable safety profile in participants with previously treated and untreated multiple myeloma. The combination of DVRd was compared with VRd in the Phase 2 Study (GRIFFIN) demonstrating an improved depth of response and a manageable safety profile without any new safety signals in participants with ND-TEMM (Voorhees 2020). Furthermore, a randomized Phase 3 study (PERSEUS) demonstrated that the addition of daratumumab SC to VRd induction and consolidation therapy and lenalidomide maintenance therapy conferred a statistically significant and clinically meaningful benefit to PFS, the occurrence of CR or better, and MRD negativity in participants with ND-TEMM; the safety profile of daratumumab combined with VRd in the study was consistent with the known safety profiles for daratumumab and VRd (Sonneveld 2023). A large, randomized Phase 3 study (CASSIOPEIA) examined the addition of daratumumab to an established triplet combination including an IMiD and a PI (VTd). The CASSIOPEIA study demonstrated that daratumumab in combination with VTd led to a significant increase in depth of response as well as a statistically significant and clinically meaningful improvement of PFS compared to VTd alone in participants with ND-TEMM undergoing ASCT (Moreau 2019). These studies demonstrated that stem cell collection and ASCT were safe and feasible though median stem cell yield was lower and use of Plerixafor was higher with the use of daratumumab. DVRd, DVTd, and DVCd are currently recommended as treatment options for patients with ND-TEMM (Dimopoulos 2021a; NCCN 2022). Data were recently presented from a randomized Phase 3 study comparing quadruplet therapy with the anti-CD38 antibody isatuximab in combination with VRd and VRd alone prior to ASCT. These early data confirm the benefit observed with the addition of an anti-CD38 antibody to VRd with significantly higher and deeper responses observed with the combination compared to VRd alone (Goldschmidt 2022).

High-dose Chemotherapy and Stem Cell Transplantation

ASCT following HDT is the standard approach to improve and deepen response in young and fit patients. ASCT in addition to combination chemotherapy has been shown to improve CR rates and prolong median OS by approximately 12 months compared with chemotherapy without ASCT (Blade 2000; Child 2003). Two Phase 3 trials evaluating the use of upfront ASCT after triplet novel agent-based induction showed that PFS was improved in the upfront ASCT arms compared with those without upfront ASCT (Attal 2017; Cavo 2017). While consolidation therapy after ASCT is used by some investigators (commonly 2 cycles), this has not been established as standard of care (Dimopoulos 2021b) and available data on benefit are not consistent (Cavo 2020; Hari 2020).

Maintenance Therapy

Maintaining response after successful induction and HDT with transplant is an important goal in treating patients with multiple myeloma. Lenalidomide maintenance after stem cell transplant has demonstrated a significant improvement in PFS and OS compared to placebo or observation and is considered SoC (Dimopoulos 2021a; NCCN 2022). However, in the registrational studies, the median PFS on lenalidomide maintenance in young and fit myeloma patients after induction therapy, HDT, and ASCT was only 52 months and most patients will still eventually relapse

(McCarthy 2017), thus indicating a persistent high unmet need to further improve longer term outcomes.

Daratumumab maintenance therapy after induction, ASCT, and consolidation has also been shown to improve longer term outcomes. In the Phase 3 study, CASSIOPEIA, administration of daratumumab maintenance therapy (16 mg/kg IV every 8 weeks) to participants who achieved PR or better following induction, ASCT, and consolidation with either DVTd or VTd, reduced the risk of progression or death by 47% (Moreau 2021). As noted above, in the randomized Phase 3 study, PERSEUS, the addition of daratumumab SC to VRd induction and consolidation therapy and to lenalidomide maintenance therapy conferred a significant benefit in terms of PFS, occurrence of CR or better, and MRD negativity (Sonneveld 2023).

The combination of lenalidomide with daratumumab with an every 4 weeks dose schedule was demonstrated to be safe in the maintenance setting in randomized studies (GRIFFIN [Voorhees 2020] and PERSEUS [Sonneveld 2023]) and ongoing studies will provide further insights into the additional benefit of adding anti-CD38 therapy to lenalidomide as maintenance therapy after ASCT (AURIGA [Shah 2019], DRAMMATIC [Krishnan 2020], and Protocol GMMG HD7 [Goldschmidt 2022]). The combination of daratumumab and IMiDs (including lenalidomide) has shown a significant and clinically meaningful improvement of efficacy parameters in several studies in different disease settings (newly diagnosed as well as relapsed disease; PERSEUS, MAIA, POLLUX, APOLLO) with a manageable safety profile without new safety findings beyond the known safety profile of the single drugs (Dimopoulos 2016; Dimopoulos 2018; Dimopoulos 2021b; Facon 2019; Sonneveld 2023).

The availability of new targeted drugs with good tolerability provides the opportunity to introduce effective multidrug combinations in induction as well as in maintenance.

2.1.3. Bispecific Antibodies to Treat B Cell Malignancies

Several bispecific antibodies are in development for multiple myeloma with 3 drugs currently being approved as monotherapy for relapsed or refractory multiple myeloma patients in Phase 3 studies (teclistamab, talquetamab, and elranatamab). Teclistamab and talquetamab are IgG4-PAA bispecific antibodies targeting the CD3 receptor expressed on the surface of T cells (Figure 12). Teclistamab additionally targets BCMA, which is expressed on the surface of multiple myeloma B cell lineage cells, as well as late-stage B cells and plasma cells. Talquetamab additionally targets GPRC5D, which is expressed on multiple myeloma cells. Both bispecific antibodies draw T cells in close proximity to target expressing cells, leading to activation of T cells and subsequent lysis of target cells.

The combination of teclistamab or talquetamab with standard of care therapies including daratumumab and IMiDs has been evaluated in multiple studies and the safety appears comparable with the individual drug components. Importantly, frequency and severity of T cell redirector toxicities of CRS and ICANS are not worsened when combining with these agents with the exception of a potentially increased risk of higher-grade CRS when IMiDs are given concurrently with step-up dosing of the bispecific antibodies. As of December 2023, in more than

400 participants treated with the bispecific antibodies and an IMiD starting in Cycle 2 after step-up dose schedule was completed, the CRS profile was consistent with monotherapy for either antibody with 4 participants experiencing Grade 3 CRS (internal data). See Section 2.1.4.1 and Section 2.1.5.1 for summaries of clinical studies evaluating teclistamab and talquetamab, respectively. A summary of available clinical data for teclistamab in combination with talquetamab is presented in Section 2.1.5.2.

Figure 12: Schematic Overview of the Mechanisms of Action of Teclistamab and Talquetamab

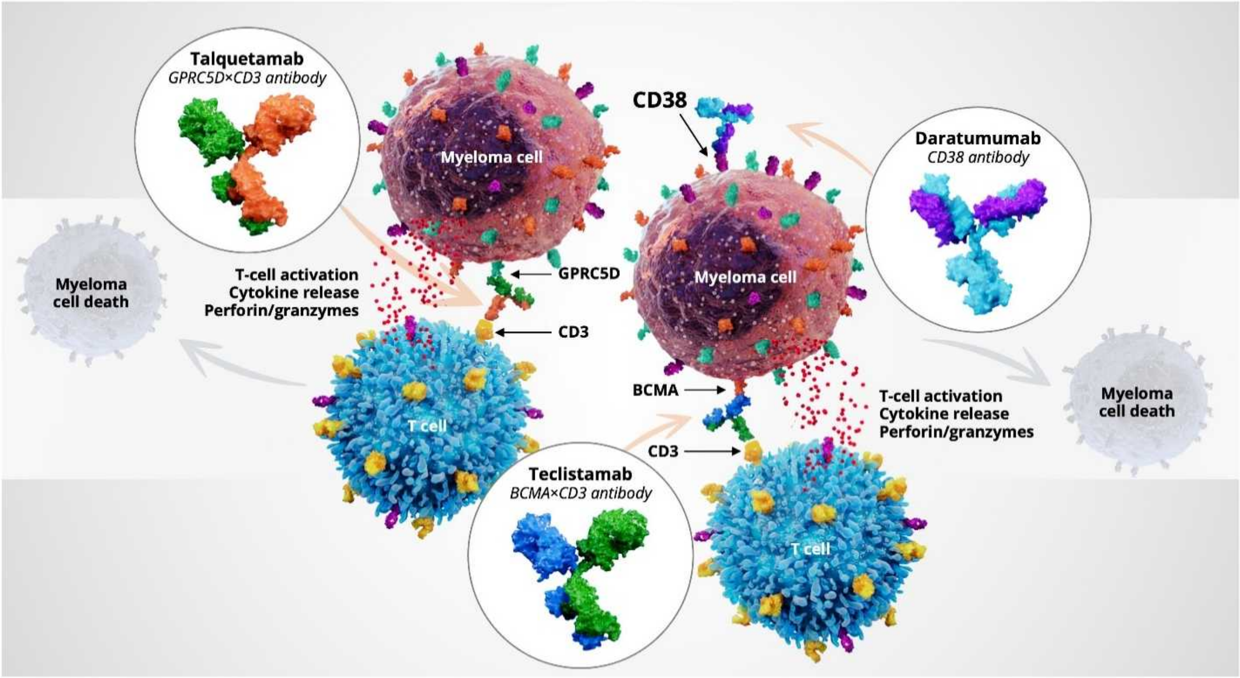

2.1.4. Teclistamab

As described in Section 2.1.4.1, teclistamab has shown promising results in pretreated, relapsed/refractory multiple myeloma patients. Teclistamab monotherapy first received marketing authorization for use in relapsed or refractory multiple myeloma populations in the European Union on 23 August 2022, was approved in the US on 25 October 2022, and has subsequently been approved in several other countries. For the most comprehensive information regarding teclistamab, refer to the latest version of the IB.

2.1.4.1. Summary of Clinical Studies

Teclistamab has already shown a manageable safety profile and promising efficacy in early phase clinical trials in participants with relapsed and refractory multiple myeloma (Usmani 2021) and is currently planned to be evaluated for safety in several early phase studies in combination with SoC drugs, including the regimen comprising teclistamab, daratumumab (D), lenalidomide (R), bortezomib (V), and dexamethasone (d) in participants with NDMM.

Ongoing studies are evaluating the safety and preliminary antimyeloma activity of teclistamab: as monotherapy (Study 64007957MMY1001 [MajesTEC-1]); in combination with daratumumab SC with and without pomalidomide (Study 64407564MMY1002 [TriMM-2]); as part of multidrug chemotherapy regimens (Study 64007957MMY1004 [MajesTEC-2]); in combination with lenalidomide and as monotherapy (Study 64007957MMY3003 [MajesTEC-4]), and in combination with daratumumab SC and lenalidomide (Study 64007957MMY3005 [MajesTEC-7]). Relevant data currently available from MajesTEC-1, TriMM-2, MajesTEC-2, MajesTEC-4, and MajesTEC-7 are described below in Section 2.1.4.1.1, Section 2.1.4.1.2, Section 2.1.4.1.3, Section 2.1.4.1.4, and Section 2.1.4.1.5, respectively. For the most comprehensive information regarding teclistamab, refer to the latest version of the IB and Addenda.

2.1.4.1.1. Study 64007957MMY1001 (MajesTEC-1)

MajesTEC-1 is an ongoing dose escalation/dose expansion Phase 1/2 study of teclistamab monotherapy in participants with relapsed or refractory multiple myeloma. This study supports the safety of teclistamab monotherapy at doses of 1.5 mg/kg weekly, [redacted], and [redacted] or Q4W.

The approved monotherapy dose is 1.5 mg/kg teclistamab SC administered weekly with the first treatment dose preceded by step-up doses of 0.06 and 0.3 mg/kg. Study results for participants treated with teclistamab at 1.5 mg/kg weekly teclistamab SC have been published (Moreau 2022). Additional information is available in the most recent teclistamab IB.

2.1.4.1.2. Study 64407564MMY1002 (TriMM-2)

TriMM-2 is an ongoing Phase 1b study of daratumumab SC regimens (1800 mg daratumumab SC, with and without pomalidomide) in combination with teclistamab or talquetamab for the treatment of participants with relapsed or refractory multiple myeloma. [redacted]

2.1.4.1.3. Study 64007957MMY1004 (MajesTEC-2)

MajesTEC-2 is an ongoing Phase 1b multi-cohort study of teclistamab with other anticancer therapies in participants with multiple myeloma. The primary objectives of this study are to characterize the safety and tolerability of teclistamab when administered in different combination regimens and to identify the optimal dose(s) of teclistamab combination regimens. Multiple myeloma regimens being evaluated in combination with teclistamab in this study include daratumumab SC and lenalidomide (Table 10).

Table 10: Overview of Relevant Study Treatment Regimens in MajesTEC-2

	Regimen B	Regimen B2	Regimen E1	Regimen E-ND
**Participants**	Relapsed/refractory multiple myeloma[a]		Relapsed/refractory multiple myeloma[b]	Newly diagnosed multiple myeloma
**Cycle duration**	C1-8: 21 days<br>C9+: 28 days		All cycles: 28 days	
**Teclistamab**	0.72 mg/kg weekly		1.5 mg/kg weekly	
**Daratumumab**	1800 mg<br>C1-2: Days 1, 8, 15<br>C3-8: Day 1<br>C9+: Day 1		1800 mg<br>C1-2: Days 1, 8, 15, 22<br>C3-6: Days 1 and 15<br>C7+: Day 1	
**Lenalidomide**	[redacted]-8: 25 mg once daily on Days 1-14 of each 21-day cycle<br><br>C9+: 10 mg once daily		[redacted]+: 25 mg once daily for 21 days of a 28-day cycle	
**Bortezomib**	1.3 mg/m^2 twice weekly<br><br>C1-8: Days 1, 4, 8, and 11	1.3 mg/m^2 weekly<br><br>C1-8: Days 1 and 8	N/A	

a. At least 1 or 2 prior lines of therapy
b. At least 1 to 3 prior lines of therapy including exposure to a PI and an IMiD.

Treatment Regimen B: 0.72 mg/kg Tec-DR and 1.3 mg/m^2 twice weekly bortezomib in Participants with Relapsed/Refractory Multiple Myeloma

As of 16 March 2023, a total of 7 participants with relapsed/refractory multiple myeloma were enrolled in Treatment Regimen B (see Table 10 for dosing schedule). The median number of prior lines of therapy was 1 and median follow-up was 15.18 months (range: 6.3 to 21.7 months). Six participants had prior exposure to PIs.

All 7 participants experienced ≥1 TEAE. One participant experienced any TEAE with maximum severity of Grade 3, 5 participants experienced any TEAE with maximum severity of Grade 4. One participant experienced a Grade 5 TEAE (COVID-19 pneumonia). Four participants discontinued teclistamab treatment due to a TEAE. Overall, 6 participants have discontinued study treatment.

One DLT was reported, Grade 3 pneumonia related to teclistamab, daratumumab, bortezomib and lenalidomide.

The most frequently reported TEAEs (at least 2 participants) were peripheral neuropathy (7 participants), CRS, diarrhea, neutropenia, and peripheral sensory neuropathy (6 participants), upper respiratory tract infection (4 participants), bone pain, constipation, fatigue, muscle spasms, nausea, pneumonia, pyrexia, thrombocytopenia, and vomiting (3 participants), and anemia, agitation, arthralgia, COVID-19, COVID-19 pneumonia, dizziness, insomnia, pneumonia, rash maculo-papular, and, viral rhinitis (2 participants).

The most frequently reported Grade 3 or 4 TEAEs (at least 2 participants) were neutropenia (6 participants) and pneumonia and thrombocytopenia (2 participants). Dose reductions in bortezomib were required as result of TEAEs in 5 participants, the most common being peripheral sensory neuropathy (4 participants). Four participants permanently discontinued bortezomib due to TEAE of peripheral sensory neuropathy.

CRS was reported in 6 participants; all events were Grade 1 (5 participants) or Grade 2 (1 participant). The median time to onset of CRS since last dose of teclistamab was 2 days (range: 1 to 5 days) and the median duration of CRS was 3.5 days (range: 1 to 12 days). Four participants received tocilizumab. All 6 participants recovered or resolved from CRS.

One participant experienced sARRs and no participants experienced ICANS, or teclistamab-related injection-site reactions of any grade.

As of 16 March 2023, there were 7 response-evaluable participants in Treatment Regimen B. ORR (PR or better) was reported in 7 participants with 4 participants achieving VGPR, 2 participants achieving CR and 1 participant achieving sCR.

Overall, safety data from Treatment Regimen B demonstrates twice weekly bortezomib was not well tolerated in a prior PI exposed population. The high efficacy rate in Treatment Regimen B supports the hypothesis that the combination of teclistamab, daratumumab, lenalidomide, and bortezomib may improve response rates and lead to better disease control, but it needs to be evaluated in the appropriate patient population. Based on the high prevalence of bortezomib-specific toxicity in this regimen, the optimal population to evaluate this combination would be in patients without prior PI exposure, such as newly diagnosed patients.

Treatment Regimen B2: 0.72 mg/kg Tec-DR and 1.3 mg/m^2 weekly bortezomib in Participants with Relapsed/Refractory Multiple Myeloma

As of 16 March 2023, a total of 9 participants with relapsed/refractory multiple myeloma were enrolled in Treatment Regimen B2 (see Table 10 for dosing schedule). Among the 9 participants, the median number of prior lines of therapy was 1 and median follow-up was 15.70 months (range: 9.6 to 17.6 months). All 9 participants had prior exposure to PIs.

All 9 participants experienced ≥1 TEAE. Three participants experienced any TEAE with maximum severity of Grade 3, 6 participants experienced any TEAE with maximum severity of Grade 4. No participants experienced a Grade 5 TEAE, and 1 participant discontinued teclistamab treatment due to a TEAE. Overall, 2 participants have discontinued study treatment. No DLTs

were reported. TEAEs reported in at least 3 participants were CRS (9 participants), fatigue (8 participants), neutropenia (7 participants), COVID-19, and diarrhea (6 participants), cough, injection site erythema, insomnia, nausea, pyrexia, and sinusitis (4 participants), abdominal pain, alanine aminotransferase increased, arthralgia, dyspnea, hypogammaglobulinemia, hypokalemia, muscle spasms, myalgia, upper respiratory tract infection, and vomiting (3 participants each).

The most frequently reported Grade 3 or 4 TEAEs (at least 2 participants) were neutropenia (7 participants) and COVID-19 (2 participants). Dose reductions in bortezomib were required as result of TEAEs in 4 participants. No participants permanently discontinued bortezomib due to a TEAE.

CRS was reported in 9 participants; all events were Grade 1 (6 participants) or Grade 2 (3 participants). The median time to onset of CRS since last dose of teclistamab was 3 days (range: 1 to 11 days) and the median duration of CRS was 2.0 days (range: 1 to 7 days). One participant received tocilizumab and 2 participants received steroids. CRS resolved in all 9 participants. No participants experienced ICANS of any grade. One participant experienced sARRs and 5 participants experienced teclistamab-related injection-site reactions.

As of 16 March 2023, there were 9 response-evaluable participants in Treatment Regimen B2. ORR (PR or better) was reported in 8 participants with 1 participant achieving VGPR. 6 participants achieving CR and 1 participant achieving sCR.

Although the Treatment Regimen B and B2 cohorts cannot be directly compared, the available data suggests that less frequent dosing of bortezomib in combination with teclistamab, daratumumab, and lenalidomide could be associated with decreased toxicity in this PI pre-exposed population and potentially support deep disease response.

Treatment Regimen E1: 1.5 mg/kg Tec-DR in Participants with Relapsed/Refractory Multiple Myeloma

A total of 19 participants with relapsed/refractory multiple myeloma were enrolled in Treatment Regimen E1 (see Table 10 for dosing schedule) as of 16 March 2023. Safety data are presented in the most recent teclistamab IB.

Treatment Regimen E-ND: 1.5 mg/kg Tec-DR in Participants with Newly Diagnosed Multiple Myeloma

A total of 11 participants with newly diagnosed multiple myeloma were enrolled in Treatment Regimen E-ND (see Table 10 for dosing schedule) as of 16 March 2023. Safety data are presented in the most recent teclistamab IB. Among the 11 participants, median follow-up was 4.70 months (range: 3.7 to 6.2 months). There were 11 response-evaluable participants. ORR (PR or better) was reported in all 11 participants with 8 participants achieving VGPR and 3 participants achieving CR.

2.1.4.1.4. Study 64007957MMY3003 (MajesTEC-4)

MajesTEC-4 is an ongoing Phase 3 randomized study of teclistamab in combination with lenalidomide (Tec-Len) and teclistamab alone versus lenalidomide alone in participants with newly diagnosed multiple myeloma as maintenance therapy following ASCT. Safety results from the Safety Run-in Phase of the study are presented in the teclistamab IB.

2.1.4.1.5. Study 64007957MMY3005 (MajesTEC-7)

MajesTEC-7 is an ongoing Phase 3 randomized study comparing teclistamab in combination with daratumumab SC and lenalidomide (Tec-DR) and talquetamab in combination with daratumumab SC and lenalidomide (Tal-DR) versus daratumumab SC, lenalidomide, and dexamethasone (DRd) in participants with newly diagnosed multiple myeloma who are either ineligible or not intended for autologous stem cell transplant as initial therapy. Results from the Safety Run-in Cohort 1 for participants treated with teclistamab (treatment dose of 1.5 mg/kg weekly in Cycles 1-2 followed by 3 mg/kg Q2W in Cycles 3-6, and 3 mg/kg Q4W in Cycle 7+) in combination with DR are presented in the teclistamab IB. The sponsor's Safety Review Committee reviewed the data (as of 10 April 2023) and assessed the safety profile as consistent with teclistamab monotherapy and that observed in the MAIA study (Facon 2019; Facon 2021), which established the benefit-risk for DRd.

2.1.5. Talquetamab

Talquetamab has received accelerated approval by the US FDA and conditional approval by the EMA for the treatment of adult patients with relapsed or refractory multiple myeloma. For the most comprehensive information regarding talquetamab, refer to the most recent version of the talquetamab IB.

2.1.5.1. Summary of Clinical Studies

Talquetamab has demonstrated significant activity with a manageable safety profile as a monotherapy in participants with relapsed or refractory multiple myeloma enrolled in the ongoing monotherapy, Phase 1/2, first-in-human, MonumenTAL-1 study (Study 64407564MMY1001).

The safety and preliminary antimyeloma activity of talquetamab is also currently being investigated as part of combination treatments (including but not limited to daratumumab, lenalidomide, and pomalidomide) in ongoing studies (Study 64407564MMY1002 [TriMM-2], and Study 64407564MMY1004 [MonumenTAL-2]). Brief descriptions of these studies are provided in Section 2.1.5.1.1 through Section 2.1.5.1.3.

Early data suggest that the combination of talquetamab with daratumumab, with or without an IMiD, is potentially effective with manageable safety profiles. CRS is a key safety concern for bispecific antibodies based on their mechanism of action. Across talquetamab studies, the majority of CRS events were Grade 1 or Grade 2, and mostly occurred during step-up dosing.

2.1.5.1.1. Study 64407564MMY1001 (MonumenTAL-1)

MonumenTAL-1 is an ongoing dose escalation/dose expansion Phase 1/2 study of talquetamab monotherapy in participants with relapsed or refractory multiple myeloma. Study results for participants treated with talquetamab IV and SC doses in the Phase 1 portion of the study have been published (Chari 2022) and key results are summarized below and in the talquetamab IB.

Two RP2Ds have been established as 0.4 mg/kg talquetamab SC weekly and 0.8 mg/kg SC Q2W with the first treatment doses preceded by step-up doses of 0.01, 0.06, and 0.3 mg/kg. The 0.4 mg/kg SC weekly dose was initially identified as the RP2D based on PK, pharmacodynamics, safety, and efficacy. At this dose level, the concentration-time profiles demonstrated a sustained pattern and maintained the exposure above the maximum EC_{90} identified in an ex vivo cytotoxicity assay. Pharmacodynamic data demonstrated consistent induction on T cell activation, cytokines, and T cell redistribution at this RP2D. To reduce the treatment burden for patients, a more convenient dose regimen of 0.8 mg/kg SC Q2W was evaluated and identified as a modified schedule of the RP2D. Both monotherapy doses are comparable in terms of efficacy, PK, and pharmacodynamics, and are both well tolerated (see talquetamab IB for a summary of MonumenTAL-1 safety data). A maximum tolerated dose has not been reached despite escalation up to 1.2 mg/kg SC Q2W.

In MonumenTAL-1, talquetamab monotherapy at 0.8 mg/kg SC Q2W is effective in terms of overall response (71.7%) and time to first response (1.3 months) when used as a second line or beyond treatment in participants with relapsed or refractory multiple myeloma. The median DOR has not been reached.

CCI [redacted]

[redacted]

[redacted]

CCI [redacted]

[redacted]

2.1.5.1.2. Study 64407564MMY1002 (TriMM-2)

CCI [redacted]

[redacted]

[redacted]

2.1.5.1.3. Study 64407564MMY1004 (MonumenTAL-2)

MonumenTAL-2 is an ongoing Phase 1b study to evaluate the safety and tolerability of talquetamab regimens in combination with daratumumab SC and lenalidomide (Table 11). Preliminary data from these regimens demonstrate that this combination is safe with promising efficacy in participants with relapsed/refractory multiple myeloma.

CCI [redacted]

CCI [redacted]

[redacted]

[redacted]

[redacted]

CCI [redacted]

[redacted]

CCI [redacted]

[redacted]

[redacted]

[redacted]

[redacted]

CCI [redacted]

[redacted]

[redacted]

[redacted]

[redacted]

[redacted]

2.1.5.2. Teclistamab/Talquetamab Combination Studies

2.1.5.2.1. Study 64007957MMY1003 (RedirecTT-1)

RedirecTT-1 is an ongoing Phase 1/2 dose escalation and expansion study in participants with relapsed or refractory multiple myeloma. The different dose combinations of teclistamab and talquetamab evaluated in the study are summarized in Table 12. Preliminary data for the CCI [redacted] combination have shown high rates of deep and robust responses in heavily pretreated participants with a manageable toxicity profile, as summarized below.

Table 12: Overview of [redacted] Dose Combinations in RedirecTT-1

Dosing Cohorts	Treated Participants (N)	Talquetamab (mg/kg)	Teclistamab (mg/kg)	Median Duration of Follow-up (months)
Dose Level 1 (DL1)	6	0.2 mg/kg QW (0.8 mg/kg per month)	0.75 mg/kg QW (3 mg/kg per month)	34.76
Dose Level 2 (DL2)	5	0.2 mg/kg QW (0.8 mg/kg per month)	1.5 mg/kg QW (6 mg/kg per month)	33.15
Dose Level 3 (DL3)	28	0.4 mg/kg QW (1.6 mg/kg per month)	1.5 mg/kg QW (6 mg/kg per month)	28.52
Dose Level 4 (DL4)	11	0.8 mg/kg Q2W (1.6 mg/kg per month)	1.5 mg/kg Q2W (3 mg/kg per month)	25.40
Dose Level 5 (DL5)	44	0.8 mg/kg Q2W (1.6 mg/kg per month)	3.0 mg/kg Q2W (6 mg/kg per month)	14.75
Dose Level 6 (DL6)	18	0.8 mg/kg Q4W (0.8 mg/kg per month)	3.0 mg/kg Q4W (3 mg/kg per month)	3.02

Step-up doses (mg/kg): DL1: 0.01, 0.06 (Tal)/0.06, 0.3 (Tec), DL2: 0.01, 0.06 (Tal)/0.06, 0.3 (Tec), DL3: 0.01, 0.06 (Tal)/0.06, 0.3 (Tec), DL4: 0.01, 0.06, 0.3 (Tal)/0.06, 0.3 (Tec), DL5: 0.01, 0.06, 0.3 (Tal)/0.06, 0.3 (Tec), and DL6: 0.01, 0.06 (Tal)/0.06, 0.3 (Tec).

As of 04 January 2024, 112 participants have been treated with the [redacted] combination and preliminary results demonstrate a safety profile that is consistent with teclistamab and talquetamab administered as monotherapy treatments, and favorable efficacy.

All 112 participants experienced at least 1 TEAE. The most frequently reported TEAEs were CRS (88 participants [78.6%]), neutropenia (74 participants [66.1%]), anemia (58 participants [51.8%]) and pyrexia (54 participants [48.2%]).

The incidence of CRS, skin-related TEAEs, and oral toxicity was consistent with the talquetamab mechanism of action and comparable to incidence observed for talquetamab monotherapy. The majority of CRS events were low grade, manageable, and reversible. Most CRS events were Grade 1 or 2, with 2 Grade 3 events reported. Of the 88 participants with CRS, 16 participants (14.3%) had a treatment cycle delay or dose modification. No CRS event led to study treatment discontinuation. ICANS was reported in 4 participants (3.6%).

Neutropenia was manageable. Grade 3 or 4 neutropenia was reported in 68 participants (60.7%). No participant discontinued study drug due to neutropenia. Fifteen participants died due to a TEAE. Of the 15 deaths, 7 (6.3% of participants) were drug related.

Of the 106 response-evaluable participants with median duration of follow-up of 16.89 months, ORR was 82.1%, with 75.5% of participants having VGPR or better. Specifically, among DL6 participants, the ORR was 83.3% with a VGPR or better rate of 66.7%. Of the 83 participants across cohorts who achieved a response of PR or better, 95% remained in response at 6 and 9 months, compared to 82% and 76% of participants who remained in response at 6 and 9 months, respectively, with talquetamab monotherapy 0.8 mg/kg Q2W in the MonumenTAL-1 study.

[redacted].

2.2. Rationale for Bispecific Antibody-based Combination Regimens

Currently, multiple myeloma is not a curable disease. With current treatments, patients derive the greatest benefit if effective treatment is administered early in the course of the disease and the greatest efficacy is achieved when the most effective drugs with different mechanisms of action are combined. As teclistamab and talquetamab with their novel mechanisms of action have been shown to be highly effective as monotherapies, synergistic combinations that target various mechanisms to overcome drug resistance need to be examined early in the course of the disease in order to realize the full potential of these bispecific antibodies.

This Phase 2 study will assess the safety and efficacy of teclistamab or talquetamab in combination with DRd (Arms A, A1, [redacted] or DVRd (Arms B, [redacted] in participants with ND-TEMM as first-line induction therapy before HDT and ASCT followed by maintenance therapy with teclistamab [redacted] in combination with DR (Arm A) or with daratumumab SC only (Arms A, A1, B, [redacted]). Arms C and C1 will assess teclistamab in combination with DR or daratumumab SC only as maintenance therapy in participants following SoC induction with HDT and ASCT, and Arm [redacted] will assess [redacted] as maintenance therapy in participants following SoC induction with HDT and ASCT. [redacted]

2.2.1. Rationale for Bispecific Antibody Therapy Combined with Daratumumab

By targeting CD38, daratumumab exhibits antitumor effects of CCDC, ADCC, ADCP, and apoptosis, as well as immunomodulatory effects. Daratumumab, in addition to its direct cytotoxicity to myeloma cells, has been shown to increase helper and cytotoxic T cells and to deplete $CD38^+$ immunomodulatory cells (Adams 2019).

In nonclinical testing, the benefits of such immune modulation were observed from in vivo pretreatment of multiple myeloma patients with daratumumab, which either increased or did not antagonize the in vitro cell lysis of multiple myeloma cells to a bispecific antibody targeting BCMA and CD3 or GPRC5D and CD3 (Frerichs 2020; Verkleij 2021).

Therefore, daratumumab in combination with talquetamab or teclistamab may lead to enhanced clinical responses in the treatment of newly diagnosed multiple myeloma through multiple mechanisms of action. By targeting either GPRC5D or BCMA with a bispecific antibody, enhanced T cell-mediated cytotoxicity is expected through recruitment of CD3-expressing T cells to the GPRC5D- or BCMA-expressing cells. By jointly targeting different epitopes on multiple myeloma cells, effector T cells and NK cells, as well as the bone marrow microenvironment through discrete and complimentary mechanisms of action, this combination may be an effective therapeutic approach in treating multiple myeloma.

Clinical data from the Phase 1b study, TriMM-2, have shown the combinations of weight-based teclistamab SC or talquetamab SC with daratumumab SC to have a tolerable safety profile and promising early efficacy in participants with relapsed or refractory multiple myeloma (see Section 2.1.4.1.2 and Section 2.1.5.1.2). The existing data for the combinations of weight-based teclistamab or talquetamab with daratumumab SC show comparable safety profiles with that of the monotherapies. Given the high response rates observed with Tec-D and [redacted] in TriMM-2, it is anticipated that the proposed regimens for this study, with monthly 3 mg/kg teclistamab dosing [redacted] dosing from Cycle 2 in combination with daratumumab SC, is sufficient to maintain efficacy in a low tumor burden maintenance treatment setting (see Section 4.2).

2.2.2. Rationale for Bispecific Antibody Therapy Combined with Daratumumab and an IMiD

IMiDs, such as lenalidomide and pomalidomide, form the backbone of several current multiple myeloma treatment regimens. IMiDs have direct anti-proliferative and pro-apoptotic effects on myeloma, as well as indirect immunomodulatory effects on the myeloma tumor microenvironment (Bazarbachi 2019; Lu 2014). IMiDs can enhance T cell activity by increasing effector T cell proliferation, by augmenting the production of Th1 cytokines, and/or by inhibiting regulatory T cell proliferation and suppression (Quach 2010).

Several studies have suggested that IMiDs may enhance or improve the efficacy of immunotherapies. Recently, preclinical studies in models of multiple myeloma have demonstrated that lenalidomide in combination with CAR-T cells had increased antitumor activity compared with CAR-T cells alone (Wang 2018; Works 2019). IMiDs also enhance talquetamab-mediated anti-multiple myeloma activity (Verkleij 2021). Moreover, IMiDs in combination with a BCMA T cell-redirector have been reported to synergistically induce T cell-dependent cellular cytotoxicity against multiple myeloma cells and induce sustained inhibition of multiple myeloma cell growth in SCID mice reconstituted with human T cells (Cho 2020).

In agreement with these reports, lenalidomide in combination with teclistamab or talquetamab leads to enhancement of the antimyeloma activity of teclistamab or talquetamab alone and higher induction of T cell activation as determined by increased CD25 expression on T cells (internal data).

In the present study, the combinations of teclistamab or talquetamab with DRd will be evaluated as induction treatments and of teclistamab or talquetamab with daratumumab or DR as maintenance treatments. It is anticipated that these regimens will combine the individual cytotoxic and immunomodulatory aspects of each drug and lead to enhanced efficacy against multiple myeloma.

DRd was approved for use in patients with NDMM based on the MAIA study (Facon 2019; Facon 2021) in which 737 participants with NDMM who were ineligible for ASCT were randomized to receive either DRd or Rd until disease progression. At a median follow-up of 28 months, the estimated PFS at 30 months was 70.6% (95% CI: 65.0-75.4) in the DRd group and 55.6% (95%CI 49.5 to 61.3) in the Rd group (HR=0.56; 95% CI: 0.43-0.83, p<0.001; Facon 2019). After a median follow-up of almost 5 years, the estimated 5-year OS rate was 66.3% in the daratumumab group and 53.1% in the control group (HR=0.68; 95% CI: 0.53-0.86, p=0.0013; Facon 2021).

Data from the recent Phase 3 study, PERSEUS, supports daratumumab and lenalidomide as maintenance treatment, with the addition of daratumumab SC to VRd induction and consolidation therapy and to lenalidomide maintenance therapy conferring a significant and clinically meaningful benefit with respect to PFS, occurrence of CR or better, and MRD negativity in patients with ND-TEMM, with no unexpected safety concerns (Sonneveld 2023). Teclistamab in combination with lenalidomide as a maintenance treatment is being evaluated in the ongoing Phase 3 study, MajesTEC-4 (Section 2.1.4.1.4).

Based on high response rates for teclistamab monotherapy (Section 2.1.4.1.1) and talquetamab monotherapy (Section 2.1.5.1.1), and promising early response data and tolerable safety profiles observed for Tec-DR (Section 2.1.4.1.3 and Section 2.1.4.1.5) and [redacted] (Section 2.1.5.1.3) in both the relapsed/refractory and newly diagnosed settings, it is hypothesized that teclistamab or talquetamab in combination with DRd, DR, or daratumumab alone will be tolerable and may offer new regimens with potential synergy to elicit deeper responses in the newly diagnosed setting.

2.2.3. Rationale for Bispecific Antibody Therapy Combined with Daratumumab, an IMiD, and Bortezomib

Bortezomib is a PI and a reversible inhibitor of the chymotrypsin-like activity of the 26S proteasome in mammalian cells (VELCADE USPI 2019). Bortezomib-based treatment regimens have demonstrated significant improvements in response, PFS, and OS compared with non-bortezomib-based therapy for multiple myeloma, both in newly diagnosed ASCT-ineligible patients and those suitable for induction and ASCT (Dimopoulos 2009; Sonneveld 2013).

In nonclinical studies, bortezomib has been shown to enhance adaptive immune responses by enhancing cytokine secretion from $CD4^+$ T cells and activating effector function of $CD8^+$ T cells (Pellom 2017; Thounaojam 2015), which may increase the potency of teclistamab and talquetamab, leading to enhanced clinical benefit. Therefore, it is anticipated that the addition of bortezomib to the Tec-DRd and [redacted] regimens has the potential to result in response rates superior to these regimens without bortezomib. The available clinical data from MajesTEC-2 demonstrate that a regimen of Tec-DVRd is tolerated (see Section 2.1.4.1.3).

Early data from MajesTEC-2 Regimen B in pretreated participants with relapsed/refractory multiple myeloma revealed that a prior exposure to bortezomib is thought to be a critical driver for high rates of neuropathy, as well as treatment modifications (See Section 2.1.4.1.3). The Tec-DVRd and [redacted] regimens are expected to be better tolerated in participants with NDMM

who have not previously received treatment; however, if polyneuropathy is observed in this study the bortezomib schedule will be adjusted (Section 6.1). Given that teclistamab in combination with other medications has not been previously evaluated in participants with NDMM eligible for ASCT, in order to ensure participant safety, the current study was designed to first confirm the safety of the Tec-DRd regimen as first-line induction therapy prior to evaluating the Tec-DVRd regimen as first-line induction therapy.

Based on an early evaluation of safety in participants being treated with Tec-DRd induction conducted by the Study Safety Committee, which includes external experts, the Study Steering Committee decided that Tec-DRd induction was safe and tolerable and Arm B (Tec-DVRd) was opened for enrollment. Arm B will only fully enroll if an early evaluation confirms that this regimen is safe and tolerable (Figure 2 and Section 4.1). Additionally, following Amendment 4, Arms [redacted] and [redacted] may be opened to evaluate [redacted] induction; the decision to open these arms will be based on emerging data from Arms A1 and B for Tec-DRd and Tec-DVRd induction, and from Arms [redacted] and [redacted] for [redacted] induction.

2.2.4. Rationale for [redacted] for HDT and ASCT

Despite notable advances in induction/maintenance therapies, which have led to extended PFS and OS in patients with ND-TEMM, ASCT remains a recommended therapy for eligible patients. While ASCT can extend PFS, it is not considered a cure for multiple myeloma, as almost all patients will relapse or progress post-transplant. Consolidation and maintenance therapy until disease progression is recommended to prolong PFS and OS after ASCT (NCCN 2022; Dimopoulos 2021a). As such, the benefits of ASCT followed by maintenance therapy until progression must be weighed against the associated risks. Advances in research and the development of novel therapies remain crucial to improving outcomes and finding more effective and less burdensome treatments for patients with multiple myeloma.

It is hypothesized that targeting 2 distinct antigens found on multiple myeloma cells may overcome some of the common mechanisms of resistance to monotherapy treatment by reducing the risk of loss of activity due to single target antigen loss and enhancing antigen antibody interaction. Therefore, dual targeting may also mitigate target molecule related escape, potentially reducing the risk of relapse (Fernández de Larrea 2020). Based on the results of the RedirecTT-1 Phase 1/2 study, the combination of [redacted] has demonstrated high rates of deep and robust responses in heavily pretreated participants with a manageable toxicity profile (see Section 2.1.5.2.1). As both [redacted] rely on the immune system of the patient, receiving this combination at earlier lines of therapy could lead to improved antimyeloma effects. [redacted]

[redacted]

CCI [redacted]

2.3. Background

Nonclinical Studies

Complete details of the in vitro and in vivo pharmacology, safety pharmacology, toxicology, and local tolerance of teclistamab and talquetamab are presented in the most recent versions of the respective IBs.

Teclistamab and Talquetamab Clinical Studies

Ongoing clinical studies of the use of teclistamab and talquetamab in participants with multiple myeloma are described in Section 2.1.4.1 and Section 2.1.5.1. Additional information is available in the most recent IBs.

2.4. Benefit-Risk Assessment

An overview of the risks associated with the study drugs is presented in the following sections. More detailed information about the known and expected risks may be found in the IBs for teclistamab and talquetamab, and prescribing information for daratumumab; bortezomib; lenalidomide; and dexamethasone. Management guidelines for potential toxicities are presented in Section 6.5.

2.4.1. Risks for Study Participation

The risks for study participation are presented by study treatment component in the sections below. The risks for a given participant in the study are a combination of the risks described for each of the components of the study treatments in the arm to which they are assigned, as well as any potential overlapping toxicities for a given combination.

2.4.1.1. Risks Associated with Teclistamab and Talquetamab

Given that teclistamab and talquetamab act by stimulating the endogenous immune system, there is potential for toxicity on other tissues or organs by activating immune cells through a potential inflammatory mechanism.

The potential and identified risks for teclistamab and talquetamab detailed in Table 13 are based on: 1) results of nonclinical studies; 2) presumed mechanism of action; 3) route of administration; and 4) results from participants treated in teclistamab (Section 2.1.4.1) and talquetamab (Section 2.1.5.1) studies.

Table 13: Mitigation Strategies for Potential Risks Associated with Teclistamab and Talquetamab

	Mitigation Strategies
CRS	Step-up dosing reduces frequency and severity of CRS. Administer pretreatment medications for teclistamab per Table 23, Table 27, Table 29 and Table 33 and for talquetamab per Table 25, Table 27, Table 31 and Table 35; see also Section 6.2.1. Follow guidance for management of CRS (Section 6.5.1.1) and instructions for dosing interruption (Section 6.9.4 for non-hematologic AEs) and treatment discontinuation (Section 7.1) as applicable. CRS occurs most commonly after administration of the first 3 doses of teclistamab or talquetamab. **Note:** CRS may occur upon re-initiation of teclistamab or talquetamab after a prolonged dosing interruption.
ICANS and other neurotoxicity	Early recognition of ICANS is critical to management. Participants should have close follow-up with the clinic and be advised to seek medical evaluation if they notice impairment in motor function (eg, weakness), changes in sensation (eg, numbness), or symptoms suggestive of possible CNS abnormalities such as new onset of headache or mental status changes. Suspected ICANS will be evaluated using the ICE Tool (see Appendix 15) with ICANS grading and recommended management per ASTCT (see Section 6.5.2.1). Follow guidance for management of ICANS (Section 6.5.2.1) and instructions for dosing interruption (Section 6.9.4 for non-hematologic AEs) and treatment discontinuation (Section 7.1) as applicable.<br><br>CCI [redacted].
sARRs	Administer pretreatment medications for teclistamab per Table 23, Table 27, Table 29 and Table 33, and for talquetamab per Table 25, Table 27, Table 31 and Table 35; see also Section 6.2.1. Follow guidance for management of sARRs in Table 41 (Section 6.5.5) and instructions for dosing interruption (Section 6.9.4 for non-hematologic AEs), and treatment discontinuation (Section 7.1) as applicable.
Cytopenias	Frequently monitor hematologic parameters and provide supportive care (eg, transfusions, growth factors) per institutional standards. Prolonged neutropenia may increase the risk of infection. Severe thrombocytopenia may increase the risk of bleeding. See Section 6.12.1 regarding use of permitted therapies for treatment of cytopenias.
Hypogammaglobulinemia	Monitor immunoglobulin levels after treatment and treat according to local guidelines, including administration of immunoglobulin replacement (see Section 6.5.3). Monitor for infection.<br>Additionally, immune response to vaccines may be reduced.<br>Vaccination with live virus vaccines is not permitted for at least 4 weeks prior to start of treatment, during treatment, and for the 30 days following the last dose of teclistamab or talquetamab.

Table 13: Mitigation Strategies for Potential Risks Associated with Teclistamab and Talquetamab

	Mitigation Strategies
Infection	See Section 6.5.4 for detailed guidance.<br>Frequently monitor for the presence of infections, with the acquisition of cultures and/or implementation of empiric antibiotic therapy as appropriate, based on clinical judgment and institutional standards. Note that rates of high-grade infections have occurred with greater frequency using BCMA targeted therapy.<br>Perform screening for HBV, HCV (see Section 8.3.5), and HIV (see Appendix 18), monitor as clinically indicated. Initiate prophylaxis or treatment therapies for infections as appropriate (Section 6.5.4.1), including prophylactic administration of antibiotics and Pneumocystis carinii/jirovecii pneumonia prophylaxis as described in Section 6.12.2.1.<br>For participants with history of HBV infection, monitor for clinical and laboratory signs of HBV reactivation and for at least 6 months after the last dose of any study treatment (Section 8.3.5.3). Withhold treatment if reactivation diagnosed and initiate treatment as appropriate (Section 6.5.4.5).<br>Appendix 19 includes specific guidance regarding study conduct during the COVID-19 pandemic.<br>PML, which can be fatal, has also been reported in patients receiving teclistamab. Monitor any new onset of or changes in pre-existing neurological signs or symptoms. If PML is suspected, withhold treatment with teclistamab and initiate appropriate diagnostic testing. Discontinue teclistamab if PML is confirmed.
Injection-site reaction	Monitor for local reactions surrounding the injection site and manage per institutional standards (see Section 6.5.6).
TLS	Frequently monitor chemistry parameters. Early recognition of signs and symptoms. Initiation of preventive measures in high-risk participants prior to treatment, as well as the prompt initiation of supportive care for participants who develop acute TLS during treatment per institutional standards (see Section 6.5.7).
Immune-related adverse event	Monitor closely for possible development of irAE(s) and treat per institutional standards (Section 6.5.8). May require dosing interruption or lead to treatment discontinuation.
Skin and nail changes (talquetamab only)	Skin and nail changes may occur. Skin changes may include skin peeling, itching, and rash, and nail changes may include nail loss, peeling, ridging, discoloration, breaking and separation. Early use of emollients is encouraged to mitigate skin changes. Rashes may be local, at the site of a subcutaneous injection, or may be systemic. Monitoring rash progression is critical and early intervention may be necessary. Rashes occurring during the first cycle should be treated with topical steroids and early consideration of a short course of oral steroids to reduce the risk of rash progression (see Section 6.5.9). Symptomatic treatment is recommended for nail dysfunction (see Section 6.5.10). A dermatologist may be consulted.
Oral toxicity (talquetamab only)	Oral side effects may occur, and may include dry mouth, altered taste, loss of taste, and difficulty swallowing. Administer supportive care, based on best clinical judgment and institutional standards, such as saliva-stimulating agents, steroid mouth wash, or consultation with a nutritionist (see Section 6.5.11). Over time, notable weight loss may occur. Weight change should be monitored regularly during therapy. Clinically significant weight loss should be further evaluated.

2.4.1.2. Risks Associated with Daratumumab SC

The primary acute toxicity for SC daratumumab is sARRs that occur mainly within 6 hours after the first injection. Although the majority of these are predominantly Grade 1 and Grade 2, severe sARRs which could be considered potential aggravating risk factors for cardiac events have occurred. Details regarding the management of sARRs are presented in Section 6.5.5.

Daratumumab may increase neutropenia and thrombocytopenia induced by background therapy. Neutropenia and hypogammaglobulinemia associated with daratumumab may contribute to the risk of infection. A higher incidence of Grade 3 or 4 neutropenia has been observed in participants with lower body weight who receive SC daratumumab. Increased incidence of serious infections, most notably pneumonia, in association with daratumumab have been reported.

Daratumumab binds to CD38 found at low levels on RBCs; it may interfere with blood typing and result in a positive Indirect Antiglobulin Test (see Section 5.3.1).

See Section 8.2.1.1 regarding the potential for daratumumab to interfere with disease evaluations.

For the most comprehensive information regarding daratumumab SC, refer to the latest version of the prescribing information.

2.4.1.3. Risks Associated with Lenalidomide

The most commonly reported adverse reactions associated with IMiDs (eg, lenalidomide and pomalidomide) in pivotal studies have been hematologic toxicities (eg, anemia, neutropenia, and thrombocytopenia). Respiratory infections occur predominately during the first treatment cycles (Delforge 2017). Thrombosis and embolism are rare but serious side effects of IMiDs. Prevention of deep vein thrombosis and pulmonary embolism is discussed in Section 6.12.2.5.

Lenalidomide in an analog of thalidomide, which is a known human teratogen that causes severe birth defects or embryo-fetal death. Participants must be willing to adhere to the pregnancy prevention guidelines as described in Inclusion Criteria, Section 5.1, and Appendix 9. Participants of childbearing potential must undergo pregnancy testing as described in the relevant Schedule of Activities and the applicable IMiD local PPP program.

Refer to the most recent labeling for lenalidomide for current information regarding risks.

2.4.1.4. Risks Associated with Bortezomib

Peripheral neuropathy is one of the most frequently reported AEs with bortezomib, although the incidence of Grade >2 peripheral neuropathy is lower with SC administration (24% and 39% with SC and IV, respectively). Weekly administration of bortezomib demonstrated to be better tolerated, including lower rates of neuropathy, and having comparable activity (Sidana 2017). Management of neurologic toxicity associated with bortezomib administration is discussed in Section 6.9.4.3. Cytopenias associated with the administration of bortezomib have potentially overlapping toxicity with administration of teclistamab.

Refer to the most recent labeling for bortezomib for current information regarding risks.

2.4.1.5. Risks of Overlapping Toxicities

2.4.1.5.1. General

With the exception of teclistamab and talquetamab, the other agents comprising the treatment regimen combinations are commonly coadministered in clinical practice. The available data from MajesTEC-2 (see Section 2.1.4.1.3), TriMM-2 (see Section 2.1.4.1.2 and Section 2.1.5.1.2), MasjesTEC-4 (see Section 2.1.4.1.4), MajesTEC-7 (see Section 2.1.4.1.5), and MonumenTAL-2 (see Section 2.1.5.1.3) support a manageable safety profile for teclistamab or talquetamab used in combination with daratumumab and/or lenalidomide and also suggest that teclistamab in combination with daratumumab, bortezomib, and lenalidomide is tolerated. In the Phase 2 GRIFFIN study and Phase 3 PERSEUS study, the addition of daratumumab to VRd was well tolerated and effective in participants with ND-TEMM (Sonneveld 2023;Voorhees 2020).

Appropriate preventive measures and guidelines for managing potential toxicities will be instituted, as detailed in the protocol (Section 6.4 and Section 6.5) and the applicable IBs, prescribing information, and the lenalidomide local PPP program (see Appendix 9). Serum drug concentrations, anti-drug antibodies, and safety will be monitored throughout the study. Pharmacokinetic-based drug-drug interactions resulting in clinically significant changes to exposure are not anticipated based on the nature of metabolism for protein therapeutics. In addition, in order to ensure participant safety, the current study first confirmed the safety of the Tec-DRd regimen as first-line induction therapy prior to evaluating the Tec-DVRd regimen as first-line induction therapy (Section 4.1.1).

2.4.1.5.2. Risk of Overlapping Toxicities for CCI [redacted]

Given the binding to CD3 regions on T cells and to antigens found primarily on multiple myeloma cells by both study drugs, there is concern for overlapping toxicity profiles. These include CRS, ICANS, infection, and hypogammaglobinemia, due to activation of a broad array of immune cells that secrete inflammatory cytokines while targeting B cells. Preliminary data from CCI [redacted] demonstrate that the TEAEs observed were manageable and consistent with those observed in the monotherapy studies (Moreau 2022; Minnema 2022). CCI [redacted]

The current study has been designed to address possible overlapping toxicities as discussed above and includes mitigation strategies (see Section 2.4.3). All safety data will be reviewed periodically to reduce the risk of toxicity to participants enrolled on the study.

2.4.2. Benefits for Study Participation

As described in Section 2.1.1, the patient population to be evaluated in the current study has unmet need. Efficacy results from studies of teclistamab and talquetamab monotherapy and from combination studies of the bispecific antibodies with daratumumab SC and daratumumab plus an

IMiD suggest that each of these bispecific antibodies may have synergistic antimyeloma effects with these established therapies. Achieving the deepest and most sustained depth of response is a critical step to control multiple myeloma and subsequently results in the longest possible PFS and reduction of disease-associated morbidities and mortality. The addition of teclistamab or talquetamab to daratumumab SC, or to the well-established combination regimen of daratumumab and lenalidomide (with or without bortezomib), may augment responses, including MRD negativity, and lead to improved PFS and OS. Additionally, the combination of CCI [redacted] has demonstrated high rates of deep and robust responses in heavily pretreated patients with relapsed/refractory multiple myeloma, and as such is hypothesized to be an effective alternative to post-induction HDT+ASCT in patients with ND-TEMM.

2.4.2.1. Benefits for Teclistamab or Talquetamab with Daratumumab, Lenalidomide, and Dexamethasone, with or without Bortezomib, as Induction

Overall survival in multiple myeloma has improved in recent years with the introduction of IMiDs and PI-based induction regimens followed by ASCT (Usmani 2018). With the emergence and success of immunotherapy, there is an opportunity to challenge the established cytotoxic drug regimens and push for novel agents that are less toxic for patients. Furthermore, many patients who progress on initial therapy are not eligible to receive second-line therapy. Recent reports suggest that attrition rates between the first and second line of therapy are as high as 50% in patients with multiple myeloma (Fonseca 2020; Venner 2015; Yong 2016), which emphasizes the need to provide the most active therapies in the first regimen. The addition of teclistamab or talquetamab to the well-established combination regimen of daratumumab, lenalidomide, with or without bortezomib may augment responses, including MRD negativity, and lead to improved PFS and OS.

2.4.2.2. Benefits for Teclistamab or Talquetamab with Daratumumab, with or without Lenalidomide, as Maintenance

The combination of daratumumab and lenalidomide has previously demonstrated a good efficacy and safety profile (Dimopoulos 2016; Facon 2019; Sonneveld 2023; Voorhees 2020). The addition of teclistamab or talquetamab to daratumumab and lenalidomide may improve antimyeloma activity by adding a new mechanism of action to an established regimen and lead to improved disease control and better long-term outcomes in a broader population (see Section 2.2). Efficacy results from studies of teclistamab and talquetamab monotherapy and from combination studies of the bispecific antibodies with daratumumab SC and daratumumab plus an IMiD suggest that each of these bispecific antibodies may have synergistic antimyeloma effects with these established therapies (Section 2.2.1 and Section 2.2.2).

2.4.2.3. CCI [redacted]

[redacted]

[redacted]

2.4.3. Benefit-Risk Assessment for Study Participation

Teclistamab- or talquetamab-based combination regimens with DRd, DVRd, DR, and daratumumab SC offer a unique mechanism of action of T cell-redirection that could lead to synergistic antimyeloma effects that potentially augment responses, including MRD negativity, and improve PFS and OS in patients with ND-TEMM (see Section 2.2). In addition, based on high rates of deep and robust response in heavily pretreated patients with multiple myeloma, the combination [redacted] a transplant-sparing treatment strategy that may provide deep and durable responses in patients with ND-TEMM.

There is potential risk for overlapping toxicities with the planned study drugs, specifically the unknown effect of IMiDs and daratumumab on CRS (which is the main toxicity of concern with teclistamab and talquetamab). For the [redacted] combination, given the binding to CD3 regions on T cells and to antigens found primarily on malignant myeloma cells by both study drugs, there is potential for overlapping toxicity profiles. These include CRS, ICANS, infection, and hypogammaglobulinemia, due to activation of a broad array of immune cells that secrete inflammatory cytokines while targeting B cells.

The risk-mitigation measures planned for this study include:

- Implementation of step-up doses of teclistamab and talquetamab to reduce risk or severity of CRS
  - Note that teclistamab and talquetamab have demonstrated mostly low-grade CRS in studies to date (Section 2.1.4.1).
- Staggered initiation of daratumumab SC and teclistamab or talquetamab therapy to reduce the risk of overlapping toxicity (Section 6.1).
- Initiation of lenalidomide administration on Cycle 2 Day 1, outside the window of highest risk for CRS with teclistamab or talquetamab (Section 6.5.1). This is also consistent with data (as of December 2023) from more than 400 participants showing that administration of an IMiD starting after step-up dosing of teclistamab or talquetamab results in a CRS profile similar to monotherapy of the bispecific antibody.

- Implementation of pretreatment medications to reduce risk or severity of sARRs and CRS (see Section 6.2).
  - Participants who experience prespecified events of sARRs and CRS will receive pretreatment medications prior to additional administration(s) of study drug (see Section 6.2.1).
- Provision of specific monitoring guidelines for participants during the first few doses of teclistamab or talquetamab when CRS risk is highest (Section 6.4).
- ICANS is a potential risk of T-cell redirector therapy, often observed concurrently with or following CRS. The above risk-mitigation measures for CRS will also address risk of ICANS associated with teclistamab or talquetamab.
- SC administration of daratumumab reduces the risk of high-grade sARRs (Mateos 2020).
  - Note that sARRs have been observed at a low frequency and low grade to date with participants treated with teclistamab or talquetamab in studies.
- Specification of recommended therapies, including antimicrobial prophylaxis and immunoglobulin to reduce risk of infection (Section 6.12.2.1).
- Robust management strategies for potential toxicities (Section 6.5):
  - CRS (Section 6.5.1.1), including provision of specific monitoring guidelines for participants during the first few doses of teclistamab or talquetamab when CRS risk is highest (Section 6.4).
  - ICANS (Section 6.5.2.1), which is often observed concurrently with or following CRS. The above risk mitigation measures for CRS will also address the risk of ICANS associated with teclistamab or talquetamab.
  - sARRs (Section 6.5.5)
  - Infection, including guidance regarding infection prophylaxis and management and specification of recommended therapies, including antimicrobial prophylaxis to reduce risk of infection (Section 6.5.4).
  - Rash (Section 6.5.9), nail dysfunction (Section 6.5.10), and oral toxicity (Section 6.5.11) in participants treated with talquetamab.

Additionally, drug combinations serving as the backbone regimens to which teclistamab or talquetamab are being added have been previously demonstrated to be safe in multiple clinical studies (see Section 2.2.1 and Section 2.2.2).

Taking into account the measures taken to minimize risk to participants in this study, including the early evaluation of safety of Tec-DRd induction treatment followed by the early evaluation of safety of Tec-DVRd induction treatment prior to expanding enrollment of these arms, the potential risks identified for Tec-DRd, Tec-DVRd, Tec-DR, Tec-D, [redacted] [redacted] [redacted] are justified by the anticipated benefits that may be afforded to participants with ND-TEMM, an incurable disease (see Section 2.1.1).

3. OBJECTIVES AND ENDPOINTS

Objectives and Endpoints for:

- Arm A (Tec-DRd induction, SoC HDT+ASCT, followed by Tec-DR or Tec-D maintenance),
- Arm A1 (Tec-DRd induction, SoC HDT+ASCT, followed by Tec-D maintenance),
- Arm B (Tec-DVRd induction, SoC HDT+ASCT, followed by Tec-D maintenance),
- [redacted]

Objectives	Endpoints
**Primary**	
• To evaluate the safety and tolerability of teclistamab- and talquetamab-based combination regimens over the entire treatment phase for each arm, in participants with ND-TEMM	• Incidence and severity of AEs and SAEs
**Secondary**	
• To evaluate MRD negativity rates, conversion, and sustainability	• Post-induction, post-ASCT (Arms A, A1, B, [redacted] and best overall<br>- MRD negative CR<br>• Sustained MRD negative CR (duration ≥12 months)<br>• MRD negative CR conversion and deepening during maintenance
• To evaluate the efficacy of teclistamab- and talquetamab-based combination regimens as induction and post-transplant maintenance treatments, and [redacted] for HDT+ASCT following induction	• Post-induction, post-ASCT (Arms A, A1, B, [redacted], post-maintenance (Arms A, A1, B[redacted] and, if applicable, [redacted] and best overall:<br>- ORR (at least a PR or better)<br>- CR or better<br>- VGPR or better<br>• DOR<br>• PFS

Objectives	Endpoints
• To assess feasibility of successful transplantation (all arms except [redacted]	• Stem cell yield and days to engraftment (all arms except [redacted]
• To characterize the PK of teclistamab and talquetamab in participants with multiple myeloma before and after an ASCT or during [redacted] treatment, and to characterize the PK of daratumumab after an ASCT	• PK parameters using population PK approach
• To assess the immunogenicity of teclistamab, talquetamab, and daratumumab	• Presence of ADAs to teclistamab, talquetamab, and daratumumab
**Exploratory**	
• To explore pharmacodynamic biomarkers of antimyeloma and immune activity of Tec-DRd, Tec-DVRd, [redacted] and (if applicable) [redacted] during induction, and of [redacted] during treatment following Tec-DRd induction<br>• To explore the relationship between PK, pharmacodynamic, AE profile, and clinical activity of Tec-DRd, Tec-DVRd, [redacted] and (if applicable) [redacted] during induction, and of [redacted] during treatment following Tec-DRd induction<br>• To explore the relationship between MRD negativity and clinical activity, including duration and depth of response of Tec-DRd, Tec-DVRd, [redacted], and (if applicable) [redacted] during induction, and of [redacted] during treatment following Tec-DRd induction<br>• To explore predictive biomarkers of response and resistance, including prognostic and disease markers at baseline, during treatment, and in relation to efficacy parameters<br>• To determine the percentage of participants with clonal plasma cell-negative autograft after 3 cycles of induction with Tec-DRd, Tec-DVRd, [redacted], and (if applicable) [redacted]<br>• To assess participants' symptoms, functioning, and HRQoL using the E[redacted] and [redacted] PROs; time to worsening of symptoms (Arms [redacted], and, if opened, [redacted] and [redacted])<br>• To explore taste-related symptoms associated with talquetamab using the [redacted]	

Arms C, C1, and [redacted] (SoC induction and HDT and ASCT followed by Tec-DR, Tec-D, or [redacted] maintenance)

Objectives	Endpoints
**Primary**	
• To evaluate the safety and tolerability of teclistamab- and talquetamab-based combination regimens over the entire treatment phase for each arm, in participants with ND-TEMM	• Incidence and severity of AEs and SAEs
**Secondary**	
• To evaluate MRD negativity rates, conversion, and sustainability	• Post-maintenance and best overall<br>- MRD negative CR<br>• Sustained MRD negative CR (duration ≥12 months)<br>• MRD negative CR conversion and deepening during maintenance

Objectives	Endpoints
• To evaluate the efficacy of Tec-DR, Tec-D, and [redacted] in post-transplant maintenance therapy	• Post- maintenance, and best overall:<br>- CR or better<br>• DOR<br>• PFS
• To characterize the PK of teclistamab, talquetamab, and daratumumab in participants with multiple myeloma who have undergone an ASCT	• PK parameters using population PK approach
• To assess the immunogenicity of teclistamab, talquetamab, and daratumumab	• Presence of ADAs to teclistamab, talquetamab, and daratumumab
**Exploratory**	
• To explore pharmacodynamic biomarkers of antimyeloma and immune activity of Tec-DR, Tec-D, and [redacted] maintenance therapy.<br>• To explore the relationship between PK, pharmacodynamic, AE profile and clinical activity of Tec-DR, Tec-D, and [redacted] during maintenance therapy.<br>• To explore the relationship between MRD negativity and clinical activity, including duration and depth of response of Tec-DR, Tec-D, and [redacted] during maintenance therapy.<br>• To explore predictive biomarkers of response and resistance, including prognostic and disease markers at baseline, during treatment, and in relation to efficacy parameters with Tec-DR, Tec-D, and [redacted] maintenance therapy.<br>• To explore taste-related symptoms associated with talquetamab using the [redacted] (Arm [redacted])	

Refer to Section 8, Study Assessments and Procedures for evaluations related to endpoints.

HYPOTHESIS

The clinical hypothesis of this study is that teclistamab- and talquetamab-based combination regimens are safe and well tolerated treatments in participants with ND-TEMM.

4. STUDY DESIGN

4.1. Overall Design

This is an open-label multicenter study in participants with ND-TEMM.

The study will be conducted in 3 phases: Screening (28 days), Treatment, and Follow-up. For Arms A, A1, B, E, [redacted] the Treatment Phase includes induction treatment and maintenance treatment. For Arm [redacted], the Treatment Phase includes induction treatment and treatment with [redacted] as replacement for HDT+ASCT. For Arms C, C1, and [redacted], the Treatment Phase includes maintenance treatment (Figure 1).

During the Screening Phase, all participants will provide written consent for study participation and will be screened for study eligibility within 28 days. All eligibility criteria must be met prior to enrollment and eligibility should be confirmed at the time of first dose of study treatment. The sponsor will strive to enroll a participant population that adequately represents the multiple myeloma population generally (Gormley 2021).

Given the limited data examining teclistamab or talquetamab in combination with other medications in participants with NDMM eligible for ASCT, the study will include early safety evaluations (Figure 2 and Section 4.1.1). To facilitate these evaluations, a Study Safety Committee which includes external experts, and a Study Steering Committee will be commissioned for the study. Refer to Committee Structure in Appendix 2.

Treatment Phase

Arms A, A1, B, D, E, E1, F, and F1

After Screening, the Treatment Phase begins with the administration of study treatment (induction with Tec-DRd [Arms A, A1, and D], Tec-DVRd [Arm B], or [redacted] [Arms [redacted] and [redacted]]) and continues until the completion of the EOT Visit. Based on emerging data from Arms A1, B, [redacted] and [redacted], the sponsor may also open Arms [redacted] and [redacted] where participants receive [redacted] induction. Periodic safety evaluations will be conducted to ensure that treatment is safe and tolerable (Section 4.1.1).

Participants will receive six 28-day cycles of Tec-DRd, Tec-DVRd, [redacted] induction therapy followed by HDT and a single ASCT according to local SoC (Arms A, A1, B, [redacted] or until confirmed progressive disease, death, intolerable toxicity, loss to follow-up, or consent withdrawal, whichever comes first [redacted] Stem cell collection is recommended to be done after Induction Cycle 3 according to local SoC.

After ASCT, per Amendment 4, participants in Arm A initially assigned to receive Tec-DR maintenance treatment may receive Tec-D maintenance per investigator's choice (participants who have started Tec-DR may discontinue lenalidomide to receive Tec-D per investigator's choice). Participants in Arms A1, B, [redacted] will receive study maintenance treatment with Tec-D, and participants in Arms [redacted] and (if opened) [redacted] will receive study maintenance treatment with [redacted]. Study maintenance treatment will be for a maximum of 18 cycles or until confirmed progressive disease, death, intolerable toxicity, loss to follow-up, or consent withdrawal, whichever comes first. Tec-DR, Tec-D, or [redacted] study maintenance treatment or [redacted] can be discontinued when 12 months of sustained MRD negativity has been observed during the study.

[redacted] treatment should be started within 28 days of induction and at least 14 days after the last dose of lenalidomide. Starting [redacted] more than 28 days after induction is only permitted after discussion and approval by the sponsor. If a participant starts [redacted] more than 28 days after induction, all End of Induction disease evaluations specified in Table 3 (except for bone marrow aspirate and imaging for MRD) must be repeated. Maintenance therapy should commence within 180 days of transplant when engraftment is complete (defined as ANC ≥1.0 x 10^9/L and platelet count ≥75 x 10^9/L) and when in the opinion of the investigator the participant is fit enough to tolerate maintenance therapy.

Arms C, C1, and [redacted]

Participants enter the Screening Phase after induction, HDT, and ASCT according to local SoC (outside of the study). The Treatment Phase begins with the administration of study maintenance treatment (per Amendment 4, participants in Arm C initially assigned to receive Tec-DR may receive Tec-D per investigator's choice, and participants in Arm [redacted] will receive [redacted]) and continues until the completion of the EOT Visit. Based on evolving data, the sponsor may also open Arm C1 where participants receive Tec-D maintenance treatment with teclistamab administered monthly as of Cycle 1. Participants will receive study maintenance treatment with Tec-DR, Tec-D, or [redacted] for a maximum of 18 cycles or until confirmed progressive disease, death, intolerable toxicity, loss to follow-up or consent withdrawal, whichever comes first. Tec-DR, Tec-D, or [redacted] study maintenance treatment can be discontinued when 12 months of sustained MRD negativity has been observed during the study. Periodic safety evaluations will be conducted to ensure that treatment is safe and tolerable.

Follow-up Phase

All Arms

Upon treatment discontinuation, an EOT Visit will be conducted. Thereafter, participants will continue in the Follow-up Phase until death, withdrawal of consent, loss to follow-up, or end of the study, whichever occurs first.

Following study maintenance therapy, additional SoC maintenance treatment per institutional standard and local investigator decision, is permitted. This additional maintenance therapy, without intercurrent progressive disease, is not considered subsequent therapy and is outside of the study. If decided to be in the best interest of the participant by the local investigator, the participant can also be observed only after the end of study treatment.

In the Follow-up Phase, participants who discontinued treatment before disease progression must continue to have disease evaluations and should not initiate any subsequent antimyeloma treatment until confirmed disease progression. After disease progression is documented, follow-up will continue and subsequent antimyeloma treatment, disease progression data (per investigator assessment) on the first line of subsequent therapy, second primary malignancies, and survival status will also be recorded.

Participants who have a response of CR or better can stop Tec-DR, Tec-D, [redacted], or [redacted] maintenance after sustained MRD negativity assessed by central labs (at or below the threshold of 10^{-5}) for 12 months, after sponsor approval. Initial MRD negativity may occur when in minimum response of VGPR, however the criteria of CR or better must be met at the time of decision to stop maintenance Tec-DR, Tec-D, [redacted] or [redacted] therapy. Qualified participants can subsequently continue standard of care maintenance treatment per institutional standard and local investigator decision.

In addition to efficacy (Section 8.2) and safety (Section 8.3 and Section 8.4) assessments, samples for PK and immunogenicity (Section 8.5) and samples for pharmacodynamics and exploratory biomarkers (Section 8.7) will be collected. All study evaluations will be conducted according to the Schedule of Activities (Section 1.3).

Diagrams of the study design are provided in Section 1.2, Schema.

4.1.1. Initiation and Expansion Rules for All Arms

Safety will be evaluated in all participants by the Study Safety Committee and based on these assessments the Study Steering Committee will make decisions regarding continuation or discontinuation of study treatment. Criteria listed below will guide decision making, however, the safety assessments performed by the Study Safety Committee and decisions by the Study Steering Committee taking into account the overall safety profile and clinical expert judgment will prevail upon continuation or discontinuation of treatment and opening of new cohorts. Safety assessment will include evaluation of non-hematological Grade 3 or 4 AEs, the overall safety profile of the combination, discontinuation of study drug, and study drug dose modifications.

- As of Amendment 4, Arm A has enrolled 10 participants. The safety profile of Arm A was evaluated after participants received at least 2 cycles of induction treatment, with successful stem cell mobilization and collection performed in at least 3 participants after Cycle 3 (see Section 8.1.1.3.1 for stem cell mobilization). There were no new safety signals in Arm A. However, based on emerging data from MajesTEC-1 suggesting a reduction in new onset of severe infections and durable responses with less frequent treatment dosing, it was decided to further optimize the teclistamab dosing schedule. Therefore, following Protocol Amendment 3 all participants in Arm A transitioned to monthly dosing of teclistamab. No further participants will be enrolled into Arm A.
- Following Amendment 3, Arms A1 and B started enrollment of approximately 20 participants each in parallel. Safety data from Arms A1 and B will be assessed after a minimum of 10 participants receive at least 3 cycles of induction treatment. Based on the safety snapshot and the totality of data, Arm A1 and/or Arm B may be expanded up to a total of 80 participants (Arms A1 and B combined).
- Following Amendment 4, Arm [redacted] will be opened to initially enroll approximately 20 participants and may enroll up to 60 participants. After Arm [redacted] has begun enrolling participants, Arms [redacted] and [redacted] will be opened to initially enroll approximately 10 participants each and may enroll up to 30 participants each. In addition, based on emerging data from Arms A1, B, [redacted], and [redacted], Arms [redacted] and [redacted] may also be opened to enroll up to 30 participants each.
- Arm C plans to enroll 10 participants. Arm C participants transitioned to teclistamab monthly dosing after the approval of Protocol Amendment 3. No further participants will be enrolled into Arm C. Safety data of Arm C was assessed after a minimum of 6 participants received at least 3 cycles of maintenance treatment. Based on evolving data, the sponsor may also open Arm C1 with 10 participants where participants receive Tec-D maintenance treatment with teclistamab administered monthly as of Cycle 1. Following Amendment 4, Arm [redacted] will start enrolling up to 30 participants.

4.1.2. Scientific Rationale for Study Design

Combination regimens have been the mainstay of cancer treatment based on the premise that the combined agents have non-overlapping toxicity and synergistic mechanisms of action. Multiple combination regimens have been established in multiple myeloma and have been successful in improving clinical outcomes. See Section 2.2 for the detailed rationales for combining teclistamab or talquetamab with daratumumab and lenalidomide with or without bortezomib, and with daratumumab alone, and for combining [redacted]

Rationale for Pharmacodynamics and Exploratory Biomarker Evaluations

Biomarker studies are aimed to help understand the clinical mechanisms of action, efficacy, and resistance of Tec-DRd, Tec-DVRd, [redacted], and Tec-DR, Tec-D, [redacted] as maintenance after ASCT in participants with ND-TEMM, to identify patient subgroups that respond differently to these treatments, and to evaluate treatment-clinical response relationships. In addition, the biomarker studies are intended to help understand the impact of Tec-DRd, Tec-DVRd, [redacted] (versus SoC) during induction on the clinical activity of Tec-DR, Tec-D, [redacted] during maintenance. Section 3 specifically addresses the intent of these studies.

4.1.3. Study-Specific Ethical Design Considerations

Potential participants will be fully informed of the risks and requirements of the study and, during the study, participants will be given any new information that may affect their decision to continue participation. They will be told that their consent to participate in the study is voluntary and may be withdrawn at any time with no reason given and without penalty or loss of benefits to which they would otherwise be entitled. Only participants who are fully able to understand the risks, benefits, and potential AEs of the study, and provide their consent voluntarily will be enrolled.

Nonclinical data and mechanism of action (Section 2.1.4 for teclistamab and Section 2.1.5 for talquetamab) as well as clinical data (Section 2.1.4.1 for teclistamab and Section 2.1.5.1 for talquetamab) suggest that the combinations proposed in this study may have synergistic antimyeloma effects. However, it is possible that these combinations, may not improve response outcomes for an individual participant. Additionally, although preliminary data suggest that overlapping toxicity will be manageable (Section 2.1.4.1.3 and Section 2.1.5.1.3), the safety of these combination regimens in patients with ND-TEMM continues to be evaluated. To ensure the well-being of participants treated in this study, the study design includes robust risk-mitigation measures (summarized in Section 2.4.3) and guidance for the management of potential toxicities (Section 6.5). Given the data from the ongoing studies for teclistamab and talquetamab, and the inclusion of well-studied, established background therapies, there is reason to believe in a positive benefit-risk profile for participation in this study (Section 2.4.3).

The blood sample collection scheme was designed to collect the minimum number of blood samples that are required to describe the PK/pharmacodynamic profile of study treatment. For Arms A, A1, B, [redacted] maximum blood volume drawn from each participant in this study during the first cycle will be approximately 230 mL, inclusive of approximately 82.5 mL that will be drawn for screening. For Arms C, C1 (if opened), and [redacted], the maximum blood volume drawn from each participant in this study during the first cycle will be approximately 202 mL, inclusive of approximately 82.5 mL that will be drawn for screening. Blood volume requirements for subsequent cycles are expected to decrease. Repeat or unscheduled samples may be taken for safety reasons or for technical issues with the samples. The estimate is considered customary and acceptable for participants in a clinical cancer study and is deemed reasonable over the timeframe of the study.

4.1.4. Rationale for Maintenance Treatment Duration

While in most studies, including the pivotal Phase 3 trials, lenalidomide maintenance was intended for use until disease progression or unacceptable toxicity, in practice the median duration of treatment varies. Prospective randomized data on the optimal duration of lenalidomide maintenance are lacking.

In the IFM2005-02 trial, the median duration of lenalidomide maintenance administration was approximately 2 years, while in the CALGB 100104 and GIMEMA RV-MM-PI-209 trials, it was 2.5 and 3 years, respectively (McCarthy 2017). In the meta-analysis by McCarthy 2017, the median duration of lenalidomide maintenance was 28 months with the following proportions of participants receiving lenalidomide for ≥ 4 years: 24.1% in CALGB, 3.6% in IFM, and 42.9 % in the GIMEMA study. Treatment discontinuation as a result of TEAEs occurred in 29% of participants in the lenalidomide arm. The most common TEAEs leading to discontinuation were neutropenia and thrombocytopenia as well as general disorders and administration site conditions not otherwise specified.

In a retrospective analysis of real-world data from 3 health centers that included 139 participants who received different induction regimens followed by an ASCT (83.5% of participants) and lenalidomide maintenance, the median time on lenalidomide maintenance was 21 months (Alonso 2020). Overall, 26 participants (18.7%) relapsed while they were receiving maintenance treatment. Based on a safety analysis of 133 participants, therapy-related AE grade >2 was observed in 34.6% of participants.

Furthermore, lenalidomide maintenance has been associated with increased risk of malignancy. In a large meta-analysis that included 3254 participants with multiple myeloma who received lenalidomide maintenance on 9 clinical trials, the cumulative incidence of secondary primary malignancies at 5 years was 6.9% in participants who received lenalidomide compared with 4.8% in those who did not (HR=1.55; 95% CI: 1.03-2.34; p=0.0037; Palumbo 2014). It is also not clear what the potential impact of prolonged lenalidomide therapy is on the effectiveness of subsequent therapy after disease progression.

Given the majority of participants discontinued lenalidomide after 2 years in pivotal and real-world studies and considering the toxicities related to long-term lenalidomide therapy, as well as the use of 2 additional potent drugs in combination with lenalidomide for maintenance in the current study, the sponsor has planned to limit the duration of the study drug maintenance treatment to a maximum of 18 months. Of note, continued maintenance therapy as per SoC is allowed. An earlier discontinuation of study maintenance treatment after 12 months of sustained MRD negativity is allowed as described in Section 4.1. This approach is supported by growing evidence that MRD has demonstrated to be a marker for long term outcomes (Munshi 2020) and MRD negativity can be used to support therapeutic decisions (Costa 2022; Derman 2022; Sonneveld 2023). A standard of care maintenance therapy can be continued according to the investigator's assessment.

4.2. Justification for Dose

4.2.1. Treatment Dose and Schedule for Teclistamab

Justification of teclistamab dose and schedule for Arm A and C prior to Protocol Amendment 3 is provided in Appendix 22.

The teclistamab SC dose schedule for induction and maintenance (see Section 4.2.3 for teclistamab SC dose schedule for CCI [redacted] treatment) will consist of 2 step-up doses (0.06 and 0.3 mg/kg) on Days 2 and 4 of Cycle 1 followed by 1.5 mg/kg on Days 8 and 15 of Cycle 1, and 3 mg/kg Q4W starting from Day 1 of Cycle 2. The PK profile following this dosing schedule is predicted to be lower than 1.5 mg/kg QW at the steady state which resembles the 0.72 mg/kg QW dose. The 3 mg/kg Q4W dosing regimen will achieve comparable PK parameters at steady state (C_{trough}, C_{max}, and AUC) to that of the 1.5 mg/kg Q2W dose, which, based on the MajesTEC-1 study, has been associated with durable responses and suggests a potential reduction in the new onset of severe infections in responders. The 3 mg/kg Q4W dosing regimen is additionally supported by emerging data from patients with relapsed/refractory multiple myeloma treated with teclistamab 0.72 mg/kg QW in combination with daratumumab and lenalidomide showing robust efficacy and a manageable safety profile (MajesTEC-2; Section 2.1.4.1.3).

4.2.2. Treatment Dose and Schedule for CCI [redacted]

CCI [redacted]

CCI [redacted].

CCI [redacted]

[redacted]

[redacted]

4.2.3. Treatment Dose and Schedule for CCI [redacted]

CCI [redacted]

[redacted]

[redacted]

CCI [redacted]

[redacted]

4.2.4. Treatment Dose and Route of Administration for Other Study Drugs

The dose schedules for daratumumab SC, bortezomib, lenalidomide, and dexamethasone are based on the standard doses used in clinical practice for the treatment of multiple myeloma, as detailed in the respective prescribing information and are supported by preliminary data from MajesTEC-2 (Section 2.1.4.1.3).

4.3. End of Study Definition

The sponsor will establish 1 or more clinical cutoff date(s) for Clinical Study Report analysis reporting, which may occur before the end of study. The clinical cutoff date(s) will be communicated to the sites. Participants who continue to receive study treatment or who are in follow-up after the final data cutoff will continue to be monitored until the end of the study. The final data from the study site will be sent to the sponsor (or designee) after completion of the final participant visit associated with the final data cutoff at that study site, in the time frame specified in the clinical trial agreement. These data will be reported to the appropriate health authorities in a final Clinical Study Report.

The end of study is defined as when all participants have completed a maximum of 18 cycles of maintenance treatment with Tec-DR, Tec-D, CCI [redacted] and have been followed for an additional 6 months or when the sponsor decides to stop the study.

5. STUDY POPULATION

Screening for eligible participants will be performed within 28 days before administration of the study treatment. Refer to Section 5.4, Screen Failures for conditions under which the repeat of any screening procedures is allowed.

The inclusion and exclusion criteria for enrolling participants in this study are described below. If there is a question about the inclusion or exclusion criteria, the investigator must consult with the appropriate sponsor representative and resolve any issues before enrolling a participant in the study. Waivers are not allowed.

Investigators should ensure that all study enrollment criteria have been met at screening. Additionally, laboratory values must be re-evaluated within 3 calendar days prior to the first dose and the participant must also meet all criteria at that time. If a participant's clinical status changes (including any available laboratory results or receipt of additional medical records) after screening but before inclusion such that the participant no longer meets all eligibility criteria, then the participant must be excluded from participation in the study. For laboratory values, if 1 or more

criteria are not met within 3 calendar days prior to dosing, 1 repeat of laboratory testing is permitted. If >1 repeat laboratory test is necessary, the sponsor must be consulted prior to dosing. Refer to Section 5.4 for conditions under which the repeat of any screening procedures is allowed. If a participant becomes ineligible between enrollment and the start of administration of study treatment, dosing must not occur, and the sponsor must be consulted. The required source documentation to support meeting the enrollment criteria are noted in Appendix 2.

5.1. Inclusion Criteria

Each potential participant must satisfy all of the following criteria to be enrolled in the study:

1. 18 to 70 years of age, inclusive

2. Have an ECOG performance status score of 0 to 2 at screening and immediately prior to the start of administration of study treatment (see Section 8.3.8 and Appendix 6).

3. Criterion modified as per Amendment 3.

   3.1. Criterion modified as per Amendment 4

   3.2 Have clinical laboratory values meeting the following criteria:

**Hematology**	
Hemoglobin	≥7.5 g/dL (≥4.65 mmol/L; without prior RBC transfusion within 7 days before the laboratory test; recombinant human erythropoietin use is permitted)
Platelets	≥75×10^9/L in participants in whom <50% of bone marrow nucleated cells are plasma cells and ≥50×10^9/L in participants in whom ≥50% of bone marrow nucleated cells are plasma cells (without transfusion support or thrombopoietin receptor agonist within 7 days before the laboratory test)
Absolute neutrophil count	≥1.0×10^9/L (prior growth factor support is permitted but must be without support for 7 days for G-CSF or GM-CSF and for 14 days for pegylated-G-CSF)
**Chemistry**	
AST and ALT	≤2.5×ULN
eGFR	≥30 mL/min based on Cockcroft-Gault formula (Appendix 7) or a 24-hour urine collection
Total bilirubin	≤2.0×ULN; (isolated total bilirubin ≥ 1.5 x ULN with conjugated [direct] bilirubin <1.5xULN is allowed for those participants with known congenital nonhemolytic hyperbilirubinemia such as Gilbert's syndrome).
Serum calcium corrected for albumin	≤14 mg/dL (≤3.5 mmol/L) or free ionized calcium ≤6.5 mg/dL (≤1.6 mmol/L; see Appendix 8)

4. Criterion modified as per Amendment 3.

   4.1. A participant of childbearing potential must have a negative highly sensitive serum pregnancy test at screening, and within 24 hours of the start of study treatment and must agree to further serum or urine pregnancy tests during the study.

5. Criterion modified as per Amendment 3.

5.1. A participant must be (as defined in Appendix 9):

a. Not of childbearing potential, or

b. Of childbearing potential and practicing 2 effective methods of contraception (at least one must be a highly effective method, see Appendix 9) from the time of signing the ICF until 6 months after the last dose of study treatment.

NOTE: Contraception must begin 4 weeks prior to dosing of lenalidomide. Reliable contraception is indicated even where there has been a history of infertility, unless due to hysterectomy, bilateral salpingectomy, or bilateral oophorectomy (Appendix 9). For participants who are of childbearing potential, see Section 6.12.3.2 for details regarding concomitant use of estrogen-containing products and lenalidomide.

NOTE: If a participant becomes of childbearing potential after start of the study the participant must comply with point (b) as described above. If a participant's reproductive status is questionable, additional evaluation should be considered.

NOTE: Sexual abstinence is considered a highly effective method only if defined as refraining from heterosexual intercourse during the entire period of risk associated with the study treatment. The reliability of sexual abstinence needs to be evaluated in relation to the duration of the study and the preferred and usual lifestyle of the participant.

6. Criterion modified as per Amendment 3.

6.1. A participant must agree not to donate eggs (ova, oocytes) or freeze for future use, for the purposes of assisted reproduction during the study and 6 months after receiving the last dose of study treatment. Participants should consider preservation of eggs prior to study treatment as anticancer treatments may impair fertility.

7. Criterion modified as per Amendment 3.

7.1. Criterion modified as per Amendment 4

7.2. A participant must wear a condom (with or without spermicidal foam/gel/film/cream/suppository) when engaging in any activity that allows for passage of ejaculate to another person during the study and for a minimum of 100 days after receiving the last dose of study treatment.

If the participant's partner is of childbearing potential, the participant must use condoms (with or without spermicide) and the partner of the participant must also be practicing a highly effective method of contraception (see Appendix 9).

NOTE: A participant who is vasectomized must still use a condom (with or without spermicidal foam/gel/film/cream/suppository), but the partner is not required to use contraception.

8. Criterion modified as per Amendment 4

8.1. A participant must agree not to donate sperm for the purpose of reproduction during the study and for a minimum of 100 days after receiving the last dose of study treatment. Participants should consider preservation of sperm prior to study treatment as anticancer treatments may impair fertility.

9. Must be willing and able to adhere to the lifestyle restrictions specified in this protocol (Section 5.3), including adherence to the global PPP or local PPP program for lenalidomide.

10. Criterion modified as per Amendment 1.

10.1. Must sign an ICF indicating that the participant understands the purpose of, and procedures required for, the study and is willing to participate in the study.

Participants in Arms A, A1, B, CCI [redacted] must also satisfy all of the following criteria to be enrolled in the study:

1A. Criterion modified as per Amendment 3.

1A.1 Documented multiple myeloma as defined by the criteria below:

a. Multiple myeloma diagnosis according to the IMWG diagnostic criteria (Appendix 4)

b. Measurable disease at screening as defined by any of the following:

1. Serum M-protein level ≥1.0 g/dL (central laboratory); or
2. Urine M-protein level ≥200 mg/24 hours (central laboratory); or
3. Serum immunoglobulin free light chain level ≥10 mg/dL (central laboratory) and abnormal serum immunoglobulin kappa lambda free light chain ratio (see Appendix 5).

NOTE: All attempts should be made to determine eligibility of the participant based on the central laboratory results of screening blood and urine M-protein measurements. In exceptional circumstances and after discussion with and approval by the sponsor, the local laboratory results of blood and urine M-protein measurements may be used to determine initial eligibility, but only if the results are ≥25% above the thresholds for measurability. In such cases, central laboratory results should still be obtained prior to the start of administration of study treatment in order to establish baseline central laboratory values and confirm the results from the local laboratory.

2A. Newly diagnosed participants for whom HDT and ASCT is part of the intended treatment plan.

Participants in Arms C, C1, and [redacted] must also satisfy all of the following criteria to be enrolled in the study:

1B. Criterion modified as per Amendment 3.

1B.1 Newly diagnosed multiple myeloma according to IMWG criteria (Appendix 4).

NOTE: Must have measurable disease at the time of diagnosis defined as measurable M-protein in the serum (≥1 g/dL) or urine (≥200 mg/24h) or serum free light chain assay (defined as ≥10 mg/dL [≥100 mg/L] on involved light chain) and abnormal serum immunoglobulin kappa lambda free light chain ratio (see Appendix 5).

2B. Must have received 4 to 6 cycles of 3 or 4 drug-induction therapy that includes a proteasome inhibitor and/or an IMiD with or without anti-CD38 monoclonal antibody and a single or tandem ASCT. Post-ASCT consolidation is permitted for up to 2 cycles as long as the total number of induction plus consolidation cycles does not exceed 6.

3B Criterion modified as per Amendment 3.

3B.1 Must have received only one line of therapy and achieved at least a PR as per IMWG 2016 response criteria based on the investigator's assessment. Participants with plasmacytomas at the time of diagnosis must meet IMWG 2016 response criteria for ≥PR based on repeat imaging utilizing the same modality (Kumar 2016).

4B Criterion modified as per Amendment 3.

4B.1 Must have received HDT and ASCT within 12 months of the start of induction therapy and be within 6 months of the last ASCT (7 months for participants who received consolidation at the time of enrollment).

5.2. Exclusion Criteria

Any potential participant who meets any of the following criteria will be excluded from participating in the study:

1. Criterion modified as per Amendment 1.

   1.1. Criterion modified as per Amendment 3.

   1.2. Excluded for any of the following:

   a. Any ongoing myelodysplastic syndrome or B-cell malignancy (other than multiple myeloma)

   b. Any history of malignancy, other than multiple myeloma, which is considered at high risk of recurrence requiring systemic therapy.

   c. Any active malignancy (ie, progressing or requiring treatment change in the last 24 months) other than multiple myeloma. The only allowed exceptions are malignancies treated within the last 24 months that are considered cured:

1) Non-muscle invasive bladder cancer (solitary Ta-PUN-LMP or low grade, <3 cm, no CIS)
2) Non-melanoma skin cancers treated with curative therapy or localized melanoma treated with curative surgical resection alone.
3) Non-invasive cervical cancer
4) Breast cancer: adequately treated lobular carcinoma in situ or ductal carcinoma in situ or history of localized breast cancer (antihormonal therapy is permitted)
5) Localized prostate cancer (M0, N0) with a Gleason Score ≤7a, treated locally only (RP/RT/focal treatment)
6) Other malignancy that is considered cured with minimal risk of recurrence in consultation with the sponsor's medical monitor.

NOTE: In the event of any questions, consult with the sponsor's medical monitor prior to enrolling a participant.

2. CNS involvement or clinical signs of meningeal involvement of multiple myeloma. If either is suspected, brain MRI and lumbar cytology are required and results must exclude CNS involvement.

3. Criterion modified as per Amendment 3.

   3.1. Stroke, transient ischemic attack or seizure within 6 months prior of Cycle 1 Day 1.

4. History of allogeneic stem cell transplant or prior organ transplant requiring immunosuppressive therapy.

5. Criterion modified as per Amendment 3.

   5.1. Any of the following:

   a. Seropositive for human immunodeficiency virus.
   b. Hepatitis B infection (ie, HBsAg or HBV-DNA positive). In the event the infection status is unclear, quantitative viral levels are necessary to determine the infection status see Section 8.3.5.1 for further required assessments.
   c. Active hepatitis C infection as measured by positive HCV-RNA testing. Participants with a history of HCV antibody positivity must undergo HCV-RNA testing. If a participant with history of chronic hepatitis C infection (defined as both HCV antibody and HCV-RNA positive) completed antiviral therapy and has undetectable HCV-RNA 12 weeks following the completion of therapy, the participant is eligible for the study.

d. COPD with a FEV1 <50% of predicted normal. Note that FEV1 testing is required for participants with known or suspected of having COPD or asthma and participants must be excluded if FEV1 <50% of predicted normal.

e. Moderate or severe persistent asthma within the past 2 years (see Appendix 11), or uncontrolled asthma of any classification. Note that FEV1 testing is required for participants with known or suspected asthma and participants must be excluded if FEV1 <50% of predicted normal.

6. Criterion modified as per Amendment 3.

6.1 Criterion modified as per Amendment 4

6.2 Concurrent medical or psychiatric condition or disease that is likely to interfere with study procedures or results, or that in the opinion of the investigator would constitute a hazard for participating in this study, such as:

a. Acute diffuse infiltrative pulmonary disease

b. Evidence of active systemic viral, fungal or bacterial infection, requiring systemic antimicrobial therapy

c. History of autoimmune disease with the exception of vitiligo, type I diabetes, and prior autoimmune thyroid disease that is currently euthyroid based on clinical symptoms and laboratory testing

d. Disabling psychiatric conditions (eg, alcohol or drug abuse), severe dementia, or altered mental status

e. Any other issue that would impair the ability of the participant to receive or tolerate the planned treatment at the investigational site, to understand informed consent or any condition for which, in the opinion of the investigator, participation would not be in the best interest of the participant (eg, compromise the well-being) or that could prevent, limit, or confound the protocol-specified assessments.

f. History of noncompliance with recommended medical treatments

7. Criterion modified as per Amendment 4.

7.1 Presence of the following cardiac conditions:

a. New York Heart Association stage III or IV congestive heart failure (Appendix 21)

b. Myocardial infarction, unstable angina, or coronary artery bypass graft ≤6 months prior to enrollment

c. History of clinically significant ventricular arrhythmia or unexplained syncope, not believed to be vasovagal in nature or due to dehydration

d. Uncontrolled cardiac arrhythmia or clinically significant ECG abnormalities.

8. Contraindications or life-threatening allergies, hypersensitivity, or intolerance to any study drug or its excipients (refer to Investigator's Brochure and appropriate package inserts).

9. Participant is pregnant, breastfeeding, or planning to become pregnant while enrolled in this study or within 6 months after the last dose of any component of the study treatment regimen.

10. Criterion modified as per Amendment 4

    10.1 Participant plans to father a child while enrolled in this study or within 100 days after the last dose of any component of the study treatment regimen.

11. Participant had significant traumatic injury or major surgery within 2 weeks prior to the start of administration of study treatment, or will not have fully recovered from surgery, or has major surgery planned during the time the participant is expected to be treated in the study or within 2 weeks after administration of the last dose of study treatment.

    NOTE: Participants with planned surgical procedures to be conducted under local anesthesia may participate. Kyphoplasty or vertebroplasty are not considered major surgery. If there is a question whether a procedure is considered a major surgery, the investigator must consult with the appropriate sponsor representative and resolve any issues before enrolling a participant in the study.

12. Received an investigational drug (including investigational vaccines; COVID vaccine released for emergency use is permitted) or used an invasive investigational medical device within 4 weeks or 5 half-lives of the respective drug/IMP (whichever is longer) before enrollment or is currently enrolled in an interventional investigational study.

13. Have gastrointestinal disease that may significantly alter the absorption of oral drugs

14. Criterion modified as per Amendment 3.

    14.1. Received a live, attenuated vaccine within 4 weeks before the first dose of study drug. Non-live or non-replicating vaccines authorized for emergency use (eg, COVID-19) by local health authorities are allowed.

15. Be unable or unwilling to undergo antithrombotic prophylactic treatment.

For Arms A, A1, B, CCI [redacted] any potential participant will also be excluded from participating in the study if they meet any of the following criteria:

1. Prior or current systemic therapy or stem cell transplant for any plasma cell dyscrasia, with the exception of emergency use of a short course (equivalent of dexamethasone 40 mg/day for a maximum 4 days) of corticosteroids before treatment.

2. Radiotherapy within 14 days or focal radiation within 7 days of enrollment. Radiotherapy on measurable soft-tissue plasmacytoma(s) is not permitted even in the setting of palliation for symptomatic management.

3. Plasmapheresis within 28 days of enrollment.

4. Criterion modified as per Amendment 3.

   4.1. Plasma cell leukemia at the time of screening, as defined by the presence of 5% or more circulating plasma cells in peripheral blood (Fernández de Larrea 2021), smoldering multiple myeloma, Waldenström's macroglobulinemia, POEMS syndrome (polyneuropathy, organomegaly, endocrinopathy, M-protein, and skin changes), or primary light chain amyloidosis.

5. Criterion modified as per Amendment 4.

   5.1. **Arms B, [redacted], and [redacted] only**: Peripheral neuropathy or neuropathic pain Grade 2 or higher, as defined by the NCI-CTCAE Version 5.

6. Criterion modified as per Amendment 4.

   6.1. **Arms B, [redacted], and [redacted] only**: Due to a potential interaction with bortezomib, received a strong CYP3A4 inducer within 5 half-lives prior to enrollment (Flockhart 2021: http://medicine.iupui.edu/flockhart/ and Appendix 20)

For Arms C, C1, and [redacted], any potential participant will also be excluded from participating in the study if they meet any of the following criteria:

1. Criterion modified as per Amendment 4.

   1.1 Received any prior BCMA-directed therapy (Arms C and C1 only) or GPRC5D-directed therapy (Arm [redacted] only).

2. Any previous therapy with an immune cell redirecting agent or gene modified adoptive cell therapy (eg, chimeric antigen receptor modified T cells, NK cells).

3. Discontinued treatment due to any AE related to lenalidomide as determined by the investigator.

4. Radiotherapy within 14 days or focal radiation within 7 days of enrollment.

5. Progressed on multiple myeloma therapy at any time prior to screening.

6. Criterion modified as per Amendment 3.

   6.1. Received a cumulative dose of corticosteroids equivalent ≥40 mg dexamethasone within the 14-day period before the start of study treatment administration (Appendix 10).

7. Criterion modified as per Amendment 3.

   7.1. Plasma cell leukemia, smoldering multiple myeloma, Waldenström's macroglobulinemia, POEMS syndrome (polyneuropathy, organomegaly, endocrinopathy, M-protein, and skin changes), or primary light chain amyloidosis.

8. Intolerant to the starting dose of lenalidomide (10 mg).

5.3. Lifestyle Considerations

Potential participants must be willing and able to adhere to the following lifestyle restrictions during the course of the study to be eligible for participation:

1. Refer to Section 6.12.3, and subsections therein for details regarding prohibited and restricted therapy during the study.

2. Agree to follow all requirements that must be met during the study as noted in the Inclusion and Exclusion Criteria (eg, contraceptive requirements) and the relevant Schedule of Activities.

3. Criterion modified as per Amendment 3

   3.1. Agree not to donate blood during therapy, during dose interruptions, and for at least 6 months after the last dose of study treatment.

4. Criterion modified as per Amendment 4

   4.1. Be willing to be hospitalized or remain in close proximity (within 30 minutes) to the hospital starting after step-up dose 1 until 48 hours after the first 3 doses of teclistamab or talquetamab as described in Section 6.4.1.

5. Criterion modified per Amendment 4

   5.1. Be willing to be hospitalized for administration of teclistamab or talquetamab following specified AEs as described in Section 6.4.1.2.

6. Due to the embryo-fetal risk associated with IMiDs, all participants must adhere to the local PPP program for lenalidomide. A participant using oral contraceptives must use an additional barrier contraceptive method (see Inclusion Criteria in Section 5.1).

7. Agree to self-monitor for signs and symptoms of CRS or infection, including fever or feeling tired, and to seek immediate medical intervention.

8. Be willing to avoid driving or operating heavy or potentially dangerous machinery starting after step-up dose 1 until 48 hours after administration of the third dose of teclistamab or talquetamab.

5.3.1. Daratumumab Interference With Indirect Antiglobulin Test Results

Daratumumab interferes with the Indirect Antiglobulin Test, which is a routine pretransfusion test performed to identify a participant's antibodies to minor antigens so that suitable donor blood can be given for transfusion. Daratumumab does not interfere with ABO/RhD typing. CD38 is expressed at very low levels on erythrocytes. Daratumumab binds to the CD38 on erythrocytes, which results in a positive Indirect Antiglobulin Test. This positive result masks the detection of antibodies to minor antigens and may prevent or delay blood banks from issuing donor blood for transfusion. This effect occurs during daratumumab treatment and for up to 6 months after treatment ends. Participants will receive a participant identification wallet card for the study that includes the blood profile (ABO, Rh, and Indirect Antiglobulin Test) determined before the first administration of daratumumab SC along with information on the Indirect Antiglobulin Test interference for healthcare providers/blood banks. Participants are to carry this card throughout the Treatment Period and for ≥6 months after treatment ends. Blood banks can eliminate the daratumumab Indirect Antiglobulin Test interference by treating reagent RBCs with dithiothreitol (Chapuy 2015).

Possible methods for blood banks to provide safe RBCs for transfusion to participants receiving daratumumab SC include:

- Providing ABO/RhD compatible, phenotypically (standard or extended phenotyping prior to daratumumab SC administration) or genotypically matched units
- Providing ABO/RhD compatible, K-negative units after ruling out or identifying alloantibodies using dithiothreitol-treated reagent RBCs.

Uncross-matched, ABO/RhD compatible RBC units should be administered if transfusion is needed emergently per local blood bank practice.

Despite daratumumab binding to CD38 on erythrocytes, no indication of clinically significant hemolysis has been observed in daratumumab studies. For additional details, refer to the daratumumab SC prescribing information.

5.4. Screen Failures

The investigator agrees to complete a participant identification and enrollment log to permit easy identification of each participant during and after the study. This document will be reviewed by the sponsor study site contact for completeness. This study will use IWRS. The investigator will not generate screening and enrollment logs directly from IWRS.

The participant identification and enrollment log will be treated as confidential and will be filed by the investigator in the study file. To ensure participant confidentiality, no copy will be made. All reports and communications relating to the study will identify participants by participant identification and age at initial informed consent. In cases where the participant is not enrolled into the study, the date seen and age at initial informed consent will be used.

Participants who fail to meet the inclusion and exclusion criteria (ie, screen failures) may be rescreened once if their condition changes. Rescreening must be discussed with and approved by the sponsor on a case-by-case basis. Participants who are determined to be eligible for rescreening must sign a new ICF and will then be assigned a new screening number.

6. STUDY TREATMENT AND CONCOMITANT THERAPY

Details of study treatment administered at the study site must be captured in the source documents and the eCRF. Teclistamab and talquetamab will be provided under the responsibility of the sponsor. The study drug labels will contain information to meet the applicable regulatory requirements. Refer to the relevant IPPI and SIPPM for additional details. Study site personnel will instruct participants on how to store study drug(s) for at home use as indicated for this protocol.

6.1. Study Treatment Administered

<table>
<tr><th>Designation</th><th>Product</th></tr>
<tr><td>Investigational Medicinal Product (IMP)</td><td>teclistamab, talquetamab, daratumumab

Authorization status in the EU/EEA:

<table><tr><td>Authorized</td><td>teclistamab, talquetamab, daratumumab</td></tr><tr><td>Unauthorized</td><td>N/A</td></tr></table>
Used in accordance with Marketing Authorization: not applicable
Used not in accordance with Marketing Authorization: teclistamab, talquetamab, daratumumab</td></tr>
<tr><td>Non-investigational Medicinal Product (NIMP)/auxiliary medicinal product (AxMP)</td><td>bortezomib, lenalidomide, dexamethasone

Authorization status in the EU/EEA:

<table><tr><td>Authorized</td><td>bortezomib, lenalidomide, dexamethasone</td></tr><tr><td>Unauthorized</td><td>N/A</td></tr></table>
Used in accordance with Marketing Authorization: not applicable
Used not in accordance with Marketing Authorization: bortezomib, lenalidomide, dexamethasone</td></tr>
</table>

An overview of administration of the study drugs is provided in:

- Table 14 and Figure 3 (Arm A, Arm A1, and [redacted]
- Table 15 and Figure 4 (Arm B),
- Table 16 and Figure 7 [redacted],
- Table 17 and Figure 5 [redacted]
- Table 18 and Figure 6 [redacted]
- Table 19 and Figure 8 (Arms A and C),
- Table 20 and Figure 9 (Arms A, A1, B, C, C1, [redacted])
- Table 21 and Figure 10 (Arm [redacted]),
- Table 22 and Figure 11 [redacted]

Additional information about administration of the study drugs is presented in the following sections. See Section 6.9 for information about dosing interruption and dose reduction.

Table 14: Study Treatment Administered – Overview – Arms A*, A1, and [redacted] (Induction)

Study Treatment	Induction Cycle (each cycle=28 days)	Dose Schedule
Teclistamab SC	Cycle 1	Step-up Dose 1 (0.06 mg/kg): Day 2<br>Step-up Dose 2 (0.3 mg/kg): Day 4<br>Treatment Dose (1.5 mg/kg): Days 8 and 15
	Cycles 2 to 6	Treatment Dose: 3 mg/kg Q4W on Day 1
Daratumumab SC (1800 mg)	Cycles 1 and 2	Weekly dose on Days 1, 8, 15 and 22
	Cycles 3 to 6	Q2W dose on Days 1 and 15
Lenalidomide PO (25 mg)	Cycle 1	No dosing
	Cycles 2 to 6	Days 1-21 from Cycle 2 onwards
Dexamethasone PO/IV (20 mg)	Cycles 1-2	Days 1-2, 8-9, 15-16 and 22-23
	Additional dexamethasone will be administered as pretreatment medication as described in Section 6.2.	

*Following Amendment 3, all participants in Arm A have transitioned to monthly dosing of 3.0 mg/kg teclistamab. Refer to Appendix 22 for dose schedule of Arm A (Table 69) with previous teclistamab weekly dosing schedule.

Table 15: Study Treatment Administered – Overview – Arm B (Induction)

Study Treatment	Induction Cycle (each cycle=28 days)	Dose Schedule
Teclistamab SC	Cycle 1	Step-up Dose 1 (0.06 mg/kg): Day 2<br>Step-up Dose 2 (0.3 mg/kg): Day 4<br>Treatment Dose (1.5 mg/kg): Days 8 and 15
	Cycles 2 to 6	Treatment Dose: 3 mg/kg Q4W on Day 1
Daratumumab SC (1800 mg)	Cycles 1 and 2	Weekly dose on Days 1, 8, 15 and 22
	Cycles 3 to 6	Q2W dose on Days 1 and 15
Bortezomib 1.3 mg/m^2 SC[a]	Cycles 1 to 6	Weekly dose on Days 1, 8, 15 and 22
Lenalidomide PO (25 mg)	Cycle 1	No dosing
	Cycles 2 to 6	Days 1-21 from Cycle 2 onwards
Dexamethasone PO/IV (20 mg)	Cycles 1-2	On Days 1-2, 8-9, 15-16 and 22-23
	Additional dexamethasone will be administered as pretreatment medication as described in Section 6.2.	

a. Based on the safety data collected in the first 10 participants in Arm B, the bortezomib regimen may be adjusted for subsequent participants (eg, delayed start of bortezomib in Cycle 2, dose reduction, or less frequent dosing).

Table 16: Study Treatment Administered – Overview – Arm [redacted] ([redacted] Treatment Following Induction)

[redacted]

Table 17: Study Treatment Administered – Overview – Arms [redacted] and [redacted] (Induction)

[redacted]

Table 18: Study Treatment Administered – Overview – Arms [redacted] and [redacted] (Induction)

[redacted]

Table 19: Study Treatment Administered – Overview – Arms A and C* (Maintenance)

Study Treatment	Maintenance Cycle [a] (each cycle=28 days)	Dose Schedule
Teclistamab SC	Cycle 1	Step-up Dose 1 (0.06 mg/kg): Day 2<br>Step-up Dose 2 (0.3 mg/kg): Day 4<br>Treatment Dose (1.5 mg/kg): Days 8 and 15
	Cycles 2 to 18	Treatment Dose: 3 mg/kg Q4W on Day 1
Daratumumab SC (1800 mg)	Cycles 1 to 18	On Day 1 of each cycle
Lenalidomide PO (10 mg)[b, c]	Cycles 2 to 18	Lenalidomide 10 mg daily on Days 1 to 28; may increase to 15 mg daily starting Cycle 5 Day 1 if tolerated

* Refer to Appendix 22 for dose schedule of Arm C (Table 70) with previous teclistamab biweekly dosing schedule.

a. Study maintenance therapy with Tec-DR to be administered for a maximum of 18 cycles. Participants who have a response of CR or better can stop Tec-DR maintenance therapy after sustained MRD negativity assessed by central labs (at or below the threshold of 10^{-5}) for 12 months (see Section 4.1).

b. Based on evolving safety data, the lenalidomide regimen may be adjusted at any timepoint by the sponsor (eg. lenalidomide dosing on Days 1-21 only).

c. Per Amendment 4, participants in Arms A and C initially assigned to receive Tec-DR maintenance may receive Tec-D maintenance per investigator's choice (participants who have started Tec-DR may discontinue lenalidomide to receive Tec-D per investigator's choice).

Table 20: Study Treatment Administered – Overview – Arms A, A1, B, C, C1, E, and [redacted] (Maintenance)

Study Treatment	Maintenance Cycle [a] (each cycle=28 days)	Dose Schedule
Teclistamab SC	Cycle 1	Step-up Dose 1 (0.06 mg/kg): Day 2<br>Step-up Dose 2 (0.3 mg/kg): Day 4<br>Treatment Dose (1.5 mg/kg): Days 8 and 15
	Cycles 2 to 18	Treatment Dose: 3 mg/kg Q4W on Day 1
Daratumumab SC (1800 mg)	Cycles 1 to 18	On Day 1 of each cycle

a. Study maintenance therapy with Tec-D to be administered for a maximum of 18 cycles. Participants who have a response of CR or better can stop Tec-D maintenance therapy after sustained MRD negativity assessed by central labs (at or below the threshold of 10-5) for 12 months (see Section 4.1).

Table 21: Study Treatment Administered – Overview – Arm [redacted] (Maintenance)

CCI [redacted]

Table 22: Study Treatment Administered – Overview – Arms [redacted] and [redacted] (Maintenance)

CCI [redacted]

6.1.1. Study Treatment Administered – Tec-DRd (Arms A, A1, or [redacted]) and Tec-DVRd (Arm B) Induction

Table 23: Dose Schedule and Pretreatment Medications for Arms A*, A1, or [redacted] (Tec-DRd), and Arm B (Tec-DVRd) Induction

Activity	Notes	Induction Treatment Phase (28-day cycle)							
**Induction Cycle**		**Cycle 1**						**Cycle 2**	**Cycles 3-6**
**Day**		**1**	**2**	**4**	**8**	**15**	**22**	**1, 8, 15, 22**	**1**
**REQUIRED PRETREATMENT MEDICATIONS: See Section 6.2.1 for event-driven required pretreatment medications. From Cycle 2 onwards, all oral pretreatment medications may be administered at home, provided they are taken within the timeframes specified below.**									
Dexamethasone 20 mg or equivalent (see Appendix 10)	• Oral/IV: administer 1-3 hours (±15 min) prior to administration of (first) study drug.<br>• On Cycle 1 Day 8 and per Table 24, an additional dose of dexamethasone (8 mg) must be given (and should be at least 15 minutes prior to teclistamab dosing) if >4 hours have elapsed since prior administration of dexamethasone (20 mg).<br>• On Days 1, 2, 8, and 15 of Cycle 1, backbone dexamethasone substitutes for premedication.	X	X	X	X	X	Dexamethasone should not be administered as pretreatment medication after Cycle 1 Day 15 except as described in Section 6.2.1 or upon discussion with the sponsor.		
Diphenhydramine 25 to 50 mg, or equivalent	• Oral/IV: administer 1-3 hours (±15 min) prior to administration of (first) study drug.<br>• On Cycle 1 Day 8 and per Section 6.2.1, a second full dose must be given prior to teclistamab if >6 hours have elapsed since prior administration.	X	X	X	X	X	X	X	X
Acetaminophen 650 to 1000 mg or equivalent		X	X	X	X	X	X	X	X
**OPTIONAL PRETREATMENT MEDICATIONS: Additional pretreatment medications such as H_2-antagonists or antiemetics may be used per investigator discretion.**									
Montelukast 10 mg	Oral: 1-3 hours (±15 min) prior to administration of daratumumab SC.	Per investigator discretion prior to administration of daratumumab SC							
**STUDY DRUGS: See Table 24 for additional detail, including the order and timing of administration of daratumumab SC and teclistamab. If they occur, CRS (fever, hypoxia, and hypotension) and ICANS must fully resolve before the next administration of teclistamab (see also criteria in Section 6.9.4.1). See Section 6.9.1.3 for information regarding acceptable windows for study drug administration.**									
Daratumumab 1800 mg SC	• There must be ≥5 days between each dose of daratumumab SC..	X			X	X	X	X	Days 1 and 15 only
Teclistamab Step-up Dose	• Administer by SC injection.<br>• Step-up Dose 1: Must be administered ≥20 hours after daratumumab SC administered on Cycle 1 Day 1.<br>• Thereafter, there must be ≥2 days between step-up doses.		0.06 mg/kg	0.3 mg/kg					
Teclistamab Treatment Dose	• Administer by SC injection.<br>• The first treatment dose of teclistamab must be administered ≥2 days after step-up dose 2.<br>• Thereafter, there must be ≥5 days between each treatment dose.				1.5 mg/kg	1.5 mg/kg		3 mg/kg Day 1 only	3 mg/kg
Bortezomib 1.3 mg/m^2 **ARM B ONLY**	• Administer by SC injection.<br>• Recalculate the dose if weight has changed ±10% from baseline.<br>• There must be ≥3 days between doses of bortezomib.	On Days 1, 8, 15 and 22 of each cycle							

Table 23: Dose Schedule and Pretreatment Medications for Arms A*, A1, or [redacted] (Tec-DRd), and Arm B (Tec-DVRd) Induction

Activity	Notes	Induction Treatment Phase (28-day cycle)							
Induction Cycle		Cycle 1						Cycle 2	Cycles 3-6
Day		1	2	4	8	15	22	1, 8, 15, 22	1
Lenalidomide 25 mg	• Oral administration<br>• Lenalidomide will not be administered during Cycle 1; it will start on Day 1 of Cycle 2<br>• Lenalidomide dose may need to be adjusted based on CrCl. See Section 6.9.4.2 and Table 56.							Days 1-21	
Dexamethasone 20 mg	• Oral/IV: administer 1-3 hours (±15 min) prior to administration of daratumumab SC. Dispense on Day 1 for self- administration	**• Cycles 1-2 on Days 1-2, 8-9, 15-16, and 22-23**<br>• Additional dexamethasone may be administered as pretreatment medication as described in Section 6.2.							

* Refer to Appendix 22 for dose schedule of Arm A (Table 71) in previous teclistamab weekly dosing schedule.

Table 24: Study Treatment Administration Instruction – Arms A, A1, [redacted] (Tec-DRd), and Arm B (Tec-DVRd) Induction

Study Drug	Teclistamab	Daratumumab SC	Bortezomib Arm B only	Lenalidomide	Dexamethasone
**Dosing Instructions**	Teclistamab treatment will start with 2 step-up doses followed by treatment doses. All doses of teclistamab follow weight-based dosing (see Table 23 for details on doses)	The volume of 1800 mg for SC injection will be approximately 15 mL and administration should occur over approximately 3 to 5 minutes.	Calculate the actual dose (mg) for administration based on the participant's BSA (m^2) (Appendix 12). If the participant's weight on dosing day has changed by +/- 10% from the first day of study treatment administration, the dose of bortezomib should be recalculated. Skipped doses of bortezomib will not be made up later in the cycle.	Lenalidomide will be supplied as 5 mg (for down titration from 10 mg), 10 mg, 15 mg, and 25 mg capsules	Dexamethasone 20 mg will be administered orally or IV 1-3 hours (±15 min) before the daratumumab or teclistamab administration during Cycle 1 only as substitution for steroid premedication. On days when daratumumab or teclistamab is not administered and in Cycle 2, dexamethasone is administered orally.
	• SC injections will be prepared as described in the appropriate IPPIs or equivalent documentation.<br>• The anatomical area of administration for each drug must be recorded in the eCRF and the area observed for injection-site reaction(s). See the IPPIs or equivalent documentation for further details.			• Lenalidomide will be self-administered orally.<br>• Lenalidomide should be taken as a single dose at the same time daily.<br>• Lenalidomide can be taken with or without food.<br>• Breaking or dividing lenalidomide capsules is strongly discouraged.<br>• Dose may need to be adjusted for participants with reduced CrCl (see Section 6.9.4.2).	Dexamethasone tablets are to be taken with or immediately after a meal or snack, preferably in the morning.

Table 24: Study Treatment Administration Instruction – Arms A, A1, [redacted] (Tec-DRd), and Arm B (Tec-DVRd) Induction

Study Drug	Teclistamab	Daratumumab SC	Bortezomib Arm B only	Lenalidomide	Dexamethasone
Order of Study Drug Administration	• When daratumumab SC and teclistamab are to be administered on the same day, daratumumab SC should be administered first.<br>• The following intervals between daratumumab SC and teclistamab should be followed:<br>o Step-up Dose 1 of teclistamab must be administered ≥20 hours after the first dose of daratumumab SC.<br>o On Induction Cycle 1 Day 8, teclistamab should be administered ≥3 hours after daratumumab SC.<br>▪ Note the requirements for repetition of the required pretreatment medications if >4 hours (steroid) or >6 hours (antihistamine, antipyretic) have elapsed since prior administration of the pretreatment medication (see Table 23).<br>o Thereafter, teclistamab should be administered at least 15 minutes after daratumumab SC.<br>o Teclistamab will not be administered during or within 2 hours after resolution of any sARR related to daratumumab SC.<br>o If a participant experiences treatment delays of daratumumab SC longer than 3 months, the interval between daratumumab SC and teclistamab should be at least 3 hours for the first daratumumab dose.<br>• Bortezomib must be administered after the daratumumab SC administration. Bortezomib dosing may be delayed up to 48 hours; in such cases, subsequent doses must be adjusted to account for the delay. Note that there should be at least 3 days between doses of bortezomib. Skipped doses of bortezomib will not be made up later in the cycle.<br>• For participants with unacceptable toxicity at the local injection site despite dose modifications or change in injection concentration, bortezomib can be administered intravenously as a 3 to 5 second bolus injection (see Section 6.5.6.1).<br>• When teclistamab, daratumumab, and bortezomib are administered on the same day, daratumumab will be given first, followed by bortezomib, and then teclistamab.<br>• When more than one component of study treatment is administered SC on the same day, each SC component should be injected at different anatomical locations. See the IPPIs or equivalent documentation for further details.<br>• For all components of study treatment that are administered SC, the anatomical area of administration must be recorded in the eCRF and the area observed for injection-site reaction(s).<br>• On daratumumab administration days, it is recommended that lenalidomide is administered either prior to or at the same time (preferred) as the pretreatment medications.				
Safety Monitoring Requirement	• First 3 doses of teclistamab (0.06, 0.3, and 1.5 mg/kg) : Section 6.4.1 and Appendix 13<br>• Subsequent administration for participants who experience specified AEs (Grade 3 sARR, CRS, or ICANS): Section 6.4.1.2 and Section 7.1.2	• Induction Cycle 1 Day 1: see Section 6.4.2<br>• Participants at risk for respiratory complications: see Section 6.3	The decision to discharge from the site or hospital should be made in accordance with the prescribing information for bortezomib.	Not applicable.	Not applicable.

Table 24: Study Treatment Administration Instruction – Arms A, A1, [redacted] (Tec-DRd), and Arm B (Tec-DVRd) Induction

Study Drug	Teclistamab	Daratumumab SC	Bortezomib Arm B only	Lenalidomide	Dexamethasone
**Vital Signs**	On dosing days, vital signs should be performed as follows:<br>• Daratumumab SC and teclistamab: perform before start of administration.<br>• Monitor until resolution of CRS or sARR<br>• Any additional vital signs assessments supporting the start and end dates of an AE (eg, fever or hypertension) should be reported in the eCRF.				

* Following Amendment 3, all participants in Arm A have transitioned to monthly dosing of 3.0 mg/kg teclistamab.

6.1.3. Study Treatment Administered – CCI [redacted] and CCI [redacted] Induction

Table 25: Dose Schedule and Pretreatment Medications for Arms CCI [redacted] and Arms F CCI [redacted]) Induction

CCI [redacted]

Table 26: Study Treatment Administration Instruction – Arms CCI [redacted] **and Arms** CCI [redacted] **Induction**

CCI [redacted]

Table 26: Study Treatment Administration Instruction – Arms CCI [redacted] and Arms CCI [redacted] Induction

CCI

[redacted]

Table 26: Study Treatment Administration Instruction – Arms CCI [redacted] **and Arms** CCI [redacted] **Induction**

CCI [redacted]

6.1.5. Study Treatment Administration – CCI [redacted]

Table 27: CCI [redacted]

CCI [redacted]

Table 28: CCI [redacted]

CCI [redacted]

6.1.6. Study Treatment Administration – Arms A and C - Tec-DR Maintenance

Table 29: Dose Schedule and Pretreatment Medications for Arms A and C* - Tec-DR Maintenance

Activity		Maintenance Treatment Phase (28-day cycle)					
**Maintenance Cycle**	**Notes**	**Cycle 1**					**Cycles 2-18**
**Day**		**1**	**2**	**4**	**8**	**15**	**1**
**REQUIRED PRETREATMENT MEDICATIONS: See Section 6.2.1 for event-driven required pretreatment medications.**							
Dexamethasone 20 mg or equivalent (see Appendix 10)	• Oral/IV: administer 1-3 hours (±15 min) prior to administration of (first) study drug.<br>• On Cycle 1 Day 8 and per Table 30, an additional dose of dexamethasone (8 mg) must be given (and should be at least 15 minutes prior to teclistamab dosing) if >4 hours have elapsed since prior administration of dexamethasone (20 mg).	X	X	X	X	X	Dexamethasone should not be administered as pretreatment medication after Cycle 1 Day 15 except as described in Section 6.2.1 or upon discussion with the sponsor.
Diphenhydramine 25 to 50 mg, or equivalent	• Oral/IV: administer 1-3 hours (±15 min) prior to administration of (first) study drug.<br>• On Cycle 1 Day 8 and per Section 6.2.1, a second full dose must be given prior to teclistamab if >6 hours have elapsed since prior administration.	X	X	X	X	X	X
Acetaminophen 650 to 1000 mg, or equivalent		X	X	X	X	X	X
**OPTIONAL PRETREATMENT MEDICATIONS: Additional pretreatment medications such as H_2-antagonists or antiemetics may be used per investigator discretion.**							
Montelukast 10 mg	Oral: 1-3 hours (±15 min) prior to administration of daratumumab SC.	Per investigator discretion prior to administration of daratumumab					
**STUDY DRUGS: See Table 30 for additional detail, including the order and timing of administration of daratumumab SC and teclistamab. If they occur, CRS (fever, hypoxia, and hypotension) and ICANS must fully resolve before the next administration of teclistamab (see also criteria in Section 6.9.4.1). See Section 6.9.1.3 for information regarding acceptable windows for study drug administration**							
Teclistamab Step-up Dose	• Administer by SC injection.<br>• Step-up Dose 1: Administer on Cycle 1 Day 2; must be administered ≥20 hours after daratumumab SC given on Cycle 1 Day 1.<br>• Thereafter, there must be ≥2 days between step-up doses.		0.06 mg/kg	0.3 mg/kg			
Teclistamab Treatment Dose 1.5 mg/kg	• Administer by SC injection.<br>• The first treatment dose of teclistamab must be administered ≥2 days after step-up dose 2.<br>• Thereafter, there must be ≥5 days between each treatment dose in Cycles 1-2.				1.5 mg/kg	1.5 mg/kg	
Teclistamab Treatment Dose 3 mg/kg	• Administer by SC injection.<br>• There must be at least 14 days +/- 2 days between each treatment dose in Cycles 3+.						3 mg/kg
Daratumumab 1800 mg SC		X					X
Lenalidomide 10 mg	• Oral administration<br>• Lenalidomide will not be administered during Cycle 1; it will start on Day 1 of Cycle 2.<br>• Lenalidomide dose may need to be adjusted based on CrCl. See Section 6.9.4.2 and Table 56.<br>• Based on evolving safety data, the lenalidomide regimen may be adjusted at any timepoint by the sponsor (eg. lenalidomide dosing on days 1-21 only).						• Starting with Cycle 2 Day 1: 10 mg daily for 28 days<br>• May increase to 15 mg daily starting Cycle 5 Day 1 if tolerated

*Refer to Appendix 22 for dose schedule of Arm C (Table 72) in previous teclistamab biweekly dosing schedule.

Table 30: Study Treatment Administration Instructions – Arms A and C - Tec-DR Maintenance

<table>
<tr><th>Study Drug</th><th>Teclistamab</th><th>Daratumumab</th><th>Lenalidomide</th></tr>
<tr><td rowspan="2">Dosing Instruction</td><td>Teclistamab treatment will start with 2 step-up doses followed by treatment doses. All doses of teclistamab follow weight-based dosing (see Table 29 for details on doses)</td><td>The volume of 1800 mg for SC injection will be approximately 15 mL and administration should occur over approximately 3 to 5 minutes.</td><td>Lenalidomide will be supplied as 5 mg, 10 mg, and 15 mg capsules</td></tr>
<tr><td colspan="2">• SC injections will be prepared as described in the appropriate IPPI or equivalent documentation
• The anatomical area of administration must be recorded for both drugs in the eCRF and the area observed for injection-site reaction(s). See the IPPI or equivalent documentation.</td><td>• Lenalidomide will be self-administered orally.
• Lenalidomide should be taken as a single dose at the same time daily.
• Lenalidomide can be taken with or without food.
• Breaking or dividing lenalidomide capsules is strongly discouraged.
• Dose may need to be adjusted for participants with reduced CrCl (see Section 6.9.4.2).</td></tr>
<tr><td>Order of Study Drug Administration</td><td colspan="3">• When daratumumab SC and teclistamab are to be administered on the same day, daratumumab SC should be administered first.
• The following intervals between daratumumab SC and teclistamab should be followed:
o Step-up Dose 1 of teclistamab must be administered ≥20 hours after the first dose of daratumumab SC.
o Note the requirements for repetition of the required pretreatment medications if >4 hours (steroid) or >6 hours (antihistamine, antipyretic) have elapsed since prior administration of the pretreatment medication (see Table 29).
o Thereafter, teclistamab should be administered 15 minutes after daratumumab SC.
o Teclistamab will not be administered during or within 2 hours after resolution of any sARR related to daratumumab SC.
o If a participant experiences treatment delays of daratumumab SC longer than 3 months, the interval between daratumumab SC and teclistamab should be at least 3 hours for the first daratumumab dose.
• When more than one component of study treatment is administered SC on the same day, each SC component should be injected at different anatomical locations. See the IPPI or equivalent documentation for further details.
• For all components of study treatment that are administered SC, the anatomical area of administration must be recorded in the eCRF and the area observed for injection-site reaction(s).
• On daratumumab administration days, it is recommended that lenalidomide is administered either prior to or at the same time (preferred) as the pretreatment medications.</td></tr>
</table>

Table 30: Study Treatment Administration Instructions – Arms A and C - Tec-DR Maintenance

Study Drug	Teclistamab	Daratumumab	Lenalidomide
**Safety Monitoring Requirements**	• First 3 doses of teclistamab (0.06, 0.3, and 1.5 mg/kg): Section 6.4.1 and Appendix 13.<br>• Subsequent administration for participants who experience specified AEs (Grade 3 sARR, CRS, or ICANS): Section 6.4.1.2 and Section 7.1.2.	• Maintenance Cycle 1 Day 1: see Section 6.4.2<br>• Participants at risk for respiratory complications: see Section 6.3	Not applicable.
**Vital Signs**	On dosing days, vital signs (refer to Section 8.3.2) should be performed as follows:<br>• Daratumumab SC and teclistamab: perform before start of administration of each medication.<br>• Monitor until resolution of CRS or sARR.<br>• Any additional vital signs assessments supporting the start and end dates of an AE (eg, fever or hypertension) should be reported in the eCRF.		

6.1.7. Study Treatment Administration – Arm [redacted] – [redacted] Maintenance

Table 31: Dose Schedule and Pretreatment Medications for Arm [redacted] - [redacted] Maintenance

CCI [redacted]

Table 32 Study Treatment Administration Instructions for Arm [redacted] - [redacted] Maintenance

CCI

[redacted]

Table 32 Study Treatment Administration Instructions for Arm [redacted] - [redacted] Maintenance

[redacted]

6.1.8. Study Treatment Administration – Arms A, A1, B, C, C1, [redacted] Tec-D Maintenance

Table 33: Dose Schedule and Pretreatment Medications for Arms A, A1, B, C, C1, [redacted] – Tec-D Maintenance

Activity		Maintenance Treatment Phase (28-day cycle)					
**Maintenance Cycle**	**Notes**	**Cycle 1**					**Cycles 2-18**
**Day**		**1**	**2**	**4**	**8**	**15**	**1**
**REQUIRED PRETREATMENT MEDICATIONS: See Section 6.2.1 for event-driven required pretreatment medications.**							
Dexamethasone 20 mg or equivalent (see Appendix 10)	• Oral/IV: administer 1-3 hours (±15 min) prior to administration of (first) study drug.<br>• On Cycle 1 Day 8 and per Table 34, an additional dose of dexamethasone (8 mg) must be given (and should be at least 15 minutes prior to teclistamab dosing) if >4 hours have elapsed since prior administration of dexamethasone (20 mg).	X	X	X	X	X	Dexamethasone should not be administered as pretreatment medication after Cycle 1 Day 15 except as described in Section 6.2.1 or upon discussion with the sponsor.
Diphenhydramine 25 to 50 mg, or equivalent	• Oral/IV: administer 1-3 hours (±15 min) prior to administration of (first) study drug.<br>• On Cycle 1 Day 8 and per Section 6.2.1, a second full dose must be given prior to teclistamab if >6 hours have elapsed since prior administration.	X	X	X	X	X	X
Acetaminophen 650 to 1000 mg, or equivalent		X	X	X	X	X	X
**OPTIONAL PRETREATMENT MEDICATIONS: Additional pretreatment medications such as H_2-antagonists or antiemetics may be used per investigator discretion.**							
Montelukast 10 mg	Oral: 1-3 hours (±15 min) prior to administration of daratumumab SC.	Per investigator discretion prior to administration of daratumumab					
**STUDY DRUGS: See Table 34 for additional detail, including the order and timing of administration of daratumumab SC and teclistamab. If they occur, CRS (fever, hypoxia, and hypotension) and ICANS must fully resolve before the next administration of teclistamab (see also criteria in Section 6.9.4.1). See Section 6.9.1.3 for information regarding acceptable windows for study drug administration**							
Teclistamab Step-up Dose	• Administer by SC injection.<br>• Step-up Dose 1: Administer on Cycle 1 Day 2; must be administered ≥20 hours after daratumumab SC given on Cycle 1 Day 1.<br>• Thereafter, there must be ≥2 days between step-up doses.		0.06 mg/kg	0.3 mg/kg			
Teclistamab Treatment Dose 1.5 mg/kg	• Administer by SC injection.<br>• The first treatment dose of teclistamab must be administered ≥2 days after step-up dose 2.<br>• Thereafter, there must be ≥5 days between each treatment dose in Cycles 1-2.				1.5 mg/kg	1.5 mg/kg	
Teclistamab Treatment Dose 3 mg/kg	• Administer by SC injection.<br>• There must be at least 14 days +/- 2 days between each treatment dose in Cycles 3+.						3 mg/kg
Daratumumab 1800 mg SC		X					X

Table 34: Study Treatment Administration Instructions for Arms A, A1, B, C, C1, [redacted] – Tec-D Maintenance

<table>
<tr><th>Study Drug</th><th>Teclistamab</th><th>Daratumumab</th></tr>
<tr><td rowspan="2">Dosing Instruction</td><td>Teclistamab treatment will start with 2 step-up doses followed by treatment doses. All doses of teclistamab follow weight-based dosing (see Table 33 for details on doses)</td><td>The volume of 1800 mg for SC injection will be approximately 15 mL and administration should occur over approximately 3 to 5 minutes.</td></tr>
<tr><td colspan="2">• SC injections will be prepared as described in the appropriate IPPI or equivalent documentation
The anatomical area of administration must be recorded for both drugs in the eCRF and the area observed for injection-site reaction(s). See the IPPI or equivalent documentation.</td></tr>
<tr><td>Order of Study Drug Administration</td><td colspan="2">• When daratumumab SC and teclistamab are to be administered on the same day, daratumumab SC should be administered first.
• The following intervals between daratumumab SC and teclistamab should be followed:
o Step-up Dose 1 of teclistamab must be administered ≥20 hours after the first dose of daratumumab SC.
o Note the requirements for repetition of the required pretreatment medications if >4 hours (steroid) or >6 hours (antihistamine, antipyretic) have elapsed since prior administration of the pretreatment medication (see Table 33).
o Thereafter, teclistamab should be administered 15 minutes after daratumumab SC.
o Teclistamab will not be administered during or within 2 hours after resolution of any sARR related to daratumumab SC.
o If a participant experiences treatment delays of daratumumab SC longer than 3 months, the interval between daratumumab SC and teclistamab should be at least 3 hours for the first daratumumab dose.
• When more than one component of study treatment is administered SC on the same day, each SC component should be injected at different anatomical locations. See the IPPI or equivalent documentation for further details.
• For all components of study treatment that are administered SC, the anatomical area of administration must be recorded in the eCRF and the area observed for injection-site reaction(s).</td></tr>
<tr><td>Safety Monitoring Requirements</td><td>• First 3 doses of teclistamab (0.06, 0.3, and 1.5 mg/kg): Section 6.4.1 and Appendix 13.
• Subsequent administration for participants who experience specified AEs (Grade 3 sARR, CRS, or ICANS): Section 6.4.1.2 and Section 7.1.2.</td><td>• Maintenance Cycle 1 Day 1: see Section 6.4.2
Participants at risk for respiratory complications: see Section 6.3</td></tr>
<tr><td>Vital Signs</td><td colspan="2">On dosing days, vital signs (refer to Section 8.3.2) should be performed as follows:
• Daratumumab SC and teclistamab: perform before start of administration of each medication.
• Monitor until resolution of CRS or sARR.
• Any additional vital signs assessments supporting the start and end dates of an AE (eg, fever or hypertension) should be reported in the eCRF.</td></tr>
</table>

6.1.9. Study Treatment Administration – Arms CCI [redacted] Maintenance

Table 35: Dose Schedule and Pretreatment Medications for Arms CCI [redacted] Maintenance

CCI [redacted]

Table 36: Study Treatment Administration Instructions for Arms [redacted] Maintenance

[redacted]

6.2. Pretreatment Medications

Pretreatment medications will be administered per the relevant dose schedule in Section 6.1.

NOTE: Dexamethasone should not be administered as pretreatment medication after Induction or Maintenance Cycle 1 Day 15 except as described in Section 6.2.1 or upon discussion with the sponsor.

Additional pretreatment medications such as H_2-antagonists or antiemetics may be used per investigator discretion. Additionally, Section 6.2.1 summarizes pretreatment medications that are required for participants who experience specified AEs. See Section 6.12.2 for recommended concomitant medications for all participants.

6.2.1. Event-driven Pretreatment Medications

A participant in any Arm who experiences Grade ≥2 CRS/sARR related to study drug must receive dexamethasone 20 mg, diphenhydramine 25 to 50 mg or equivalent, and acetaminophen 650 to 1000 mg or equivalent, as pretreatment medication for at least the subsequent dose of the study drug to which the event was related. These event-driven pretreatment medications are not required if already being administered as planned pretreatment described in the relevant dose schedule in Section 6.1.

6.3. Posttreatment Medication for Daratumumab SC for Participants with Higher Risk of Respiratory Complications

For participants with a higher risk of respiratory complications (eg, participants with mild asthma or participants with COPD who have an FEV1 <80% at screening or developed FEV1 <80% during the study without any medical history) the following post-infusion medications should be considered:

- Antihistamine (diphenhydramine or equivalent)
- Leukotriene inhibitor (montelukast or equivalent)
- Short-acting β2 adrenergic receptor agonist such as salbutamol aerosol
- Control medications for lung disease (eg, inhaled corticosteroids ± long-acting β2 adrenergic receptor agonists for participants with asthma; long-acting bronchodilators such as tiotropium or salmeterol ± inhaled corticosteroids for participants with COPD).

In addition, these at-risk participants may be hospitalized for monitoring for up to 2 nights after daratumumab SC administration. If participants are hospitalized, then an improvement per investigator's discretion in FEV1 is required and must be documented prior to discharge. If the participant has not experienced a significant medical event but is hospitalized overnight only for observation, then the hospitalization should not be reported as an SAE. Investigators may prescribe bronchodilators, H_1-antihistamines, and corticosteroids that are deemed necessary to provide adequate supportive care in the event a bronchospasm occurs after participants are released from the hospital/clinic. If an at-risk participant experiences no major sARRs, then these post-infusion medications may be waived after 4 doses of daratumumab SC at the investigator's discretion.

6.4. Required Safety Monitoring

6.4.1. Safety Monitoring for Teclistamab and Talquetamab Dosing Through the First Three Doses

Participants will be admitted for inpatient monitoring for a minimum of 48 hours after the first 3 doses of teclistamab (0.06, 0.3, and 1.5 mg/kg) or talquetamab [redacted] will be admitted for inpatient monitoring for the first 3 doses of talquetamab dosing). Alternatively, under special circumstances (including for maintenance treatment if a participant is to receive the same bispecific antibody for maintenance as was received for induction) and after discussion and approval by the sponsor, participants may receive these doses as an outpatient (see Appendix 13 for guidance to be considered for outpatient dosing).

6.4.1.1. Guidance for Outpatient Administration of First Three Doses of Teclistamab or Talquetamab (only after consultation with the sponsor)

The following applies in the exceptional case of a participant receiving the first 3 doses of teclistamab (0.06, 0.3, and 1.5 mg/kg) or talquetamab [redacted] in the outpatient setting.

If the participant experiences any signs of CRS, ICANS, or other clinically significant event(s) following any of the first 3 doses of teclistamab (0.06, 0.3, and 1.5 mg/kg) or talquetamab [redacted] the participant will be hospitalized for a minimum of 48 hours. Additionally, if CRS/ICANS occurs after the first 2 doses of teclistamab (0.06 and 0.3 mg/kg) or talquetamab [redacted] the subsequent dose must occur inpatient. If CRS/ICANS occurs at the third dose of teclistamab (1.5 mg/kg) or talquetamab [redacted] or beyond, see Table 37 for guidance.

The participant should remain in close proximity (within 30 minutes) to the site and in the company of a competent adult for a minimum of 48 hours after first 3 doses of teclistamab (0.06, 0.3, and 1.5 mg/kg) or talquetamab [redacted] During this period, participants will be asked to check their temperature at least twice daily at an interval of ≥8 hours and record in a participant diary. They will be instructed to report any fever (≥38°C or ≥100.4°F) to the investigator immediately and be admitted to the hospital to initiate monitoring for development of CRS (see additional details in Appendix 13).

6.4.1.2. Required Hospitalization for Teclistamab or Talquetamab Dosing Following Specified Adverse Events

Any participant who experiences any of the specified AEs in Table 37 starting from the third dose of teclistamab or talquetamab must be hospitalized for administration of the next dose of teclistamab or talquetamab as indicated.

Table 37: Adverse Events Requiring Hospitalization for Subsequent Administration of Teclistamab or Talquetamab

Prior AE(s)	Safety Monitoring Requirement[a]
Grade 3 sARR	≥24 hours after administration of next dose of teclistamab or talquetamab
Grade 3 CRS	≥48 hours after administration of next dose of teclistamab or talquetamab
Grade 3 ICANS	

a. Consider hospitalization if Grade 2 laryngeal edema or Grade 2 bronchospasm occurs (Table 41).

6.4.1.3. Hospitalization Discharge Criteria

If a participant is hospitalized for administration of teclistamab or talquetamab, the following criteria must be met at minimum before the participant is discharged:

- Absence of fever, defined as a temperature ≤38°C, for ≥8 hours without use of antipyretics
- Absence of ICANS or suspected ICANS of CNS (any grade).

6.4.2. Outpatient Monitoring After Daratumumab SC Administration

It is recommended that all participants be observed for ≥6 hours after the end of the daratumumab SC injection on Induction and Maintenance Cycle 1 Day 1 and, if deemed necessary by the investigator, after subsequent injections.

Participants at risk for respiratory complications may be hospitalized for monitoring for up to 2 nights after injection of daratumumab SC (see Section 6.3).

6.5. Management Guidelines for Potential Toxicities

Management guidelines for potential toxicities of the study drugs are described in the following sections. Trained study staff at the clinic should be prepared to intervene and resources necessary for resuscitation (eg, agents such as epinephrine and aerosolized bronchodilator and medical equipment such as oxygen tanks, tracheostomy equipment, and a defibrillator) must be available nearby when participant is at high risk for developing CRS (ie, during first 3 doses of teclistamab [0.06, 0.3, and 1.5 mg/kg] or talquetamab CCI [redacted] or sARR (ie, during first 3 doses of teclistamab [0.06, 0.3, and 1.5 mg/kg] or talquetamab CCI [redacted] and the first dose of daratumumab SC).

AEs that require dosing interruption/dose reduction or study drug discontinuation, are discussed in Section 6.9 and Section 7.1, respectively.

6.5.1. CRS

As the mechanisms of action of teclistamab and talquetamab are based on the binding and activation of T cells and the release of cytokines in the tumor environment, CRS is anticipated. As noted in Section 2.1.4.1, Section 2.1.5.1, and Section 2.4.1.1, CRS is most likely to occur during the first 3 doses of teclistamab (0.06, 0.3, and 1.5 mg/kg) or talquetamab CCI [redacted] or potentially after a prolonged interruption in dosing of teclistamab or talquetamab and

has been observed mostly as Grade 1 or 2. The risk for CRS has been mitigated as discussed in Section 2.4.1.1 and Section 2.4.3 by step-up doses and pretreatment medications. Additionally, the participant will undergo safety monitoring, which is more intensive during the time of highest risk for CRS (see Section 6.4).

Clinical symptoms indicative of CRS may include, but are not limited to, fever (with or without rigors), arthralgia, nausea, vomiting, tachypnea, hypoxia, tachycardia, hypotension, headache, confusion, tremor, delirium, dyspnea, pulmonary edema, and capillary leak (Klinger 2012, Lee 2019). Potentially life-threatening complications of CRS may include cardiac dysfunction, adult respiratory distress syndrome, neurologic toxicity, renal failure, hepatic failure, and disseminated intravascular coagulation. Trained clinical personnel should be prepared to intervene in the event of CRS and resources described in Section 6.5 should be available.

6.5.1.1. Management Guidelines for CRS

In June 2018, members of the ASTCT convened to develop a consensus for severity grading of CRS and ICANS that may be induced by CAR-T cells and can be applied to other immunomodulatory therapeutics. The grading system and description of symptoms and signs was published in April 2019. Toxicity grading for CRS per ASTCT guidelines is described in Appendix 14. Symptoms of CRS will be graded according to NCI-CTCAE Version 5.0 and also reported in eCRF.

Table 38 provides recommendations for the clinical management of CRS. At the first sign of CRS (such as fever), dosing of all study drugs should be interrupted, and the participant should be hospitalized immediately for evaluation, if not already hospitalized (see Section 6.4).

Infection and CRS may have a similar presentation. Therefore, investigators are strongly encouraged to evaluate for an infection at the first signs or symptoms of CRS. However, treatment for CRS should not be delayed. Cultures and imaging should be obtained; the clinical signs and symptoms should determine which tests are appropriate.

Supportive care for CRS (including but not limited to antipyretic agents, IV fluid support, vasopressors, supplemental oxygen, etc) should be administered according to the clinical manifestations of the participant's illness (see Table 38). Laboratory testing including daily monitoring of chemistry and hematology assessments and coagulation laboratory tests should be performed; pulmonary, renal, and hepatic function must be monitored closely. Rarely, severe CRS can evolve into a presentation consistent with HLH/MAS that may require additional therapy.

Tocilizumab intervention should be considered in response to a presenting symptom of fever per investigator discretion if infection is not suspected or in the presence of persistent (>24 hours) fever. Early administration of tocilizumab should be considered in participants at high risk of severe CRS. Other monoclonal antibodies targeting cytokines (eg, anti-IL-1 and/or anti-TNF-α) may be used based on institutional standards, especially for cases of CRS which do not respond to tocilizumab. In the rare event that high-grade CRS with clinical findings overlapping with HLH/MAS occurs (including hyperferritinemia) and remains unresponsive to tocilizumab and

corticosteroids, additional therapy, including chemotherapy, may be considered in consultation with the sponsor. The use of growth factors, particularly G-CSF or GM-CSF, should be avoided during CRS.

See Section 6.4.1.2 for required monitoring for subsequent administration of teclistamab or talquetamab following an event of CRS. After an event of CRS, pretreatment medication is required as described in Section 6.2.1.

Table 38: Recommended Management of CRS

	Treatment Options	
**Presenting Symptoms**	**Tocilizumab**[a]	**Corticosteroids**[b]
Temperature ≥38°C[c]	May be considered	May be considered
Temperature ≥38°C[c] with either:<br>Hypotension responsive to fluids and not requiring vasopressors.<br>Or, oxygen requirement of low-flow nasal cannula[d] or blow-by	Administer tocilizumab[b] 8 mg/kg IV over 1 hour (not to exceed 800 mg).<br>Repeat tocilizumab every 8 hours as needed if not responsive to IV fluids or increasing supplemental oxygen.<br>Limit to a maximum of 3 doses in a 24-hour period; maximum total of 4 doses.	Manage per guidance below if no improvement within 24 hours of starting tocilizumab.
Temperature ≥38°C[c] with either:<br>Hypotension requiring 1 vasopressor with or without vasopressin.<br>Or, oxygen requirement of high-flow nasal cannula[d], facemask, non-rebreather mask, or Venturi mask	Administer tocilizumab 8 mg/kg IV over 1 hour (not to exceed 800 mg).<br>Repeat tocilizumab every 8 hours as needed if not responsive to IV fluids or increasing supplemental oxygen.<br>Limit to a maximum of 3 doses in a 24-hour period; maximum total of 4 doses.	If no improvement, administer methylprednisolone 1 mg/kg IV twice daily or equivalent dexamethasone (eg, 10 mg IV every 6 hours).<br>Continue corticosteroids use until the event is Grade 1 or less, then taper over 3 days.
Temperature ≥38°C[c] with either:<br>Hypotension requiring multiple vasopressors (excluding vasopressin).<br>Or, oxygen requirement of positive pressure (eg, CPAP, BiPAP, intubation, and mechanical ventilation)	Administer tocilizumab 8 mg/kg IV over 1 hour (not to exceed 800 mg).<br>Repeat tocilizumab every 8 hours as needed if not responsive to IV fluids or increasing supplemental oxygen.<br>Limit to a maximum of 3 doses in a 24-hour period; maximum total of 4 doses.	As above or administer methylprednisolone 1000 mg IV per day for 3 days per investigator discretion.<br>If no improvement or if condition worsens, consider alternate immunosuppressants.[b]

a. Refer to tocilizumab prescribing information (ACTEMRA USPI 2022) for details.
b. Monoclonal antibodies targeting cytokines may be considered based on institutional practice for unresponsive CRS.
c. Attributed to CRS. Fever may not always be present concurrently with hypotension or hypoxia as it may be masked by interventions such as antipyretics or anticytokine therapy (eg, tocilizumab or steroids).
d. Low-flow nasal cannula is ≤6 L/min, and high-flow nasal cannula is >6 L/min.

6.5.2. ICANS and Other Neurotoxicity

Based on the mode of action of teclistamab and talquetamab, ICANS or other neurotoxicities may occur. Early recognition of neurotoxicity is critical to management. CCI [redacted] CCI

[redacted] The investigator should also exclude other causes of neurological symptomatology, including infarction/bleeding or infection (bacterial and neurotrophic viruses) and consider cerebral spinal fluid analysis and CT/MRI.

A basic neurologic examination including the ICE tool will be performed during screening and at baseline (within 48 hours prior to the administration of teclistamab or talquetamab) and repeated as indicated after the first symptoms of ICANS are suspected until resolution.

6.5.2.1. Management Guidelines for ICANS

ICANS events will be graded per ASTCT guidelines (Appendix 16), including administration of the ICE tool (Appendix 15) to assess encephalopathy and evaluation of other neurologic domains. Symptoms of ICANS will be graded according to NCI-CTCAE Version 5.0 and also reported in eCRF.

At the first sign of ICANS, dosing of all study drugs should be interrupted, and the participant should be hospitalized immediately for evaluation, if not already hospitalized (see Section 6.4). Table 39 summarizes recommendations for the management of ICANS. Table 40 presents guidelines for the management of increased ICP/cerebral edema.

Table 39: Recommended Management of ICANS

Presenting Symptoms[a]	Concurrent CRS	No Concurrent CRS
ICE score 7-9[b]<br>or depressed level of consciousness[c]: awakens spontaneously.	Management of CRS as appropriate per Table 38. Monitoring of neurologic symptoms and consider neurology consultation and evaluation, per investigator discretion.	Monitor neurologic symptoms and consider neurology consultation and evaluation, per investigator discretion.
	Consider non-sedating, anti-seizure medicines (eg, levetiracetam) for seizure prophylaxis.	
ICE score-3-6[b]<br>or depressed level of consciousness[c]: awakens to voice.	Management of CRS as appropriate per Table 38. If no improvement after starting tocilizumab, administer dexamethasone[d] 10 mg IV every 6 hours if not already taking other corticosteroids. Continue dexamethasone use until the event is Grade 1 or less, then taper.	Administer dexamethasone[d] 10 mg IV every 6 hours. Continue dexamethasone use until the event is Grade ≤1, then taper.
	Consider non-sedating, anti-seizure medicines (eg, levetiracetam) for seizure prophylaxis. Consider neurology consultation and other specialists (ie, intensivists) for further evaluation, as needed.	
ICE score-0-2[b]<br>or depressed level of consciousness[c]: awakens only to tactile stimulus,<br>or seizures[c], either:<br>• any clinical seizure, focal or generalized, that resolves rapidly, or<br>• non-convulsive seizures on EEG that resolve with intervention,	Management of CRS as appropriate per Table 38. In addition, administer dexamethasone[d] 10 mg IV with the first dose of tocilizumab and repeat dose every 6 hours. Continue dexamethasone use until the event is Grade 1 or less, then taper.	Administer dexamethasone[d] 10 mg IV every 6 hours. Continue dexamethasone use until the event is Grade ≤1, then taper.

Table 39: Recommended Management of ICANS

<table>
<tr><th>Presenting Symptoms[a]</th><th>Concurrent CRS</th><th>No Concurrent CRS</th></tr>
<tr><td>or
• raised ICP: focal/local edema on neuroimaging[c].</td><td colspan="2">Consider non-sedating, anti-seizure medicines (eg, levetiracetam) for seizure prophylaxis. Consider neurology consultation and other specialists (ie, intensivists) for further evaluation, as needed.</td></tr>
<tr><td rowspan="2">ICE score-0[b]
or depressed level of consciousness[c] either:
• participant is unarousable or requires vigorous or repetitive tactile stimuli to arouse, or
• stupor or coma,
or seizures[c], either:
• life-threatening prolonged seizure (>5 min), or
• repetitive clinical or electrical seizures without return to baseline in between,
or motor findings[c]:
• deep focal motor weakness such as hemiparesis or paraparesis,
or raised ICP/cerebral edema[c], with signs/symptoms such as:
• diffuse cerebral edema on neuroimaging, or
• decerebrate or decorticate posturing, or
• cranial nerve VI palsy, or
• papilledema, or
• Cushing's triad.</td><td>Management of CRS as appropriate per Table 38. As above, or consider administration of methylprednisolone 1000 mg IV per day with first dose of tocilizumab and continue methylprednisolone 1000 mg IV per day for 2 or more days, per investigator discretion.</td><td>As above or consider administration of methylprednisolone 1000 mg IV per day for 3 days; if improves, then manage as above.</td></tr>
<tr><td colspan="2">Consider non-sedating, anti-seizure medicines (eg, levetiracetam) for seizure prophylaxis.
Consider neurology consultation and other specialists (ie, intensivists) for further evaluation, as needed.
In case of raised ICP/cerebral edema, refer to Table 40 for additional management guidelines.</td></tr>
</table>

a. Management is determined by the most severe event, not attributable to any other cause.
b. If the participant is arousable and able to perform Mental Status assessment, the following domains should be tested: orientation, naming, following commands, writing, and attention (see ICE tool in Appendix 15).
c. Attributable to no other cause.
d. All references to dexamethasone administration are dexamethasone or equivalent.

Table 40: Guidelines for the Management of Raised ICP/Cerebral Edema

- Elevate head of participant's bed to an angle of 30 degrees.
- If participant has an Ommaya reservoir, drain cerebrospinal fluid to target opening pressure of <20 mm Hg.
- Hyperventilation to achieve target $PaCO_2$ of 28 to 30 mm Hg but maintained for no longer than 24 hours.
- Consider neurology and/or neurosurgery consultation.
- Use high-dose corticosteroids with methylprednisolone IV 1 g/day, as recommended above.
- Hyperosmolar therapy with either mannitol (20 g/dL solution) or hypertonic saline (3% or 23.4%, as detailed below):
  - Mannitol: initial dose 0.5 to 1 g/kg; maintenance at 0.25 to 1 g/kg every 6 hours while monitoring metabolic profile and serum osmolality every 6 hours and withhold mannitol if serum osmolality is ≥320 mOsm/kg, or the osmolality gap is ≥40.
  - Hypertonic saline: initial 250 mL of 3% hypertonic saline; maintenance at 50 to 75 mL/hr while monitoring electrolytes every 4 hours, and withhold infusion if serum sodium levels reach ≥155 mEq/L.
  - For participants with imminent herniation: initial 30 mL of 23.4% hypertonic saline; repeat after 15 min, if needed.
- Consider IV anesthetics for burst-suppression pattern on electroencephalography.

6.5.2.2. Management Guidelines for CCI [redacted]

CCI [redacted]

[redacted]

6.5.3. Hypogammaglobulinemia

In MajesTEC-1, reduction of CD19$^+$ B cells was observed during the first cycle in subjects treated with teclistamab SC monotherapy at RP2D. Persistently decreased levels were noted at Cycle 3. Administration of immunoglobulin replacement for hypogammaglobulinemia should be considered per institutional guidelines. Due to the potential for reactions from IVIg infusions, it is recommended IVIg be avoided for a minimum of 48 hours after each step-up dose and also after the first treatment dose of teclistamab or talquetamab, as well as during any event of CRS. Additionally, the following guidance may be followed:

- Immunoglobulin replacement should be used to maintain serum IgG levels ≥4 g/L. It is recommended to administer immunoglobulin (ie, intravenous immunoglobulin 0.4 g/kg every 3 to 6 weeks). After reaching a steady-state, IgG levels should be measured every 3 months (Hayden 2022; Ludwig 2023).
- For serious or recurrent/chronic infections, immunoglobulin replacement should be administered per institutional guidelines.
- Where applicable (IgG heavy chain disease type), assessments to calculate the true IgG level should be considered by subtracting the monoclonal component IgG from the total IgG as a pragmatic approach to determine the underlying non−paraprotein IgG level (Mohan 2023; Giralt 2023).

6.5.4. Infection

Infection is frequently reported in patients with multiple myeloma due to both disease and treatment-related factors causing hypogammaglobulinemia and immunosuppression (Terpos 2015; Raje 2022). BCMA directed therapies cause depletion of B cells, resulting in hypogammaglobulinemia, and may increase the risk of infection (Mazahreh 2023). Infection, including severe events, is a known risk for subjects receiving teclistamab (see Table 13 and the IB).

6.5.4.1. Infection Prophylaxis

Prophylactic measures per institutional guidelines are recommended due to the susceptibility of patients with multiple myeloma to infections (Drayson 2019; Mohyuddin 2020). These include:

- Prophylactic immunoglobulin replacement (see Section 6.5.3)
- Prophylactic administration of antibiotics and antivirals per institutional guidelines (see Section 6.12.2.1)
- Pneumocystis carinii/jirovecii pneumonia prophylaxis (see Section 6.12.2.1)
- Prophylaxis for herpes zoster reactivation
  - Initiate antiviral prophylaxis to prevent herpes zoster reactivation within 1 week after the start of administration of study treatment and continue for 3 months following study treatment. Acceptable antiviral therapy includes acyclovir, famciclovir, or valacyclovir.

In addition to the above, see permitted therapies for management of cytopenias in Section 6.12.1.

During stem cell mobilization and intensification (HDT+ASCT) institutional guidelines apply.

6.5.4.2. Vaccinations

Vaccination is allowed per local guidelines (including annual influenza, respiratory syncytial virus, Streptococcus pneumoniae [pneumococcal vaccine], recombinant herpes zoster, and inactivated SARS CoV-2 vaccines; see also Appendix 19); however, some types of vaccines (eg, live, attenuated) are not permitted for participants receiving teclistamab or talquetamab (see Section 6.12.3). Note that antibody responses to vaccines may be suboptimal during study treatment (Ariza-Heredia 2015) and that the expected adverse reactions to vaccines (eg, fever) and CRS may have a similar presentation. It is recommended to provide the above vaccinations prior to the first dose of study drug (as appropriate) and administer vaccinations per schedule while receiving teclistamab.

Post-transplant vaccination series should be initiated as per local guidelines.

It is strongly recommended that participants receive a COVID-19 vaccination series post-transplant regardless of vaccination status prior to transplant based on the most recent available guidance (COVID-19 and BMT 2022; ASH-ASTCT COVID-19 Vaccination 2022).

For COVID-19 vaccinations, refer to Appendix 19.

6.5.4.3. Infection Management

See guidance regarding non-hematologic AEs for which dosing should be interrupted in Table 55.

It is recommended that empirical broad-spectrum antibiotics are commenced while performing diagnostic tests in participants with febrile neutropenia or signs/symptoms of infection per institutional guidelines (Raje 2022).

Targeted antimicrobial agents are recommended depending on clinical, radiological, and microbiological findings (Raje 2022).

For participants with persistent fever with undetermined cause, consider testing for opportunistic infections including new onset or reactivation of viral infections such as herpesviruses (eg, HSV,

VZV, CMV, EBV), parvovirus B19, and adenovirus. Diagnostic imaging should be considered as clinically indicated.

PML, which can be fatal, has also been reported in patients receiving teclistamab. Monitor any new onset of or changes in pre-existing neurological signs or symptoms. If PML is suspected, withhold treatment with teclistamab (and other study medication, as applicable) and initiate appropriate diagnostic testing. Discontinue teclistamab if PML is confirmed.

See guidance regarding requirements for restarting therapy with teclistamab, talquetamab, and daratumumab pertinent to infection in Section 6.9.4.1. Additionally, following any dosing interruption of teclistamab, talquetamab, or daratumumab SC, there must be no evidence of an active bacterial, viral, or fungal infection before proceeding to the next dose of study drug.

6.5.4.4. COVID-19 Infection

For prevention and treatment of COVID-19 infections, refer to Appendix 19.

6.5.4.5. HBV Reactivation

HBV reactivation is a potential risk of study treatment in either arm. Primary antiviral prophylaxis is permitted per local standard of care. HBV-DNA testing by PCR is mandatory for participants at risk for HBV reactivation (for timing of testing, see Section 8.3.5.3).

During and following study treatment, participants who have history of HBV infection (eg, anti-HBc-positive irrespective of anti-HBs status, anti-HBs-positive and unknown HBV vaccination history, or known history of prior HBV infection irrespective of Hep B serology findings) will be closely monitored for clinical and laboratory signs (including DNA PCR) of reactivation of HBV Q12 weeks (±4 weeks) from C1D1 up to 6 months after the last treatment dose and as clinically indicated. Where required by local law, the results of HBV testing may be reported to the local health authorities.

For participants who are diagnosed with HBV reactivation (ie, HBV-DNA becomes detectable) while on treatment, suspend treatment and any steroids. Manage participants according to current clinical guidelines and consider consulting a hepatitis disease expert as clinically indicated. Resumption of treatment with concomitant antiviral prophylaxis as per standard of care in participants whose HBV reactivation is adequately controlled should be discussed with physicians with expertise in managing HBV and approved by the sponsor.

6.5.4.6. HCV Reactivation

For participants who are diagnosed with HCV reactivation (ie, HCV-RNA becomes detectable) while on treatment, suspend study treatment and any steroids and institute appropriate treatment. Resumption of study treatment should be discussed with physicians with expertise in managing HCV and approved by the sponsor.

6.5.5. sARRs

sARRs may manifest as wheezing, flushing, hypoxemia, fever, chills, rigors, bronchospasm, headache, rash, pruritus, arthralgia, hypo- or hypertension, or other symptoms and may occur with SC administration of teclistamab, talquetamab, or daratumumab. If sARR occurs during the administration of teclistamab, talquetamab, or daratumumab SC, the injection should be interrupted immediately if possible. Symptoms of sARRs should be managed per the recommendations in Table 41. See additional information related to management of sARRs related to daratumumab SC in Section 6.5.5.1, including guidance about re-starting of daratumumab SC injection in the event of sARR.

After a sARR event, pretreatment medication should be administered per Section 6.2.1 for at least the subsequent dose of the study drug to which the event was related.

Table 41: Guidelines for the Management of sARRs

NCI-CTCAE Grade(s)	Presenting Symptoms	Recommended Treatment/Intervention
**Grade 1 or Grade 2**	Mild or moderate reaction; requires therapy interruption but responds promptly to symptomatic treatment	Start IV fluids; give diphenhydramine 50 mg (or equivalent) IV and/or paracetamol 650 to 1000 mg (acetaminophen); consider corticosteroids and bronchodilator therapy; monitor participant closely until recovery from symptoms.<br>Consider hospitalization if Grade 2 laryngeal edema or Grade 2 bronchospasm occurs.
**Grade 3**	Severe, prolonged (ie, not rapidly responsive to symptomatic medication; recurrence of symptoms following initial improvement; hospitalization indicated for other clinical sequelae [eg, renal impairment, pulmonary infiltrates])	Start IV saline infusion; recommend bronchodilators (if indicated), epinephrine 0.2 to 1 mg of a 1:1000 solution for subcutaneous administration or 0.1 to 0.25 mg of a 1:10000 solution injected slowly for IV administration, and/or diphenhydramine 50 mg IV with methylprednisolone 100 mg IV (or equivalent), as needed, and other drugs as appropriate.<br>Investigators should follow their institutional guidelines for the treatment of anaphylaxis.<br>Monitor until medically stable, per the investigator's medical judgment.<br>See Section 6.4.1.2 for required hospitalization for subsequent administration(s) of teclistamab or talquetamab after a Grade 3 sARR related to teclistamab or talquetamab.<br>Participants who experience a Grade 4 sARR must discontinue study treatment (see below).
**Grade 4**	Life-threatening; pressor or ventilator support indicated	
**Discontinuations of treatment**		See sARRs requiring discontinuation of study treatment in Section 7.1.2 (teclistamab or talquetamab) and Section 7.1.3 (daratumumab SC).
**General**		**Prophylactic medications (after initial event)** must be used as described in Section 6.2.1.
		In the case of late-occurring hypersensitivity symptoms (eg, appearance of a localized or generalized pruritis within 1 week after treatment), symptomatic treatment must be given (eg, oral antihistamine or corticosteroids), as appropriate.

6.5.5.1. Management of sARRs Related to Daratumumab SC

If a sARR develops during daratumumab SC administration, then the administration should be temporarily interrupted. Participants who experience AEs during daratumumab SC administration must be treated for their symptoms. Participants should be treated with paracetamol (acetaminophen), antihistamine, or corticosteroids, as needed. IV saline may be indicated. For bronchospasm, urticaria, or dyspnea, participants may require antihistamines, oxygen, corticosteroids, or bronchodilators. For hypotension, participants may require vasopressors. If ocular symptoms (including choroidal effusion, acute myopia, and acute angle-closure glaucoma) occur, interrupt daratumumab and seek immediate ophthalmologic evaluation prior to re-starting daratumumab. In the event of a life-threatening sARR (which may include pulmonary or cardiac events) or an anaphylactic reaction, daratumumab SC should be discontinued.

Refer to the SIPPM for further details regarding continuation of daratumumab SC administration if temporarily interrupted.

Grade 1 or 2 sARRs

If the investigator assesses a Grade 1 or 2 sARR to be related to administration of study treatment, then daratumumab SC administration should be interrupted. When the participant's condition is stable, daratumumab SC administration may be restarted at the investigator's discretion.

If the participant experiences a Grade ≥2 event of laryngeal edema, or a Grade ≥2 event of bronchospasm that does not respond to systemic therapy and does not resolve within 6 hours from onset, then the participant must be permanently discontinued from daratumumab SC treatment.

Grade ≥3 sARRs

For sARR AEs (other than laryngeal edema or bronchospasm) that are Grade 3, daratumumab SC administration must be stopped and the participant must be observed carefully until resolution of the AE or until the intensity of the event decreases to Grade 1, at which point daratumumab SC administration may be restarted at the investigator's discretion.

If the intensity of the AE returns to Grade 3 after restart of daratumumab SC administration, then the participant must be permanently discontinued from daratumumab SC treatment.

For sARR AEs that are Grade 4, the daratumumab SC administration must be stopped, and the participant permanently discontinued from daratumumab SC treatment.

Recurrent sARRs

If a Grade 3 sARR (or Grade ≥2 event of laryngeal edema or a Grade ≥2 event of bronchospasm) recurs during or within 24 hours after a subsequent daratumumab SC administration, the participant must be permanently discontinued from daratumumab SC treatment.

6.5.6. Injection-site Reactions

SC administration of any medication can be associated with localized injection-site reactions including, but not limited to, induration and erythema. Injection-site reactions should be managed per institutional standards.

6.5.6.1. Injection-site Reactions Related to Bortezomib

For participants with unacceptable toxicity at the local injection site despite dose modifications or change in injection concentration (see Section 6.9.4.3), bortezomib may be administered IV as a 3- to 5-second bolus injection. Refer to local prescribing information for further details regarding SC or IV administration.

6.5.7. Tumor Lysis Syndrome

Participants should be monitored for evidence of TLS, including monitoring of uric acid and phosphate. Management of TLS, including hydration/forced diuresis, correction of electrolyte disturbances (eg, hyperkalemia, hyperuricemia, and hypocalcemia), and rasburicase, is highly recommended. It is also recommended that high-risk participants (ie, those with a high tumor burden [as indicated by: ≥60% plasma cell infiltrate on the bone marrow biopsy or aspirate, whichever is higher, SPEP ≥5 g/dL, FLC ≥5000 mg/L; or multiple soft-tissue plasmacytomas], pre-existing renal impairment, or other risk factors as assessed by the investigator), be treated prophylactically in accordance with institutional standards (eg, rehydration, diuretics, allopurinol 300 mg daily, rasburicase, and medication to increase urate excretion/metabolism).

6.5.8. Immune-related AEs

Treatment with teclistamab or talquetamab may lead to specific immune-related AEs including but not limited to pneumonitis, colitis, hypophysitis, and hypothyroidism. Continuous, careful monitoring and timely management of immune-related AEs may help to mitigate more severe toxicity. Symptomatic and best supportive care measures for potential immune-related AEs should be in progress as soon as clinically indicated in accordance with institutional standards. These treatments may include corticosteroids and/or other immunosuppressive agents as required and interrupting or discontinuing teclistamab or talquetamab.

6.5.9. Rash (Participants Receiving Talquetamab)

A variety of rashes have been seen in clinical studies of talquetamab, ranging from local injection site erythema to generalized rashes. It is unknown if these rashes are mediated by target expression. Cutaneous reactions have also been reported in association with IMiD administration. Rashes can appear with early doses or be a late manifestation (beyond 1 cycle) and should be managed per institutional standards. It is important for site staff to recognize rashes on the body that are distinct from injection-site reactions. Monitoring of rash progression is critical and early intervention, including treatment with topical ointments and topical or systemic steroids, may be necessary. Rashes occurring during the first cycle should be managed more aggressively with topical steroids and early consideration of a short course of oral steroids to reduce the risk of rash progression. Rashes that have not responded to treatment should be evaluated by a dermatologic consult.

6.5.10. Nail Dysfunction (Participants Receiving Talquetamab)

Nail dysfunction, including brittle nails, cracking nails, painful nails, and loss of nails on both hands and feet, has been seen in multiple participants dosed with talquetamab. Nail changes are often a late manifestation (beyond the first cycle of talquetamab) and symptomatic management with nail soaks, topical moisturizers, and topical steroids is recommended. Nail complications that persist should be evaluated by a dermatologic consult.

Good hygienic practices should be followed. Participants should be advised to keep fingers and toes cleaned and nails trimmed (avoid aggressive trimming), and to wear comfortable shoes with extra room around the toes. Nail polish and imitation fingernails should not be worn until the nails have grown out and returned to normal. Participants should use gloves for housecleaning and gardening to minimize nail damage and prevent infection. The presence of infection should be monitored (periungual edema, erythema, tenderness, and/or discharge) with the acquisition of cultures and/or implementation of empiric antibiotic therapy as appropriate, based on clinical judgment and institutional standards.

6.5.11. Oral Toxicity (Participants Receiving Talquetamab)

Oral toxicity has typically manifested ageusia, dysgeusia, dry mouth, and dysphagia. These events are often low grade and do not lead to treatment discontinuations. Over time, due to changes in appetite/eating habits, significant weight loss may result. Weight change should be monitored regularly during therapy. Clinically significant weight loss should be further evaluated. Oral toxicity AEs have generally been managed with dose holds or skips; symptomatic management with mouth rinses (salt water, steroid liquid formulations), nutrition consult, pain management as indicated, and possibly a short course of oral steroids in consultation with the sponsor. Refractory symptoms may require further evaluation by an ear, nose, and throat or gastrointestinal specialist.

6.6. Preparation/Handling/Storage/Accountability

6.6.1. Teclistamab and Talquetamab Preparation/Handling/Storage

Teclistamab and talquetamab must be protected from light during storage and stored at controlled temperatures according to the label. Teclistamab and talquetamab must be prepared according to the sponsor's instructions provided in the IPPI. Detailed instructions for storage conditions and handling will accompany clinical drug supplies to the clinical study sites. The storage conditions are indicated on the label. Teclistamab and talquetamab will be stored according to applicable expiry dates. Refer to the IPPI for additional guidance regarding teclistamab and talquetamab preparation, handling, and storage.

6.6.2. Preparation/Handling/Storage for Other Combination Agents

Refer to the SIPPM and respective labels for daratumumab SC, bortezomib, lenalidomide, and dexamethasone for guidance on preparation, handling, and storage. SC daratumumab injections will be prepared as described in the site IPPI, SmPC, USPI, local prescribing information, or equivalent documentation.

6.6.3. Accountability

Except as noted in Section 6.1, study drugs will be administered or prescribed by qualified site staff, and the details of the components of each treatment combination will be recorded in the eCRF. For any study drug taken at home, participants should bring the container(s) with all remaining doses to each study visit for counting by site staff. Study accountability and additional details regarding study treatment are provided in the SIPPM or equivalent document.

The investigator is responsible for ensuring that all study treatment received at the site is inventoried and accounted for throughout the study. The study treatment administered to the participant must be documented on the treatment accountability form. All study drugs will be stored and disposed of according to the sponsor's instructions. Study site personnel must not combine contents of the study drug containers.

Study drugs must be handled in strict accordance with the protocol and the container label; they must be stored at the study site in a limited-access area or in a locked cabinet under appropriate environmental conditions. Unless otherwise instructed, unused study drug(s) must be available for verification by the sponsor's study site monitor during on-site monitoring visits. The sponsor may request the return of unused study drug(s), or, if approved by the sponsor, unused and used study drug may be destroyed at the study site. Disposition of all study drug(s) provided by the sponsor will be documented by study site personnel. When the study site is an authorized destruction unit and study drug(s) supplies are destroyed on-site, this must also be documented on the treatment return form.

Potentially hazardous materials containing hazardous liquids, such as used ampules, needles, syringes and vials, should be disposed of immediately in a safe manner and therefore will not be retained for intervention accountability purposes.

Study treatment should be dispensed under the supervision of the investigator or a qualified member of the study site personnel, or by a hospital/clinic pharmacist. Study treatment will be supplied only to participants in the study. Returned study drug(s) must not be dispensed again, even to the same participant. Study drug(s) may not be relabeled or reassigned for use by other participants. The investigator agrees neither to dispense the study drug(s) from, nor store it at, any site other than the study sites agreed upon with the sponsor. Further guidance and information for the final disposition of unused study treatments are provided in the IPPI and/or SIPPM.

6.7. Measures to Minimize Bias: Randomization and Blinding

Treatment Allocation

Participants will be assigned to either Arm A1 or Arm B in parallel based on a schedule prepared before the study, by or under the supervision of the sponsor.

Participants will be assigned to Arm [redacted] or Arm [redacted] in parallel, and to Arm [redacted] (if opened) or Arm [redacted] (if opened) in parallel based on a schedule prepared before the study, by or under the supervision of the sponsor.

(If it is decided that other combinations of arms are to be assigned in parallel, for example due to the flexibility on opening or expanding an arm, then allocation will be based on a schedule prepared before the study, by or under the supervision of the sponsor.)

Blinding

As this is an open study, blinding procedures are not applicable.

6.8. Study Treatment Compliance

Study treatment is to be prescribed only by the principal investigator or a qualified physician listed as a sub-investigator on required forms. Drug supplies for each participant will be inventoried and accounted for throughout the study. Dispensing/administration of the study drug(s) must also be recorded in the participant's source documents.

An IWRS will be used to assign centrally supplied study treatment kits for each participant enrolled in the study. Study treatment may not be used for any purpose other than that outlined in this protocol, including other human studies, animal investigations, or in vitro testing.

For study treatment, administered at the study site, the details of each administration will be recorded in the eCRF (including date, start and stop times of the administration of study drug(s), location of administration, and the volume injected, as applicable). Precautions associated with the use of the components of study treatment and prohibited or restricted concomitant therapies will be reviewed with the participant. Study drug(s) start and stop dates, including dates for any dosing interruptions or dose reductions, will also be recorded in the eCRF.

Instructions for proper self-administration and at home study drug storage conditions should be provided for study drug(s) that will be administered at home. For these study drug(s), the amount dispensed will be recorded and compared with the amount brought to each study visit. Compliance with oral study drug(s) will be assessed at each visit (by recording and reconciling the number of tablets dispensed to and taken by each participant) and documented in the source documents and eCRF.

Deviation(s) from the prescribed dose schedule should be recorded in the eCRF. Any dose/dosage adjustment should be overseen by medically qualified study site personnel (principal or sub-investigator unless an immediate safety risk appears to be present). Except for management of TEAEs, if the dosing compliance is not 100%, then the investigator or designated study site personnel should re-instruct the participant regarding proper dosing procedures, and the participant may continue study treatment. If multiple instances of noncompliance occur, the sponsor's medical monitor should be notified.

6.9. Dosing Interruption and Dose Reduction

Toxicities should be attributed, whenever possible, to a specific component of study treatment so that dosing interruption, dose skipping, or (if applicable) dose reduction can be made appropriately.

Dosing interruption is the primary method of management of AEs for teclistamab, talquetamab, or daratumumab SC. Dose reduction is not permitted for daratumumab SC. Dose reductions of teclistamab during step-up dosing and Cycle 1 are not permitted. Dose reduction(s) of teclistamab by 50% or a change in dosing frequency may be considered starting in Cycle 2 for participants with teclistamab-related AE after consultation with and approval by the sponsor. A re-escalation or return to protocol-specified dosing schedule may be permitted in the absence of a recurrence of the AE that led to a reduction and if there is a clinical benefit and manageable risk profile in the

judgment of the investigator. Dose reduction and dosing interruptions may be implemented for bortezomib, lenalidomide, and dexamethasone as indicated. Dose modifications of talquetamab may be permitted starting in Cycle 2 after discussion with and approval by the sponsor.

Table 42,Table 43,and Table 44 summarize specific requirements for dosing interruption and dose reduction for each study drug used for induction regimens, the [redacted] regimen, and maintenance regimens, respectively.

Table 42: Guide to Dosing Interruption and Dose Reduction of Study Treatment for Induction Regimens

<table>
<tr><th rowspan="2">Study Drug</th><th colspan="2">Dosing Interruption</th><th colspan="2">Dose Reduction</th></tr>
<tr><th>Hematologic AE</th><th>Non-hematologic AE</th><th>Hematologic AE</th><th>Non-hematologic AE</th></tr>
<tr><td>Teclistamab</td><td rowspan="5">Section 6.9.3</td><td rowspan="3">Section 6.9.4.1</td><td colspan="2">Discuss with sponsor</td></tr>
<tr><td>Talquetamab</td><td colspan="2">Discuss with sponsor</td></tr>
<tr><td>Daratumumab SC</td><td colspan="2">Not permitted</td></tr>
<tr><td>Lenalidomide</td><td>Section 6.9.4.2</td><td>Section 6.9.3</td><td>Section 6.9.4.2</td></tr>
<tr><td>Bortezomib</td><td>Section 6.9.4.3</td><td>Section 6.9.3</td><td>Section 6.9.4.3</td></tr>
<tr><td>Dexamethasone</td><td colspan="4">Section 6.9.4.4</td></tr>
</table>

Table 43: Guide to Dosing Interruption and Dose Reduction of Study Treatment for Arm [redacted] for the [redacted] Regimen

<table>
<tr><th rowspan="2">Study Drug</th><th colspan="2">Dosing Interruption</th><th colspan="2">Dose Reduction</th></tr>
<tr><th>Hematologic AE</th><th>Non-hematologic AE</th><th>Hematologic AE</th><th>Non-hematologic AE</th></tr>
<tr><td>[redacted]</td><td rowspan="2">Section 6.9.3</td><td rowspan="2">Section 6.9.4.1</td><td colspan="2">Discuss with sponsor</td></tr>
<tr><td>[redacted]</td><td colspan="2">Discuss with sponsor</td></tr>
</table>

Table 44: Guide to Dosing Interruption and Dose Reduction of Study Treatment for Maintenance Regimens

<table>
<tr><th rowspan="2">Study Drug</th><th colspan="2">Dosing Interruption</th><th colspan="2">Dose Reduction</th></tr>
<tr><th>Hematologic AE</th><th>Non-hematologic AE</th><th>Hematologic AE</th><th>Non-hematologic AE</th></tr>
<tr><td>Teclistamab</td><td rowspan="4">Section 6.9.3</td><td rowspan="3">Section 6.9.4.1</td><td colspan="2">Discuss with sponsor</td></tr>
<tr><td>Talquetamab</td><td colspan="2">Discuss with sponsor</td></tr>
<tr><td>Daratumumab SC</td><td colspan="2">Not permitted</td></tr>
<tr><td>Lenalidomide</td><td>Section 6.9.4.2</td><td>Section 6.9.3</td><td>Section 6.9.4.2</td></tr>
</table>

Discontinuation criteria are presented in Section 7.1. Participants who discontinue treatment with any 1 or more component(s) of study treatment may continue to receive treatment with the other components of study treatment, as assigned.

6.9.1. Guidance for Dosing Interruption and Skipping (All Study Drugs)

6.9.1.1. Cycle Delays

If a participant experiences an AE(s) such that none of the study drugs in the regimen can be given on Day 1 of a cycle, the cycle must be delayed and dosing of all study drugs held (see Section 6.1 for special instructions for Cycle 1 Day 1). If Day 1 of a cycle is delayed, the full cycle duration will remain 28 days, and Day 1 of subsequent cycles should be adjusted accordingly to maintain the 28-day cycle duration. Day 1 of a cycle must not be skipped.

If at least 1 component of the regimen can be given, the cycle can begin as planned.

6.9.1.2. Dosing Interruption of a Study Drug During a Cycle

If a component of the regimen cannot be given on a dosing day after Day 1 for reasons other than CRS or ICANS (see Table 55), the other study drugs should be administered as scheduled.

6.9.1.3. Skipping of Study Drugs

See Section 7.1 for information regarding permanent discontinuation of study drug(s).

Teclistamab, Talquetamab, and Daratumumab SC

Except on Day 1 of a cycle, if a treatment dose of teclistamab, talquetamab, or daratumumab SC does not occur within the prespecified window (Table 45 and Table 47), the dose will be considered as skipped and not made up. Step-up doses of teclistamab or talquetamab cannot be skipped but may be delayed. Following a skipped treatment of teclistamab, talquetamab, or daratumumab SC, administration may resume at the next planned dosing date.

If a dose of teclistamab or talquetamab is delayed, therapy should be restarted based on the recommendations listed in Table 45, Table 46, and Table 47. The treatment schedule of teclistamab, talquetamab, and daratumumab should be adjusted to maintain the appropriate treatment interval as specified in the relevant Schedule of Events in Section 6.1. Please note, disease evaluations as specified in the relevant Schedule of Activities and Section 8.2 must be performed by the central laboratory for all treatment regimens, even if treatment has been delayed for any reason, regardless of any changes to the dosing regimen. A delay of >28 days from planned dose not administered must be discussed and approved by the sponsor before re-starting teclistamab or talquetamab. Pretreatment medications including dexamethasone (see Section 6.2) must be administered prior to the repeat step-up doses and repeat treatment doses of teclistamab or talquetamab specified in Table 48 and Table 49. Hospitalization is recommended if step-up doses are repeated.

Table 45: Teclistamab, Talquetamab, and Daratumumab SC Dose Skips during Tec-DRd, Tec-DVRd, [redacted] Induction

Study Drug(s)		Cycles	Dosing Frequency	Skip Dose if Dosing Interrupted	Resume Dosing
Teclistamab	Step-up doses	Cycle 1	See Table 23	May not be skipped	
	Initial 1.5 mg/kg dose	Cycle 1	See Table 23	May not be skipped	
	Subsequent 1.5 mg/kg	Cycle 1	Weekly	>7 days from planned dose date	Next planned dosing day[a]
	3 mg/kg	Cycles 2+	Q4W	>3 days from planned dose date[b,c]	Next planned Q4W dosing day[b]
Talquetamab	[redacted]				
Daratumumab SC		Cycles 1-2	Weekly	>3 days from planned dose date	Next planned weekly dosing day
		Cycles 3-6	Q2W	>7 days from planned dose date	Next planned Q2W dosing day

a. Participants who skip C1D15 dose should proceed to Cycle 2 Day 1 dosing.
b. Day 1 of a cycle cannot be skipped. If teclistamab or talquetamab cannot be given, delay cycle and adjust Day 1 of subsequent cycles accordingly to maintain a 28-day cycle.
c. Only applicable if daratumumab was started on Day 1. If the daratumumab was not started on Day 1 the cycle should be delayed.

***NOTE:** Teclistamab and daratumumab SC dose skips during Arm A Tec-DR induction with teclistamab weekly dosing schedule (Table 73) are available in Appendix 22 (Section 10.22.4)

Table 46: Teclistamab and Talquetamab Dose Skips During [redacted] Treatment

Study Drug(s)		Cycles	Dosing Frequency	Skip Dose if Dosing Interrupted	Resume Dosing
Teclistamab[a]	3 mg/kg	Cycles 1+	Q4W	Not applicable[b]	Next planned Q4W dosing day
Talquetamab[a]	[redacted]				

a. If one drug is held for an AE then the other can continue, and when the AE has resolved both drugs should be synchronized to the same day at the next scheduled dosing day.
b. Day 1 of a cycle cannot be skipped. If talquetamab or teclistamab cannot be given, delay cycle and adjust Day 1 of subsequent cycles accordingly to maintain a 28-day cycle.

Table 47: Teclistamab, Talquetamab, and Daratumumab SC Dose Skips During Tec-DR, Tec-D, [redacted] Maintenance*

Study Drug(s)		Cycles	Dosing Frequency	Skip Dose if Dosing Interrupted	Resume Dosing
Teclistamab	Step-up doses	Cycle 1	See Table 29	May not be skipped	
	Initial 1.5 mg/kg dose	Cycle 1	See Table 29	May not be skipped	
	Subsequent 1.5 mg/kg	Cycle 1	Weekly	>7 days from planned dose date	Next planned dosing day[a]
	3 mg/kg	Cycles 2+	Q4W	>3 days from planned dose date[b,c]	Next planned Q4W dosing day[b]
Talquetamab	[redacted]				
Daratumumab SC		Cycles 1-18	Monthly	>14 days from planned dose date	Next planned monthly dosing day

a. Participants who skip C1D15 dose should proceed to Cycle 2 Day 1 dosing.

b. Day 1 of a cycle cannot be skipped. If teclistamab or talquetamab cannot be given, delay cycle and adjust Day 1 of subsequent cycles accordingly to maintain a 28-day cycle.

c. Only applicable if daratumumab was started on Day 1. If the daratumumab was not started on Day 1 the cycle should be delayed.

***NOTE:** Teclistamab and daratumumab SC dose skips during Arm C Tec-DR maintenance, with teclistamab biweekly dosing (Table 74) are available in Appendix 22 (Section 10.22.4).

When treatment is resumed, use the allowable dosing windows and required gaps between study drug doses (see Table 23, Table 25, Table 29, and Table 31) to eventually restore a regular teclistamab-/talquetamab-daratumumab dosing schedule.

See Section 7.1.3 regarding the duration of dosing interruption of daratumumab SC that requires discontinuation. Note that sARRs or CRS may occur upon re-initiation of daratumumab SC, teclistamab, or talquetamab after a prolonged dosing interruption. A disease evaluation to confirm whether disease has progressed may be required prior to resuming study treatment.

Table 48 Recommendations For Teclistamab Dosing After Delays in Treatment

Last Dose Administered Prior to Delay	Duration From the Last Dose Administered	Initial Action	Subsequent Action
**Critical Notes:**<br>• **For guidance on disease evaluations refer to Section 8.2.**<br>• **Please refer to Section 7.1.2 for sponsor approval needed to continue teclistamab treatment following dose interruption.**<br>• **Please refer to Section 6.9 and obtain sponsor approval for proposed dose reduction or dosing frequency change.**			
Step-up Dose 1<br><br>(0.06 mg/kg, Initial or Repeat)	7 days or less	Continue teclistamab at Step-Up Dose 2 (0.3 mg/ kg).[a] Then proceed to 1.5 mg/kg[a] treatment dose ≥2 days later.	After the required 1.5 mg/kg dose, resume planned treatment per Schedule of Activities.[b]<br><br>**Note:** If participant's cycle specifies 3 mg/kg as the next scheduled dose after the required 1.5 mg/kg dose above, please defer this dose to Day 1 of the following cycle.
	More than 7 days	Restart teclistamab at Step-up dose 1 (0.06 mg/kg).[a]<br>Administer Step-up Dose 2 (0.3 mg/kg)[a] ≥2 days later. Then proceed to 1.5 mg/kg[a] treatment dose ≥2 days later.	
Step-Up Dose 2 (0.3 mg/kg, Initial or Repeat)	7 days or less	Continue teclistamab at 1.5 mg/kg treatment dose.[a]	After the required 1.5 mg/kg dose, resume planned treatment per Schedule of Activities.[b]<br><br>**Note:** If participant's cycle specifies 3 mg/kg as the next scheduled dose after the required 1.5 mg/kg dose above, please defer this dose to Day 1 of the following cycle.
	8 to 28 days	Repeat Step-up dose 2 (0.3 mg/kg).[a] Then proceed to 1.5 mg/kg[a] treatment dose ≥2 days later.	
	More than 28 days	Restart teclistamab at Step-up dose 1 (0.06 mg/kg).[a]<br>Administer Step-up Dose 2 (0.3 mg/kg)[a] ≥2 days later. Then proceed to 1.5 mg/kg[a] treatment dose ≥2 days later.	
Treatment Dose (1.5 mg/kg or 3 mg/kg)	63 days or less	Resume planned treatment per Schedule of Activities.[b]	Continue planned treatment per Schedule of Activities.
	64 to 112 days	Restart teclistamab at Step-up dose 2 (0.3 mg/kg).[a] Then proceed to 1.5 mg/kg[a] treatment dose ≥2 days later.	After the required 1.5 mg/kg dose, resume planned treatment per Schedule of Activities.[b]<br><br>**Note:** If participant's cycle specifies 3 mg/kg as the next scheduled dose after the required 1.5 mg/kg dose above, please defer this dose to Day 1 of the following cycle.
	More than 112 days	Restart teclistamab at Step-up dose 1 (0.06 mg/kg).[a]<br>Administer Step-up Dose 2 (0.3 mg/kg)[a] ≥2 days later. Then proceed to 1.5 mg/kg[a] treatment dose ≥2 days later.	

a. Administer pretreatment medications, including steroids, prior to teclistamab dose and monitor participants accordingly.

b. Dosing may also resume at reduced dose or frequency as agreed by the sponsor.

Table 49: Recommendations CCI [redacted] **Dosing After Delays in Treatment**

CCI [redacted]

Lenalidomide

Lenalidomide should be taken at the same time each day. If a dose of lenalidomide is missed and <12 hours have elapsed from the time of the missed regular dose administration, the participant should take the missed dose as soon possible and continue with the next dose at the normal time. If a dose is missed and ≥12 hours have passed since the time of regular dose administration, the missed dose should be skipped and not made up (see the prescribing information). The participant should not take 2 doses at the same time. If dosing of lenalidomide is interrupted, the skipped doses should not be made up.

Bortezomib

Skipped doses of bortezomib will not be made up later in the cycle.

Dosing Interruptions Due to Reasons Other Than Toxicity

If dosing has not occurred for >28 days for reasons other than toxicity, the sponsor should be consulted for resuming treatment.

6.9.2. Guidance for Dose Reduction

Dose reduction is not permitted for daratumumab. In exceptional circumstances, dose reductions or changes in frequency of teclistamab or talquetamab could be considered starting in Cycle 2 after consultation with the sponsor. Dose reduction guidelines are provided in Table 50 and Table 51 for lenalidomide, Table 52 for bortezomib, and Table 53 for dexamethasone. Physician discretion and clinical judgment should prevail. If the investigator determines that an AE may be related to lenalidomide, bortezomib, or dexamethasone, the dose may be adjusted even if not specified in this protocol. Once dose reduction has been implemented for a participant, a return to the protocol-specified dosing level should not occur for that participant unless in the judgment of the investigator there is clinical benefit and a reasonable and manageable risk profile. Approval from the sponsor is also required for resumption of the protocol-specified dose.

Table 50: Dose Reduction Guidance for Lenalidomide Adverse Events for Arms A, A1, and [redacted] (Tec-DRd), Arm B (Tec-DVRd), Arms [redacted] and [redacted] ([redacted]), and Arms [redacted] and [redacted] ([redacted]) Induction Treatment

	Lenalidomide Dose
Starting dose	25 mg Days 1-21
1st dose reduction	15 mg Days 1-21
2nd dose reduction	10 mg Days 1-21
3rd dose reduction	5 mg Days 1-21
4th dose reduction	Discontinue lenalidomide permanently (see Section 7.1.4)

Table 51: Dose Reduction Guidance for Lenalidomide Adverse Events for Arms A and C (Tec-DR), and Arm [redacted] ([redacted] Maintenance Treatment

	Lenalidomide Dose	Lenalidomide Dose
Starting dose	10 mg Days 1-28	Increased to 15 mg Days 1-28
1st dose reduction	5 mg Days 1-28	10 mg Days 1-28
2nd dose reduction	5 mg Days 1-21	5 mg Days 1-28
3rd dose reduction	Discontinue lenalidomide permanently (see Section 7.1.4)	5 mg Days 1-21
4th dose reduction	Not applicable	Discontinue lenalidomide permanently (see Section 7.1.4)

Table 52: Dose Reduction Guidance for Bortezomib Adverse Events for Arm B (Tec-DVRd) and Arms [redacted] and [redacted] ([redacted]) Induction Treatment

	Bortezomib Dose
Starting dose	1.3 mg/m^2
1st dose reduction	1.0 mg/m^2
2nd dose reduction	0.7 mg/m^2
3rd dose reduction	Discontinue bortezomib (see Section 7.1.5)

Table 53: Dose Reduction Guidance for Dexamethasone Adverse Events for Arms A, A1, and [redacted] (Tec-DRd), Arm B (Tec-DVRd), Arms [redacted] and [redacted] ([redacted]), and Arms [redacted] and [redacted] ([redacted]) Induction Treatment

	Dexamethasone Dose
Starting dose	20 mg
1st dose reduction	12 mg
2nd dose reduction	8 mg
3rd dose reduction	Discontinue dexamethasone (see Section 7.1.6)

6.9.3. Hematologic Adverse Events

Dosing of teclistamab, talquetamab, daratumumab SC, lenalidomide, or bortezomib must be interrupted due to hematologic AEs per Table 54. Subsequently, the dose of lenalidomide or bortezomib should be reduced as indicated (see also Table 50, Table 51, and Table 52). The dose of daratumumab cannot be reduced. Dose reduction for teclistamab or talquetamab is generally not permitted; refer to Section 6.9 for guidance on exceptional circumstances addressing teclistamab or talquetamab dose reduction. Growth factors (eg, G-CSF) for management of cytopenias should be considered where appropriate.

Table 54: Study Drug Dosing Interruption or Dose Reduction (Lenalidomide or Bortezomib Only) Due to Hematologic Adverse Events

Hematologic AEs	Daratumumab SC	Teclistamab	Lenalidomide[a]	Bortezomib	Talquetamab
**Neutropenia**					
Any of the following:<br>• ANC $<0.5\times10^9$/L (Grade 4)<br>• febrile neutropenia (fever ≥38.5°C and ANC $<1\times10^9$/L)<br>• ANC $<1\times10^9$/L and infection of any grade	**Interrupt**	**Interrupt**	**Interrupt**	**Interrupt**	**Interrupt**
	Monitor CBC weekly				
ANC return to $\geq1\times10^9$/L	Resume	Resume	Resume 1 dose level lower than previous dose (see Table 50 and Table 51)	First occurrence: Resume at the same dose as previously.<br>For each subsequent occurrence, resume 1 dose level lower than previous dose (see Table 52).	Resume
**Thrombocytopenia**					
Grade 3					
Platelet count 25-49$\times10^9$/L	**Interrupt**	**Interrupt**	**Interrupt**	**Interrupt until recovery to baseline or Grade ≤2.**	**Interrupt**
	Monitor CBC weekly				
Platelet count return to $\geq50\times10^9$/L	Resume	Resume	Resume at previous dose or 1 dose level lower than previous dose (see Table 50 and Table 51)	Resume at previous dose or 1 dose level lower than previous dose. For recurrence, reduce by 1 dose level (see Table 52).	Resume
Grade 4					
Platelet count $<25\times10^9$/L	**Interrupt**	**Interrupt**	**Interrupt**	**Interrupt**	**Interrupt**
	Monitor CBC weekly				
Platelet count return to $\geq50\times10^9$/L	Resume	Resume	Resume at 1 dose level lower than previous dose (see Table 50 and Table 51)	Resume at 1 dose level lower than previous dose (see Table 52)	Resume
**Anemia**					
Grade 4	**Interrupt**	**Interrupt**	**Interrupt**	**Interrupt**	**Interrupt**
	Monitor CBC weekly				
Return to Grade ≤3	Resume	Resume	Resume at previous dose	Resume at previous dose or 1 dose level lower than previous dose (see Table 52).	Resume

NOTE: See Section 6.9.2 for additional guidance regarding dose reduction and requirements for subsequent dose escalation.

a. Note that Table 54 represents suggested does modifications of lenalidomide; however, physician discretion and clinical judgment should prevail. If the investigator determines that an AE may be related to lenalidomide, the dose may be adjusted even if not specified in this protocol. Once dose reduction has been implemented for a participant, a return to the protocol-specified dosing level should not occur for that participant unless in the judgment of the investigator there is clinical benefit and a reasonable and manageable risk profile. Approval from the sponsor is also required for resumption of the previous dose.

6.9.4. Non-hematologic Adverse Events

6.9.4.1. Teclistamab, Talquetamab, and Daratumumab SC

Dosing of teclistamab, talquetamab, and daratumumab SC must be interrupted due to non-hematologic AEs per Table 55. Additionally, teclistamab, or talquetamab must not be administered during or within 2 hours after resolution of any sARR related to daratumumab SC (see Table 24, Table 26, Table 30, Table 32, Table 34, and Table 36). Note that an extended delay between administration of daratumumab SC and teclistamab or talquetamab may require repetition of pretreatment medications (see Table 23, Table 25, Table 29, Table 31, Table 33, and Table 35).

Table 55: Teclistamab, Talquetamab, and Daratumumab SC Dosing Interruption for Non-hematologic Adverse Events

Non-hematologic AEs	Teclistamab	Talquetamab	Daratumumab SC
First sign of CRS (see Section 6.5.1)[a]	X	X	X
First sign of ICANS (see Section 6.5.2)[a]	X	X	X
Grade ≥3 TEAEs (except disease-related pain) or Grade ≥3 laboratory toxicity that are clinically significant (eg, requiring intervention and/or associated with an AE)[b]	X	X	X
HBV reactivation (see Section 6.5.4.5)	X	X	X
Any grade infection prior to/during step-up dosing and throughout subsequent treatment doses[c]	X	If deemed related to talquetamab	Investigator discretion
CCI [redacted]		X	

a. See Section 7.1.2 for CRS or ICANS events that require permanent discontinuation of teclistamab or talquetamab.
b. Isolated laboratory hypogammaglobulinemia does not require dose interruption of any drug. Refer to Section 6.5.3 for guidance on immunoglobulin replacement.
c. Localized Grade 1 infections that are not at risk of becoming systemic according to investigator judgement, do not require teclistamab dosing interruption.
d. Treatment with talquetamab should only be resumed after discussion with medical monitor.

Following dosing interruption of teclistamab or talquetamab due to one or more of the non-hematological AEs listed in Table 55, any clinically significant non-hematologic AE other than CRS or ICANS must resolve to Grade ≤2 or baseline (as documented in the medical history) before proceeding to the next dose. CCI [redacted] CRS (fever, hypoxia, and hypotension) and ICANS must fully resolve (ie, end date reported in the eCRF) before the next administration of teclistamab and the following criteria must be met:

- Minimum of 12 hours since last fever without use of antipyretics

- Minimum of 24 hours since last administration of tocilizumab or equivalent (eg, siltuximab, anakinra).

Additionally, following any dosing interruption of teclistamab, talquetamab, or daratumumab SC, there must be no evidence of an active bacterial, viral, or fungal infection before proceeding to the next dose.

6.9.4.2. Lenalidomide

Renal Impairment Associated with Lenalidomide

Lenalidomide is primarily excreted unchanged by the kidney. Therefore, adjustments to the dose of lenalidomide are recommended to provide appropriate drug exposure in participants with moderate or severe renal impairment. Lenalidomide dose adjustment should be instituted for participants with a CrCl<60 mL/minute. To be enrolled in the study, participants must have CrCl ≥30 mL/min (Section 5.1). If during treatment a participant's renal status changes, the dose should be adjusted. The recommended doses for participants with multiple myeloma and renal impairment are shown in Table 56.

Table 56: Lenalidomide Dose Modifications Due to Renal Impairment

Category	Renal Function[a]	Induction Dose (Arms A, A1, B, [redacted], and [redacted])	Maintenance Dose (Arms A, C, an[redacted])
Moderate renal impairment	CrCl 30-59 mL/min	10 mg daily	5 mg daily
Severe renal impairment	CrCl <30 mL/min (not requiring dialysis)	15 mg every 48 hours	5 mg daily D1-21 each cycle
End-stage renal disease	CrCl <30 mL/min (requiring dialysis)	5 mg[b] once daily[c]	5 mg daily D1-21 each cycle[c]

a. Estimated by creatinine clearance as calculated by the Cockcroft-Gault, modification of diet in renal disease.
b. Consider escalating dose to 15 mg daily after 2 cycles if well tolerated.
c. On dialysis days, administer dose after dialysis.

Non-Hematologic Adverse Events Associated With Lenalidomide

Dosing of lenalidomide must be interrupted due to non-hematologic AEs per Table 57; dose reductions may be considered as noted.

Table 57: Lenalidomide Dosing Interruption or Dose Reduction for Non-hematologic Adverse Events

Non-hematologic AE	Dosing Interruption	Dosing Resumption
Grade ≥3 or 4 events that do not require discontinuation (Section 7.1.4)	X	May resume lenalidomide at 1 dose level less than previous dose (see Table 50 and Table 51) at investigator discretion whenthe AE has resolved to Grade ≤2
Grade 2 skin rash	Investigator discretion	May resume lenalidomide at previous dose

Non-hematologic AE	Dosing Interruption	Dosing Resumption
Interstitial lung disease	Interstitial lung disease and related events, including cases of pneumonitis, have been observed with lenalidomide. Careful assessment of participants with an acute onset or unexplained worsening of pulmonary symptoms should be performed to exclude interstitial lung disease.<br>Dosing of lenalidomide should be interrupted pending investigation of these symptoms and if interstitial lung disease is confirmed, appropriate treatment should be initiated. Lenalidomide should only be resumed after a thorough evaluation of the benefits and the risks.	

a. See Section 6.9.2 for additional guidance regarding dose reduction and requirements for subsequent dose escalation.

6.9.4.3. Bortezomib

Treatment guidelines for bortezomib-related neuropathy are outlined in Table 58.

See also Section 6.5.6.1 for modification of administration of bortezomib in participants due to injection-site reactions.

For other Grade 3 or 4 non-hematologic AEs judged by the investigator to be related to bortezomib alone, dosing with bortezomib should be interrupted and resumed once the AE has resolved to Grade ≤2. The dose should be reduced to the next lower dose level (see Table 52). Treatment with daratumumab SC and dexamethasone may continue unless the AE toxicity meets the criteria for daratumumab SC dose delay as specified in Section 6.9.3 (hematologic AEs related to daratumumab SC) or Section 6.9.4.1 (non-hematologic AEs related to daratumumab SC).

Table 58: Treatment Guidelines for Bortezomib-related Neuropathy

Severity (per NCI-CTCAE)	Bortezomib Dosing Interruption or Dose Modification[a]
Grade 1 (asymptomatic; clinical or diagnostic observations only) with no pain or loss of function	No action
Grade 1 with pain or Grade 2 (moderate symptoms; limiting instrumental ADL)<br><br>**NOTE**: Instrumental ADL refers to preparing meals, shopping for groceries or clothes, using telephone, managing money, etc.	• Withhold bortezomib until the AE resolves or returns to baseline.<br>• Reduce bortezomib dosing by 1 level (see Table 52)<br><br>OR<br><br>Change treatment schedule to 1.3 mg/m^2 once per week
Grade 2 with pain or Grade 3 (severe symptoms; limiting self-care ADL)<br><br>**NOTE**: Self-care ADL refers to bathing; dressing and undressing; feeding self; using the toilet; taking medicinal products; and not bedridden.	Withhold bortezomib until the AE resolves<br>Bortezomib therapy may be reinitiated at a reduced dose level of 0.7 mg/m^2 and treatment schedule changed to once per week
Grade 4 (life-threatening consequences; urgent intervention indicated) and/or severe autonomic neuropathy	Permanently discontinue bortezomib

a. See Section 6.9.2 for additional guidance regarding dose reduction and requirements for subsequent dose escalation.

6.9.4.4. Dexamethasone

Table 59 summarizes management guidelines for dexamethasone-related AEs.

Table 59: Treatment Guidelines for Dexamethasone-related Adverse Events

Symptom	Findings	Recommended Action
Cardiovascular	Edema Grade ≥3 (anasarca or limiting function and unresponsive to therapy)	Diuretics as needed, and resume dexamethasone at 1 dose level lower (see Table 53); if edema persists despite above measures, decrease dose by another dose level lower.<br>Discontinue dexamethasone permanently if symptoms persist despite second reduction.
Gastrointestinal	Dyspepsia, gastric or duodenal ulcer, or gastritis Grade 1 or 2 (requiring medical management)	Continue dexamethasone at same dose and treat with therapeutic doses of H_2 blockers, or proton pump inhibitor.<br>Consider adding sucralfate or other antiulcer treatment as clinically indicated.<br>If symptoms persist despite above measures, decrease dexamethasone dose 1 dose level lower (see Table 53).
	Dyspepsia, gastric or duodenal ulcer, or gastritis Grade ≥3 (requiring hospitalization or surgery)	Hold dexamethasone until symptoms return to baseline.<br>Resume dexamethasone at 1 dose level lower along with concurrent therapy with H_2 blockers, sucralfate, or omeprazole.<br>If symptoms persist despite above measures, discontinue dexamethasone permanently.
	Acute pancreatitis	Discontinue dexamethasone permanently.
General Disorders	Limb edema Grade ≥3 (>30% limb discrepancy in volume; gross deviation from normal anatomic contour; limiting self-care activities of daily living)	Hold dexamethasone until symptoms return to baseline.<br>Diuretics as needed, and resume dexamethasone at 1 dose level lower; if edema persists despite above measures, decrease dose another dose level lower (see Table 53).<br>Discontinue dexamethasone permanently if symptoms persist despite second reduction.
Psychiatric Disorders	Confusion or mood alteration Grade ≥2 (interfering with function ± interfering with activities of daily living)	Hold dexamethasone until symptoms return to baseline.<br>Resume dexamethasone at 1 dose level lower (see Table 53).<br>If symptoms persist despite above measures, reduce by another dose level lower. If symptoms recur, discontinue dexamethasone.
Musculoskeletal	Muscle weakness Grade ≥2 (symptomatic and interfering with function ± interfering with activities of daily living)	Decrease dexamethasone by 1 dose level (see Table 53).<br>If weakness persists, decrease dose another dose level lower.<br>Discontinue dexamethasone permanently if symptoms persist.
Metabolism and Nutrition Disorders	Hyperglycemia Grade ≥3 (fasting glucose >250 mg/dL)	Withhold dexamethasone, treat with insulin or other hypoglycemic agents as needed until glucose is Grade ≤2 (<250 mg/dL) then resume dexamethasone.<br>If uncontrolled despite above measures, decrease dose by 1 dose level lower (see Table 53) until Grade ≤2 (<250 mg/dL).

Table 59: Treatment Guidelines for Dexamethasone-related Adverse Events

Symptom	Findings	Recommended Action
All Other	Other Grade ≥3 AEs felt related to dexamethasone	Hold dexamethasone dose. Resume at 1 dose level lower when the AE has resolved to Grade ≤2.<br>If the AE recurs, hold dexamethasone dose until resolution to Grade ≤2 and resume dexamethasone dose by another dose level lower.<br>If AE recurs despite 2 dose reductions, discontinue dexamethasone permanently.

Table 53 presents dose reductions guidelines for dexamethasone. If recovery from AEs is prolonged beyond 14 days, then the dose of dexamethasone will be decreased by 1 dose level when dosing is resumed.

At investigator discretion, dexamethasone may be tapered prior to complete discontinuation according to institutional practice. Dexamethasone dosing may continue even if daratumumab SC, bortezomib, or lenalidomide are interrupted within a cycle.

6.10. Continued Access to Study Treatment After the End of the Study

As end of study is defined as when all participants having completed 18 cycles of maintenance treatment with Tec-DR, Tec-D, [redacted], or [redacted], or 18 cycles of [redacted] treatment in Arm [redacted], and have been followed for an additional 6 months; access to study treatment is not available after that timepoint.

6.11. Treatment of Overdose

The highest dose of daratumumab SC in clinical studies to date is 1800 mg. The highest dose of teclistamab administered in clinical studies to date is 6 mg/kg with various dosing schedules (including monotherapy and combination studies) and the highest dose of talquetamab SC in clinical studies to date is [redacted] For any overdose involving study treatment, best supportive care measures should be administered per institutional guidelines and in ongoing consultation with the sponsor.

In the event of an overdose, the investigator or treating physician should:

- Contact the medical monitor immediately.
- Closely monitor the participant for AEs/SAEs and laboratory abnormalities for ≥3 weeks.
- Obtain a serum sample for PK analysis within 4 days of overdosed daratumumab SC and within 8 days of overdosed teclistamab or talquetamab if requested by the medical monitor (determined on a case-by-case basis).
- Document the quantity of the excess dose as well as the duration of the overdosing in the eCRF.

Overdoses of the other components of study treatment should be managed in accordance with the applicable IB/local prescribing information.

Decisions regarding dosing interruptions or dose reductions will be made by the investigator in consultation with the medical monitor based on the clinical evaluation of the participant.

6.12. Concomitant Therapy

Throughout the study, investigators may prescribe any concomitant medications or treatments deemed necessary to provide adequate supportive care except for those listed in Section 6.12.3.

During the post-transplant period (defined as the first day of high-dose melphalan administration to the first dose of maintenance therapy) only the concomitant treatments used to treat AEs specified in Section 8.1.1.3.4 will have to be recorded.

Throughout the rest of the study, all medications (including prescriptions and over-the-counter products, and transfusions of blood products) different from the study drugs must be recorded in the appropriate section of the eCRF throughout the study beginning with the signing of the ICF until 30 days after the last dose of study drug(s), or until the start of subsequent systemic antimyeloma treatment, if earlier

This includes any concomitant therapies and any medications used to treat or support AEs, SAEs, or conditions reported as medical history. Recorded information in the eCRF will include a description of the type of the drug, dose schedule, route of administration, duration of treatment, and its indication.

Modification of an effective pre-existing therapy should not be made for the explicit purpose of entering a participant into the study.

6.12.1. Permitted Therapies

Participants are to receive full supportive care during the study. The following are examples of supportive therapies that may be used:

- Standard supportive care therapies (drugs affecting bone structure and mineralization [see guidance in Section 6.12.2.3]; antiemetics; antidiarrheals; anticholinergics; antispasmodics; antipyretics; antihistamines; analgesics; antibiotics and other antimicrobials; H_2 antagonists or proton pump inhibitors; and other medications intended to treat symptoms or signs of disease) as clinically indicated, per institutional guidelines, and as deemed necessary by the investigator.

- Growth factor support, erythropoietin-stimulating agents, and transfusions are permitted to treat symptoms or signs of anemia, neutropenia, or thrombocytopenia according to local standards of care. Growth factor support should be avoided through the first 3 doses of teclistamab (0.06, 0.3, and 1.5 mg/kg) or CCI [redacted] and also during events of CRS. Also see Section 6.12.2.5 regarding monitoring for potential thromboembolic events with concomitant use of erythropoietin-stimulating agents with lenalidomide. G-CSF should be considered for Grade 3 neutropenia with infection or fever or any Grade 4 neutropenia.
- Administration of immunoglobulin replacement for hypogammaglobulinemia is strongly recommended per institutional guidelines (see Section 6.5.3).
- Prophylaxis for TLS as described in Section 6.5.7.
- Prophylactic use of antibiotics is highly recommended (see Section 6.12.2.1).
- Documented infectious complications should be treated with oral or IV antibiotics or other anti-infective agents as considered appropriate by the treating investigator for a given infectious condition per institutional guidelines.
- Corticosteroids used as pretreatment medication are permitted as described in the relevant Schedule of Activities.
- Best supportive care to prevent or treat AEs noted in Section 6.5.

6.12.2. Recommended Therapies

6.12.2.1. Infection Prophylaxis

See infection prophylaxis guidelines in Section 6.5.4.1.

- Prophylactic administration of antibiotics (eg, sulfamethoxazole 800 mg/trimethoprim 160 mg twice daily po OR amoxicillin 875 mg/clavulanic acid 125 mg twice daily po) is strongly recommended during the first 3 cycles of induction and maintenance therapy. Continuation of prophylaxis through the remaining cycles of induction and maintenance therapy is recommended in case of risk factors for infectious complications such as age, comorbidities, and neutropenia and for participants with infectious complications during the previous cycles.
- Pneumocystis carinii/jirovecii pneumonia prophylaxis as per institutional guidelines through the entire study (eg, sulfamethoxazole 800 mg/trimethoprim 160 mg po 3 days per week). If sulfamethoxazole/trimethoprim is used as antibiotic prophylaxis, no additional prophylaxis is needed for pneumocystis carinii/jirovecii pneumonia.

For COVID-19 vaccinations, see Section 6.5.4.2.

6.12.2.2. Management of HBV Reactivation

Primary antiviral prophylaxis and treatment is permitted per local SoC. See Section 6.5.4.5 for additional details on management.

6.12.2.3. Drugs Affecting Bone Structure and Mineralization

Drugs affecting bone structure and mineralization like bisphosphonate therapy or monoclonal antibodies are strongly recommended for all participants. Therapy is recommended to be continued per treatment guidelines (Moreau 2013). Commercially available IV bisphosphonates (pamidronate and zoledronic acid) are preferred when available, and should be used according to the manufacturer's recommendations, as described in the prescribing information, for participants with osteolytic or osteopenic myeloma associated bone disease. Oral bisphosphonates may be used as alternatives if IV bisphosphonates are not available at the study site. It is preferred that investigators use the same route of bisphosphonate therapy for all participants at their sites.

Participants who are using bisphosphonate therapy when they enter the study should continue the same treatment. Participants with evidence of lytic destruction of bone or with osteopenia who are not using a bisphosphonate at the time of enrollment should start a bisphosphonate as soon as possible during the first 6 to 8 weeks of study treatment. However, investigators should not start bisphosphonate therapy during the study if there are any signs of disease progression.

6.12.2.4. Prevention of Steroid-induced Gastritis

Dexamethasone and other corticosteroids may induce gastritis. Medications to prevent gastritis are permitted per institutional standards (eg, proton pump inhibitors [omeprazole or equivalent], sucralfate, or H_2 blockers [famotidine or equivalent]).

6.12.2.5. Prevention of Deep Vein Thrombosis and Pulmonary Embolism

IMiDs have been associated with increased incidence of deep vein thrombosis and pulmonary embolism (Delforge 2017). Therefore, it is strongly recommended that all participants receiving IMiDs or IMiD-based combinations be treated with prophylactic anticoagulation (Appendix 17). Risk assessment for anticoagulation and choice of drug should be guided by international guidelines and institutional practice (Palumbo 2008; Farge 2022). Vitamin K antagonists or direct thrombin inhibitors are another option for deep vein thrombosis/pulmonary embolism prophylaxis and treatment; these may be used at the discretion of the investigator. However, the use of warfarin (or other vitamin K antagonists) during Induction or Maintenance Cycle 1 is not recommended unless no other therapeutic option is available (see Section 6.12.3.1).

During Cycle 1 of the Induction Phase (Arms A, A1, B, [redacted]) and Cycle 1 of the Maintenance Phase (Arms A, C, and [redacted]) no lenalidomide is administered per protocol. During this time period, anticoagulation is generally not recommended and a decision regarding use should be made by the investigator based on individual risk assessment (eg, other underlying comorbidities, hospitalization for step-up dosing).

6.12.2.6. Mitigation Measures in Case of Positive Testing for COVID-19

In the event a participant is diagnosed with a COVID-19 infection and requires treatment or is eligible for prophylactic treatment after contact with an individual infected with COVID-19, it is recommended that the participant receives all necessary treatments for COVID-19 that are available (including those available under Emergency Use Authorization) including, but not

limited to antiviral drugs (eg, remdesivir, ritonavir, favipiravir, molnupiravir, paxlovid), monoclonal antibodies (casirivimab and imdevimab, bamlanivimab and etesevimab, tixagevimab and cilgavimab, sotrovimab), and dexamethasone.

As the prevention and treatment of COVID-19 is rapidly evolving investigators should refer to the most up to date consensus guidelines such as but not limited to NCCN or ESMO standards (see Appendix 19).

6.12.3. Prohibited or Restricted Therapies

The medications in the list below are prohibited during the study. The sponsor must be notified in advance (or as soon as possible thereafter) of any instances in which prohibited therapies are administered.

- Any chemotherapy, anticancer immunotherapy (other than drugs under this study and hormonal therapy for prostate or breast cancer), or experimental therapy
- Radiotherapy is generally prohibited for all participants during the Treatment Phase. However, emergency radiotherapy may be permitted under the following conditions:
  - Measurable soft-tissue plasmacytoma at baseline must not be in the radiation field.
  - Absence of disease progression.
  - Localized radiotherapy is permitted only for pain control or for stabilization of an extensive bone lesion at high risk of pathologic fracture or damage to surrounding tissues.
  - Radiation must not occur beyond the start of Induction Cycle 3 for participants in Arms A, A1, B, [redacted] and beyond the start of Maintenance Cycle 3 for participants in Arms C, C1, or [redacted]. However, radiation should be avoided until after the period of high risk of CRS (eg, the first 3 doses of teclistamab [0.06, 0.3, and 1.5 mg/kg] or talquetamab [redacted]
  - Approval from the sponsor is needed prior to continuation of systemic study treatment.
- Live attenuated vaccines are not permitted while on treatment and must only be administered ≥30 days after last dose of teclistamab, talquetamab, and daratumumab (see Appendix 19).
- Continuation of the study treatment after emergency orthopedic surgery is allowed only in the absence of disease progression and after consultation with and agreement by the sponsor.
- Medications used for other indications that have antimyeloma properties (eg, IFN and clarithromycin; Niesvizky 2008; Rossi 2013).
  - As an exception, clarithromycin that is prescribed for a course of ≤14 days to treat an infection for which there is no therapeutic alternative is allowed.
- Systemic corticosteroids should be avoided other than for management of AEs and pretreatment medication as specified in the relevant Schedule of Activities.
  - Corticosteroids dose regimen is not to exceed a cumulative dose of 140 mg of prednisone or equivalent for 14 days, unless administered for the management of AEs if there is not another therapeutic option.

- Other immunosuppressant agents should be avoided unless used as pretreatment medications or medication to treat an AE (eg, CRS, ICANS) or unless approved by sponsor.
- Nonsteroidal anti-inflammatory agents should be avoided to minimize the risk of exacerbation of potential sub-clinical myeloma-related kidney disease.
- The use of IV contrast infusions should be avoided to prevent myeloma-related kidney disease. If administration of IV contrast is necessary, then adequate precautions including hydration are indicated.
- Routine transfusions should not be given on dosing days for study treatment administration.
- The use of transdermal patches at the injection site should be avoided.

6.12.3.1. Prohibited and Restricted Therapies Specific to Teclistamab and Talquetamab

- CYP450 substrates with narrow therapeutic index should be used with caution from the start of step-up doses up to 7 days after the first treatment dose of teclistamab or talquetamab, as well as during any event of CRS.
- Use of warfarin (or other vitamin K antagonist) during Induction (Arms A, A1, B, [redacted] Cycle 1 of treatment with [redacted] or Maintenance Cycle 1 [redacted] is not recommended unless no other therapeutic option is available. For participants who cannot switch to a different anticoagulant and who experience CRS, coagulation parameters should be monitored closely during a CRS event and until CRS symptoms resolve.

6.12.3.2. Prohibited and Restricted Therapies Specific to Lenalidomide

- Concomitant use of erythropoietin-stimulating agents or estrogen-containing therapies with lenalidomide may increase the risk of thrombosis. If required concomitantly during treatment with lenalidomide, these drugs should be used with caution and the participant should be monitored for potential thromboembolic events.
- Concomitant use of lenalidomide may increase digoxin plasma levels. If required concomitantly during treatment with lenalidomide, digoxin plasma levels should be monitored in accordance with clinical judgment and based on standard clinical practice in patients receiving this medication during administration of lenalidomide.

6.12.3.3. Prohibited and Restricted Therapies Specific to Bortezomib (Arms B, [redacted] and [redacted]

Concomitant administration of strong CYP3A4 inducers (Appendix 20) is prohibited with the use of bortezomib. Administration of strong CYP3A4 inhibitors (eg, ketoconazole, ritonavir) should be avoided and is not recommended in participants receiving bortezomib. If a strong CYP3A4 inhibitor must be given in combination with bortezomib, monitor participants for signs of bortezomib toxicity and consider a bortezomib dose reduction. For an ongoing list of CYP3A inhibitors and inducers, see https://drug-interactions.medicine.iu.edu.

Concomitant administration of bortezomib with CYP2C19 substrates should be avoided if possible due to the potential risk of increasing exposure of these drugs. If required concomitantly during treatment with bortezomib, CYP2C19 substrates should be used with caution, and the participant monitored for potential toxicities. For an ongoing list of CYP2C19 substrates, see https://drug-interactions.medicine.iu.edu.

6.13. Subsequent Antimyeloma Therapy

Participants who discontinue all study treatment for reasons other than progressive disease must not start subsequent antimyeloma therapy unless and until disease progression is confirmed by the sponsor. If an investigator deems it is in the best interest of a participant to start subsequent antimyeloma therapy in the absence of disease progression, this decision should be discussed with the sponsor for approval prior to the start of the desired therapy.

After confirmation of disease progression, subsequent therapy is left to the investigator's discretion. All lines of subsequent antimyeloma therapy should be recorded in the eCRF. Note that SoC maintenance treatment is not considered subsequent antimyeloma therapy.

7. DISCONTINUATION OF STUDY TREATMENT AND PARTICIPANT DISCONTINUATION/WITHDRAWAL

7.1. Discontinuation of Study Treatment

Participants who discontinue treatment with any 1 or more component(s) of study treatment (teclistamab, talquetamab, daratumumab SC, lenalidomide, bortezomib [if applicable], and/or dexamethasone [if applicable]) may continue to receive treatment with the other components of study treatment, as assigned.

7.1.1. Discontinuation of All Study Treatment

All study treatment must be discontinued for any of the following:

- The participant has confirmed disease progression per IMWG criteria (Appendix 5).
- The participant becomes pregnant.
- The participant receives concurrent (non-protocol) systemic anticancer treatment. If a participant requires radiotherapy for a second primary malignancy, consultation with the sponsor is required.
- The participant has an intercurrent illness that prevents further administration of treatment.
- The participant refuses further treatment with the study treatment.
- Noncompliance with study treatment or procedure requirements.
- The participant experiences a second primary malignancy that cannot be treated by surgery alone. Participants who require radiation therapy for treatment of second primary malignancy must have study treatment discontinued unless, upon consultation with the sponsor and review of data, continuation is agreed upon.

- The investigator believes that for safety reasons or tolerability reasons (eg, AE) it is in the best interest of the participant to discontinue all study treatment.

Participants who discontinue all study treatment should continue assessments in the Follow-up Phase (see relevant Schedule of Activities).

7.1.2. Discontinuation of Teclistamab and Talquetamab

Teclistamab and talquetamab must be discontinued for any of the following:

- First event of Grade 3 CRS if unresolved in ≥48 hours.
- Second event of Grade 3 CRS or any event of Grade 4 CRS.
- Second event of Grade 3 ICANS or any event of Grade 4 ICANS.
- Second event of Grade 3 sARR or any event of Grade 4 sARR associated with administration of teclistamab or talquetamab.
- Grade ≥3 injection-site reaction associated with administration of teclistamab or talquetamab.
- The participant experiences a dosing interruption for teclistamab or talquetamab for >4 weeks (unless continuation is agreed in consultation with the sponsor after review of safety and efficacy).

7.1.3. Discontinuation of Daratumumab SC

Daratumumab SC must be discontinued for any of the following:

- Participants who experience a Grade 4 sARR associated with administration of daratumumab SC.
- Second event of Grade 3 sARR (including during re-starting of daratumumab SC administration; see Section 6.5.5.1).
- Second event of Grade ≥2 laryngeal edema or Grade ≥2 event of bronchospasm associated with administration of daratumumab SC; or
- Any Grade 2 or 3 laryngeal edema or Grade 2 or 3 event of bronchospasm associated with administration of daratumumab SC that does not respond to systemic therapy and does not resolve within 6 hours from onset.
- Grade ≥3 injection-site reaction associated with administration of daratumumab SC.
- Participants for whom dosing is interrupted for >4 weeks (Induction – Arms A, A1, B, [redacted] or >6 weeks (Maintenance with Tec-DR, Tec-D, [redacted], [redacted] should have study treatment discontinued, unless, upon consultation with the sponsor and the review of safety and efficacy, continuation is agreed upon. If the sponsor agrees to continue treatment and the treatment delay is greater than 3 months, on the first day dosing is resumed, there should be at least 3 hours between daratumumab SC administration and teclistamab or talquetamab administration, as sARRs or CRS may occur upon re-initiation of daratumumab SC, teclistamab, or talquetamab following a prolonged dosing interruption.

7.1.4. Discontinuation of Lenalidomide

Lenalidomide must be discontinued for any of the following:

- Angioedema associated with lenalidomide.
- Anaphylactic reaction associated with lenalidomide
- Grade 4 rash or any grade of exfoliative or bullous rash associated with lenalidomide. Of note, discontinuation should be considered for Grade 2 or 3 skin rash.
- Stevens-Johnson syndrome/toxic epidermal necrolysis, drug reaction with eosinophilia and systemic symptoms (DRESS), or other severe dermatologic reaction associated with lenalidomide.
- Confirmed PML
- If toxicities related to lenalidomide occur after dose reduction of lenalidomide to 5 mg (see Table 50 and Table 51) unless continuation is agreed upon in consultation with the sponsor after review of safety and efficacy.
- The participant experiences a dosing interruption for lenalidomide for >3 cycles (unless continuation is agreed in consultation with the sponsor after review of safety and efficacy).

7.1.5. Discontinuation of Bortezomib

Bortezomib must be discontinued for any of the following:

- Grade 4 (life-threatening consequences; urgent intervention indicated) and/or severe autonomic neuropathy associated with administration of bortezomib.
- If toxicities related to bortezomib occur after dose reduction of bortezomib to 0.7 mg/m^2 (see Table 52); unless continuation is agreed in consultation with the sponsor after review of safety and efficacy.

7.1.6. Discontinuation of Dexamethasone

Dexamethasone should be discontinued if toxicities related to dexamethasone occur after 2 dose reductions of dexamethasone (unless continuation is agreed in consultation with the sponsor after review of safety and efficacy). See Table 53 for guidance regarding dose reduction of dexamethasone.

7.2. Participant Discontinuation/Withdrawal From the Study

A participant will be withdrawn from the study for any of the following reasons:

- Lost to follow-up
- Withdrawal of consent

When a participant withdraws before study completion, the reason for withdrawal is to be documented in the eCRF and in the source document. If the reason for withdrawal from the study is withdrawal of consent, then no additional assessments are allowed.

Withdrawal of Consent

A participant declining to return for scheduled visits does not necessarily constitute withdrawal of consent. Alternate follow-up mechanisms that the participant agreed to when signing the consent form apply (eg, consult with family members, contacting the participant's other physicians, medical records, database searches, use of locator agencies at study completion), as local regulations permit.

7.2.1. Withdrawal From the Use of Study Samples

The participant may withdraw consent for use of study samples for research (refer to Storage, Use, Transfer, and Retention of Data and Samples in Appendix 2). In such a case, samples will be destroyed after they are no longer needed for the clinical study. Details of the sample retention for research are presented in the main ICF.

7.3. Lost to Follow-up

A participant will be considered lost to follow-up if he/she repeatedly fails to return for scheduled visits and is unable to be contacted by the study site. A participant cannot be deemed lost to follow-up until all reasonable efforts made by the study site personnel to contact the participant are deemed futile. The following actions must be taken if a participant fails to return to the study site for a required study visit:

- The study site personnel must attempt to contact the participant to reschedule the missed visit as soon as possible, to counsel the participant on the importance of maintaining the assigned visit schedule, to ascertain whether the participant wishes to or should continue in the study.
- Before a participant is deemed lost to follow-up, the investigator or designee must make every reasonable effort to regain contact with the participant (where possible, 3 telephone calls, e-mails, fax, and, if necessary, a certified letter to the participant's last known mailing address, or local equivalent methods). These contact attempts should be documented in the participant's medical records.
- Should the participant continue to be unreachable, they will be considered to have withdrawn from the study.
- Site personnel will attempt to collect the vital status of the participant within legal and ethical boundaries for all participants enrolled, including those who did not get study treatment. The site may engage a third party to search public sources for vital status information. If vital status is determined as deceased, this will be documented and the participant will not be considered lost to follow-up. Sponsor personnel will not be involved in any attempts to collect vital status information.

Should a study site close, eg, for operational, financial, or other reasons, and the investigator cannot reach the participant to inform them, their contact information will be transferred to another study site.

8. STUDY ASSESSMENTS AND PROCEDURES

8.1. Study Procedures

8.1.1. Overview

This study begins with an initial evaluation of safety for all arms (see Section 4.1).

This study is divided into 3 phases: Screening, Treatment, and Follow-up. For Arms A, A1, B, [redacted] the Treatment Phase includes induction treatment and maintenance treatment. For [redacted] or Arms C, C1, and [redacted], the Treatment Phase includes maintenance treatment.

The relevant Schedule of Activities (Section 1.3) summarizes the frequency and timing of measurements applicable to this study. Blood collections for PK and biomarker assessments should be kept as close to the specified time as possible. Other measurements may be done earlier than specified timepoints if permitted by the protocol. Actual dates and times of assessments will be recorded in the source documentation and eCRF.

Pregnancy tests are required at screening and as specified in the relevant Schedule of Activities (Section 1.3). Additional pregnancy tests may be performed, as determined necessary by the investigator or required by local regulation or local PPP program for participants treated with lenalidomide (see Appendix 9), to establish the absence of pregnancy at any time during the participation in the study. Repeat or unscheduled samples may be taken for safety reasons or for technical issues with the samples.

8.1.1.1. Screening Phase

In Arms A, A1, B, D, [redacted] the Screening Phase begins prior to induction treatment. In Arms C, C1, and [redacted] the Screening Phase begins prior to maintenance treatment, after ASCT. All participants must sign an ICF prior to the conduct of any study-related procedures. The Screening Phase begins when the ICF is signed and all screening tests should be performed within 28 days of enrollment thereafter except as specified in the relevant Schedule of Activities (Section 1.3). The following data must be collected during the Screening Phase: disease characteristics (tumor type and histology); date of diagnosis; cancer stage; available pathology, and molecular data (see Section 1.3 and Section 8.2). In addition, for Arms C, C1, and [redacted] prior antimyeloma therapies should be collected (see Section 1.3).

See Section 5 for details about changes to eligibility during screening (ie, before enrollment) and before the start of administration of study treatment.

8.1.1.2. Treatment Phase

The Treatment Phase begins with the start of administration of study treatment (induction treatment in Arms A, A1, B, [redacted], and maintenance treatment in Arms C, C1, and [redacted]) and continues until the completion of the EOT Visit. AEs (related to any of the study drugs, as well as certain unrelated AEs) will be managed as described throughout the protocol, including safety monitoring as described in Section 6.4. Study treatment may be administered for 18 cycles of maintenance, or (for Arm [redacted]) 18 cycles of Tec-Tal, or until confirmed progressive disease, death, intolerable toxicity (see discontinuation criteria in Section 7.1), withdrawal of consent, or end of the study, whichever occurs first. Tec-DR, Tec-D, [redacted], or [redacted] study maintenance treatment, or [redacted] study treatment, can be discontinued when 12 months of sustained MRD negativity has been observed during the study. Upon discontinuation of study treatment, the participant will complete an EOT Visit.

8.1.1.2.1. Induction Treatment (Arms A, A1, B, [redacted] Only)

- Participants in Arms A, A1, [redacted] will receive six 28-day cycles of Tec-DRd induction therapy (Induction Cycles 1-6) as described in Section 6.1.
- Participants in Arm B will receive six 28-day cycles of Tec-DVRd induction therapy (Induction Cycles 1-6) as described in Section 6.1.
- [redacted]
- [redacted]

Efficacy will be assessed as per the Schedule of Activities Table 3 and Table 4.

8.1.1.2.1.1. End of Induction Treatment

Within 2 weeks of completing Induction Cycle 6, and [redacted] prior to [redacted] participants in Arms A, A1, B, [redacted] will be assessed as per the Schedule of Activities Table 3 and Table 4.

8.1.1.3. Stem Cell Collection, HDT, and ASCT (as per SoC)

8.1.1.3.1. Mobilization and Harvesting of Stem Cells

Stem cell mobilization should be performed after Induction Cycle 3 per local SoC. Sites should be prepared to utilize Plerixafor in addition to standard agents such as cyclophosphamide and G-CSF to ensure adequate mobilization. Additional use of Plerixafor is recommended if there is a suspicion of inadequate mobilization. The use of a second mobilization as per local SoC or alternatively a bone marrow harvest should occur to ensure adequate stem cell yield as per institutional practice if the stem cell yield is deemed to be suboptimal per investigator discretion. It is recommended to collect at least 2.5×10^6 $CD34^+$ cells/kg body weight but ideally at least 5×10^6 $CD34^+$ cells/kg body weight should be collected. An assessment of the efficiency of mobilization/harvesting will be recorded in the eCRF (Section 8.2).

[redacted]. However, HDT+ASCT will be deferred in these participants and they will not undergo the steps described below (Section 8.1.1.3.2, Section 8.1.1.3.3, and Section 8.1.1.3.4); these participants will instead receive [redacted] treatment as described in Section 8.1.1.4.

8.1.1.3.2. Conditioning (Melphalan)

Melphalan 200 mg/m^2 therapy prior to ASCT is recommended as conditioning therapy. Melphalan may be given at a lower dose of 140 mg/m^2, per institutional standards (ie, renal insufficiency).

8.1.1.3.3. Transplant

There should be no more than 12 weeks between end of induction and transplant. Participants should have a single re-infusion of stem cells 24 to 48 hours after high-dose melphalan.

8.1.1.3.4. Engraftment/Recovery

Participants will be monitored for successful engraftment by means of hematopoietic reconstitution (defined as absolute neutrophil count ≥0.5 x 10^9/L and platelet count ≥20 x 10^9/L). Supportive therapy will be administered according to institutional standards.

During the immediate post-transplant period (defined as the first day of high-dose melphalan administration to the first dose of maintenance therapy), only the following AEs, and concomitant medications and procedures associated with these AEs, have to be recorded in the eCRF:

- Any evolution of an ongoing AE at the time of ASCT
- Any new AE related, or that appears to be related, to Tec-DRd, Tec-DVRd, [redacted]

8.1.1.4. [redacted]

[redacted]

[redacted]

8.1.1.5. Maintenance Treatment

Participants will receive a maximum of eighteen 28-day cycles of maintenance therapy (Maintenance Cycles 1-18) with Tec-DR (Arms A and C), Tec-D (Arms A, A1, B, C, [redacted] and [if opened] Arms C1 and [redacted], [redacted] (Arm [redacted]), [redacted] as described in Section 6.1. For Arms A, A1, B, [redacted] maintenance therapy should commence within 180 days of transplant when engraftment is complete (defined as ANC ≥1.0 x 10^9/L and platelet count ≥75 x 10^9/L) and when in the opinion of the investigator the participant is fit enough to tolerate maintenance therapy. Initiation of maintenance treatment in Arms C, C1, and [redacted] will be in line with inclusion criteria (see Section 5.1). Efficacy will be assessed as per the Schedule of Activities Table 6. Tec-DR, Tec-D, [redacted] study maintenance treatment can be discontinued when 12 months of sustained MRD negativity has been observed during the study.

8.1.1.5.1. EOT Visit

The EOT Visit is required for all participants, including those discontinuing treatment for any reason except being lost to follow-up, death, or withdrawal of consent for study participation. This visit will be completed up to 30 days (+7 days) after the last dose of study treatment (Tec-DRd, Tec-DVRd, [redacted] induction treatment if not continued on maintenance or [in Arm D] [redacted] or before the start of a new antimyeloma therapy, whichever comes first.

If a participant is unable to return to the site for the EOT Visit, or if the EOT Visit occurs before Day 30 after the last dose of study treatment, the participant should be contacted to collect AEs that occur up to 30 days after the last dose of study treatment or until the start of a subsequent antimyeloma therapy, whichever comes first. The primary reason for treatment discontinuation will be documented in the eCRF.

8.1.1.6. Follow-up Phase

The Follow-up Phase starts after the EOT Visit and will continue until death, withdrawal of consent, loss to follow-up, or end of the study, whichever occurs first. If the participant has died, the date and cause of death will be collected and documented on the eCRF, if or when available. AEs will be followed for 30 days after the last dose of study treatment (Tec-DRd, Tec-DVRd, [redacted] maintenance) or longer if required (see follow-up for SAEs in Section 8.4.3); see also Section 8.3.5.3 for follow-up related to the risk of HBV reactivation associated with study treatment and continued monitoring required for 6 months after the last dose of teclistamab, talquetamab, or daratumumab SC.

If study treatment is discontinued prior to the onset of disease progression per IMWG criteria (Appendix 5), disease evaluation should continue to be performed until confirmed disease progression, death, start of subsequent antimyeloma therapy (unless disease progression is not confirmed prior to start of such therapy; see Section 6.13), withdrawal of consent, or end of the study, whichever occurs first. Although subsequent antimyeloma therapy should not start until after disease progression (see Section 6.13), if progressive disease is not confirmed before the start of a new antimyeloma regimen, disease evaluation should continue for up to 30 days after the start of subsequent therapy for purpose of confirmation of progression only as indicated in the relevant Schedule of Activities (Section 1.3).

Subsequent antimyeloma treatment and response to treatment including date of subsequent progression (ie, PFS2) will be recorded and survival status will be obtained.

If the information is obtained via telephone contact, written documentation of the communication must be available for review in the source documents. Where allowed by local law, public records may be used to document death to obtain survival status.

8.1.1.6.1. SoC Maintenance

During the Follow-up Phase, SoC maintenance treatment, per institutional standard and local investigator decision, is permitted. If SoC maintenance treatment is initiated, the treatment regimen should be documented. If the SoC treatment is discontinued, the reason for discontinuation should be documented. Changes in SoC maintenance should also be documented. If decided to be in the best interest of the participant by the local investigator, the participant may also be observed only after the end of study maintenance treatment (Tec-DR, Tec-D, CCI [redacted]). For drugs prescribed as SoC maintenance treatment after EOT, safety reporting will follow practices used for routine care. Only events related to study drugs should be reported.

8.1.2. Local Laboratory Assessments

All efforts should be made to collect efficacy data centrally. However, local laboratory data may be collected if central laboratory data are not available at a particular timepoint. See Section 5.1 for details of disease assessment using local laboratory at screening. Documentation of the local laboratory data should be sent to the principal investigator and filed in the medical record. It is the principal investigator's responsibility to ascertain that these results are reviewed and entered into the participant's medical record and the eCRF.

8.1.3. Sample Collection and Handling

The actual dates and times of sample collection must be recorded in the eCRF or laboratory requisition form. Instructions for the collection, handling, storage, and shipment of samples are found in the Laboratory Manual that will be provided. Collection, handling, storage, and shipment of samples must be under the specified, and where applicable, controlled temperature conditions as indicated in the Laboratory Manual.

8.1.4. Study-Specific Materials

The investigator will be provided with the following supplies:

- Study protocol
- IBs for teclistamab and talquetamab
- Local Prescribing Information: daratumumab SC, lenalidomide, bortezomib, and dexamethasone
- IPPI(s) or equivalent documentation for teclistamab, talquetamab, and daratumumab SC
- SIPPM
- Laboratory Manual
- IWRS manual
- eDC manual
- eCRF completion guidelines
- ICF

- Diary card for recording of daily temperature as noted in Section 6.4.1.1
- Ancillary supplies for study treatment preparation and administration.

8.1.5. Home Health Care and Telehealth Visits

In exceptional circumstances, home health care and telehealth visits may be implemented by or with approval from the sponsor and per the clinical judgment of the investigator, where feasible and permissible by local policy and regulations. Participants for whom there is no safety concern related to administration of study drug(s) or any pre-existing condition(s) may have home health care and telehealth (conducted via phone or video conference) visits.

Study procedures such as participant reconsenting; ECOG assessment; AE and concomitant medication reporting; review of body systems; and collection of information on the participant's current health status may be performed with home health care and telehealth visits. Protocol-specified laboratory assessments for efficacy and safety may be collected during home health care visits. All assessments should be followed with in-person examination, as applicable.

If local laboratories are used, it is important to ensure appropriate documentation of laboratory reference ranges. Source documentation and if applicable the appropriate eCRFs should be completed and detail how each assessment was collected (eg, remote vs on-site, central vs local laboratory, vital signs taken at home by delegated in-home nursing).

8.2. Efficacy Assessments

Disease evaluations must be performed by the central laboratory Day 1 of every cycle during the Treatment Phase for all treatment regimens, as specified in the relevant Schedule of Activities. Local laboratory assessments may be used under specified circumstances (Section 8.1.2). If both central and local assessments are performed on the same day, central laboratory results will be prioritized. Response assessments will be based on IMWG criteria (Appendix 5).

A validated computer algorithm will be used to analyze response to treatment. See Section 8.1.1.6 for details regarding disease evaluation during the Follow-up Phase.

The investigational sites are requested to notify the sponsor if a participant is diagnosed with disease progression (which must be confirmed with a consecutive assessment ≥1 calendar day apart if based on M-protein/serum FLC levels) and provide documentation of disease progression. (eg, by completing a disease progression form or by contacting the IWRS). The sponsor's medical monitor must review the data provided to confirm that IMWG criteria for progressive disease have been met. If disease progression is confirmed by sponsor, then the participant will discontinue study treatment, complete the EOT Visit, and enter the Follow-up Phase. If disease progression has not occurred at the time of the EOT Visit, disease evaluations must continue until disease progression is confirmed. Subsequent antimyeloma treatment will not be started until after disease progression is confirmed by the sponsor.

8.2.1. M-protein Measurements in Serum and Urine

Blood and 24-hour urine samples for M-protein measurements will be sent to and analyzed by a central laboratory. If results from both central and local laboratory for the same date are obtained, the former values should be used for the assessment of response.

The following assessments are required to be performed per the relevant Schedule of Activities:

- Albumin
- Serum β2-microglobulin
- Quantitative immunoglobulins
- SPEP
- 24-hour UPEP
- Serum FLC assay (note that timing differs by whether disease is measurable by light chain or degree of response)
- SIFE/UIFE.

Blood and 24-hour urine samples will be collected as specified in the relevant Schedule of Activities until the development of confirmed disease progression (see Section 8.1.1.6 for guidance regarding disease evaluations in the event that subsequent antimyeloma therapy is begun prior to confirmation of disease progression). Disease progression based on 1 of the laboratory tests alone must be confirmed by ≥1 repeat investigation. Disease evaluations will continue beyond relapse from CR until disease progression is confirmed.

8.2.1.1. Potential for Daratumumab Interference with Disease Evaluations

Daratumumab is a human IgG1κ monoclonal antibody that may be detected on SPEP and SIFE assays used for monitoring disease monoclonal immunoglobulins (M-protein). This can lead to false positive SPEP and SIFE assay results for participants with IgG kappa myeloma protein and affect assessments of responses.

Therefore, a daratumumab-specific IFE will be performed when daratumumab interference is suspected based on SPEP and SIFE results. This reflex assay relies on the use of a daratumumab-specific murine anti-idiotype antibody that binds and shifts daratumumab's migration pattern during electrophoresis, thus distinguishing daratumumab from the endogenous myeloma M-protein (McCudden 2016). The daratumumab-specific IFE will be performed at a central laboratory to confirm a VGPR or better in participants with IgGκ myeloma when daratumumab interference is suspected based on SPEP and SIFE results.

8.2.2. Bone Marrow Examination

Bone marrow aspirate and core biopsy will be performed for clinical assessments, MRD, and biomarker evaluations (see relevant Schedule of Activities and Table 60). Clinical staging (morphology, immunohistochemistry, immunofluorescence, or flow cytometry) should be performed as described in Table 60. Cytogenetics evaluation by FISH markers, including del17p, t[4;14]), t[14;16]), del13q and amplification of 1q21, will be done at a central laboratory. MRD will be evaluated at a central laboratory. Bone marrow samples may also be evaluated for other markers of pharmacological activity and predictor of response, including immune cell infiltration, markers of immune-cell activation and/or exhaustion, and expression of BCMA.

Table 60: Bone Marrow Testing – All Arms

Timepoint[a]	Notes	Local Testing	Central Testing
Screening	• See notes for bone marrow aspirate and core biopsy in Table 2.<br>• IHC/IF (both require kappa/lambda ratio from analysis of >100 plasma cells) or 2- to 8-color flow cytometry are acceptable methods to evaluate plasma cell clonality.	Evaluate BMPC%	FISH analysis[a]<br>MRD by NGF<br>Immunophenotyping and molecular markers<br>Evaluate clonality of BMPCs by flow cytometry or IHC/IF[b]
Arms A, A1, B, CCI [redacted] only:<br>After completion of Induction Cycle 3 (before mobilization procedures) and then after completion of Induction Cycle 6 in all participants	• IHC/IF (both require kappa/lambda ratio from analysis of >100 plasma cells) or 2- to 8-color flow cytometry are acceptable methods to evaluate plasma cell clonality.	Evaluate BMPC%	MRD by NGF after completion of Induction Cycle 3 (before mobilization procedures) and in all participants after completion of Induction Cycle 6<br>Immunophenotyping and molecular markers<br>Evaluate clonality of BMPCs by flow cytometry or IHC/IF[b]
After completion of HDT+ASCT and prior to maintenance	• IHC/IF (both require kappa/lambda ratio from analysis of >100 plasma cells) or 2- to 8-color flow cytometry are acceptable methods to evaluate plasma cell clonality.	Evaluate BMPC%	MRD by NGF<br>Immunophenotyping and molecular markers<br>Evaluate clonality of BMPCs by flow cytometry or IHC/IF[b]
For participants who have not progressed and remain on study, at 6, 12, 18, and 24 months (±1 month) post start of maintenance	• IHC/IF (both require kappa/lambda ratio from analysis of >100 plasma cells) or 2- to 8-color flow cytometry are acceptable methods to evaluate plasma cell clonality.	Evaluate BMPC%	MRD by NGF<br>Immunophenotyping and molecular markers<br>Evaluate clonality of BMPCs by flow cytometry or IHC/IF[b]
Time of suspected CR (including sCR)	• If these time points occur within 1 month of another bone marrow aspiration, a repeat bone marrow aspiration will not be requested.<br>• IHC/IF (both require kappa/lambda ratio from analysis of >100 plasma cells) or 2- to 8-color flow cytometry are acceptable methods to evaluate plasma cell clonality.	Evaluate BMPC% to confirm CR	MRD by NGF<br>Immunophenotyping and molecular markers<br>Evaluate clonality of BMPCs by flow cytometry or IHC/IF to confirm sCR[b]
Time of disease progression		Evaluate BMPC%	Immunophenotyping and molecular markers<br>Evaluate clonality of BMPCs by flow cytometry or IHC/IF[b]

NOTE: Samples collected outside of the specified window will be retained for potential analysis.

a. If central FISH analysis fails, results of local FISH analysis can be provided, if available

b. If central testing is not available, local testing will be allowed.

8.2.3. Minimal Residual Disease Evaluations

Collection of bone marrow aspirates are required to monitor MRD in all participants as specified in the relevant Schedule of Activities. MRD will be evaluated by NGF (EuroFlow). Bone marrow aspirate should be sent to central laboratory for MRD assessments. Alternative methods of MRD assessment may be additionally explored.

See additional details for biomarker-related evaluations of MRD in Section 8.7.2.

8.2.4. Imaging for Disease Evaluation

Imaging must be performed at screening and thereafter as clinically indicated. Imaging will be interpreted locally. Any of the following modalities are acceptable but must include imaging of skull, entire vertebral column, pelvis, chest, humeri, femora, and any other bones for which the investigator suspects involvement by disease:

- Whole-body MRI
- Low-dose whole-body CT
- PET-CT with diagnostic CT component. If a CT scan is used, it must be of diagnostic quality (see the disease response criteria in Appendix 5).

The modality used for screening should be maintained for any subsequent imaging procedures and, if indicated, to evaluate for possible disease progression.

In addition, following study treatment, and before disease progression is confirmed, imaging should be performed locally, whenever clinically indicated based on symptoms, to document response or disease progression. If a participant presents with disease progression manifested by symptoms of pain due to bone changes, then disease progression may be documented by skeletal survey or other radiographs, depending on the symptoms that the participant experiences. If the diagnosis of disease progression is obvious by radiographic investigations, then no repeat confirmatory imaging is necessary. If changes are equivocal, then a repeat imaging is needed in 1 to 3 weeks.

The modality used for imaging must be indicated on the eCRF. If cross-sectional imaging was obtained, the report must be made available to sponsor for review upon request.

8.2.4.1. Imaging at Time of MRD Assessments

Optional for sites where available, a DW-MRI scan should be obtained at baseline; after 6 cycles of induction treatment for Arms A, A1, B, CCI [redacted]; at start of maintenance treatment CCI [redacted]

8.2.5. Documentation of Soft-Tissue Plasmacytomas

There are 2 types of soft-tissue plasmacytoma in multiple myeloma: 1) extramedullary plasmacytoma from metastatic or hematogenous spread involving only soft tissues and 2) paraskeletal plasmacytomas with tumor masses arising from skeletal lesions (Rosinol 2021). Instructions in this section apply to both extramedullary and paraskeletal soft-tissue plasmacytomas.

Soft-tissue plasmacytomas must be assessed and documented by clinical examination or radiologic imaging for all participants during Screening. Clinical examination, MRI, or CT with IV contrast (only if there is no contraindication to the use of IV contrast) may be used to document the presence and size of soft-tissue plasmacytomas. PET scan or ultrasound are not acceptable methods. However, PET/CT scans may be used to document the presence and size of soft-tissue plasmacytomas if the CT component of the PET/CT scan is of sufficient diagnostic quality.

For as long as a plasmacytoma remains measurable on physical examination, it will be evaluated (presence and size) every 4 weeks (±1 week) and continue until development of confirmed CR or confirmed disease progression or start of subsequent antimyeloma therapy. For plasmacytomas only detectable by radiologic imaging, then the evaluation of such plasmacytomas should be performed every 12 weeks (±14 days) from C1D1 until the plasmacytoma(s) meet CR criteria or confirmed disease progression or start of subsequent antimyeloma therapy. The methodology used for evaluation of each plasmacytoma should be consistent across all visits. Irradiated or excised lesions will be considered "not measurable" and will be monitored only for disease progression.

If biopsy of soft-tissue plasmacytoma is performed for any reason, a sample should be sent to the central laboratory. See Appendix 5 for additional information regarding soft-tissue plasmacytomas and disease response.

8.2.6. Patient-reported Outcomes

PRO data will be captured to assess the changes in symptoms, functioning, and overall HRQoL with treatment regimens using the following standard PRO instruments:

CCI [redacted]

During the Treatment Phase, standard PRO instruments should be completed by the participant within 2 days prior to dosing before any clinical tests, procedures, or other consultations that would influence their perceptions of their current health state. Additionally:

- The PRO instruments will be provided in the local language in accordance with local guidelines.

- The PRO instruments will be available for regulators and for IRB/IEC submissions and will be provided separately in a companion manual with the instruments that will be submitted with the protocol.
- The PRO and AE data will not be reconciled with one another.

For PRO assessments conducted beyond disease progression or subsequent antimyeloma therapy, if no site visits are scheduled for additional disease evaluations, participants may complete the PRO assessments via telephone.

8.2.6.1. [redacted]

[redacted]

8.2.6.2. [redacted]

[redacted]

8.2.6.3. [redacted]

[redacted]

8.3. Safety Assessments

Safety will be measured by AEs, laboratory test results, vital sign measurements, physical examination findings, assessment of ECOG performance status grade, and ICE score. Any clinically relevant changes occurring during the study must be recorded on the Adverse Event section of the eCRF. Any clinically significant abnormalities persisting at the end of the study/early withdrawal will be followed by the investigator until resolution or until a clinically stable condition is reached.

Details regarding the Study Safety Committee and the Study Steering Committee are provided in Appendix 2.

8.3.1. Physical Examinations

Medical history, including but not limited to all ongoing relevant and clinically significant comorbidities according to NCI-CTCAE Version 5.0, including severity grade, will be recorded during screening. Clinically significant postbaseline abnormalities should be recorded as AEs.

A complete physical examination will be conducted at screening. Symptom-directed physical examinations will be conducted thereafter.

Body weight will be measured at screening and the beginning of each cycle.

8.3.2. Vital Signs

Vital signs (temperature, pulse/heart rate, respiratory rate, blood pressure) and oxygen saturation will be assessed. Blood pressure and pulse/heart rate measurements should be preceded by ≥5 minutes of rest in a quiet setting without distractions (eg, television, cell phones). Blood pressure and pulse/heart rate measurements will be assessed with a completely automated device. Manual techniques will be used only if an automated device is not available.

Any additional vital signs assessments supporting the start and end dates of an AE (eg, fever, hypertension) should be reported in the eCRF.

8.3.3. Electrocardiograms

During the collection of ECGs, participants should be in a quiet setting without distractions (eg, television, cell phones). Participants should rest in a supine position for ≥5 minutes before ECG collection and should refrain from talking or moving arms or legs.

8.3.4. Clinical Safety Laboratory Assessments

Blood samples for serum chemistry and hematology will be collected as noted in Appendix 18, Clinical Laboratory Tests. The investigator must review the laboratory results, document this review, and record any clinically relevant changes occurring during the study in the AE section of the CRF. The laboratory reports must be filed with the source documents.

Any additional chemistry and hematologic laboratory assessment supporting the start and end dates of an AE should be reported in the eCRF.

8.3.5. Hepatitis Testing

8.3.5.1. Hepatitis B Virus Testing

HBV serology is not required at screening if this was performed as part of SoC within 3 months prior to the start of administration of study treatment. The HBV screening guide in Table 61 will be used to determine participant eligibility for the study.

Table 61: Eligibility Related to Hepatitis B Test Results

Action	Hepatitis B Test Result			
	HBsAg	anti-HBs	anti-HBc[a]	HBV-DNA[b]
Exclude	Participants who are HBsAg-positive or HBV-DNA positive are excluded from the study regardless of the status of anti-HBs and anti-HBc.			
Include	Negative	Negative	Negative	Not required[c]
	Negative	Positive	Positive	Negative
	Negative	Negative	Positive	Negative
	Negative	Positive	Negative	Negative[d]

a. Total antibody (not only IgM) should be measured
b. In participants with negative HBsAg test, an HBV-DNA quantification test is required in the following participants to determine eligibility:
   a. Participants who are anti-HBs positive and without history of vaccination
   b. Participants with positive anti-HBc and either positive or negative anti-HBs.
c. Participants with a known history of HBV irrespective of serology results are required to have an HBV-DNA quantification test for eligibility
d. Participants with serologic findings suggestive of HBV vaccination (ie, anti-HBs positivity as the only serologic marker) and a known history of prior HBV vaccination do not need to be tested for HBV DNA by PCR.

8.3.5.2. Hepatitis C Virus Testing

HCV antibody testing is not required at screening if this was performed as part of SoC within 3 months prior to the start of administration of study treatment. Participants with a history of HCV antibody positivity must undergo HCV-RNA testing.

8.3.5.3. Ongoing Hepatitis B Virus and Hepatitis C Virus Testing

During and following study treatment, participants who have history of HBV infection (eg, anti-HBc-positive irrespective of anti-HBs status anti-HBs-positive, and unknown HBV vaccination history, or known history of prior HBV infection irrespective of Hep B serology findings) will be closely monitored for clinical and laboratory signs (including DNA PCR) of reactivation of HBV every 12 weeks (±4 weeks) up to 6 months after the last dose of teclistamab, talquetamab, or daratumumab SC and as clinically indicated. Where required by local law, the results of HBV testing may be reported to the local health authorities. For HBV reactivation see Section 6.5.4.5.

Participants with a history of HCV antibody positivity will be monitored for HCV-RNA testing every 12 weeks (±4 weeks) up to 6 months after the last dose of teclistamab, talquetamab, or daratumumab SC. For HCV reactivation, see Section 6.5.4.6.

8.3.6. Pregnancy Testing

See relevant Schedule of Activities for timing of pregnancy testing.

A serum pregnancy test will be done at screening in participants of childbearing potential in all arms. In Arms A, A1, B, [redacted], a urine pregnancy test will also be done within 24 hours of Induction Cycle 1 Day 1; 10 to 14 days prior, as well as 24 hours prior, to the first dose of lenalidomide; weekly for the first 4 weeks of lenalidomide treatment; and thereafter every 4 weeks (every 2 weeks for participants with irregular menses). In all arms, a urine pregnancy test will also be done within 24 hours of Maintenance Cycle 1 Day 1 or within 24 hours of Cycle 1 Day 1 of [redacted] treatment; 10 to 14 days prior, as well as 24 hours prior, to the first dose of lenalidomide (if applicable); weekly for the first 4 weeks of lenalidomide treatment (if applicable); and thereafter every 4 weeks (every 2 weeks for participants with irregular menses).

Additional urine pregnancy tests may be performed, as determined necessary by the investigator or required by local regulation, to establish the absence of pregnancy at any time during the participation in the study.

Investigators should follow the local label for guidance on participant education/counseling and ensure that all participants treated with lenalidomide adhere to the lenalidomide local PPP program as described in Appendix 9.

8.3.7. Neurological Examination

The ICE tool evaluation presented in Appendix 15 will be performed on Cycle 1 Day 1 and on the same day as step-up dose 1 (Cycle 1 Day 2) prior to administration of pretreatment medication for teclistamab or talquetamab in both induction and maintenance to establish baseline neurologic status and during the Treatment Phase if ICANS is suspected. Appendix 16 details the ICE score(s) associated with each severity grade of ICANS and includes additional assessments that should be performed to assess severity per the ASTCT grading scale.

[redacted]

8.3.8. ECOG Performance Status

The ECOG performance status scale will be used to grade changes in the participant's activities of daily living (see Appendix 6, which also includes the conversion from Karnofsky Performance Status).

8.4. Adverse Events, Serious Adverse Events, and Other Safety Reporting

Timely, accurate, and complete reporting and analysis of safety information, including AEs, SAEs, and PQC, from clinical studies are crucial for the protection of participants, investigators, and the sponsor, and are mandated by regulatory agencies worldwide. The sponsor has established Standard Operating Procedures in conformity with regulatory requirements worldwide to ensure

appropriate reporting of safety information; all clinical studies conducted by the sponsor or its affiliates will be conducted in accordance with those procedures.

AEs will be reported by the participant (or, when appropriate, by a caregiver, surrogate) as noted in the relevant Schedule of Activities.

Further details on AEs, SAEs, and PQC can be found in Appendix 3.

8.4.1. Time Period and Frequency for Collecting Adverse Event and Serious Adverse Event Information

All AEs, except CRS and ICANS, will be graded per NCI-CTCAE Version 5.0 (see Appendix 3, Section 10.3.3). CRS and ICANS will be graded based on the ASTCT guidelines (Lee 2019; see Appendix 14 and Appendix 16 respectively). Symptoms of CRS and ICANS will be graded according to NCI-CTCAE Version 5.0 and also reported in eCRF. See also supplementary information for grading of sARRs in Table 41.

All AEs

All AEs (with the exception of progression of multiple myeloma) and special reporting situations, whether serious or non-serious, will be reported from the time a signed and dated ICF is obtained until 30 days after the last dose of study treatment or until the start of subsequent antimyeloma therapy, if earlier, which may include contact for follow-up of safety. Any AEs occurring more than 30 days after the last dose of study treatment considered to be related to study treatment must be reported to the sponsor. AEs will be followed by the investigator as specified in Appendix 3.

Participants with Grade 3 or higher AEs or unresolved AEs that lead to study drug(s) discontinuation will continue to be assessed until recovery to Grade ≤1 or baseline, the event is deemed irreversible, the participant discontinues the study, or a maximum of 6 months, whichever comes first.

SAEs

All SAEs, as well as PQC, occurring during the study must be reported to the appropriate sponsor contact person by study site personnel immediately, but no later than 24 hours of their knowledge of the event.

SAEs, including those spontaneously reported to the investigator occurring within 30 days after the last dose of study treatment, or until the start of subsequent antimyeloma therapy if earlier, must be reported. The sponsor will evaluate any safety information that is spontaneously reported by an investigator beyond the time frame specified in the protocol.

Information regarding SAEs will be transmitted to the sponsor using the SAE Form and Safety Report Form of the eCRF, which must be completed and reviewed by a physician from the study site, and transmitted to the sponsor immediately but no later than 24 hours of their knowledge of the event. The initial and follow-up reports of an SAE should be transmitted by one of the following acceptable and secure methods:

- Safety Gateway or comparable system.
- Electronically via sponsor SECURE email service.
- Electronically via sponsor-approved secure application (eg, Graphnet).
- Telephone reporting should be the exception and the reporter should be asked to complete the appropriate form(s) first. If this will jeopardize the reporting timelines, the sponsor representative should complete the SAE Form Cover Page and ensure that it contains minimal information for a reportable SAE, including a statement that it concerns a telephone report. The SAE Form Cover Page will be forwarded as defined in the safety management plan and transmitted to the report with the instruction to complete the appropriate form(s) as soon as possible.
- Facsimile (fax), receipt of which is evidenced in a successful fax transmission report.

8.4.2. Method of Detecting Adverse Events and Serious Adverse Events

Care will be taken not to introduce bias when detecting AEs or SAEs. Open-ended and nonleading verbal questioning of the participant is the preferred method to inquire about AE occurrence.

Solicited Adverse Events

Solicited AEs are predefined local and systemic events for which the participant is specifically questioned.

Unsolicited Adverse Events

Unsolicited AEs are all AEs for which the participant is not specifically questioned.

8.4.3. Follow-up of Adverse Events and Serious Adverse Events

The investigator is obligated to perform or arrange for the conduct of supplemental measurements and evaluations as medically indicated to elucidate the nature and causality of the AE, SAE, or PQC as fully as possible. This may include additional laboratory tests or investigations, histopathological examinations, or consultation with other health care professionals.

AEs, including pregnancy, will be followed by the investigator as specified in Appendix 3.

8.4.4. Regulatory Reporting Requirements for Serious Adverse Events

The sponsor assumes responsibility for appropriate reporting of the Safety Information to the Regulatory Authorities/IECs/IRBs in each respective country/territory, as applicable.

8.4.5. Pregnancy

All initial reports of pregnancy in participants or their partners (through sperm of participant/from sexual intercourse) must be made to the sponsor by the study site personnel within 24 hours of their knowledge of the event using the appropriate pregnancy notification form. Abnormal pregnancy outcomes (eg, spontaneous abortion, fetal death, stillbirth, congenital anomalies, ectopic pregnancy) are considered SAEs and must be reported using an SAE reporting form. Any participant who becomes pregnant during the study must promptly discontinue further study treatment.

Investigators should follow the local label for guidance on participant education/counseling and ensure that all participants treated with lenalidomide adhere to the local PPP as described in Appendix 9.

8.4.6. Disease-Related Events and Disease-Related Outcomes Not Qualifying as Adverse Events or Serious Adverse Events

All events that meet the definition of an SAE will be reported as SAEs, regardless of whether they are protocol-specific assessments.

Expected progression of disease, which is part of the natural course of the disease under study, should not be considered or reported as an adverse event (or serious adverse event).

Death or hospitalization that is attributed by the investigator to progression of disease should not be considered nor reported as an adverse event (or serious adverse event).

Of note, worsening of disease (and associated hospitalization or death) determined by the investigator to be caused by the study treatment should be reported per the usual reporting requirements (refer to Adverse Event Definitions and Classifications in Appendix 3).

Progression of disease and death due to disease progression should be documented on the appropriate eCRF forms (eg, the Disease Progression form and the Death form).

Signs or symptoms of disease progression that are of clinical significance, such as spinal cord compression, vena cava superior syndrome, major vessel rupture, efflux obstruction or organ failure, should be documented on the appropriate eCRF forms (eg, the Symptomatic Progression Form).

Additionally, the following should not be considered nor reported as an SAE:

- Hospitalization required by the protocol for safety monitoring for teclistamab and talquetamab (Section 6.4.1 and Section 6.4.1.2).
- Hospitalization per the protocol for safety monitoring for daratumumab SC (Section 6.3).

8.4.7. Adverse Events of Special Interest

In this study, Grade ≥3 CRS, Grade ≥3 ICANS, and Grade ≥3 non-ICANS teclistamab- or talquetamab-related neurotoxicity are considered AESIs. Therefore, as part of standard safety monitoring activities by the sponsor, any occurrence of Grade ≥3 CRS, Grade ≥3 ICANS, or Grade ≥3 non-ICANS teclistamab- or talquetamab-related neurotoxicity must be followed until resolution. All AESIs will be reported to the sponsor within 24 hours of awareness of the event. Serious AESIs will be reported via standard SAE reporting while non-serious AESIs will be reported solely via the eCRF to facilitate sponsor follow-up if needed.

8.5. Pharmacokinetics and Immunogenicity

8.5.1. Evaluations

Serum from venous blood samples will be collected for measurement of concentrations of teclistamab, talquetamab, and daratumumab (maintenance treatment only) and the generation of ADAs (where applicable) to teclistamab, talquetamab, and daratumumab (maintenance treatment only) per the relevant Schedule of Activities. Note that an additional PK/immunogenicity sample will be collected as follows:

- Suspected sARR Grade ≥2 (if related to daratumumab, only collect if associated with the second dose or later).
- Suspected CRS or teclistamab-related or talquetamab-related neurotoxicity event Grade ≥2 (as soon as CRS is detected, if feasible).

On days where both PK and immunogenicity of either drug (teclistamab, talquetamab, or daratumumab) are specified, serum aliquots from the same blood collection will be utilized. The exact dates and times of blood sampling must be recorded on the laboratory requisition form.

Samples collected for analyses of teclistamab, talquetamab, or daratumumab serum concentrations and ADAs may additionally be used to evaluate sBCMA or to evaluate safety or antimyeloma activity aspects that address concerns arising during or after the study period for further characterization of immunogenicity. Genetic analyses will not be performed on these serum samples. Participant confidentiality will be maintained.

8.5.2. Analytical Procedures

PK

Serum samples will be analyzed to determine concentrations of teclistamab, talquetamab, or daratumumab (maintenance treatment only) using validated, specific, and sensitive methods. These analyses will be conducted by or under the supervision of the sponsor.

Immunogenicity

The detection and characterization of ADAs to teclistamab, talquetamab, or daratumumab (maintenance treatment only) will be performed using validated or appropriately qualified assay methods by or under the supervision of the sponsor. All samples collected for detection of ADAs

will also be evaluated for serum concentrations of teclistamab, talquetamab, or daratumumab (maintenance treatment only), as appropriate.

8.5.3. Pharmacokinetic Parameters and Evaluations

Parameters

Sparse samples collected for teclistamab, talquetamab, and daratumumab will be analyzed using a population PK approach, which may include data from other studies, and the results may be reported separately. Descriptive summary for teclistamab, talquetamab, and daratumumab concentrations at each timepoint will be presented.

8.5.4. Pharmacokinetic/Pharmacodynamic Evaluations

Concentration data and pharmacodynamic data of interest (including sBCMA) may be pooled in a population PK/pharmacodynamic modeling and the results of this analysis may be reported in a separate report.

8.5.5. Immunogenicity Parameters and Evaluations

ADAs to teclistamab, talquetamab, and daratumumab (maintenance treatment only) will be assessed from immunogenicity samples collected according to the relevant Schedule of Activities. These samples will be analyzed by the sponsor or sponsor's designee.

Immunogenicity samples will be screened for ADAs to teclistamab, talquetamab, or daratumumab (maintenance treatment only) and the titer of confirmed positive samples will be reported. Positive samples for binding antibodies will be tested for neutralizing antibodies to teclistamab, talquetamab, and daratumumab, as applicable. Immunogenicity bioanalysis may be conducted on PK samples collected at other timepoints, if deemed necessary.

8.6. Genetics

FISH of plasma cells should be performed to evaluate cytogenetic risk. Additionally, whole genome and gene expression profiling may be performed for exploratory studies.

8.7. Biomarkers

Peripheral blood, bone marrow aspirate, and core biopsy will be collected at baseline and during treatment per the relevant Schedule of Activities (Table 2, Table 7, Table 8 and Table 9). Additional biomarker samples may be collected to help understand unexplained AEs.

Biomarker analysis may be deferred or not performed, if during or at the end of the study, it becomes clear that the analysis will not have sufficient scientific value for biomarker evaluation, or if there are not enough samples or responders to allow for adequate biomarker evaluation.

8.7.1. Pharmacodynamics and Exploratory Biomarker Studies

Biomarker assessments will evaluate pharmacodynamic biomarkers indicative of the mechanism of action of Tec-DRd, Tec-DVRd, Tec-DR, Tec-D, CCI [redacted] (if applicable),

[redacted] Whole blood samples, bone marrow aspirate samples, and core biopsy will be collected to evaluate immune and tumor cell populations by flow cytometry, CITE-Seq, SeaHorse, SeqFiSH, and/or Next Gen sequencing, among others. In addition, the available apheresis product from the stem cell harvesting procedure described in Section 8.1.1.3.1 may be assessed for the presence of clonal plasma cells in the autograft.

Biomarker objectives will focus on:

- Characterization of antimyeloma activity (eg, effects on bone marrow).
- Characterization of immune responses in periphery indicative of mechanism of action for teclistamab (eg, changes in frequency and/or activation/exhaustion phenotypes of $CD8^+$, $CD4^+$ T cells), and daratumumab and lenalidomide (eg, impact on NK or T reg cells in the periphery).
- Exploration of biomarkers predictive of response/resistance in immune and tumor cell populations. These may include changes in: frequency of circulating immune cells and bone marrow cellular composition, spatial cell distribution in bone marrow, gene expression (eg, BCMA and GPRC5D in tumor plasma cells), and metabolic, genetic and epigenetic markers.
- Exploration of prognostic and disease markers in relation to efficacy parameters, including but not limited to standard cytogenetics (eg, del17p, t(4;14), t(14;16)) and molecular markers of risk stratification; bone marrow MRD.
- Determination of the proportion of participants with clonal plasma cell-negative autograft after 3 cycles of induction with Tec-DRd, Tec-DVRd, [redacted].

8.7.2. Minimal Residual Disease

MRD negativity is being evaluated in the field as a potential surrogate for PFS and OS. The relationship between MRD negativity, pharmacological, and clinical activity, including duration of response to Tec-DRd, Tec-DVRd, Tec-DR, Tec-D, [redacted], [redacted] will be explored. Bone marrow aspirate samples will be evaluated for MRD by NGF (EuroFlow). All MRD assessments will be analyzed by a central laboratory. Alternative methods of MRD assessment may be additionally explored.

See additional details for efficacy-related MRD evaluations in Section 8.2.3.

8.7.3. Additional Collections

If it is determined at any time before study completion that additional material is needed from a FFPE tumor sample for the successful completion of the protocol-specified analyses, the sponsor may request that additional material be retrieved from existing samples. Also, based on emerging scientific evidence, the sponsor may request additional material from previously collected tumor samples during or after study completion for a retrospective analysis. In this case, such analyses would be specific to research related to study drug(s) or the disease being investigated.

If a tissue biopsy is obtained as part of the SoC workup of an AE, the sponsor may request additional material from the previously collected biopsy sample during or after study completion for a retrospective research analysis related to study drug(s) or the disease being investigated.

9. STATISTICAL CONSIDERATIONS

Statistical analysis will be done by the sponsor or under the authority of the sponsor. A general description of the statistical methods to be used to analyze the efficacy and safety data is outlined below. Specific details will be provided in the Statistical Analysis Plan.

9.1. Statistical Hypotheses

No formal statistical hypothesis testing will be performed in this study.

9.2. Sample Size Determination

This study anticipates enrolling up to a maximum of approximately 320 participants. Arm A will enroll approximately 10 participants. Arms A1 and B will initially enroll approximately 20 participants each and may be expanded up to a total of 80 participants (Arms A1 and B combined). Arm C will enroll approximately 10 participants, and based on evolving data Arm C1 may also be opened to enroll approximately 10 participants. Arm [redacted] will initially enroll approximately 10 participants and may enroll up to a total of 30 participants. Arm [redacted] will initially enroll approximately 20 participants and may enroll up to 60 participants. Arms [redacted] and [redacted] will initially enroll approximately 10 participants each and may enroll up to 30 participants each. Based on emerging data from Arms A1, B, [redacted], and [redacted], Arms [redacted] and [redacted] may also be opened to enroll up to 30 participants each.

Table 62 presents the length of the widest 95% confidence interval for an AE rate, given the possible sample sizes per arm.

Table 62: Length of Widest 95% Confidence Interval Around AE Rates

Sample Size in Treatment Arm [a]	Length of Widest 95% CI [b]
10	62.6%
20	45.6%
30	37.4%
40	32.4%
50	28.9%
60	26.4%

a. Arm A1 and Arm B will initially enroll approximately 20 participants per cohort. Based on the safety snapshot and the totality of data, Arm A1 *and/or* Arm B may be expanded up to a total of 80 participants (Arms A1 and B combined); ie, a given treatment arm may have up to a maximum of 60 participants. Arms A, C and (if opened) C1 will have approximately 10 participants each. Arm [redacted] may have up to 30 participants. Arm [redacted] may have up to 60 participants. Arms [redacted] and [redacted], and (if opened) [redacted] and [redacted] will have up to 30 participants each.

b. Based on Clopper and Pearson 95% confidence interval, assuming an AE rate of 50%.

9.3. Populations for Analysis Sets

All safety analyses will be performed on the all treated population, which consists of participants who received at least one dose of study treatment. For the efficacy analysis, there will be 2 analysis populations: efficacy analysis population and response-evaluable population. The efficacy analysis

population consists of enrolled participants who received at least one dose of the study treatment. The response-evaluable population includes all enrolled participants who received at least one disease evaluation and have measurable disease at baseline (or at diagnostic baseline for Arms C, C1, and [redacted]), or who do not have measurable disease at baseline or screening but are assessed as CR or better.

9.4. Statistical Analyses

The SAP for this study will be finalized prior to any analysis of data. The SAP will include a more detailed description of the statistical analyses described in this section.

9.4.1. General Considerations

The primary endpoints are AE and SAE rate and severity during treatment with teclistamab-[redacted] based combination regimens for each arm. The primary analysis for each regimen will be performed after all treated participants have completed the maintenance phase [redacted] treatment phase or have been discontinued from study treatment by this time point. The final data cutoff and analysis, to potentially update secondary endpoints and safety, will occur at the end of study.

In addition, the following safety analyses are planned to be performed and data will be reviewed by the Study Safety Committee and the Study Steering Committee as outlined in Section 4.1.1:

Arm A:

- After at least 6 participants have received at least 2 cycles of Tec-DRd induction treatment with successful stem cell mobilization and collection performed in at least 3 participants after at least 3 cycles. As of Amendment 4, Arm A has enrolled 10 participants.

Arms A1 and B:

- After at least 10 participants in each arm have received at least 3 cycles of Tec-DR (Arm A1) or Tec-DVRd (Arm B) induction treatment. It is expected that 20 participants will be enrolled in each arm (Arm A1 and Arm B) at the time of this safety snapshot analysis, as per Figure 2 and Section 4.1.1.

[redacted]

Arms C, C1 (if opened), and [redacted]:

- After approximately 6 participants have received at least 3 cycles of Tec-DR, Tec-D, or [redacted] maintenance treatment. It is expected that 10 participants will be enrolled in each arm at the time of the respective safety snapshot analyses, as per Section 4.1.1.

The sponsor may take the decision that based on emerging data from other arms or from external data or based on practical considerations (eg, to reasonably align safety snapshots from different arms), these planned safety analyses may be modified. In addition, safety data from all arms will be reviewed on an ongoing basis and additional safety analyses may be triggered at any time during the study.

9.4.2. Primary Endpoints

The primary endpoints are AE and SAE rate and severity during treatment with teclistamab- and talquetamab-based combination regimens for each arm.

9.4.3. Secondary Endpoints

Summary of efficacy endpoints include:

- Rate of MRD negative CR, defined as the proportion of participants who achieve MRD negative status as determined by NGF with a sensitivity of 10^{-5}, and achieve CR or better response post-induction (Arms A, A1, B, [redacted], post-ASCT (Arms A, A1, B, [redacted].
- Rate of sustained MRD negative CR is defined as the proportion of participants who achieve MRD negative CR, confirmed for a minimum of 12 months apart and without any examination showing MRD-positive status in between, during the entire study treatment phase of each arm (all arms).
- Rate of MRD negative CR conversion and deepening during maintenance (all arms except [redacted]
- Response rates post-induction treatment, post-ASCT, post-maintenance, and best overall (Arms A, A1, B, [redacted]
  - ORR defined as the proportion of participants who achieve PR or better, according to the IMWG criteria, by the respective time point.
  - CR or better rate, defined as the proportion of participants who achieve CR or better, according to the IMWG criteria, by the respective time point.
  - VGPR or better rate is defined as the proportion of participants achieving VGPR or better, according to the IMWG criteria, by the respective time point.
- Response rates post-maintenance and best overall (Arms C, C1, and [redacted])
  - CR or better rate.

- Arms A, A1, B, D, [redacted] and [redacted], and [if applicable] [redacted] and [redacted] Duration of response, defined as the duration from the date of initial documentation of a response (PR or better) according to the IMWG criteria (Appendix 5) to the date of first documented evidence of progressive disease according to the IMWG criteria (Appendix 5).
- Arms C, C1, and [redacted]: Duration of response defined as the date from the first maintenance dose to the date of first documented evidence of progressive disease according to the IMWG criteria (Appendix 5).
- PFS defined as the duration from the date of first dosing to the date of first documented evidence of progressive disease or death, whichever comes first (all arms).

All secondary endpoints will be summarized utilizing descriptive statistics.

MRD negativity as measured regardless of response per IMWG will also be described.

9.4.4. Safety Analyses

All safety analyses will be made on the All-treated Population.

AEs

The verbatim terms used in the CRF by investigators to identify AEs will be coded using the Medical Dictionary for Regulatory Activities. Any AE occurring at or after the initial administration of study treatment through the day of last dose plus 30 days is considered to be treatment-emergent. All reported TEAEs will be included in the analysis. For each AE, the percentage of participants who experience at least 1 occurrence of the given event will be summarized by arm.

Summaries, listings, datasets, or participant narratives may be provided, as appropriate, for those participants who die, who discontinue treatment due to an AE, or who experience a severe event or an SAE.

Parameters with predefined NCI-CTCAE severity grades will be summarized. ASTCT severity grading will be utilized for CRS and ICANS (see Section 8.4.1). Change from baseline to the worst AE grade experienced by the participant during the study will be provided as shift tables.

Worst AE grade during treatment will be presented, according to NCI-CTCAE Version 5.0.

Clinical Laboratory Tests

Laboratory data will be summarized by type of laboratory test. Descriptive statistics will be calculated for each laboratory analytes at baseline and for observed values and changes from baseline at each scheduled time point. Changes from baseline results will be presented in pre- versus posttreatment cross-tabulations (with classes for below, within, and above normal ranges).

ECGs

Electrocardiogram data will be summarized.

Stem Cell Harvest (Arms A, A1, B, CCI [redacted]

Number (%) of participants who undergo stem cell mobilization and who undergo ASCT will be summarized. Stem cell yield, the number of $CD34^+$ cells transplanted, days to engraftment for neutrophils, and days to engraftment for platelets will be summarized by mean, standard deviation, median, minimum, and maximum.

9.4.5. Other Analyses

9.4.5.1. Pharmacokinetic Analyses

PK analyses will be performed on the teclistamab PK-evaluable, talquetamab PK-evaluable, and daratumumab PK-evaluable population defined as participants who have received at least 1 dose of teclistamab, talquetamab, or daratumumab and have at least 1 evaluable PK sample. All serum concentrations below the lowest quantifiable concentration or missing data will be labeled as such in the concentration data presentation. Concentrations below the lowest quantifiable concentration will be treated as zero in the summary statistics. All participants and samples excluded from the analysis will be clearly documented in the study report.

Descriptive statistics will be used to summarize teclistamab, talquetamab, and daratumumab serum concentrations at each sampling time.

Population PK analysis of serum concentration-time data of teclistamab, talquetamab, and daratumumab will be performed using nonlinear mixed effects modeling. The details will be given in a population PK analysis plan and the results of the analysis will be presented in a separate report.

9.4.5.2. Biomarkers Analyses

Results of exploratory biomarker and pharmacodynamic biomarker analyses may be presented in a separate report. Planned analyses are based on the availability of clinically valid assays and may be deferred if emerging study data show no likelihood of providing useful scientific information.

9.4.5.3. Immunogenicity Analyses

Immunogenicity samples will be screened for ADAs to teclistamab, talquetamab or daratumumab, and the titer of confirmed positive samples will be reported. Positive samples for binding antibodies will be tested for neutralizing antibodies to teclistamab, talquetamab or daratumumab as applicable. Immunogenicity bioanalysis may be conducted on PK samples collected at other timepoints, if deemed necessary. The incidence of antibodies to teclistamab, talquetamab, and daratumumab will be summarized separately for all participants who receive ≥1 dose of teclistamab, talquetamab, or daratumumab and have appropriate samples for detection of ADAs. A listing of any participants who are positive for ADAs to teclistamab, talquetamab or daratumumab will also be presented.

9.4.5.4. Pharmacokinetic/Pharmacodynamic Analyses

If sufficient data are available, PK/pharmacodynamic modeling may be performed, including but not limited to, exploring the relationship between serum concentrations of teclistamab, talquetamab, or daratumumab and endpoints of clinical safety and efficacy.

9.4.5.5. PRO Analyses

The CCI [redacted] will be summarized descriptively at each timepoint.

9.5. Interim Analyses

Not applicable.

10. SUPPORTING DOCUMENTATION AND OPERATIONAL CONSIDERATIONS

10.1. Appendix 1: Abbreviations and Definitions

ADA	anti-drug antibody
ADC	antibody drug conjugate
ADL	activities of daily living
AE	adverse event
AESI	adverse event of special interest
ALT	alanine aminotransferase
ANC	absolute neutrophil count
anti-HBc	hepatitis B core antibody
anti-HBs	hepatitis B surface antibody
aPTT	activated partial thromboplastin time
ASCT	autologous stem cell transplant
AST	aspartate aminotransferase
ASTCT	American Society for Transplantation and Cellular Therapy
$AUC_{ss\ (x-y)}$	area under the serum concentration versus time curve during a dose interval time period (tau) at steady state (time x – time y)
AUC_{tau}	area under the serum concentration versus time curve during a dose interval time period (tau)
B cell	B lymphocyte
BCMA	B cell maturation antigen
BiPAP	bilevel positive airway pressure
BMI	body mass index
BMNC	bone marrow mononuclear cells
BMPC	bone marrow plasma cell
BSA	body surface area
CAR	chimeric antigen receptor
CBC	complete blood count
CD	cluster of differentiation
CI	confidence interval
CIS	carcinoma in situ
C_{max}	maximum concentration
$C_{max\cdot ss}$	maximum concentration at steady state
CMV	cytomegalovirus
CNS	central nervous system
COPD	chronic obstructive pulmonary disease
COVID-19	coronavirus disease 2019
CPAP	continuous positive airway pressure
CR	complete response
CrCl	creatinine clearance
CRS	cytokine release syndrome
CT	computed tomography
C_{trough}	trough concentration
$C_{trough.ss}$	trough concentration at steady state
CYP	cytochrome P450
DGHO	Deutsche Gesellschaft Für Hämatologie und Medizinische Onkologie
DLT	dose-limiting toxicity
DNA	deoxyribonucleic acid
DOR	duration of response
DRd	daratumumab in combination with lenalidomide and dexamethasone

DVRd	daratumumab in combination with bortezomib, lenalidomide, and dexamethasone
DVTd	daratumumab in combination with bortezomib, thalidomide, and dexamethasone
DW-MRI	diffusion-weighted magnetic resonance imaging
EBV	Epstein Barr virus
EC_{50}	concentration at 50% of maximal effect
EC_{90}	concentration at 90% of maximal effect
$EC_{90\ max}$	maximum EC_{90} (upper bound concentration of 90% maximum effect)
$EC_{90,mean}$	mean EC_{90},
ECG	electrocardiogram
ECOG	Eastern Cooperative Oncology Group
eCRF	electronic case report form
eDC	electronic data capture
EEA	European Economic Area
EEG	electroencephalogram
CCI [redacted]	[redacted]
EOT	end of treatment
EU	European Union
FEV1	forced expiratory volume in 1 second
FFPE	formalin-fixed, paraffin-embedded
FISH	fluorescence in situ hybridization
FLC	free light chain
FSH	follicle-stimulating hormone
GCP	Good Clinical Practice
G-CSF	granulocyte colony-stimulating factor
GFR	glomerular filtration rate
GM-CSF	granulocyte-macrophage colony-stimulating factor
H_2	histamine 2 receptor
HBsAg	hepatitis B surface antigen
HBV	hepatitis B virus
HCV	hepatitis C virus
HDT	high-dose chemotherapy
Hep B	hepatitis B
HIV	human immunodeficiency virus
HLH	hemophagocytic lymphohistiocytosis
HR	hazard ratio
HRQoL	heath-related quality of life
HRT	hormone replacement therapy
IB	Investigator's Brochure
ICANS	immune effector cell-associated neurotoxicity syndrome
ICE	immune effector cell-associated encephalopathy
ICF	informed consent form
ICH	International Council for Harmonisation
ICMJE	International Committee of Medical Journal Editors
ICP	intracranial pressure
IEC	Independent Ethics Committee
IF	immunofluorescence
IFE	immunofixation electrophoresis
IFN	interferon
Ig	immunoglobulin
IgG4-PAA	immunoglobulin G4 proline, alanine, alanine
IgM	Immunoglobulin M

IHC	immunohistochemistry
IL	interleukin
IMiD	immunomodulatory drug
IMP	Investigational Medicinal Product
IMWG	International Myeloma Working Group
IPPI	Investigational Product Preparation Instructions
IRB	Institutional Review Board
IRC	Independent Review Committee
IV	intravenous(ly)
IVIg	intravenous immunoglobulin
IWRS	interactive web response system
LMWH	low molecular weight heparin
MAS	macrophage activation syndrome
MHC	major histocompatability complex
M-protein	monoclonal paraprotein
MR	minimal response
MRD	minimal residual disease
MRI	magnetic resonance imaging
mRNA	messenger ribonucleic acid
CCI [redacted]	[redacted]
NCCN	National Comprehensive Cancer Network
NCI-CTCAE	National Cancer Institute Common Terminology Criteria for Adverse Events
NDMM	newly diagnosed multiple myeloma
ND-TEMM	newly diagnosed transplant eligible multiple myeloma
NGF	next-generation flow
NK	natural killer
ORR	overall response rate
OS	overall survival
$PaCO_2$	partial pressure of arterial carbon dioxid
PC	plasma cell
PCR	polymerase chain reaction
PD	progressive disease
PD-L1	programmed death ligand 1
PET	positron emission tomography
PFS	progression-free survival
PFS2	progression-free survival on next-line therapy
PI	proteasome inhibitor
PK	pharmacokinetic(s)
PML	progressive multifocal leukoencephalopathy
PO	per os (oral)
PPP	Pregnancy Prevention Plan
PQC	product quality complaint
PR	partial response
QW	weekly
Q2W	every 2 weeks
Q4W	every 4 weeks
RBC	red blood cell
RNA	ribonucleic acid
RP	radical prostatectomy
RP2D	recommended Phase 2 dose or regimen
RT	radiation therapy
RVd	lenalidomide, bortezomib, dexamethasone

SAE	serious adverse event
SAP	Statistical Analysis Plan
SARS	severe acute respiratory syndrome
sBCMA	soluble BCMA
SC	subcutaneous(ly)
sCR	stringent complete response
SIFE	serum immunofixation electrophoresis
SIPPM	Site Investigational Product Procedures Manual
SmPC	Summary of Product Characteristics
SoA	Schedule of Activities
SoC	standard of care
SOC	system organ class
SPD	sum of the products of the maximal perpendicular diameters of the measured lesions
SPEP	serum M-protein quantitation by electrophoresis
SST	subsequent antimyeloma therapy
SUSAR	suspected unexpected serious adverse reaction
SWOG	South West Oncology Group
CCI [redacted]	[redacted]
T cell	T lymphocyte
TEAE	treatment-emergent adverse event
Tec-D	teclistamab in combination with daratumumab
Tec-DR	teclistamab in combination with daratumumab SC and lenalidomide
Tec-DRd	teclistamab in combination with daratumumab SC, lenalidomide, and dexamethasone
Tec-DVRd	teclistamab in combination with daratumumab SC, bortezomib, lenalidomide, and dexamethasone
CCI [redacted]	[redacted]
TLS	tumor lysis syndrome
TSH	thyroid stimulating hormone
UIFE	urine immunofixation electrophoresis
ULN	upper limit of normal
UPEP	urine M-protein quantitation by electrophoresis
US	United States
USPI	United States Prescribing Information
VCd	bortezomib, cyclophosphamide, and dexamethasone
VGPR	very good partial response
VRd	bortezomib, lenalidomide, and dexamethasone
VSV	varicella-zoster virus
VTd	bortezomib in combination with thalidomide, and dexamethasone

Definitions of Terms

sARR	systemic reaction related to study drug administration (regardless of the route of administration)

10.2. Appendix 2: Regulatory, Ethical, and Study Oversight Considerations

10.2.1. Regulatory and Ethical Considerations

The study will be performed in compliance with ICH-GCP E6 (R2) guidelines, as well as country-specific regulations, in particular Arzneimittelgesetzes (AMG) and GCP-Verordnung (GCP-V).

Investigator Responsibilities

The investigator is responsible for ensuring that the study is performed in accordance with the protocol, current ICH guidelines on GCP, and applicable regulatory and country-specific requirements.

Good Clinical Practice is an international ethical and scientific quality standard for designing, conducting, recording, and reporting studies that involve the participation of human participants. Compliance with this standard provides public assurance that the rights, safety, and well-being of study participants are protected, consistent with the principles that originated in the Declaration of Helsinki, and that the study data are credible.

Protocol Amendments

Neither the investigator nor the sponsor will modify this protocol without a formal amendment by the sponsor. All protocol amendments must be issued by the sponsor, and signed and dated by the investigator. Protocol amendments must not be implemented without prior IEC/IRB approval, or when the relevant competent authority has raised any grounds for non-acceptance, except when necessary to eliminate immediate hazards to the participants, in which case the amendment must be promptly submitted to the IEC/IRB and relevant competent authority. Documentation of amendment approval by the investigator and IEC/IRB must be provided to the sponsor. When the change(s) involve only logistic or administrative aspects of the study, the IEC/IRB (where required) only needs to be notified.

During the course of the study, in situations where a serious departure from the protocol is unavoidable, the investigator or other physician in attendance will contact the appropriate sponsor representative listed in the Contact Information page(s), which will be provided as a separate document. Except in emergency situations, this contact should be made before implementing any departure from the protocol. In all cases, contact with the sponsor must be made as soon as possible to discuss the situation and agree on an appropriate course of action. The data recorded in the CRF and source documents will reflect any departure from the protocol, and the source documents will describe this departure and the circumstances requiring it.

Regulatory Approval/Notification

This protocol and any amendment(s) must be submitted to the appropriate regulatory authorities in each respective country, if applicable. A study may not be initiated until all local regulatory requirements are met.

Required Prestudy Documentation

The following documents must be provided to the sponsor before shipment of study drug(s) to the study site:

- Protocol and amendment(s), if any, signed and dated by the principal investigator
- A copy of the dated and signed (or sealed, where appropriate per local regulations), written IEC/IRB approval of the protocol, amendments, ICF, any recruiting materials, and if applicable, participant compensation programs. This approval must clearly identify the specific protocol by title and number and must be signed (or sealed, where appropriate per local regulations) by the chairman or authorized designee.
- Name and address of the IEC/IRB, including a current list of the IEC/IRB members and their function, with a statement that it is organized and operates according to GCP and the applicable laws and regulations. If accompanied by a letter of explanation, or equivalent, from the IEC/IRB, a general statement may be substituted for this list. If an investigator or a member of the study site personnel is a member of the IEC/IRB, documentation must be obtained to state that this person did not participate in the deliberations or in the vote/opinion of the study.
- Regulatory authority approval or notification, if applicable
- Signed and dated statement of investigator if applicable
- Documentation of investigator qualifications (eg, curriculum vitae)
- Completed investigator financial disclosure form from the principal investigator, where required
- Signed and dated clinical trial agreement, which includes the financial agreement
- Any other documentation required by local regulations

The following documents must be provided to the sponsor before enrollment of the first participant:

- Completed investigator financial disclosure forms from all sub-investigators.
- Documentation of sub-investigator qualifications (eg, curriculum vitae).
- Name and address of any local laboratory conducting tests for the study, and a dated copy of current laboratory normal ranges for these tests, if applicable.
- Local laboratory documentation demonstrating competence and test reliability (eg, accreditation/license), if applicable.

IEC/IRB

Before the start of the study, the investigator (or sponsor where required) will provide the IEC/IRB with current and complete copies of the following documents (as required by local regulations):

- Final protocol and, if applicable, amendments
- Sponsor-approved ICF (and any other written materials to be provided to the participants)

- IB (or equivalent information) and amendments/addenda
- Sponsor-approved participant recruiting materials
- Information on compensation for study-related injuries or payment to participants for participation in the study, if applicable
- Investigator's curriculum vitae or equivalent information (unless not required, as documented by the IEC/IRB)
- Information regarding funding, name of the sponsor, institutional affiliations, other potential conflicts of interest, and incentives for participants
- Any other documents that the IEC/IRB requests to fulfill its obligation

This study will be undertaken only after the IEC/IRB has given full approval of the final protocol, amendments (if any, excluding the ones that are purely administrative, with no consequences for participants, data or study conduct, unless required locally), the ICF, applicable recruiting materials, and participant compensation programs, and the sponsor has received a copy of this approval. This approval letter must be dated and must clearly identify the IEC/IRB and the documents being approved.

During the study the investigator (or sponsor where required) will send the following documents and updates to the IEC/IRB for their review and approval, where appropriate:

- Protocol amendments (excluding the ones that are purely administrative, with no consequences for participants, data or study conduct)
- Revision(s) to ICF and any other written materials to be provided to participants
- If applicable, new or revised participant recruiting materials approved by the sponsor
- Revisions to compensation for study-related injuries or payment to participants for participation in the study, if applicable
- New edition(s) of the IB and amendments/addenda
- Summaries of the status of the study at intervals stipulated in guidelines of the IEC/IRB (at least annually)
- Reports of AEs that are serious, unlisted/unexpected, and associated with study treatment
- New information that may adversely affect the safety of the participants or the conduct of the study
- Deviations from or changes to the protocol to eliminate immediate hazards to the participants
- Report of deaths of participants under the investigator's care
- Notification if a new investigator is responsible for the study at the site
- Development Safety Update Report and Line Listings, where applicable
- Any other requirements of the IEC/IRB

For all protocol amendments (excluding the ones that are purely administrative, with no consequences for participants, data or study conduct), the amendment and applicable ICF revisions must be submitted promptly to the IEC/IRB for review and approval before implementation of the change(s).

At the end of the study, the investigator (or sponsor where required) will notify the IEC/IRB about the study completion (if applicable, the notification will be submitted through the head of investigational institution).

Other Ethical Considerations

For study-specific ethical design considerations, refer to Section 4.1.3.

10.2.2. Financial Disclosure

Investigators and sub-investigators will provide the sponsor with sufficient, accurate financial information in accordance with local regulations to allow the sponsor to submit complete and accurate financial certification or disclosure statements to the appropriate regulatory authorities. Investigators are responsible for providing information on financial interests during the course of the study and for 1 year after completion of the study.

Refer to Required Prestudy Documentation (above) for details on financial disclosure.

10.2.3. Informed Consent Process

Each participant must give written consent according to local requirements after the nature of the study has been fully explained. The ICF(s) must be signed before performance of any study-related activity. The ICF(s) that is/are used must be approved by both the sponsor and by the reviewing IEC/IRB and be in a language that the participant can read and understand. The informed consent should be in accordance with principles that originated in the Declaration of Helsinki, current ICH and GCP guidelines, applicable regulatory requirements, and sponsor policy.

Before enrollment in the study, the investigator or an authorized member of the study site personnel must explain to potential participants the aims, methods, reasonably anticipated benefits, and potential hazards of the study, and any discomfort participation in the study may entail. Participants will be informed that their participation is voluntary and that they may withdraw consent to participate at any time. They will be informed that choosing not to participate will not affect the care the participant will receive for the treatment of his or her disease. Participants will be told that alternative treatments are available if they refuse to take part and that such refusal will not prejudice future treatment. Finally, they will be told that the investigator will maintain a participant identification register for the purposes of long-term follow-up if needed and that their records may be accessed by health authorities and authorized sponsor personnel without violating the confidentiality of the participant, to the extent permitted by the applicable law(s) or regulations. By signing the ICF the participant is authorizing such access, which includes permission to obtain information about his or her survival status. It also denotes that the participant agrees to allow his or her study physician to recontact the participant for the purpose of obtaining consent for additional safety evaluations, and subsequent disease-related treatments, if needed.

The participant will be given sufficient time to read the ICF and the opportunity to ask questions. After this explanation and before entry into the study, consent should be appropriately recorded by means of the participant's personally dated signature. After having obtained the consent, a copy of the ICF must be given to the participant.

If the participant is unable to read or write, an impartial witness should be present for the entire informed consent process (which includes reading and explaining all written information) and should personally date and sign the ICF after the oral consent of the participant is obtained.

10.2.4. Recruitment Strategy

The sponsor may provide the study sites with optional patient recruitment material templates, to be used in accordance with local regulations and policies.

10.2.5. Data Protection

Privacy of Personal Data

The collection and processing of personal data from participants enrolled in this study will be limited to those data that are necessary to fulfill the objectives of the study.

These data must be collected and processed with adequate precautions to ensure confidentiality and compliance with applicable data privacy protection laws and regulations, ie, Datenschutz-Grundverordnung (DSGVO) and Bundes-/Landesdatenschutzgesetze (BDSG/LDSG). Appropriate technical and organizational measures to protect the personal data against unauthorized disclosures or access, accidental or unlawful destruction, or accidental loss or alteration must be put in place. Sponsor personnel whose responsibilities require access to personal data agree to keep the identity of participants confidential.

The informed consent obtained from the participant includes explicit consent for the processing of personal data and for the investigator/institution to allow direct access to his or her original medical records (source data/documents) for study-related monitoring, audit, IEC/IRB review, and regulatory inspection. This consent also addresses the transfer of the data to other entities and to other countries.

The participant has the right to request through the investigator access to his or her personal data and the right to request rectification of any data that are not correct or complete. Reasonable steps will be taken to respond to such a request, taking into consideration the nature of the request, the conditions of the study, and the applicable laws and regulations.

Pharmacodynamic and exploratory biomarker; PK; and immunogenicity research is not conducted under standards appropriate for the return of data to participants. In addition, the sponsor cannot make decisions as to the significance of any findings resulting from exploratory research. Therefore, exploratory research data will not be returned to participants or investigators, unless required by law or local regulations. Privacy and confidentiality of data generated in the future on stored samples will be protected by the same standards applicable to all other clinical data.

10.2.6. Storage, Use, Transfer, and Retention of Data and Samples

Study samples will be coded or anonymized at all times in accordance with the informed consent and will not be labeled with personal identifiers.

Investigator and study site will only store, use, transfer and retain data and study samples, including optional study samples, in accordance with the informed consent and applicable law, and in accordance with any separate written agreement with sponsor. Other than what is specified in a separate written agreement with sponsor, study site and investigator shall not conduct or facilitate any research by a third party not required by the protocol (i) on participants if such research interferes with the conduct of the study or (ii) on samples collected from study participants during the study, including optional samples, if the research relates to teclistamab, talquetamab, daratumumab, bortezomib, or lenalidomide or (iii) on data collected from study participants during the study if the research relates to teclistamab, talquetamab, daratumumab, bortezomib, or lenalidomide.

Sponsor may store, use, transfer or retain the data and share study samples, including optional study samples, for uses not specified by the protocol, including compatible research, in compliance with the informed consent and applicable law.

10.2.7. Committees Structure

A Study Safety Committee will be established to monitor safety. This committee will include 2 independent external medical experts in the relevant therapeutic area. After their review, the Study Safety Committee will make recommendations regarding the continuation of the study to the Study Steering Committee. The Study Steering Committee will make a decision regarding the continuation of the study.

10.2.8. Use of Information and Publication

All information, including but not limited to information regarding teclistamab and talquetamab, supplied by the sponsor to the study site or investigator and not previously published, and any data or analysis generated as a result of this study, are considered confidential and remain the sole property of the sponsor. Study site and investigator shall not use this information except in the performance of this study and shall not disclose this information to anyone except to persons involved in the study that need such information to assist in conducting the study, and then only on like terms of confidentiality and non-use.

Study site and investigator shall not publish study results except as required by law or as specified in a separate, written agreement between the sponsor and the study site or investigator.

The sponsor will register the study and publish the study results in compliance with applicable law and may register the study or publish study results when not required.

Authorship of any peer-reviewed publications will be determined by mutual agreement in line with International Committee of Medical Journal Editors authorship guidelines.

In accordance with standard editorial and ethical practice, the sponsor will generally support publication of multicenter studies only in their entirety and not as individual site data. In this case, a coordinating investigator will be designated by mutual agreement.

Registration of Clinical Studies and Disclosure of Results

The sponsor will register and disclose the existence of and the results of clinical studies as required by law. The disclosure of the final study results will be performed after the end of study in order to ensure the statistical analyses are relevant.

10.2.9. Data Quality Assurance

Data Quality Assurance/Quality Control

Steps to be taken to ensure the accuracy and reliability of data include the selection of qualified investigators and appropriate study sites, review of protocol procedures with the investigator and study site personnel before the study, periodic monitoring visits by the sponsor, and direct transmission of clinical laboratory data from a central laboratory into the sponsor's data base. Written instructions will be provided for collection, handling, storage, and shipment of samples.

Guidelines for eCRF completion will be provided and reviewed with study site personnel before the start of the study. The sponsor will review eCRF for accuracy and completeness during on-site monitoring visits and after transmission to the sponsor; any discrepancies will be resolved with the investigator or designee, as appropriate. After upload of the data into the study database they will be verified for accuracy and consistency with the data sources.

10.2.10. Case Report Form Completion

eCRFs are prepared and provided by the sponsor for each participant in electronic format. All data relating to the study must be recorded in CRF. All CRF entries, corrections, and alterations must be made by the investigator or authorized study site personnel. The investigator must verify that all data entries in the CRF are accurate and correct.

The study data will be transcribed by study site personnel from the source documents onto an eCRF, if applicable. Study-specific data will be transmitted in a secure manner to the sponsor.

Worksheets may be used for the capture of some data to facilitate completion of the CRF. Any such worksheets will become part of the participant's source documents. Data must be entered into eCRF in English. The eCRF must be completed as soon as possible after a participant visit and the forms should be available for review at the next scheduled monitoring visit.

All participative measurements (eg, pain scale information or other questionnaires) will be completed by the same individual who made the initial baseline determinations whenever possible.

If necessary, queries will be generated in the eDC tool. If corrections to an CRF are needed after the initial entry into the CRF, this can be done in either of the following ways:

- Investigator and study site personnel can make corrections in the eDC tool at their own initiative or as a response to an auto query (generated by the eDC tool).
- Sponsor or sponsor delegate can generate a query for resolution by the investigator and study site personnel.

10.2.11. Source Documents

At a minimum, source documents consistent in the type and level of detail with that commonly recorded at the study site as a basis for standard medical care must be available for the following: participant identification, eligibility, and study identification; study discussion and date of signed informed consent; dates of visits; results of safety and efficacy parameters as required by the protocol; record of the assessment by the investigator of all AEs and follow-up of AEs; concomitant medication; treatment receipt/dispensing/return records; study treatment administration information; and date of study completion and reason for early discontinuation of study treatment or withdrawal from the study, if applicable.

The author of an entry in the source documents should be identifiable.

Specific details required as source data for the study and source data collection methods will be reviewed with the investigator before the study and will be described in the monitoring guidelines (or another equivalent document).

The minimum source documentation requirements for Section 5.1, Inclusion Criteria and Section 5.2, Exclusion Criteria that specify a need for documented medical history are as follows:

- Referral letter from treating physician or
- Discharge summaries

Inclusion and exclusion criteria not requiring documented medical history must be verified at a minimum by participant interview or other protocol-required assessment (eg, physical examination, laboratory assessment) and documented in the source documents.

An eSource system may be utilized, which contains data traditionally maintained in a hospital or clinic record to document medical care (eg, electronic source documents) as well as the clinical study-specific data fields as determined by the protocol. There data are electronically extracted for use by the sponsor. If eSource is utilized, references made to the eCRF in the protocol include the eSource system but information collected through eSource may not be limited to that found in the eCRF.

10.2.12. Monitoring

The sponsor will use a combination of central, remote, or on-site monitoring techniques to monitor this study.

The sponsor will perform on-site monitoring visits as frequently as necessary. The monitor will record dates of the visits in a study site visit log that will be kept at the study site. The first post-initiation visit will be made as soon as possible after enrollment has begun. At these visits, the monitor will compare the data entered into the eCRF with the source documents (eg, hospital/clinic/physician's office medical records). The nature and location of all source documents will be identified to ensure that all sources of original data required to complete the eCRF are known to the sponsor and study site personnel and are accessible for verification by the sponsor study site contact. If electronic records are maintained at the study site, the method of verification must be discussed with the study site personnel.

Direct access to source documents (medical records) must be allowed for the purpose of verifying that the recorded data are consistent with the original source data. Findings from this review will be discussed with the study site personnel. The sponsor expects that, during monitoring visits, the relevant study site personnel will be available, the source documents will be accessible, and a suitable environment will be provided for review of study-related documents. The monitor will meet with the investigator on a regular basis during the study to provide feedback on the study conduct.

In addition to on-site monitoring visits, remote contacts can occur. It is expected that during these remote contacts, study site personnel will be available to provide an update on the progress of the study at the site.

Central monitoring will take place for data identified by the sponsor as requiring central review.

10.2.13. On-Site Audits

Representatives of the sponsor's clinical quality assurance department may visit the study site at any time during or after completion of the study to conduct an audit of the study in compliance with regulatory guidelines and company policy. These audits will require access to all study records, including source documents, for inspection. Participant privacy must, however, be respected. The investigator and study site personnel are responsible for being present and available for consultation during routinely scheduled study site audit visits conducted by the sponsor or its designees.

Similar auditing procedures may also be conducted by agents of any regulatory body, either as part of a national GCP compliance program or to review the results of this study in support of a regulatory submission. The investigator should immediately notify the sponsor if he or she has been contacted by a regulatory agency concerning an upcoming inspection.

10.2.14. Record Retention

In compliance with the ICH/GCP guidelines, the investigator/institution will maintain all CRF and all source documents that support the data collected from each participant, as well as all study documents as specified in ICH/GCP Section 8, Essential Documents for the Conduct of a Clinical Trial, and all study documents as specified by the applicable regulatory requirement(s). The investigator/institution will take measures to prevent accidental or premature destruction of these documents.

Essential documents must be retained until at least 2 years after the last approval of a marketing application in an ICH region and until there are no pending or contemplated marketing applications in an ICH region or until at least 2 years have elapsed since the formal discontinuation of clinical development of the investigational product. These documents will be retained for a longer period if required by the applicable regulatory requirements or by an agreement with the sponsor. It is the responsibility of the sponsor to inform the investigator/institution as to when these documents no longer need to be retained. For trials performed under Regulation [EU] No. 536/2014, the sponsor and the investigator shall archive the content of the clinical trial master file for at least 25 years after the end of the clinical trial.

If the responsible investigator retires, relocates, or for other reasons withdraws from the responsibility of keeping the study records, custody must be transferred to a person who will accept the responsibility. The sponsor must be notified in writing of the name and address of the new custodian. Under no circumstance shall the investigator relocate or dispose of any study documents before having obtained written approval from the sponsor.

If it becomes necessary for the sponsor or the appropriate regulatory authority to review any documentation relating to this study, the investigator/institution must permit access to such reports.

10.2.15. Study and Site Start and Closure

First Act of Recruitment

The first participant screened is considered the first act of recruitment and it becomes the study start date.

Study/Site Termination

The sponsor reserves the right to close the study site or terminate the study at any time for any reason at the sole discretion of the sponsor. Study sites will be closed upon study completion. A study site is considered closed when all required documents and study supplies have been collected and a study site closure visit has been performed.

The investigator may initiate study site closure at any time, provided there is reasonable cause and sufficient notice is given in advance of the intended termination.

Reasons for the early closure of a study site by the sponsor or investigator may include but are not limited to:

- Failure of the investigator to comply with the protocol, the requirements of the IEC/IRB or local health authorities, the sponsor's procedures, or GCP guidelines
- Inadequate recruitment of participants by the investigator
- Discontinuation of further study treatment development

10.3. Appendix 3: Adverse Events, Serious Adverse Events, Product Quality Complaints, and Other Safety Reporting: Definitions and Procedures for Recording, Evaluating, Follow-up, and Reporting

10.3.1. Adverse Event Definitions and Classifications

AE

An AE is any untoward medical occurrence in a clinical study participant administered a pharmaceutical (investigational or non-investigational) product. An AE does not necessarily have a causal relationship with the study drug(s). An AE can therefore be any unfavorable and unintended sign (including an abnormal finding), symptom, or disease temporally associated with the use of a medicinal (investigational or non-investigational) product, whether or not related to that medicinal (investigational or non-investigational) product (definition per ICH).

This includes any occurrence that is new in onset or aggravated in severity or frequency from the baseline condition, or abnormal results of diagnostic procedures, including laboratory test abnormalities.

NOTE: The sponsor collects AEs starting with the signing of the ICF (refer to All Adverse Events under Section 8.4.1).

All AEs, regardless of seriousness, severity, or presumed relationship to study intervention, must be recorded using medical terminology in the source document and the CRF. Whenever possible, diagnoses should be given when signs and symptoms are due to a common etiology (eg, cough, runny nose, sneezing, sore throat, and head congestion should be reported as "upper respiratory infection"). Investigators must record in the CRF their opinion concerning the relationship of the AE to study therapy. All measures required for AE management must be recorded in the source document and reported according to sponsor instructions.

SAE

An SAE based on ICH and EU Guidelines on Pharmacovigilance for Medicinal Products for Human Use is any untoward medical occurrence that at any dose:

- Results in death
- Is life-threatening
  (The participant was at risk of death at the time of the event. It does not refer to an event that hypothetically might have caused death if it were more severe).
- Requires inpatient hospitalization or prolongation of existing hospitalization
- Results in persistent or significant disability/incapacity
- Is a congenital anomaly/birth defect
- Is a suspected transmission of any infectious agent via a medicinal product
- Is Medically Important*

NOTE: Events that do not qualify as an AE cannot be reported as a SAE, even if the conditions for seriousness are met. In particular, this is the case for events due to disease progression leading to death, hospitalization, etc.

*Medical and scientific judgment should be exercised in deciding whether expedited reporting is also appropriate in other situations, such as important medical events that may not be immediately life threatening or result in death or hospitalization but may jeopardize the participant or may require intervention to prevent 1 of the other outcomes listed in the definition above. These should usually be considered serious. This also includes the situation when treatment-invoked signs and symptoms of disease progression are determined by the investigator to be more likely related to the study treatment than being expected from the underlying disease.

If a serious and unexpected AE occurs for which there is evidence suggesting a causal relationship between study treatment and the event (eg, death from anaphylaxis), the event must be reported as a serious and unexpected suspected adverse reaction even if it is a component of the study endpoint (eg, all-cause mortality).

Unlisted (Unexpected) Adverse Event/Reference Safety Information

An AE is considered unlisted if the nature or severity is not consistent with the applicable product reference safety information. For teclistamab, talquetamab, or daratumumab, the expectedness of an AE will be determined by whether or not it is listed in the IB and Addenda. For lenalidomide, bortezomib, and dexamethasone with a marketing authorization, the expectedness of an AE will be determined by whether or not it is listed in the applicable product information sheet (eg, package insert/summary of product characteristics).

10.3.2. Attribution Definitions

Assessment of Causality

The causal relationship to study treatment is assessed by the investigator and documented in the Medical Records.

The assessment of causality must consider the following factors:

- Temporal relationship
- Clinical characteristics of event
- Pharmacological plausibility
- Confounding risk factors:
  - Concomitant medication
  - Underlying/concurrent disease
  - Family/social history

- Challenge:
  - De-challenge: Did the reaction improve when the investigational product was withdrawn, in the absence of any other treatment?
  - Re-challenge: What happens if participant is re-challenged with investigational product?
- Other considerations: Participant characteristics and past medical history, and quality of information

The following selection must be used to assess all AEs.

Related

There is a reasonable causal relationship between study drug administration and the AE.

Not Related

There is not a reasonable causal relationship between study drug administration and the AE.

10.3.3. NCI-CTCAE Grading of Adverse Event Severity

Adverse event severity is a clinical determination of the intensity of an AE. The severity assessment for an AE or serious AE should be completed using the NCI-CTCAE, Version 5.0. Any AE or SAE not listed in the NCI-CTCAE, Version 5.0 will be graded according to the investigator clinical judgment by using the standard grades as follows:

- Grade 1: Mild; asymptomatic or mild symptoms; clinical or diagnostic observations only; intervention not indicated.
- Grade 2: Moderate; minimal, local or noninvasive intervention indicated; limiting age-appropriate instrumental activities of daily living (ADL)[a].
- Grade 3: Severe or medically significant but not immediately life-threatening; hospitalization or prolongation of hospitalization indicated; disabling; limiting self-care activities of daily living[b].
- Grade 4: Life-threatening consequences; urgent intervention indicated.
- Grade 5: Death related to AE.

a. Instrumental ADL refers to preparing meals, shopping for groceries or clothes, using the telephone, managing money, etc.
b. Self-care ADL refers to bathing; dressing and undressing; feeding self; using the toilet; taking medications; and not bedridden.

The investigator should use clinical judgment in assessing the severity of events not directly experienced by the participant (eg, laboratory abnormalities).

10.3.4. Special Reporting Situations

Safety events of interest on a study drug in an interventional study that may require expedited reporting or safety evaluation include, but are not limited to:

- Overdose of a study drug
- Suspected abuse/misuse of a study drug
- Accidental or occupational exposure to a study drug
- Any failure of expected pharmacologic action (ie, lack of effect if used according to the local label) of a sponsor study treatment (to be reported as a PQC for marketed products)
- Unexpected therapeutic or clinical benefit from use of a study drug
- Medication error, intercepted medication error, or potential medication error involving a Johnson & Johnson medicinal product (with or without participant exposure to the Johnson & Johnson medicinal product, eg, product name confusion, product label confusion, intercepted prescribing or dispensing errors)
- Exposure to study treatment from breastfeeding

Special reporting situations should be recorded in the CRF. Any special reporting situation that meets the criteria of an SAE should be recorded on the SAE page of the CRF.

10.3.5. Adverse Event Reporting Procedures

All AEs

All AEs, regardless of seriousness, severity, or presumed relationship to study treatment, must be recorded using medical terminology in the source document and the CRF. Whenever possible, diagnoses should be given when signs and symptoms are due to a common etiology (eg, cough, runny nose, sneezing, sore throat, and head congestion should be reported as "upper respiratory infection") with the exception of CRS and ICANS where symptoms should be reported as well. Investigators must record in the CRF their opinion concerning the relationship of the AE to study therapy. All measures required for AE management must be recorded in the source document and reported according to sponsor instructions.

For all studies with an outpatient phase, including open-label studies, the participant must be provided with a "wallet (study) card" and instructed to carry this card with them for the duration of the study indicating the following:

- Study number
- Statement, in the local language(s), that the participant is participating in a clinical study
- Investigator's name and 24-hour contact telephone number
- Local sponsor's name and 24-hour contact telephone number (for medical personnel only)
- Site number

- Participant number
- Any other information that is required to do an emergency breaking of the blind

SAEs

All SAEs that have not resolved by the end of the study, or that have not resolved upon the participant's discontinuation from the study, must be followed until any of the following occurs:

- The event resolves
- The event stabilizes
- The event returns to baseline, if a baseline value/status is available
- The event can be attributed to agents other than the study treatment or to factors unrelated to study conduct
- It becomes unlikely that any additional information can be obtained (participant or health care practitioner refusal to provide additional information, lost to follow-up after demonstration of due diligence with follow-up efforts)

Any event requiring hospitalization (or prolongation of hospitalization) that occurs during participation in the study must be reported as an SAE, except hospitalizations for the following:

- Hospitalizations not intended to treat an acute illness or AE (eg, social reasons such as pending placement in long-term care facility).
- Surgery or procedure planned before entry into the study (must be documented in the eCRF). **NOTE:** Hospitalizations that were planned before the signing of the ICF, and where the underlying condition for which the hospitalization was planned has not worsened, will not be considered SAEs. Any AE that results in a prolongation of the originally planned hospitalization is to be reported as a new SAE.
- The administration of blood or platelet transfusion unless prolonged hospitalization occurs.
- Should additional monitoring of participants be required following resolution of clinical symptoms of CRS, extended hospitalization for participant observation will not be considered an SAE.

Disease progression should not be recorded as an AE or SAE term; instead, signs and symptoms of clinical sequelae resulting from disease progression/lack of efficacy that are determined by the investigator to be of clinical significance should be reported per the usual reporting requirements (refer to Adverse Event Definitions and Classifications in Appendix 3).

Information regarding SAEs will be transmitted to the sponsor using an SAE reporting form and safety report form of the CRF, which must be completed and reviewed by a physician from the study site, and transmitted in a secure manner to the sponsor immediately, but no later than 24 hours of their knowledge of the event. The initial and follow-up reports of an SAE should be transmitted in a secure manner electronically or by facsimile (fax). Telephone reporting should be the exception and the reporter should be asked to complete the appropriate form(s) first.

10.3.6. Product Quality Complaint Handling

Definition

A PQC is defined as any suspicion of a product defect related to manufacturing, labeling, or packaging, ie, any dissatisfaction relative to the identity, quality, durability, reliability, or performance of a distributed product, including its labeling, drug delivery system, or package integrity. A PQC may have an impact on the safety and efficacy of the product. In addition, it includes any technical complaints, defined as any complaint that indicates a potential quality issue during manufacturing, packaging, release testing, stability monitoring, dose preparation, storage or distribution of the product or the drug delivery system.

Procedures

All initial PQCs must be reported to the sponsor by the study site personnel within 24 hours after being made aware of the event.

A sample of the suspected product should be maintained under the correct storage conditions until a shipment request is received from the sponsor.

10.3.7. Contacting Sponsor Regarding Safety, Including Product Quality

The names (and corresponding telephone numbers) of the individuals who should be contacted regarding safety issues, PQC, or questions regarding the study are listed in the Contact Information page(s), which will be provided as a separate document.

10.4. Appendix 4: IMWG Diagnostic Criteria

Diagnostic criteria for myeloma must be met when the participant was diagnosed. Multiple myeloma is defined as clonal BMPCs ≥10% or biopsy-proven bony or extramedullary plasmacytoma[a] and at least 1 of the following:

- Evidence of end-organ damage, specifically:
  - **C:** Hypercalcemia: serum calcium >0.25 mmol/L (>1 mg/dL) higher than the ULN or >2.75 mmol/L (>11 mg/dL)
  - **R:** Renal insufficiency: creatinine clearance <40 mL per min[b] or serum creatinine >177 µmol/L (>2 mg/dL)
  - **A:** Anemia: hemoglobin value of >20 g/L below the lower limit of normal, or a hemoglobin value <100 g/L
  - **B:** Bone lesions: 1 or more osteolytic lesions on skeletal radiography, CT, or PET-CT[c,d]
- Any 1 or more of the following biomarkers of malignancy:
  - Clonal BMPC%[a] ≥60%
  - Involved:uninvolved serum FLC ratio[e] ≥100
  - >1 focal lesions on MRI studies[f]

a. Clonality should be established by showing κ/λ-light-chain restriction on flow cytometry, immunohistochemistry, or immunofluorescence. Bone marrow plasma cell percentage should preferably be estimated from a core biopsy specimen; in case of a disparity between the aspirate and core biopsy, the highest value should be used.
b. Measured or estimated by validated equations.
c. If bone marrow has less than 10% clonal plasma cells, more than one bone lesion is required to distinguish from solitary plasmacytoma with minimal marrow involvement.
d. PET-CT=^{18}F-fluorodeoxyglucose PET with CT.
e. These values are based on the serum Freelite assay (The Binding Site Group, Birmingham, UK). The involved FLC must be ≥100 mg/L.
f. Each focal lesion must be 5 mm or more in size.

Source: Rajkumar 2011

10.5. Appendix 5: IMWG Response Criteria

Response	Response Criteria
sCR	• CR as defined below, *plus*<br>• Normal FLC ratio, *and*<br>• Absence of clonal PCs by immunohistochemistry (κ/λ ratio ≤4:1 or ≥1:2 for κ and λ participants, respectively, after counting ≥100 plasma cells) or negative 2-4 color flow cytometry
CR[a]	• Negative immunofixation of serum and urine, *and*<br>• Disappearance of any soft tissue plasmacytomas, *and*<br>• <5% PCs in bone marrow<br>• No evidence of initial M-protein isotype(s) on immunofixation of the serum and urine[b]
VGPR[a]	• Serum and urine M-component detectable by immunofixation but not on electrophoresis, *or*<br>• ≥90% reduction in serum M-component plus urine M-component <100 mg/24 hours<br>• In addition to the above criteria, if present at baseline >90% reduction in the sum of the products of the maximal perpendicular diameters of the measured lesions (SPD) compared with baseline for soft tissue plasmacytoma
PR	• ≥50% reduction of serum M-protein and reduction in 24-hour urinary M-protein by ≥90% or to < 200 mg/24 hours<br>• If serum and urine M-protein are not measurable, a decrease ≥50% in the difference between involved and uninvolved FLC levels is required in place of the M-protein criteria<br>• In addition to the above criteria, if present at baseline, ≥50% reduction in the size (SPD) of soft tissue plasmacytomas is also required
Minimal response	• ≥25% but ≤49% reduction of serum M-protein *and* reduction in 24-hour urine M-protein by 50% to 89%<br>• In addition to the above criteria, if present at baseline, ≥50% reduction in the size of soft tissue plasmacytomas is also required
Stable disease	• Not meeting criteria for sCR, CR, VGPR, PR, MR, or progressive disease
Progressive disease[c]	Any one or more of the following criteria:<br>• Increase of 25% from lowest response value in any of the following:<br>– Serum M-component (absolute increase must be ≥0.5 g/dL), *and/or*<br>– Urine M-component (absolute increase must be ≥200 mg/24 hours), *and/or*<br>– Only in participants without measurable serum and urine M-protein levels and without measurable disease by FLC levels, bone marrow plasma cell percentage (the absolute percentage must be >10%)<br>• Appearance of a new lesion(s), ≥50% increase from nadir in SPD of >1 lesion, or ≥50% increase in the longest diameter of a previous lesion >1 cm in short axis<br>• Definite development of new bone lesions or definite increase in the size of existing bone lesions<br>• ≥50% increase in circulating plasma cells (minimum of 200 cells/μL) if this is the only measure of disease

a. Clarifications to the criteria for coding CR and VGPR in participants in whom the only measurable disease is by serum FLC levels: CR in such participants indicates a normal FLC ratio of 0.26 to 1.65 (or reference range for sponsor central laboratory for this study: 0.22 to 1.52) in addition to CR criteria listed above. VGPR in such participants requires a ≥90% decrease in the difference between involved and uninvolved FLC levels.

b. In some cases, it is possible that the original M-protein light chain isotype is still detected on immunofixation but the accompanying heavy-chain component has disappeared; this would not be considered as a CR even though the heavy-chain component is not detectable, because it is possible that the clone evolved to one that secreted only light chains. Thus, if a participant has IgA lambda myeloma, then to qualify as CR there should be no IgA detectable on serum or urine immunofixation; if free lambda is detected without IgA, then it must be accompanied by a different heavy-chain isotype (IgG, IgM, etc.).

c. Clarifications to the criteria for coding progressive disease: bone marrow criteria for progressive disease are to be used only in participants without measurable disease by M-protein and by FLC levels; "25% increase" refers to M-protein, and FLC, and does not refer to bone lesions, or soft tissue plasmacytomas and the "lowest response value" does not need to be a confirmed value.

NOTE: All response categories (CR, sCR, VGPR, PR, MR, and progressive disease) require 2 consecutive assessments made at any time before the institution of any new therapy; CR, sCR, VGPR, PR, MR, and stable disease categories also require no known evidence of progressive or new bone lesions if radiographic studies were performed. VGPR and CR categories require serum and urine studies regardless of whether disease at baseline was measurable on serum, urine, both, or neither.

NOTE: To qualify for VGPR or PR/minimal response, the sum of products of the perpendicular diameters of the existing extramedullary plasmacytomas must have decreased by >90% or ≥50%, respectively, and new plasmacytomas must not have developed. To qualify for disease progression, either the sum of products of the perpendicular diameters of the existing extramedullary plasmacytomas must have increased by ≥50%, or the longest diameter of previous lesion >1 cm in short axis must have increased ≥50%, or a new plasmacytoma must have developed. When not all existing extramedullary plasmacytomas are reported, but the sum of products of the perpendicular diameters of the reported plasmacytomas have increased by ≥50%, then the criterion for disease progression is met.

Radiographic studies are not required to satisfy these response requirements. Bone marrow assessments need not be confirmed. For progressive disease, serum M-component increases of ≥1 g/dL are sufficient to define relapse if lowest M-component is ≥5 g/dL.

Source: Adapted from Durie 2015; Kumar 2016; and Rajkumar 2011

10.6. Appendix 6: ECOG Performance Status

Grade	ECOG Performance Status
0	Fully active, able to carry on all pre-disease performance without restriction
1	Restricted in physically strenuous activity but ambulatory and able to carry out work of a light or sedentary nature (eg, light housework, office work)
2	Ambulatory and capable of all self-care but unable to carry out any work activities. Up and about >50% of waking hours
3	Capable of only limited self-care, confined to bed or chair more than 50% of waking hours
4	Completely disabled. Cannot carry on any self-care. Totally confined to bed or chair
5	Dead

Source: Oken 1982

The conversion between Karnofsky Performance Status and ECOG is shown below.

Karnofsky Performance Status	ECOG Performance Status
100	0
90	1
70	2
50	3
30	4
0	5

Source: Ma 2010

10.7. Appendix 7: Cockcroft-Gault Formula

Cockcroft-Gault formula:

To calculate the participant's creatinine clearance (CrCl), use the following Cockcroft-Gault formula:

$$\text{CrCl}= \frac{(140 - \text{age [in years]}) \times \text{weight}^{a}\ (\text{kg})\ \ (\times\ 0.85 \text{ for females})}{(72 \times \text{serum creatinine [mg/dL]})}$$

[a.] In obese individuals (body mass index ≥ 30 kg/m^2), instead of using total body weight, use adjusted body weight for more accurate estimate of renal function.

Source: Pai 2010; US Dept Health 2020

If the serum creatinine is obtained using the International System of Units (SI) (ie, µmol/L), use the following formula to convert SI units to conventional (mg/dL) units (Fischbach 2004):

$$\text{serum creatinine (mg/dL)} = \frac{\text{serum creatinine (}\mu\text{mol/L)}}{88.4}$$

Formula to measure CrCl:

$$\text{CrCl}= \frac{U_{Cr} \times U_{vol}}{P_{Cr} \times T_{min}}$$

$$\text{Corrected CrCl}= \frac{\text{CrCl} \times 1.73}{\text{BSA}}$$

U_{Cr},=Urine creatinine concentration; U_{vol},=Urine volume from 24-hour collection; P_{Cr},=plasma creatinine concentration; T_{min},=collection time in minutes (24 hours x 60 minutes); BSA=body surface area.

Source: Cockcroft and Gault 1976

For the calculation of BSA, see Appendix 12.

10.8. Appendix 8: Serum Calcium Corrected for Albumin

If calcium is expressed in mg/dL and albumin is expressed in g/dL:

Corrected calcium (mg/dL) = Serum calcium (mg/dL) + 0.8 × (4 - serum albumin [g/dL])

If calcium is expressed in mmol/L and albumin is expressed in g/L:

Corrected calcium (mmol/L) = Serum calcium (mmol/L) + 0.02 × (40 - serum albumin [g/L])

Source: Burtis 1999

10.9. Appendix 9: Contraceptive Guidance and Lenalidomide Global/Local PPP Programs

Participants must follow contraceptive measures as outlined in Section 5.1. Pregnancy information will be collected and reported as noted in Section 8.4.5.

Definitions

Participants of Childbearing Potential

A participant is considered fertile following menarche and until becoming postmenopausal unless permanently sterile (see below).

Participants Not of Childbearing Potential

- **Premenarchal:** A premenarchal state is one in which menarche has not yet occurred.
- **Postmenopausal:** A postmenopausal state is defined as no menses for 24 months without an alternative medical cause. A high FSH level (>40 IU/L or mIU/mL) in the postmenopausal range may be used to confirm a postmenopausal state in participants not using hormonal contraception or HRT, however in the absence of 12 months of amenorrhea, a single FSH measurement is insufficient.
  If there is a question about menopausal status in a participant on HRT, the participant will be required to use one of the non-estrogen-containing hormonal highly effective contraceptive methods if they wish to continue HRT during the study.
- **Permanent absence of reproductive potential: (for the purpose of this study)**
  - Has undergone a procedure that precludes reproductive potential
  - Has a congenital abnormality that precludes reproductive potential

NOTE: If the childbearing potential changes after start of the study (eg, a premenarchal participant experiences menarche) or the risk of pregnancy changes (eg, a participant who is not heterosexually active becomes active), a participant must begin a highly effective method of contraception, as described throughout the inclusion criteria.

If reproductive status is questionable, additional evaluation should be considered.

Contraceptive (birth control) use by participants must be consistent with local regulations regarding the acceptable methods of contraception for those participating in clinical studies.

Due to the embryo-fetal risk associated with lenalidomide, all participants treated with lenalidomide must be enrolled in the applicable PPP in their region. Investigators must comply with lenalidomide global PPP or local PPP program, as implemented in the postmarketing setting, and ensure that all participants adhere to the applicable program. If the centrally supplied generic lenalidomide is approved in a country/territory, the locally approved PPP for the product should be used. If the centrally supplied lenalidomide is not approved in a country/territory, the global PPP should be used. If locally supplied lenalidomide, the locally approved PPP for the product should be used.

Typical use failure rates may differ from those when used consistently and correctly. Use must be consistent with local regulations regarding the use of contraceptive methods for participants in clinical studies.

**EXAMPLES OF HIGHLY EFFETIVE METHODS OF CONTRACEPTIVES[a]:**
**USER INDEPENDENT**<br>**Highly Effective Methods That Are User Independent** *Failure rate of <1% per year when used consistently and correctly.*
• Implantable progestogen-only hormone contraception associated with inhibition of ovulation[b]
• Intrauterine device (IUD)
• Intrauterine hormone-releasing system (IUS)
• Tubal closure (eg, bilateral tubal occlusion, bilateral tubal ligation)
• Azoospermic partner *(vasectomized or due to medical cause)*<br>*(Vasectomized partner is a highly effective contraceptive method provided that the vasectomized partner is the sole sexual partner of the participant of childbearing potential and the absence of sperm in the vasectomized partner has been confirmed. If not, additional highly effective method of contraception must be used. Spermatogenesis cycle is approximately 74 days.)*
**USER DEPENDENT**<br>**Highly Effective Methods That Are User Dependent** *Failure rate of <1% per year when used consistently and correctly.*
• Combined (estrogen- and progestogen-containing) hormonal contraception associated with inhibition of ovulation[b]<br>– oral<br>– intravaginal<br>– transdermal<br>– injectable
• Progestogen-only hormone contraception associated with inhibition of ovulation[b]<br>– oral<br>– injectable
• Sexual abstinence<br>*(Sexual abstinence is considered a highly effective method only if defined as refraining from heterosexual intercourse during the entire period of risk associated with the study treatment. The reliability of sexual abstinence needs to be evaluated in relation to the duration of the study and the preferred and usual lifestyle of the participant.)*
**NOT ALLOWED AS SOLE METHOD OF CONTRACEPTION DURING THE STUDY (not considered to be highly effective - failure rate of ≥1% per year)**
• Progestogen-only oral hormonal contraception where inhibition of ovulation is not the primary mode of action.
• Condom with or without spermicide[c]
• Cap, diaphragm, or sponge with spermicide
• A combination of external condom with either cap, diaphragm, or sponge with spermicide (double-barrier methods)[c]
• Periodic abstinence (calendar, symptothermal, post-ovulation methods)
• Withdrawal (coitus-interruptus)
• Spermicides alone

• Lactational amenorrhea method (LAM)
a) Typical use failure rates may differ from those when used consistently and correctly. Use must be consistent with local regulations regarding the use of contraceptive methods for participants in clinical studies.<br>b) Hormonal contraception may be susceptible to interaction with the study treatment, which may reduce the efficacy of the contraceptive method. In addition, consider if the hormonal contraception may interact with the study treatment. A participant using oral contraceptives must use an additional contraceptive method.<br>c) Multiple condoms should not be used together (due to risk of failure with friction).

Please refer to the most up to date SmPC contraception language for the specific agents being used. Where the contraceptive language differs from this protocol the more stringent/conservative requirements should be applied.

10.10. Appendix 10: Conversion Table for Steroids

Steroid	Approximate Equivalent Dose to Dexamethasone 8 mg	Approximate Equivalent Dose to Dexamethasone 20 mg	Approximate Equivalent Dose to Dexamethasone 40 mg
Methylprednisolone	45 mg	105 mg	215 mg
Prednisone/Prednisolone	55 mg	135 mg	270 mg

Source: Global RxPh 2017

10.11. Appendix 11: Asthma Guidelines (Adults)

**Components of Severity**		**Classification of Asthma Severity**			
			**Persistent**		
		**Intermittent**	**Mild**	**Moderate**	**Severe**
**Impairment**	Symptoms	≤2d/week	≥2d/week but not daily	Daily	Throughout the day
	Nighttime awakenings	≤2×month	3-4×month	>1×week, but not nightly	Often 7×week
Normal FEV1/FVC: 8-19 years: 85% 20-39 years: 80% 40-59 years: 75% 60-80 years: 70%	Short acting beta$_2$-agonist use for symptom control (not prevention of exercise-induced bronchospasm)	≤2d/week	>2d/week, but not daily, and not >1×day	Daily	Several times per day
	Interference with normal activity	None	Minor limitation	Some limitation	Extremely limited
	Lung function	Normal FEV1 between exacerbations			
	FEV1 (% predicted)	>80%	>80%	60%-80%	<60^
	FEV1/FVC	Normal	Normal	Reduced 5%	Reduced >5%
**Risk**	Exacerbations requiring oral systemic corticosteroids	0-1/year	≥2/year Relative annual risk may be related to FEV1	≥2/year Relative annual risk may be related to FEV1	≥2/year Relative annual risk may be related to FEV1
		Consider severity and interval since last exacerbation. Frequency and severity may fluctuate over time for participants in any severity category.			
**Recommended Step for Initiating Treatment**		Step 1	Step 2	Step 3 and consider short course of oral steroids	Step 4 or 5 and consider short course of oral steroids
		In 2-6 weeks, evaluate level of asthma control that is achieved. Adjust therapy accordingly.			

**Components of Control**		Classification of Asthma Control		
		Well Controlled	Not Well Controlled	Very Poorly Controlled
**Symptoms**		≤2d/week	>2d/week	Throughout the day
**Impairment**	Nighttime awakenings	≤2×month	1-3×/week	≥4×/week
	Interference with normal activity	None	Some limitation	Extremely limited
	Short acting beta$_2$-agonist use for symptom control (not prevention of exercise-induced bronchospasm)	≤2d/week	>2d/week	Several times per day
	Lung function:			
	FEV1 or peak flow	>80%	60%-80%	<60%
	FEV1/FV	n/a	n/a	n/a
	Validated questionnaires:			
	Asthma Therapy Assessment Questionnaire	0	1-2	3-4

**Components of Severity**		**Classification of Asthma Severity**			
		**Intermittent**	**Persistent**		
			**Mild**	**Moderate**	**Severe**
	Asthma Control Questionnaire	≤0.75		≥1.5	n/a
	Asthma Control Test	≥20		16-19	≤15
**Risk**	Exacerbations requiring oral systemic corticosteroids	0-1/year	≥2/year		
		Consider severity and interval since last exacerbation			
	Reduction in lung growth/ Progressive loss of lung function	Evaluation requires long-term follow-up			
**Recommended Action for Treatment**		Well Controlled		Not Well Controlled	Very Poorly Controlled
		Maintain current step<br>Regular follow-up every 1-6 months<br>Consider step down if well controlled for ≥3 months		Step-up 1 step<br>Re-evaluate in 2-6 week<br>For side effects, consider alternative treatment options	Consider short course of oral steroids<br>Step-up 1-2 steps<br>Re-evaluate in 2 weeks<br>For side effects, consider alternative treatment options

Source: https://www.nhlbi.nih.gov/files/docs/guidelines/asthma_qrg.pdf

10.12. Appendix 12: Body Surface Area Calculation

Body surface area should be calculated using the Mosteller Formula (shown below).

$$\text{BSA} = \sqrt{\frac{Ht(inches) \times Wt(lbs)}{3131}}$$

or

$$\text{BSA} = \sqrt{\frac{Ht(cm) \times Wt(kg)}{3600}}$$

BSA = body surface area; Ht = height; Wt = weight

Source: Mosteller 1987

10.13. Appendix 13: Considerations for Outpatient Teclistamab [redacted] Administration Through the First Treatment Dose

When evaluating the suitability for outpatient administration, if allowed by local regulations and institutional guidance, investigators should assess the participant's clinical status and the healthcare facility capability to safely manage outpatient logistics and discuss with sponsor. General recommendations for each of these considerations are provided below.

Clinical Consideration

General guidance for clinical considerations for a participant who is suitable for outpatient administration and follow-up includes the following:

- No packed red blood cell or platelet transfusions within the last 7 days prior to dosing.
- No fever or active infection (bacterial, fungal, viral) since study enrollment.
- No Grade ≥3 hematologic AEs or Grade ≥3, clinically significant non-hematologic AEs.
- No clinically significant coagulopathy that would increase the risk of bleeding in the setting of cytopenia.
- No high tumor burden, as defined in Section 6.5.7.
- No risk factors for developing clinically significant TLS and requiring management with increased hydration, allopurinol, or rasburicase. Participants who are receiving prophylactic treatment for TLS are eligible for outpatient infusion, if deemed stable by the investigator.
- No rapidly progressing disease per investigator assessment.
- No deterioration in neurologic status, including mental status changes such as confusion or increased somnolence. The only exception is confusion or somnolence that has resolved and must be attributed to diphenhydramine premedication for teclistamab or talquetamab.
- The following laboratory parameters:
  - CrCL ≥30 mL/min
  - AST and ALT ≤3×ULN.

Logistical Consideration for Qualified Healthcare Facility

Outpatient administration and post-teclistamab or post-talquetamab injection follow-up must take place at a qualified healthcare facility. The following should be considered for outpatient administration and follow-up:

- Participant is required to stay within 30 minutes of transportation to the site and remain in the company of a competent adult at all times for a minimum of 48 hours after the first 3 doses of teclistamab (0.06, 0.3, and 1.5 mg/kg) or talquetamab (0.01, 0.06, and 0.4 mg/kg) (see Section 6.4.1).
- Site must prepare a documented plan to ensure timely management of CRS/ICANS if these occur in an outpatient setting, including a mechanism to notify the hematology team and treat with tocilizumab if clinically indicated.

- Participant and accompanying competent adult must be made aware of the presenting signs and symptoms of teclistamab- or talquetamab- associated toxicities, including but not limited, to CRS, ICANS, infections, etc.
- Site must provide participant with thermometer, educational material, and wallet card, including but not limited to, notification of enrollment in a clinical trial, emergency contact information, and signs and symptoms of CRS/ICANS. Participant will be able to contact a clinical team member after hours until 48 hours after the third dose.
- Participant should carry the wallet card for study participant identification at all times.
- Participant must comply with all the protocol requirement procedures, including measuring and recording of body temperature twice daily (≥8 hours apart) and coming to the site for safety assessments according to Table 3, Table 4, Table 5, and Table 6.
- Admission to the hospital is required at any time in the event of any presenting signs and symptoms of CRS and/or ICANS in association with teclistamab or talquetamab. Participants will be promptly treated upon presentation to the hospital and will remain hospitalized for at least 48 hours. If CRS/ICANS occurs at the first treatment dose or beyond of teclistamab or talquetamab, specifications in Table 37 must be followed.

10.14. Appendix 14: Severity Grading for CRS (ASTCT)

Grade	Toxicity
**Grade 1**	Fever[a] (Temperature ≥38°C)
**Grade 2**	Fever[a] (Temperature ≥38°C) with either:<br>• Hypotension not requiring vasopressors.<br>• And/or[c] hypoxia requiring low-flow nasal cannula[b] or blow-by.
**Grade 3**	Fever[a] (Temperature ≥38°C) with either:<br>• Hypotension requiring a vasopressor with or without vasopressin,<br>• And/or[c] hypoxia requiring high-flow nasal cannula,[b] facemask, nonrebreather mask, or Venturi mask.
**Grade 4**	Fever[a] (Temperature ≥38°C) with either:<br>• hypotension requiring multiple vasopressors (excluding vasopressin),<br>• And/or[c] hypoxia requiring positive pressure (eg, CPAP, BiPAP, intubation and mechanical ventilation).
**Grade 5**	Death

NOTE: By convention, Grade 5 CRS is defined as death due to CRS in which another cause is not the principal factor leading to this outcome.
NOTE: Organ toxicities associated with CRS may be graded according to NCI-CTCAE Version 5.0, but they do not influence CRS grading.

a. Fever not attributable to any other cause. In participants who have CRS then receive antipyretics or anticytokine therapy such as tocilizumab or steroids, fever is no longer required to grade subsequent CRS severity. In this case, CRS grading is driven by hypotension and/or hypoxia.
b. Low-flow nasal cannula is defined as oxygen delivered at ≤6 L/minute or blow-by oxygen delivery. High-flow nasal cannula is defined as oxygen delivered at >6 L/minute.
c. CRS grade is determined by the more severe event: hypotension or hypoxia not attributable to any other cause.

Source: Lee 2019

10.15. Appendix 15: ICE Tool

Category	Points
Orientation: orientation to year, month, city, hospital	4 points
Naming: ability to name 3 objects (eg, point to clock, pen, button)	3 points
Following commands: ability to follow simple commands (eg, "Show me 2 fingers" or "Close your eyes and stick out your tongue")	1 point
Writing: ability to write a standard sentence (eg, "Our national bird is the bald eagle.")	1 point
Attention: ability to count backward from 100 by 10	1 point

Source: Lee 2019

10.16. Appendix 16: Severity Grading for ICANS (ASTCT)

Neurotoxicity Domain	Grade 1	Grade 2	Grade 3	Grade 4
ICE score[a]	7-9	3-6	0-2	0 (patient is unarousable and unable to perform ICE testing)
Depressed level of consciousness[b]	Awakens spontaneously	Awakens to voice	Awakens only to tactile stimulus	Patient is unarousable or requires vigorous or repetitive tactile stimuli to arouse<br>Stupor or coma
Seizures	N/A	N/A	Any clinical seizure focal or general that resolves rapidly; or non-convulsive seizures on EEG that resolve with intervention	Life-threatening, prolonged seizures (>5 min) or repetitive clinical or electrical seizures without return to baseline in between
Motor findings[c]	N/A	N/A	N/A	Deep focal motor weakness such as hemiparesis or paraparesis
Raised ICP/cerebral edema	N/A	N/A	Focal/local edema on neuroimaging[d]	Diffuse cerebral edema on neuroimaging; decerebrate or decorticate posturing; or cranial nerve VI palsy; or papilledema; or Cushing's Triad

NOTE: By convention, Grade 5 ICANS is defined as death due to ICANS in which another cause is not the principal factor leading to this outcome.

NOTE: ICANS grade is determined by the most severe event (ICE score, level of consciousness, seizure, motor findings, raised ICP/cerebral edema) not attributable to any other cause. For example, a participant with an ICE score of 3 who has a generalized seizure is classified as having Grade 3 ICANS.

a. A participant with an ICE score of 0 may be classified as having Grade 3 ICANS if the participant is awake with global aphasia. But a participant with an ICE score of 0 may be classified as having Grade 4 ICANS if the participant is unarousable. See Section 8.3.7 for instructions for administering the ICE tool.

b. Depressed level of consciousness should be attributable to no other cause (eg, no sedating medication).

c. Tremors and myoclonus associated with immune effector cell therapies may be graded according to NCI-CTCAE Version 5.0 but they do not influence ICANS grading.

d. Intracranial hemorrhage with or without associated edema is not considered a neurotoxicity feature and is excluded from ICANS grading. It may be graded according to NCI-CTCAE Version 5.0.

Source: Lee 2019

10.17. Appendix 17: Prophylaxis of Venous Thromboembolism

Risk Assessment Model for the Management of Venous Thromboembolism in Patients with Multiple Myeloma Treated With IMiDs

Risk Factors	Actions
Individual risk factors	
Obesity[a]	If no risk factor or any 1 individual/myeloma-related risk factor is present<br>• Aspirin 81 to 325 mg once daily<br><br>If 2 or more individual/myeloma-related risk factors are present:<br>• LMWH (equivalent of enoxaparin 40 mg once daily)<br>• Full-dose warfarin or other vitamin K antagonists (target INR 2-3)
Previous venous thromboembolism	
Central venous catheter or pacemaker	
Associated disease<br>Cardiac disease<br>Chronic renal disease<br>Diabetes<br>Acute infection<br>Immobilization	
Surgery<br>General surgery<br>Any anesthesia<br>Trauma	
Medications<br>Erythropoietin	
Blood clotting disorders	
Myeloma-related risk factors	
Diagnosis	
Hyperviscosity	
Myeloma therapy-related risk factors	
High-dose dexamethasone[b]	All patients who receive high-dose dexamethasone or doxorubicin or multiagent chemotherapy, independent of the presence of additional risk factors:<br>• LMWH (equivalent of enoxaparin 40 mg once daily)<br>• Full-dose warfarin or other vitamin K antagonists (target INR 2-3)
Doxorubicin	
Multiagent chemotherapy	

NOTE: Per Section 6.12.2.5 and Section 6.12.3.1, the use of warfarin (or other vitamin K antagonists) during Induction or Maintenance Cycle 1 is prohibited unless no other therapeutic option is available.

a. Obesity defined as BMI $\geq$30 kg/m^2
b. $\geq$480 mg per month

Source: Palumbo 2008.

10.18. Appendix 18: Clinical Laboratory Tests

The following tests will be performed by the local laboratory:

Laboratory Assessments	Parameters	
Hematology	hemoglobin<br>white blood cell count with differential<br>absolute neutrophil count<br>absolute lymphocyte count<br>platelet count	
Coagulation	PT/INR<br>aPTT<br>fibrinogen	
Serum chemistry	sodium<br>potassium<br>creatinine<br>creatine kinase[a]<br>glucose<br>aspartate aminotransferase<br>alanine aminotransferase<br>gamma-glutamyl transferase<br>total bilirubin (direct bilirubin if Gilbert's disease)	alkaline phosphatase<br>lactic acid dehydrogenase<br>phosphate<br>albumin<br>lipase<br>calcium<br>TSH[b]
TLS	• Uric acid	
Pregnancy[c]	• Pregnancy tests must have a minimum sensitivity of 25 mIU/mL.	
Serology (screening only)	• Hepatitis B (see Table 61)[d]<br>– HBsAg, anti-HBs and anti-HBc<br>– HBV DNA if needed (see Table 61)<br>• Hepatitis C:<br>– Anti-HCV<br>– HCV-RNA if anti-HCV-positive<br>• HIV: HIV antibody	

aPTT=activated partial thromboplastin time; anti-HBc=hepatitis B core antibody; anti-HBs=hepatitis B surface antibody; β-hCG=β-human chorionic gonadotropin; CrCl=creatinine clearance; CRS=cytokine release syndrome; HBV=hepatitis B virus; HCV=hepatitis C virus; HBsAg=hepatitis B surface antigen; HIV=human immunodeficiency virus; INR=international normalized ratio; PCR=polymerase chain reaction; PT=prothrombin time; RBC=red blood cell; TLS=tumor lysis syndrome; TSH=thyroid-stimulating hormone

NOTE: CrCl will be calculated per Appendix 7.

a. As an increased risk of rhabdomyolysis has been associated with lenalidomide and concurrent statin use, monitoring of creatine kinase is to be performed only on participants who are treated with statins and receive concurrent treatment with lenalidomide. In these participants, creatine kinase is to be measured during the first weeks of treatment, at a minimum from Cycle 2 to Cycle 3 of induction and maintenance therapy.

b. The thyroid function assessment will be performed on Cycle 1 Day 1 and Cycle 4 Day 1 of induction for Arms A, A1, B, D, E, E1, F and F1, on Cycle 1 Day 1 of maintenance for Arms A, C, and [redacted].

c. Serum pregnancy test is required for screening. After screening, urine dipstick is suggested but assessment of pregnancy may be performed by other locally available testing (including serum pregnancy test).

d. See Section 5 and Section 8.3.5 to determine eligibility. See Section 8.3.5.3 for routine HBV and HCV monitoring.

10.19. Appendix 19: Study Conduct During COVID-19 Pandemic

It is recognized that the COVID-19 pandemic or other natural disaster may have an impact on the conduct of this clinical study due to, for example, self-isolation/quarantine by participants and study site personnel; travel restrictions/limited access to public places, including hospitals; study site personnel being reassigned to critical tasks.

In alignment with recent health authority guidance, the sponsor is providing options for study-related participant management in the event of disruption to the conduct of the study. This guidance does not supersede any local or government requirements or the clinical judgment of the investigator to protect the health and well-being of participants and site staff. If, at any time, a participant's safety is considered to be at risk, study treatment will be discontinued, and study follow-up will be conducted.

Scheduled visits that cannot be conducted in person at the study site will be performed to the extent possible remotely/virtually or delayed until such time that on-site visits can be resumed. At each contact, participants will be interviewed to collect safety data. Key efficacy endpoint assessments should be performed if required and as feasible. Participants will also be questioned regarding general health status to fulfill any physical examination requirement.

Every effort should be made to adhere to protocol-specified assessments for participants on study treatment, including follow-up. Modifications to protocol-required assessments may be permitted after consultation with the participant, investigator, and the sponsor. Missed assessments/visits will be captured in the clinical trial management system for protocol deviations. Discontinuations of study treatments and withdrawal from the study should be documented with the prefix "COVID-19 related" in the CRF.

The sponsor will continue to monitor the conduct and progress of the clinical study, and any changes will be communicated to the sites and to the health authorities according to local guidance. If a participant has tested positive for SARS CoV-2, the investigator should contact the sponsor's responsible medical officer to discuss plans for study treatment and follow-up. Modifications made to the study conduct as a result of COVID-19 should be summarized in the Clinical Study Report.

Recommendations for COVID-19 Vaccination

Per Section 6.12.1, administration of non-live vaccines approved or authorized for emergency use (eg, COVID-19) by local health authorities are allowed before or during this study.

It is recommended that participants receive prophylactic COVID-19 vaccination when locally available, at the discretion of investigator judgment or institutional practice, and in compliance with the study protocol and local labels for the vaccine. Below is general guidance for consideration.

Many vaccines against COVID-19 are being developed with different technologies and platforms and may have safety and efficacy profiles that are not fully characterized even after preliminary health authority approval. However, the benefit-risk ratio of receiving a COVID-19 vaccine among patients with multiple myeloma participating in teclistamab and talquetamab studies is considered to be positive and should be considered for administration while in compliance with the study protocol and when not otherwise contraindicated for use in the vaccine label.

Per protocol, live attenuated vaccines must be completed ≥4 weeks before the first dose of study treatment (see Section 5.2) or initiated ≥30 days after last dose of teclistamab and talquetamab (see Section 6.12.3.1). There are no specific timing restrictions for inactivated vaccines, which include vaccines which use alternative technology like mRNA or replication-incompetent viral vectors, per protocol. Enrollment into an interventional clinical trial for an experimental vaccine is prohibited during study. Any vaccination, including COVID-19 vaccinations, must be recorded on the Concomitant Medication page of the eCRF. AEs and SAEs associated with the COVID-19 vaccine must be collected and reported in accordance with the pharmacovigilance practice.

Consider COVID-19 re-vaccination after transplant according to the current NCCN or DGHO guidelines, or local standards, whichever is most up to date.

No data are currently available to suggest that COVID-19 vaccines pose specific or additional safety risk beyond other vaccines for cancer patients undergoing treatment. Theoretically, a diminished immune response may occur in immunocompromised patients, and therefore these patients may have reduced vaccine effectiveness.

For guidance on vaccination, please refer to:

National Comprehensive Cancer Network. Preliminary recommendations of the NCCN COVID-19 Vaccination Advisory Committee* Version 1.0 1/22/2021. NCCN https://www.nccn.org/covid-19/pdf/COVID-19_Vaccination_Guidance_V1.0.pdf (2021),

Garassino MC, et al. The ESMO call to action on COVID-19 vaccinations and patients with cancer: Vaccinate. Monitor. Educate. Ann Oncol. 2021;32:579-581. https://doi.org/10.1016/j.annonc.2021.01.068, and

Desai A, et al. COVID-19 vaccine guidance for patients with cancer participating in oncology clinical trials. Nature Reviews Clin Oncol. 2021;18:313-319. https://doi.org/10.1038/s41571-021-00487-z.

For additional information for reference or guidance, several organizations and journals have published recommendations for COVID-19 vaccine administration in cancer patients, including the following:

- European Society for Blood and Marrow Transplantation: https://www.ebmt.org/covid-19-and-bmt
- ASTCT: https://www.hematology.org/covid-19/ash-astct-covid-19-vaccination-for-hct-and-car-t-cell-recipients
- Centers for Disease Control and Prevention: https://www.cdc.gov/vaccines/covid-19/info-by-product/clinical-considerations.html

10.20. Appendix 20: CYP3A4 Inducers

The following is a list of CYP3A4 inducers:

- Barbiturates
- Brigatinib
- Carbamazepine
- Clobazam
- Dabrafenib
- Efavirenz
- Elagolix
- Enzalutamide
- Eslicarbazepine
- Letermovir
- Lorlatinib
- Modafinil
- Nevirapine
- Oritavancin
- Oxcarbazepine
- Perampanel
- Phenobarbital
- Phenytoin
- Pioglitazone
- Rifabutin
- Rifampin
- St. John's wort
- Telotristat
- Troglitazone

Adapted from: http://medicine.iupui.edu/flockhart/ 29 December 2021

Additional information about CYP3A4 inducers is available at: Flockhart: http://medicine.iupui.edu/flockhart/

10.21. Appendix 21: New York Heart Association Functional Classification

NYHA Class	Symptoms
I	Cardiac disease, but no symptoms and no limitation in ordinary physical activity, e.g. shortness of breath when walking, climbing stairs etc.
II	Mild symptoms (mild shortness of breath and/or angina) and slight limitation during ordinary activity.
III	Marked limitation in activity due to symptoms, even during less-than-ordinary activity, e.g. walking short distances (20–100 m).<br>Comfortable only at rest.
IV	Severe limitations. Experiences symptoms even while *at rest*. Mostly bedbound patients.

10.22. Appendix 22: Previous Schedule of Activities and Dose Regimens for Arm A and Arm C (Prior to Protocol Amendment 3)

Table 63: Guide to Previous Schedule of Activities and Dosing for Arm A and Arm C

	Arm A - Tec-DRd induction	Arm C – Tec-DR maintenance
Screening	Table 2	Table 2
Schedule of Activities	Table 64	Table 65
Schedule of PK, Immunogenicity, and Biomarker Sample Collection	Table 66	Table 67
Justification of treatment dose	Table 68	
Study treatment administered	Table 69	Table 70
Dose schedule and pretreatment medications	Table 71	Table 72
Skipping of Study Drugs	Table 73	Table 74

10.22.1. Schedule of Activities

Table 64: Schedule of Activities for Treatment Phase, EOT Visit, and Posttreatment Follow-up Phase – Arm A - Tec-DRd Induction

Assessment	Notes	Treatment Phase (28-day cycle)										Follow-up Phase
**Induction Cycle**		**Cycle 1**					**Cycles 2-6 (stem cell collection after C3 - Section 8.1.1.3.1)**		**End of Induction**	**HDT + ASCT**	**EOT (if not continued on maintenance)**	
**Day**		**1**	**2**	**4 (+2d)**	**8 (+2d)**	**15, 22 (±2d)**	**1 (±3d)**	**8, 15, 22 (±3d)**	**Within 2wk of com-pleting C6**	**Limited data collection (Section 8.1.1.3.3)**	**≤30d after last dose (±7d)**	**Pre-PD Q 12 wk (±14d) Post-PD Q 16 wk (±28 d)**
**STUDY PROCEDURES**												
ECOG performance status	See Appendix 6	X					X		X			
Physical examination	Symptom-directed	As clinically indicated										
Weight		X					X					
Neurologic examination including ICE tool	See Section 8.3.7 and Appendix 15<br>Prior to administration of study drugs.	X	X	As clinically indicated (see Section 6.5.2)								
Vital signs including oxygen saturation	See Table 24 for timing	X	X	X	X	X	X	X	X		X	
12-lead ECG		As clinically indicated										
**LABORATORY ASSESSMENTS: See Appendix 18**												
Hematology	Must be performed ≤72 hours before dosing and laboratory values for C1D1 must meet Section 5.1 criteria. If criteria are not met see Section 5.1 for guidance or repeated testing requirements.	X	X	X	X	X	X	X	X		X	
Chemistry		X	X	X	X	X	X	D15	X		X	
TSH		X					C4					
Creatine kinase	Perform only on participants who are treated with statins and receive concurrent treatment with lenalidomide, from Cycle 2 to Cycle 3, at a minimum.						X	D15				
TLS-related (uric acid)	Perform ≤72 hours before dosing<br>See Section 6.5.7 for additional assessments and management of TLS.	X			X	X						
HBV, HCV PCR	• For participants with history of HBV and/or HCV infection.<br>• See Section 8.3.5.3	X					C4				3 and 6 months (±1 month) after last dose	
Coagulation		As clinically indicated (including at CRS onset if applicable)										

Table 64: Schedule of Activities for Treatment Phase, EOT Visit, and Posttreatment Follow-up Phase – Arm A - Tec-DRd Induction

Assessment		Treatment Phase (28-day cycle)										Follow-up Phase
**Induction Cycle**	**Notes**	**Cycle 1**					**Cycles 2-6 (stem cell collection after C3 - Section 8.1.1.3.1)**		**End of Induction**	**HDT + ASCT**	**EOT (if not continued on maintenance)**	
**Day**		**1**	**2**	**4** (+2d)	**8** (+2d)	**15, 22** (±2d)	**1** (±3d)	**8, 15, 22** (±3d)	**Within 2wk of com-pleting C6**	**Limited data collection (Section 8.1.1.3.3)**	**≤30d after last dose** (±7d)	**Pre-PD Q 12 wk** (±14d) **Post-PD Q 16 wk** (±28 d)
Urine Pregnancy Test	• Females of childbearing potential<br>• Additional testing may be required per the local PPP<br>• Serum pregnancy test is acceptable	• Within 24 hours of C1D1.<br>• 10 to 14 days prior and again within 24 hours prior to first dose of lenalidomide.<br>• Weekly for first 4 weeks of lenalidomide treatment, thereafter, every 4 weeks (every 2 weeks for women with irregular menses) and as clinically indicated.									X	
**STUDY VISITS: See Section 6.4.1 for required safety monitoring through the first treatment dose of teclistamab & Appendix 13 for considerations for outpatient administration during this period**												
Study visits	If a visit occurs later than planned, adjust subsequent visit(s) accordingly or skip dose as per Table 45	X	X	X	X	X	X	X	X		X	X
**DISEASE EVALUATIONS: See Section 8.2 for details regarding evaluations and Section 8.1.1.6 for guidance regarding follow-up requirements.**												
Quantitative immunoglobulins	Central laboratory (local laboratory assessments may be used under specified circumstances [see Section 8.1.2]). The sample for C1D1 may be collected on or within 3 days prior to C1D1.	X					X		X		X (until confirmed PD or SST, whichever occurs earlier)<br>**NOTE**: SPEP and UPEP assessments continue until 30 days after the start of the first SST for participants with no PD or unconfirmed PD (ie, single instance of PD by laboratory assessment) while on treatment. For participants with measurable disease by light chain, serum FLC will also continue during this period.	
SPEP		X					X		X			
24-hour UPEP		X					X		X			
Serum FLC		• For participants with measurable disease by light chain: D1 each cycle<br>• Others: whenever CR or sCR is suspected										
SIFE/UIFE		X	D1 of each cycle and wherever CR or sCR are suspected									
Imaging for disease evaluation	See Section 8.2.4 for acceptable modalities	As clinically indicated to document response or progression										
Assessment of soft-tissue plasmacytoma	See Section 8.2.5 for acceptable modalities and instructions regarding biopsies	• For participants with a history of soft-tissue plasmacytoma<br>-For assessment by physical examination (if applicable), every 4 weeks (±1 week) until development of confirmed CR or PD or start of SST<br>-For assessment by radiology, every 12 weeks (±1 week) until confirmed CR or PD or start of SST<br>• As clinically indicated for other participants										
Bone marrow aspirate and core biopsy	See Table 60 (a portion of aspirate should be sent to the central laboratory for biomarker analysis)	• Sampling for MRD will be done after completion of Cycle 3 (before mobilization procedure) in participants with a response of VGPR or better and after completion of Cycle 6 in all participants irrespective of response<br>• Time of suspected CR (including sCR)<br>• Time of PD										

Table 64: Schedule of Activities for Treatment Phase, EOT Visit, and Posttreatment Follow-up Phase – Arm A - Tec-DRd Induction

**Assessment**		**Treatment Phase (28-day cycle)**										**Follow-up Phase**
**Induction Cycle**		**Cycle 1**					**Cycles 2-6 (stem cell collection after C3 - Section 8.1.1.3.1)**		**End of Induction**	**HDT + ASCT**	**EOT (if not continued on maintenance)**	
**Day**	**Notes**	**1**	**2**	**4** (+2d)	**8** (+2d)	**15, 22** (±2d)	**1** (±3d)	**8, 15, 22** (±3d)	**Within 2wk of com-pleting C6**	**Limited data collection (Section 8.1.1.3.3)**	**≤30d after last dose** (±7d)	**Pre-PD Q 12 wk** (±14d) **Post-PD Q 16 wk** (±28 d)
Imaging for MRD assessment (DW-MRI)	Optional for sites where available. See Section 8.2.4.1	• After Cycle 6 of induction treatment										
**ONGOING REVIEW**												
AEs		• Continue until 30 days after last dose of study treatment or until the start of SST, whichever comes first. Continue to report any AEs/SAEs related to study treatment until EOS.<br>• Limited AE/SAE reporting required during HDT+ASCT, see Section 8.1.1.3.4										
2nd primary malignancy		Continuous until EOS.										
Concomitant therapy	See Section 6.12	• Continuous until 30 days after last dose of study treatment or until the start of SST, whichever comes first. Continue to report concomitant therapy given for any AEs/SAEs considered related to study treatment until EOS.<br>• During HDT+ASCT, record only the concomitant treatments used to treat AE specified in Section 8.1.1.3.4										
SST	See Section 6.13											X (post-PD)
Survival		Continuous										Q 16 wk

Table 65: Schedule of Activities for Treatment Phase, EOT Visit, and Posttreatment Follow-up Phase – Arm A, Arm B, and Arm C - Tec-DR Maintenance

Assessments	Notes	Pre Maintenance	Maintenance Treatment Phase (28-day cycle)										Follow-up Phase
**Maintenance Cycle**		(Arm A or Arm B only)	**Cycle 1**					**Cycle 2**		**Cycles 3-18**		**EOT**	(Including Potential SoC Maintenance Treatment)
**Day**		**Within 28 d prior to starting maintenance**	**1**	**2**	**4** (+2d)	**8** (+2d)	**15** (+2d) **22** (±2d)	**1** (±3d)	**8, 15, 22** (±2d)	**1** (±3d)	**15** (±3d)	**≤30 d after last dose** (+7d)	**Pre-PD** Q 12 wk (±14d) **Post-PD** Q 16 wk (±28 d)
**STUDY PROCEDURES**													
ECOG performance status	See Appendix 6		X					X		X		X	
Physical examination	Symptom-directed		As clinically indicated										
Weight			X					X		X			
Neurologic examination including ICE Tool	See Section 8.3.7 and Appendix 15<br>Prior to administration of study drugs.		X	X	As clinically indicated (see Section 6.5.2)								
Vital signs including oxygen saturation	See Table 30 for timing in relation to dosing		X	X	X	X	X	X	X	X	X	X	
12-lead ECG			As clinically indicated										
**LABORATORY ASSESSMENT: See Appendix 18**													
Hematology	Must be performed ≤72 hours before dosing and laboratory values for C1D1 dose must meet Section 5.1 criteria. If criteria are not met see Section 5.1 for guidance or repeated testing requirements		X	X	X	X	X	X	X	X	X	X	
Chemistry			X	X	X	X	X	X	D15	X		X	
TSH			X										
Creatine kinase	Perform only on participants who are treated with statins and receive concurrent treatment with lenalidomide, from Cycle 2 to Cycle 3, at a minimum.							X	D15	X	X		
HBV, HCV PCR	• For participants with history of HBV and/or HCV infection.<br>• See Section 8.3.5.3		X							C4, C7, C10, C13, C16		3 and 6 months (±1 month) after last dose	

Table 65: Schedule of Activities for Treatment Phase, EOT Visit, and Posttreatment Follow-up Phase – Arm A, Arm B, and Arm C - Tec-DR Maintenance

Assessments	Notes	Pre Maintenance (Arm A or Arm B only)	Maintenance Treatment Phase (28-day cycle)										Follow-up Phase (Including Potential SoC Maintenance Treatment)
**Maintenance Cycle**			**Cycle 1**					**Cycle 2**		**Cycles 3-18**		**EOT**	
**Day**		**Within 28 d prior to starting maintenance**	**1**	**2**	**4** (+2d)	**8** (+2d)	**15** (+2d) **22** (±2d)	**1** (±3d)	**8, 15, 22** (±2d)	**1** (±3d)	**15** (±3d)	**≤30 d after last dose** (+7d)	**Pre-PD** Q 12 wk (±14d) **Post-PD** Q 16 wk (±28 d)
Coagulation			As clinically indicated (including at CRS onset if applicable)										
Urine pregnancy test	• Females of childbearing potential<br>• Additional testing may be required per the local PPP<br>• Serum pregnancy test is acceptable		• Within 24 hours of C1D1.<br>• 10 to 14 days prior and again within 24 hours prior to first dose of lenalidomide.<br>• Weekly for first 4 weeks of lenalidomide treatment, thereafter, every 4 weeks (every 2 weeks for women with irregular menses) and as clinically indicated.									X	
**STUDY VISITS: See Section 6.4.1 for required safety monitoring through the first treatment dose of teclistamab & Appendix 13 for considerations for outpatient administration during this period**													
Study visit	If a visit occurs later than planned, adjust subsequent visit(s) accordingly or skip dose as per Table 47		X	X	X	X	X	X	X	X	X	X	X
**DISEASE EVALUATIONS: See Section 8.2 for details regarding evaluations and Section 8.1.1.6 for guidance regarding follow-up requirements.**													
Quantitative immunoglobulins	Central laboratory (local laboratory assessments may be used under specified circumstances [see Section 8.1.2]). The sample for C1D1 may be collected on or within 3 days prior to C1D1.	X	X					X		X		X (until confirmed PD or SST, whichever occurs earlier)<br><br>**NOTE**: SPEP and UPEP assessments will continue until 30 days after the start of the first SST for participants with no PD or unconfirmed PD (ie, single instance of PD by laboratory assessment) while on treatment. For participants with measurable disease by light chain, serum FLC will also continue during this period.	
SPEP		X	X					X		X			
24-hour UPEP		X	X					X		X			
Serum FLC		X	• For participants with measurable disease by light chain: Day 1 of each cycle<br>• Others: Whenever CR or sCR is suspected										
SIFE/UIFE		X	X	Day 1 of each cycle and whenever CR or sCR are suspected									
Imaging for disease evaluation	See Section 8.2.4 for acceptable modalities		• As clinically indicated to document response or progression										
Assessment of soft-tissue plasmacytomas	See Section 8.2.5 for acceptable modalities and instructions regarding biopsies		• For participants with a history of soft-tissue plasmacytoma<br>-For assessment by physical examination (if applicable), every 4 weeks (±1 week) until development of confirmed CR or PD or start of SST<br>-For assessment by radiology, every 12 weeks (±1 week) until confirmed CR or PD or start of SST<br>• As clinically indicated for other participants										

Table 65: Schedule of Activities for Treatment Phase, EOT Visit, and Posttreatment Follow-up Phase – Arm A, Arm B, and Arm C - Tec-DR Maintenance

Assessments	Notes	Pre Maintenance	Maintenance Treatment Phase (28-day cycle)										Follow-up Phase
Maintenance Cycle		(Arm A or Arm B only)	Cycle 1					Cycle 2		Cycles 3-18		EOT	(Including Potential SoC Maintenance Treatment)
Day		Within 28 d prior to starting maintenance	1	2	4 (+2d)	8 (+2d)	15 (+2d) 22 (±2d)	1 (±3d)	8, 15, 22 (±2d)	1 (±3d)	15 (±3d)	≤30 d after last dose (+7d)	Pre-PD<br>Q 12 wk (±14d)<br>Post-PD<br>Q 16 wk (±28 d)
Bone marrow aspirate	See Table 60 (a portion of aspirate should be sent to the central laboratory for MRD assessment)	X (for MRD sampling)	• For participants who have not progressed, and remain on study, an additional bone marrow aspirate will be obtained at 6, 12, 18, and 24 months (±1 month) post start of maintenance. For participants who remain on study drug, cycles should be used to guide MRD sampling (ie, 6 months is after Maintenance Cycle 6)<br>• Time of suspected CR (including sCR)<br>• Time of PD										
Imaging for MRD (DW-MRI)	Optional for sites where available.<br>See Section 8.2.4.1	X	• After 18 cycles of maintenance										
**ONGOING REVIEW**													
AEs			Continuous until 30 days after last dose of study treatment or until the start of subsequent therapy, whichever comes first. Continue to report any AEs/SAEs related to study treatment until EOS.										
2nd primary malignancy			Continuous until EOS										
Concomitant therapy	See Section 6.12		Continuous until 30 days after last dose of study treatment or until the start of SST, whichever comes first. Continue to report concomitant therapy given for any AEs/SAEs considered related to study treatment until EOS										
SoC Maintenance	Per institutional standard and local investigator decision.<br>See Section 8.1.1.6.1												If applicable, document treatment regimen and reason for discontinuation or changes in SoC maintenance
SST	See Section 6.13. SoC maintenance without PD is not regarded as SST												X (post-PD)
Survival			Continuous										X

Table 66: Schedule of PK, Immunogenicity, and Biomarker Sample Collection for Arm A Tec-DRd Induction

Assessment	Time	Induction Treatment Phase (28-day cycle)										Follow-up Phase (if not continued on main-tenance)
**Induction Cycle**		**Cycle 1**					**Cycles 2-6 (stem cell collection after Cycle 3 as per Section 8.1.1.3.1)**		**End of Induction**	**HDT + ASCT**	**EOT (if not continued on main-tenance)**	
**Day**		**1**	**2**	**4** (+2d)	**8** (+2d)	**15** (+2d) **22** (±2d)	**1** (±3d)	**8, 15, 22** (±7d)	**2 weeks after completing Cycle 6**	Limited data collection (Section 8.1.1.3.3)	**≤30d after last dose**	**Pre-PD** Q 12 wk (±14d) **Post-PD** Q 16 wk (±28 d)
**PHARMACOKINETICS AND IMMUNOGENICITY SAMPLING**												
Teclistamab	Prior to first dose of daratumumab (-2 h)	X										
	Predose (prior to teclistamab dose) on day of dosing				X	D15 (PK only)	C3 and C6				X	8 weeks after EOT
	Suspected sARR Grade ≥2	Collect additional PK/immunogenicity sample as soon as sARR is detected, if feasible										
	Suspected CRS or teclistamab-related neurotoxicity Grade ≥2	Collect additional PK/immunogenicity sample as soon as CRS or neurotoxicity is detected, if feasible										
**BIOMARKER SAMPLING (BLOOD): See Section 8.7. For bone marrow aspirate, see Table 3, Table 4, and Table 60 for Disease Evaluation**												
Immunopheno-typing (whole blood)[a]	Time-based	X										
		• In all participants, along with bone marrow collection, after completion of Induction Cycle 3 (before mobilization) in participants with a response of VGPR or better and after Induction Cycle 6, irrespective of response										
	Response-based	• Time of suspected CR or sCR (unless this occurs within 1 month of another sample)<br>• Time of PD										
Molecular markers (whole blood)[a]	Time-based	X										
		• In all participants, along with bone marrow collection, after completion of Induction Cycle 3 (before mobilization) in participants with a response of VGPR or better and after Induction Cycle 6, irrespective of response .										
	Response-based	• Time of suspected CR or sCR (unless this occurs within 1 month of another sample)<br>• Time of PD										
Biopsy of soft-tissue plasmacytomas	As clinically indicated	See Section 8.2.5										

a. All collections should be predose.

Table 67: Schedule of PK, Immunogenicity, and Biomarker Sample Collection for Arm C - Tec-DR Maintenance

Assessments	Time	Maintenance Treatment Phase (28-day cycle)										Follow-up Phase (Including Potential SoC Maintenance Treatment)
Maintenance Cycle		Cycle 1					Cycle 2		Cycles 3-18		EOT	
Day		1	2	4 (+2d)	8 (+2d)	15 (+2d), 22 (±2d)	1 (±3d)	8, 15, 22 (±2d)	1 (±3d)	15 (±7d)	≤30d after last dose	Pre-PD Q 12 wk (±14 d) Post-PD Q 16 wk (±28 d)
**PHARMACOKINETICS AND IMMUNOGENICITY SAMPLING**												
Teclistamab	Prior to first dose of daratumumab (-2 h)	X										
	Predose (prior to teclistamab dose) on day of dosing				X	D15 (PK only)	X (PK only)		C3, C6, C12, C18		X	8 weeks after EOT
	Suspected sARR Grade ≥2	Collect additional PK/immunogenicity sample as soon as sARR is detected, if feasible										
	Suspected CRS or teclistamab-related neurotoxicity event Grade ≥2	Collect additional PK/immunogenicity sample as soon as CRS or neurotoxicity is detected, if feasible										
Daratumumab	Predose (prior to daratumumab dose) on day of dosing	X					X		C6			
**BIOMARKER SAMPLING (BLOOD): See Section 8.7. For bone marrow aspirate, see Table 60 for Disease Evaluation**												
Immunopheno-typing (whole blood)[a]	Time-based	X										
	Response-based	• For participants who have not progressed and remain on study, at 6, 12, 18, and 24 months (±1 month) post start of maintenance<br>• Time of suspected CR or sCR (unless this occurs within 1 month of another sample)<br>• Time of PD										
Molecular markers (whole blood)[a]	Time-based	X										
	Response-based	• For participants who have not progressed and remain on study, at 6, 12, 18, and 24 months (±1 month) post start of maintenance<br>• Time of suspected CR or sCR (unless this occurs within 1 month of another sample)<br>• Time of PD										
Biopsy of soft-tissue plasmacytomas	As clinically indicated	See Section 8.2.5										

a. All collections should be predose.

10.22.2. Justification for Treatment Dose

10.22.2.1. Treatment Dose and Schedule for Teclistamab

Arm A (Induction Treatment) and Arm C (Maintenance Treatment)

For induction treatment, the teclistamab SC dose schedule is 2 step-up doses (0.06 and 0.3 mg/kg) followed by a weekly treatment dose of 1.5 mg/kg through Cycle 6. For maintenance treatment, the teclistamab SC dose schedule consists of 2 step-up doses (0.06 and 0.3 mg/kg) followed by a weekly treatment dose of 1.5 mg/kg in Cycles 1 and 2, followed by a 3 mg/kg dose Q2W in Cycles 3 through 18 to increase convenience and flexibility for patients, caregivers, and healthcare providers. Given the teclistamab PK, it is predicted that 3[redacted] dosing will provide comparable exposure to that of 1.5 mg/kg weekly with adequate exposure over the intended dosing interval (Figure 13). The step-up and treatment doses of teclistamab were selected after review of safety, efficacy, PK, and pharmacodynamic data from participants with relapsed or refractory multiple myeloma treated with this dose as a monotherapy in MajesTEC-1. As summarized in Section 2.1.4.1, the safety profile of teclistamab monotherapy in MajesTEC-1, [redacted], was consistent with the mechanism of action with respect to T-cell activation and targeting of B cells. CRS was frequent, but generally of low grade and short in duration. Neurotoxicity (including ICANS) occurred at low frequency and was of low grade. Among the 150 participants treated with teclistamab 1.5 mg/kg, the RP2D dose, who were response evaluable, ORR was robust. Furthermore, the safety profile of teclistamab [redacted] and [redacted] appears consistent with that for lower dose levels, although analysis for the [redacted] weekly dose level is limited by short duration of follow-up. The proposed dose of teclistamab to be used in the combination therapy in this study is 25% of the highest dose evaluated as monotherapy.

Trough levels following the 1.5 mg/kg SC injections are comparable to the maximum EC_{90} of the ex vivo cytotoxicity assay (Figure 13). This assay assessed the ability of teclistamab to induce killing using mononuclear cells from the bone marrow samples of patients with multiple myeloma in co-culture with T cells from healthy donors. Pharmacodynamic data also support this regimen with induction of cytokines and T cell activation following step-up doses indicative of the mechanism of action.

In MajesTEC-1, teclistamab exposure increased in a dose-proportional manner over the range of 0.08 to [redacted] administration. Given the robust PK data and the tolerable safety data in all cohorts evaluated to date (see Section 2.1.4.1.1), and comparable efficacy data at dose levels ranging from 0.72 to [redacted] mg/kg weekly teclistamab, this SC dose schedule of 1.5 mg/kg teclistamab weekly for 2 cycles and 3 mg/kg teclistamab Q2W thereafter is predicted to provide comparable exposure with the 1.5 mg/kg teclistamab weekly dose schedule (see Figure 13). Consequently, this dose schedule is expected to achieve comparable efficacy and safety.

Figure 13: Predicted Teclistamab Concentration-time Profiles Following Teclistamab SC Dose of 1.5 mg/kg Weekly (C1-C2) Followed by 3 mg/kg Q2W in Cycle 3+ Versus Teclistamab SC Dose of 1.5 mg/kg Weekly

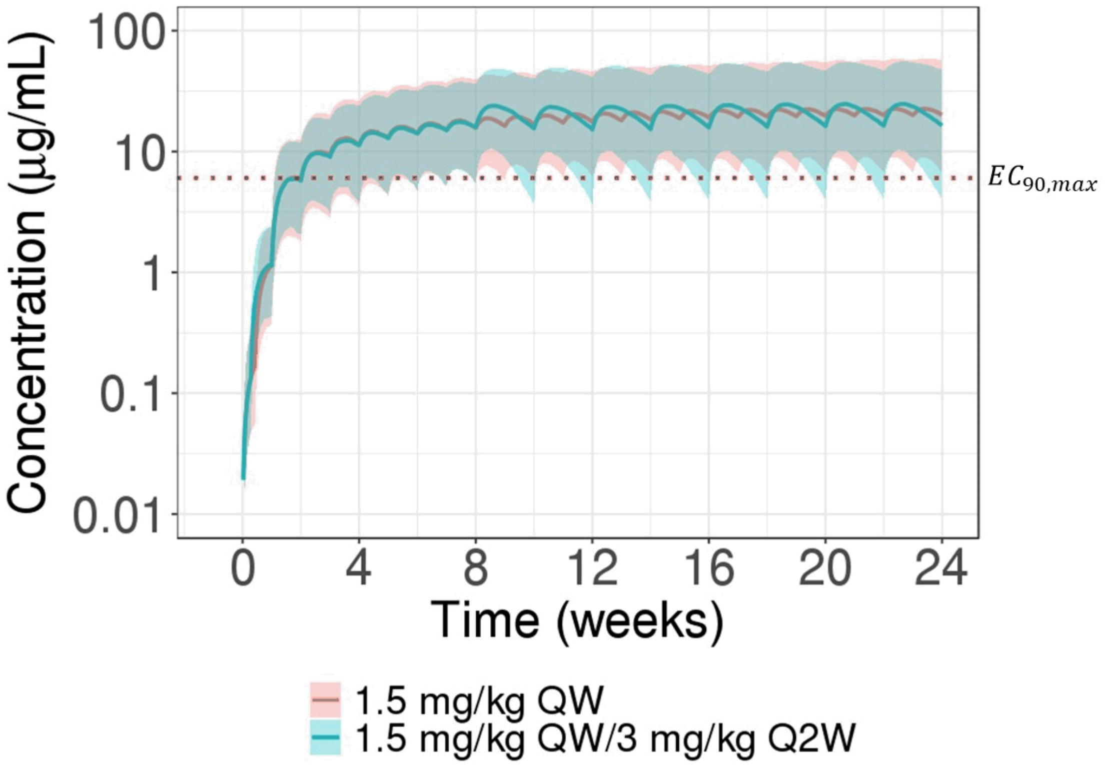

$EC_{90\ max}$=maximum EC_{90} (upper bound concentration of 90% maximum effect)
Step-up dosing: 0.06 and 0.3 mg/kg

The population PK analysis used serum teclistamab concentration data from MajesTEC-1 with the PK data cutoff of 14 June 2021. The final dataset included a total of 4,143 measurable serum teclistamab concentrations from 308 PK-evaluable adult participants (n=83 received IV dose; n=225 received SC dose) with relapsed or refractory multiple myeloma. The weight distribution in this study population ranged from 41 to 139 kg, with a median body weight of 74 kg. The final population PK model was used to simulate teclistamab concentration-time profiles. The participants for simulation were randomly sampled from the analysis dataset (n=1,000).

As shown in Figure 14, 3 mg/kg teclistamab SC Q2W dosing is predicted to result in a majority of participants with C_{trough} above the maximum EC_{90} (target exposure) at steady-state, which was comparable with 1.5 mg/kg teclistamab SC weekly dosing. Following 3 mg/kg teclistamab SC Q2W dosing, the model predicted that steady-state median C_{trough}, C_{max}, and AUC_{tau} to be comparable with those of 1.5 mg/kg teclistamab SC weekly dosing (Table 68). The accumulation ratio following teclistamab SC Q2W dosing is expected to be less than half of weekly 1.5 mg/kg teclistamab SC dosing.

Figure 14: Predicted Teclistamab Steady-state PK Parameters for 3 mg/kg Q2W SC Dosing (Cycle 3+) Versus 1.5 mg/kg Weekly SC Dosing

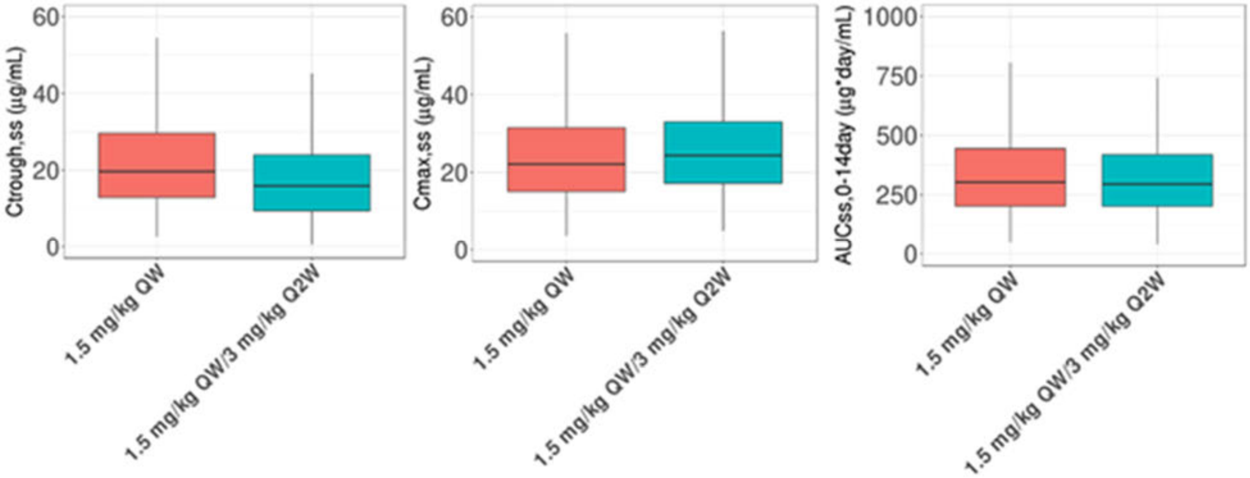

$AUC_{ss,\ 0\text{-}14\ day}$=area under the serum concentration versus time curve during a dose interval time period (14 days) at steady-state; AUC_{ss}=area under the serum concentration versus time curve during a dose interval time period (tau) at steady-state; $C_{max,ss}$=maximum concentration at steady state; $C_{trough,ss}$=trough concentration at steady state; ss=steady-state.

NOTE: 1.5 mg/kg QW $AUC_{ss,0\text{-}14}$ was calculated as 2×AUC_{ss}.

NOTE: 1.5 mg/kg weekly in Cycles 1 – 2, followed by 3 mg/kg Q2W in Cycle 3+. PK parameters for 3 mg/kg Q2W dosing are presented.

Table 68: Model-predicted Median (5th-95th Percentiles) Steady-state For the Two Dose Schedules

Dose	$C_{trough,ss}$ (µg/mL)	$C_{max,ss}$ (µg/mL)	$AUC_{ss,0\text{-}14\ day}$ (µg×day/mL)
1.5 mg/kg QW	20.1 (6.75, 57.4)	22.7 (9.04, 59.2)	307 (116, 821)
1.5 mg/kg QW / 3 mg/kg Q2W	16.2 (3.42, 49.8)	25.2 (10.7, 59.6)	300 (118, 785)

$AUC_{ss,\ 0\text{-}14\ day}$=area under the serum concentration versus time curve during a dose interval time period (14 days) at steady-state; C_{max}=maximum concentration; C_{trough}=trough concentration ss=steady-state

NOTE: 1.5 mg/kg QW $AUC_{ss,0\text{-}14}$ was calculated as 2×AUC_{ss}.

NOTE: 1.5 mg/kg weekly in Cycles 1 – 2, followed by 3 mg/kg Q2W in Cycle 3+. PK parameters for 3 mg/kg Q2W dosing are presented.

Notably, the safety profile of teclistamab in combination with daratumumab SC in TriMM-2 (including teclistamab 3 mg/kg Q2W dose schedule) appears to be consistent with those of the individual study drugs with no deleterious effects on the incidence or severity of CRS, sARR, neurotoxicity or other potential clinically important overlapping toxicities (ie, injection-site reactions, cytopenias, or infections; see Section 2.1.4.1.2). Overall, teclistamab SC dose schedule of 1.5 mg/kg weekly for 2 cycles and 3 mg/kg Q2W thereafter is predicted to provide comparable exposure and hence comparable safety with the 1.5 mg/kg teclistamab weekly dose schedule. Response data in this study are preliminary but suggest a promising effect of teclistamab in combination with daratumumab SC.

In addition, preliminary data from MajesTEC-2 (Section 2.1.4.1.3) support the proposed dosing regimen for teclistamab in combination with daratumumab SC, lenalidomide, and bortezomib.

10.22.3. Study Treatment Administered

The information included in this section is for reference only. Additional information on treatment schedules in Arms A and C was described in Protocol Amendment 2 (17 October 2022). For current dosing schedule of Arms A and C participants refer to Section 1.2 and Section 6.1.

Figure 15: Schematic of Dose Schedule – Arm A

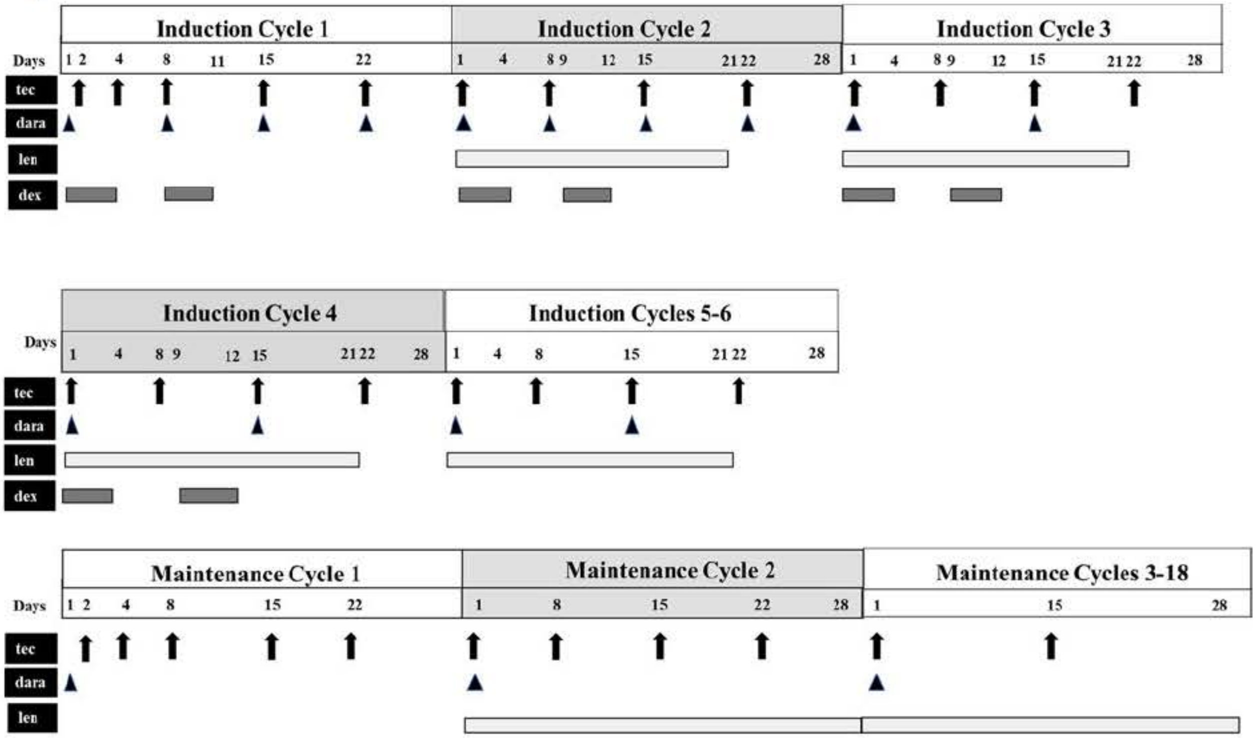

tec=teclistamab; dara=daratumumab; len=lenalidomide; dex=dexamethasone
NOTE: Arrows indicate a teclistamab dosing day and a triangle indicates a daratumumab dosing day.

Table 69: Study Treatment Administered – Overview – Arm A

Induction

Study Treatment	Induction Cycle (each cycle=28 days)	Dose Schedule
Teclistamab SC	Cycle 1	Step-up Dose 1 (0.06 mg/kg): Day 2<br>Step-up Dose 2 (0.3 mg/kg): Day 4<br>Treatment Dose (1.5 mg/kg): Days 8, 15, 22
	Cycles 2 to 6	1.5 mg/kg Weekly Treatment Dose on Days 1, 8, 15, 22
Daratumumab SC (1800 mg)	Cycles 1 and 2	Weekly dose on Days 1, 8, 15, 22
	Cycles 3 to 6	Q2W dose on Days 1 and 15
Lenalidomide PO (25 mg)	Cycle 1	No dosing
	Cycles 2 to 6	Days 1-21 from Cycle 2 onwards

Study Treatment	Induction Cycle (each cycle=28 days)	Dose Schedule
Dexamethasone PO/IV (20 mg)	Cycle 1	On Days 1-4 and 8-11
	Cycles 2-4	Days 1-4 and 9-12
	Additional dexamethasone will be administered as pretreatment medication as described in Section 6.2.	

Maintenance

Study Treatment	Maintenance Cycle (each cycle=28 days)	Dose Schedule
Teclistamab SC	Cycle 1	Step-up Dose 1 (0.06 mg/kg): Day 2<br>Step-up Dose 2 (0.3 mg/kg): Day 4<br>Treatment Dose (1.5 mg/kg): Days 8, 15, 22
	Cycle 2	1.5 mg/kg Weekly Treatment Dose on Days 1, 8, 15, 22
	Cycles 3 to 18	3 mg/kg Q2W Treatment Dose on Days 1 and 15
Daratumumab SC (1800 mg)	Cycles 1 to 18	On Day 1 of each cycle
Lenalidomide PO (10 mg)	Cycles 2 to 18	Lenalidomide 10 mg daily on Days 1 to 28; may increase to 15 mg daily starting Cycle 5 Day 1 if tolerated

Figure 16: Schematic of Dose Schedule – Arm C

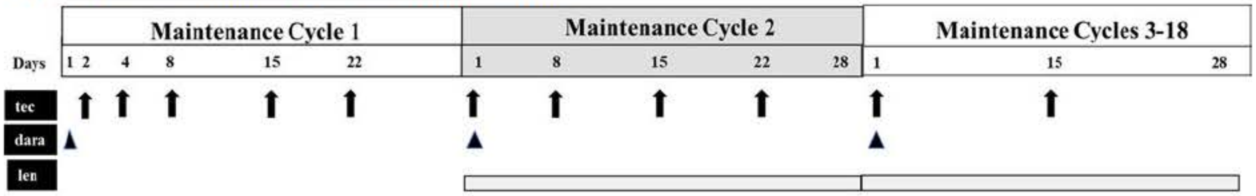

Abbreviations: dara=daratumumab, len=lenalidomide; tec=teclistamab
NOTE: in the figure, the arrows indicate a teclistamab dosing day and a triangle indicates a daratumumab dosing day.

Table 70: Study Treatment Administered – Overview – Arm C

Study Treatment	Maintenance Cycle (each cycle=28 days)	Dose Schedule
Teclistamab SC	Cycle 1	Step-up Dose 1 (0.06 mg/kg): Day 2<br>Step-up Dose 2 (0.3 mg/kg): Day 4<br>Treatment Dose (1.5 mg/kg): Days 8, 15, 22
	Cycle 2	1.5 mg/kg Weekly Treatment Dose on Days 1, 8, 15, 22
	Cycles 3 to 18	3 mg/kg Q2W Treatment Dose on Days 1 and 15
Daratumumab SC (1800 mg)	Cycles 1 to 18	On Day 1 of each cycle
Lenalidomide PO (10 mg)	Cycles 2 to 18	Lenalidomide 10 mg daily on Days 1 to 28; may increase to 15 mg daily starting Cycle 5 Day 1 if tolerated

Table 71: Dose Schedule and Pretreatment Medications for Arm A Tec-DRd Induction

**Activity**	**Notes**	**Induction Treatment Phase (28-day cycle)**						
**Induction Cycle**		**Cycle 1**					**Cycle 2**	**Cycles 3-6**
**Day**		**1**	**2**	**4**	**8**	**15, 22**	**1, 8, 15, 22**	**1, 8, 15, 22**
**REQUIRED PRETREATMENT MEDICATIONS: See Section 6.2.1 for event-driven required pretreatment medications. From Cycle 2 onwards, all oral pretreatment medications may be administered at home, provided they are taken within the timeframes specified below.**								
Dexamethasone 20 mg or equivalent (see Appendix 10)	• Oral/IV: administer 1-3 hours (±15 min) prior to administration of (first) study drug.<br>• On Cycle 1 Day 8 and per Table 24, a second full dose must be given prior to teclistamab if >4 hours have elapsed since prior administration of dexamethasone (20 mg).<br>• On Days 1, 2, 4, and 8 of Cycle 1, backbone dexamethasone substitutes for premedication.	X	X	X	X	Dexamethasone should not be administered as pretreatment medication after Cycle 1 Day 8 except as described in Section 6.2.1 or upon discussion with the sponsor.		
Diphenhydramine 25 to 50 mg, or equivalent	• Oral/IV: administer 1-3 hours (±15 min) prior to administration of (first) study drug.<br>• On Cycle 1 Day 8 and per Section 6.2.1, a second full dose must be given prior to teclistamab if >6 hours have elapsed since prior administration.	X	X	X	X	X	X	Day 1 and 15 only
Acetaminophen 650 to 1000 mg or equivalent		X	X	X	X	X	X	Day 1 and 15 only
**OPTIONAL PRETREATMENT MEDICATIONS: Additional pretreatment medications such as H_2-antagonists or antiemetics may be used per investigator discretion.**								
Montelukast 10 mg	Oral: 1-3 hours (±15 min) prior to administration of daratumumab SC.	Per investigator discretion prior to administration of daratumumab SC						
**STUDY DRUGS: See Table 24 for additional detail, including the order and timing of administration of daratumumab SC and teclistamab. If they occur, CRS (fever, hypoxia, and hypotension) and ICANS must fully resolve before the next administration of teclistamab (see also criteria in Section 6.9.4.1). See Section 6.9.1.3 for information regarding acceptable windows for study drug administration.**								
Daratumumab 1800 mg SC	• There must be ≥5 days between each dose of daratumumab SC..	X			X	X	X	Day 1 and 15 only
Teclistamab Step-up Dose	• Administer by SC injection.<br>• Step-up Dose 1: Must be administered ≥20 hours after daratumumab SC administered on Cycle 1 Day 1.<br>• Thereafter, there must be ≥2 days between step-up doses.		0.06 mg/kg	0.3 mg/kg				
Teclistamab Treatment Dose	• Administer by SC injection.<br>• The first treatment dose of teclistamab must be administered ≥2 days after step-up dose 2.<br>• Thereafter, there must be ≥5 days between each treatment dose.				1.5 mg/kg	1.5 mg/kg	1.5 mg/kg	1.5 mg/kg
Lenalidomide 25 mg	• Oral administration<br>• Lenalidomide will not be administered during Cycle 1; it will start on Day 1 of Cycle 2<br>• Lenalidomide dose may need to be adjusted based on CrCl. See Section 6.9.4.2 and Table 56.						Days 1-21 Cycles 2-6	
Dexamethasone 20 mg	• Oral/IV: administer 1 hour (±15 min) prior to administration of daratumumab SC. Dispense on Day 1 for self- administration	• On Days 1-4 and 8-11 for Cycle 1 only. On Days 1-4 and 9-12 for Cycles 2-4 only.<br>• Additional dexamethasone may be administered as pretreatment medication as described in Section 6.2.						

Table 72: Dose Schedule and Pretreatment Medications for Arm C Tec-DR Maintenance

Activity		Maintenance Treatment Phase (28-day cycle)							
**Maintenance Cycle**	**Notes**	**Cycle 1**						**Cycle 2**	**Cycles 3-18**
**Day**		**1**	**2**	**4**	**8**	**15**	**22**	**1, 8, 15, 22**	**1, 15**
**REQUIRED PRETREATMENT MEDICATIONS: See Section 6.2.1 for event-driven required pretreatment medications.**									
Dexamethasone 20 mg or equivalent (see Appendix 10)	• Oral/IV: administer 1-3 hours (±15 min) prior to administration of (first) study drug.<br>• On Cycle 1 Day 8 and per Table 30 a second full dose must be given prior to teclistamab if >4 hours have elapsed since prior administration or dexamethasone.	X	X	X	X	Dexamethasone should not be administered as pretreatment medication after Cycle 1 Day 8 except as described in Section 6.2.1 or upon discussion with the sponsor.			
Diphenhydramine 25 to 50 mg, or equivalent	• Oral/IV: administer 1-3 hours (±15 min) prior to administration of (first) study drug.<br>• On Cycle 1 Day 8 and per Section 6.2.1, a second full dose must be given prior to teclistamab if >6 hours have elapsed since prior administration.	X	X	X	X	X	X	Day 1 only	Day 1 only
Acetaminophen 650 to 1000 mg, or equivalent		X	X	X	X	X	X	Day 1 only	Day 1 only
**OPTIONAL PRETREATMENT MEDICATIONS: Additional pretreatment medications such as H_2-antagonists or antiemetics may be used per investigator discretion.**									
Montelukast 10 mg	Oral: 1-3 hours (±15 min) prior to administration of daratumumab SC.	Per investigator discretion prior to administration of daratumumab							
**STUDY DRUGS: See Table 30 for additional detail, including the order and timing of administration of daratumumab SC and teclistamab. If they occur, CRS (fever, hypoxia, and hypotension) and ICANS must fully resolve before the next administration of teclistamab (see also criteria in Section 6.9.4.1). See Section 6.9.1.3 for information regarding acceptable windows for study drug administration**									
Teclistamab Step-up Dose	• Administer by SC injection.<br>• Step-up Dose 1: Administer on Cycle 1 Day 2; must be administered ≥20 hours after daratumumab SC given on Cycle 1 Day 1.<br>• Thereafter, there must be ≥2 days between step-up doses.		0.06 mg/kg	0.3 mg/kg					
Teclistamab Treatment Dose 1.5 mg/kg	• Administer by SC injection.<br>• The first treatment dose of teclistamab must be administered ≥2 days after step-up dose 2.<br>• Thereafter, there must be ≥5 days between each treatment dose in Cycles 1-2.				1.5 mg/kg	1.5 mg/kg	1.5 mg/kg	1.5 mg/kg	
Teclistamab Treatment Dose 3 mg/kg	• Administer by SC injection.<br>• There must be at least 14 days +/- 2 days between each treatment dose in Cycles 3+.								3 mg/kg
Daratumumab 1800 mg SC		X						Day 1 only	Day 1 only
Lenalidomide 10 mg[a]	• Oral administration<br>• Lenalidomide will not be administered during Cycle 1; it will start on Day 1 of Cycle 2.<br>• Lenalidomide dose may need to be adjusted based on CrCl. See Section 6.9.4.2 and Table 56.							• Starting with Cycle 2 Day 1: 10 mg daily for 28 days<br>• May increase to 15 mg daily starting Cycle 5 Day 1 if tolerated	

10.22.4. Skipping of Study Drugs

Table 73: Teclistamab and Daratumumab SC Dose Skips During Arm A Tec-DRd and Arm B Tec-DVRd Induction

Study Drug(s)	Cycles	Dosing Frequency	Skip Dose if Dosing Interrupted:	Resume Dosing
Teclistamab step-up doses	Cycle 1	See Table 71	May not be skipped	
Teclistamab treatment doses	All cycles	Weekly	>3 days	Next planned weekly dosing day
Daratumumab SC	Cycles 1-2	Weekly	>3 days	Next planned weekly dosing day
	Cycle 3-6	Q2W	>7 days	Next planned Q2W dosing day

Table 74: Teclistamab and Daratumumab SC Dose Skips During Arm A, Arm B, and Arm C Tec-DR Maintenance

<table>
<tr><th colspan="2">Study Drug(s)</th><th>Cycles</th><th>Dosing Frequency</th><th>Skip Dose if Dosing Interrupted:</th><th>Resume Dosing</th></tr>
<tr><td rowspan="3">Teclistamab</td><td>Step-up doses</td><td>Cycle 1</td><td>See Table 72</td><td colspan="2">May not be skipped</td></tr>
<tr><td>1.5 mg/kg weekly</td><td>Cycles 1-2</td><td>Weekly</td><td>>3 days</td><td>Next planned weekly dosing day</td></tr>
<tr><td>3 mg/kg Q2W</td><td>Cycles 3+</td><td>Q2W</td><td>>7 days</td><td>Next planned Q2W dosing day</td></tr>
<tr><td colspan="2">Daratumumab SC</td><td>Cycle 1-18</td><td>Monthly</td><td>>14 days</td><td>Next planned monthly dosing day</td></tr>
</table>

10.23. Appendix 23: Protocol Amendment History

The Protocol Amendment Summary of Changes Table for this amendment is located directly before the Table of Contents (TOC).

Amendment 4 (13 March 2024)

Overall Rationale for the Amendment: The overall rationale for this amendment is to add new treatment arms to evaluate: (1) [redacted] therapy as replacement for HDT+ASCT following Tec-DRd induction therapy (Arm [redacted]); (2) HDT+ASCT per SoC with [redacted] induction therapy and Tec-D (Arm [redacted]) or [redacted] (Arm [redacted]) maintenance therapy; and (3) [redacted] as maintenance therapy after, per SoC, induction and high-dose chemotherapy plus ASCT (Arm [redacted]). Potential arms [redacted] to evaluate HDT+ASCT per SoC with [redacted] induction therapy and Tec-D (Arm [redacted]) or [redacted] (Arm [redacted]) maintenance therapy have also been added to the protocol; the decision to open these arms will be based on emerging data from Arms A1 and B for Tec-DRd and Tec-DVRd induction, and Arms [redacted] and [redacted]1 for [redacted] induction. Additionally, the maintenance treatments planned for existing arms have been updated to evaluate Tec-D as a post-transplant maintenance therapy.

The changes made to the clinical protocol 64007957MMY2003 as part of Protocol Amendment 4 are listed below, including the rationale of each change and a list of all applicable sections. Changes made in previous protocol amendments are listed in: Protocol Amendment History Appendix 23.

Section Number and Name	Description of Change	Brief Rationale
Title page; 1.1. Synopsis	Updated study title.	To reflect the addition of new teclistamab- and talquetamab-based combination regimens to be evaluated in the study
	Added text on EU Regulation.	To align with EU CTR requirements.
Throughout the protocol	Added new treatment arms and teclistamab- and talquetamab-based combination regimens, as follows:<br>- Arm [redacted]: [redacted] maintenance therapy<br>- Arm [redacted]: Tec-DRd induction therapy and [redacted] therapy as replacement for HDT+ASCT<br>- Arm [redacted] induction therapy and Tec-D maintenance therapy<br>- Arm [redacted] induction therapy and [redacted] maintenance therapy<br>- Arm [redacted] (if opened based on emerging data from Arms A1, B, [redacted] induction therapy and Tec-D maintenance therapy<br>- Arm [redacted] (if opened based on emerging data from Arms A1, B [redacted]): [redacted] induction therapy and [redacted] maintenance therapy<br>Descriptions of the new arms and regimens, and associated study assessments and procedures, have been added to the relevant sections throughout the protocol (including new and updated SoA tables in Section 1.3).	Based on emerging data, Tec-D, [redacted] are anticipated to be effective antimyeloma regimens in patients with newly diagnosed multiple myeloma.
Throughout the protocol	Changed planned maintenance treatments for existing arms from Tec-DR to Tec-D in Arms A1, B, and (if opened) C1. Added that participants in existing Arms A and C initially assigned to receive Tec-DR maintenance may receive Tec-D maintenance per investigator's choice (participants who have started Tec-DR may	Based on emerging efficacy and safety data, a combination regimen of teclistamab with daratumumab SC only is anticipated to be

Section Number and Name	Description of Change	Brief Rationale
	discontinue lenalidomide to receive Tec-D per investigator's choice).	sufficient to maintain efficacy in a low tumor burden maintenance treatment setting.
1.1. Synopsis	Revised benefit-risk assessment.	To consider new study treatment regimens and shorten existing text for clarity.
1.1. Synopsis;<br>3 Objectives and Endpoints	Updated primary, secondary, and exploratory objectives and endpoints, and clinical hypothesis.	To incorporate new treatment arms and regimens, and additional exploratory objectives/endpoints for PROs
1.1. Synopsis; 4.1.1. Initiation and Expansion Rules for All Arms; 9.2. Sample Size Determination.	Updated the number of participants to be enrolled and initiation and expansion rules for the treatment arms.	To align with changes in the study conduct (ie, addition of new treatment arms)
1.1 Synopsis; 6.1 Study Treatment Administered; 6.1.1. Study Treatment Administered – Tec-DRd (Arms A, A1, or D) and Tec-DVRd (Arm B) Induction.	Updated dexamethasone dosing schedule from administration on Days 21-22 of Cycles 1-2 to Days **22-23** of Cycles 1-2.	To align with dosing schedule in other teclistamab combination studies.
1.1. Synopsis; 1.3. Schedule of Activities; 3. Objectives and Endpoints; 8.2.6. Patient-reported Outcomes; 9.4.5.5. PRO Analyses	Added PROs:<br>- CCI [redacted]	CCI [redacted]
1.1. Synopsis; 3 Objectives and Endpoints; 8.7.1. Pharmacodynamics and Exploratory Biomarker Studies	Added an optional assessment of available apheresis product to assess the presence of clonal plasma cells in the autograft; a new exploratory endpoint has been added for this assessment.	To determine the proportion of participants with clonal plasma cell-negative autograft after 3 cycles of induction with Tec-DRd, Tec-DVRd, CCI [redacted]
1.2. Schema	Updated to add new regimens and footnotes for existing figures were shortened.	To support the addition of new regimens and streamline.
1.3. Schedule of Activities, Table 2	Updated whole body CT time window from 28 to 42 days prior to study inclusion.	To align with other teclistamab studies and provide more flexibility to the planning at screening for sites and participants.
1.3. Schedule of Activities, Table 6	Updated table to reflect that Day 15 visits and activities are no longer planned after Cycle 4 of maintenance treatment.	To reduce burden for participants.

Section Number and Name	Description of Change	Brief Rationale
1.3. Schedule of Activities, Table 3, Table 4, Table 5, and Table 6; 8.2.5. Documentation of Soft-Tissue Plasmacytomas	For participants with a history of soft-tissue plasmacytoma, clarified that no further radiology assessments are needed if the plasmacytoma(s) meet CR criteria. The window for radiology assessments for these participants was also updated to ±14 days.	To provide clarification regarding follow-up of participants whose soft-tissue plasmacytoma(s) have met CR criteria.
2.1.2. Treatment Options for Patients with Newly Diagnosed Multiple Myeloma; 2.2.2. Rationale for Bispecific Antibody Therapy Combined with Daratumumab and an IMiD; 2.4.1.5. Risks of Overlapping Toxicities; 2.4.1.5.1. General; 11. References	Added key results from the Phase 3 PERSEUS study and supporting reference.	To provide recent data relevant to the study treatment regimens and rationale.
2.1.3. Bispecific Antibodies to Treat B Cell Malignancies	Added new section and figure.	To provide background on the use of bispecific antibodies for the treatment of multiple myeloma.
2.1.5. Talquetamab	Added new section providing background for talquetamab and summaries of clinical studies that are relevant to this study.	To support the addition of talquetamab-based combination regimens to the study.
2.1.4. Teclistamab	Added new section with revised text replacing the text previously included under Section 2.1.3. "B Cell Maturation Antigen".	For clarity
2.1.4.1. Summary of Clinical Studies	Updated section to provide preliminary efficacy data from the Phase 1b TriMM-2 study and information on the Phase 3 MajesTEC-4 study.<br>Streamlined section by referring to IB for some safety data where available in the current IB.	To provide further information to support the study design updates and streamline text where appropriate.
2.2. Rationale for Bispecific Antibody-based Combination Regimens	Revised rationale subsections within Section 2.2 to incorporate text to support the rationales for the new treatment regimens and streamlined these sections to reduce repetition of information.	To support study design updates and new regimens and improve readability.
2.4.1. Risks for Study Participation	Added sentence to clarify that the risks for a given participant depend on the combination of risks described for each of the components of the study treatments in the arm to which they are assigned, and any overlapping toxicities for a given combination.	To clarify the risks for study participation for participants.
2.4.1.1. Risks Associated with Teclistamab and Talquetamab	Updated section to include risks associated with talquetamab.	To provide pertinent information relevant to the addition of talquetamab-based combination regimens to the study.
2.4.1.1. Risks Associated with Teclistamab and Talquetamab; 6.5.4.3 Infection Management	Added information regarding PML to the risks section for the bispecific antibodies and guidance regarding management of infection.	To align with updates to the teclistamab label, IB, and informed consent form.

Section Number and Name	Description of Change	Brief Rationale
2.4.1.5.2. Risk of Overlapping Toxicities for Teclistamab and Talquetamab	Added new section to describe risks of overlapping toxicities for teclistamab and talquetamab.	To provide pertinent information supporting the addition of a CCI [redacted] treatment regimen to the study.
2.4.2.3. CCI [redacted]	CCI [redacted].	
2.4.2. Benefits for Study Participation	Updated the text to reflect the benefits associated with the new talquetamab-based combination regimens.	To support the addition of the new arms and talquetamab-based combination regimens to the study.
2.4.3. Benefit-Risk Assessment for Study Participation	Revised text for the overall benefit-risk assessment for study participation.	To consider the new treatment arms and regimens added to the study.
4.1.2. Scientific Rationale for Study Design	Revised text for brevity and to reflect addition of talquetamab-based combination regimens.	For clarity and to support addition of talquetamab-based combination regimens.
4.2.1. Treatment Dose and Schedule for Teclistamab	Condensed and streamlined section, with cross-references added to other sections for further information where applicable.	To improve clarity and reduce repetition in the protocol.
4.2.2. Treatment Dose and Schedule for Talquetamab	Added new section providing justification for talquetamab treatment dose and schedule.	To support the addition of talquetamab-based combination regimens to the study.
4.2.3. CCI [redacted]	CCI [redacted]	[redacted]
5.1. Inclusion Criteria	Inclusion Criterion 3: clarified that isolated total bilirubin ≥1.5xULN with conjugated (direct) bilirubin <1.5xULN is required for those participants with known **congenital nonhemolytic hyperbilirubinemia** such as Gilbert's Syndrome.	To clarify which potential participants the bilirubin value thresholds apply to.
	Inclusion Criteria 7.1 and 8: Updated the minimum time that participants must agree to use condoms when engaging in any activity that allows for passage of ejaculate to another person, and to not donate sperm, from 3 months to 100 days.	To align with talquetamab risk language.
5.2. Exclusion Criteria	Exclusion Criterion 6.1: Revised "prior autoimmune thyroiditis" to "prior autoimmune thyroid disease".	To align with teclistamab and talquetamab program level protocol language requirements and clarify the conditions that would require exclusion from the study.
	Exclusion Criterion 7: Added "unstable angina" to list of cardiac conditions. Updated appendix number referenced to Appendix 21.	
	Exclusion Criterion 10: Updated the time within which a participant plans to father a child after last dose of study treatment that would result in exclusion from the study from 3 months to 100 days.	To align with talquetamab risk language.

Section Number and Name	Description of Change	Brief Rationale
	Exclusion Criteria for Arms A, A1, B, D, E, E1, F, and F1: Revised Exclusion Criteria 5 and 6 to add participants to be enrolled in Arms F and F1 (if opened).	To reflect the addition of Arms F and F1 evaluating HDT+ASCT per SoC with [redacted]
	Exclusion Criteria for Arms C, C1, and [redacted]: Revised Exclusion Criterion 1 to add prior "GPRC5D-directed therapy" for Arm [redacted] only.	To reflect the addition of Arm [redacted] evaluating [redacted] maintenance therapy after, per SoC, induction and HDT+ASCT.
5.3. Lifestyle Considerations	Lifestyle Consideration 4.: Updated criterion to state that participants must be willing to be hospitalized or remain in close proximity (within 30 minutes) to the hospital starting after step-up dose 1 until 48 hours after the first 3 doses of teclistamab or talquetamab.<br>Lifestyle Consideration 5.: Updated to add that participants must be willing to be hospitalized for talquetamab administration following specified AEs.<br>Added Lifestyle Consideration 8.: To avoid driving or operating heavy or potentially dangerous machinery starting after step-up dose 1 until 48 hours after administration of the third dose of teclistamab or talquetamab.	To align with teclistamab and talquetamab program level protocol language.
6.1. Study Treatment Administered; 6.1.2. Study Treatment Administered – [redacted] and [redacted] Induction; 6.1.3. Study Treatment Administration – [redacted] Following Induction; 6.1.5. Study Treatment Administration – Arm [redacted] – [redacted] Maintenance; 6.1.6. Study Treatment Administration – Arms A, A1, B, C, C1, [redacted] – Tec-D Maintenance; 6.1.7. Study Treatment Administration – Arms [redacted] Maintenance	Added new tables with dose schedules and administration instructions for the [redacted] and [redacted] induction regimens, [redacted] treatment regimen, and [redacted] Tec-D, and [redacted] maintenance regimens.<br>Added the term "treatment dose" to tables for teclistamab doses that are not step-up doses.	To support the addition of new teclistamab- and talquetamab-based combination regimens to the study, and to clarify which teclistamab doses are treatment doses (ie, not step-up doses).
6.1.1. Study Treatment Administered – Tec-DRd (Arms A, A1, [redacted] and Tec-DVRd (Arm B) Induction	Updated dosing schedules to reflect that daratumumab should be administered on Days 1 and 15 only from Cycles **3**-**6**.	To correct an inconsistency in the daratumumab dosing schedule for induction treatment.

Section Number and Name	Description of Change	Brief Rationale
6.4. Required Safety Monitoring; 6.5. Management Guidelines for Potential Toxicities	Revised text to update and clarify the specific teclistamab and talquetamab doses to which the monitoring requirements and management guidelines described apply.<br>Updated Section 6.4 to note that participants who are to receive the same bispecific antibody for maintenance treatment as received for induction can be considered for outpatient dosing for the first three maintenance doses.	To clarify safety monitoring requirements and potential toxicity management guidelines for teclistamab and/or talquetamab treatment.
6.5.4.2. Vaccinations	Added respiratory syncytial virus, pneumococcal and recombinant herpes zoster vaccines as permitted vaccines.	To clarify vaccinations that are permitted.
6.5.4.5. HBV Reactivation; 8.3.5.3. Ongoing Hepatitis B Virus and Hepatitis C Virus Testing	Updated testing window to ±4 weeks.<br>Added examples of what constitutes a history of HBV infection to Section 8.3.5.3.	To align with current teclistamab program requirements for HBV and HCV testing permitting a 4-week testing window.
6.5.3. Hypogammaglobulinemia	Added a bullet point with additional guidance for IgG monitoring and supporting references.	To include guidance for accurate calculation of IgG levels.
6.5.9. Rash (Participants Receiving Talquetamab); 6.5.10. Nail Dysfunction (Participants Receiving Talquetamab); 6.5.11. Oral Toxicity (Participants Receiving Talquetamab)	Added new sections.	To provide guidance for the management of rash, nail dysfunction, and oral toxicity for participants who receive talquetamab.
6.7. Measures to Minimize Bias: Randomization and Blinding	Revised text to add new treatment arms and clarify which arms will enroll in parallel.	To clarify study design and conduct and reflect addition of new treatment arms.
6.9. Dosing Interruption and Dose Reduction	Added guidance for potential dosing interruption and dose reduction for talquetamab.<br>Clarified that “dose reduction is not permitted for daratumumab SC”.	To provide pertinent information relevant to the addition of the [redacted] regimen and talquetamab-based combination regimens to the study and clarify guidance for dose reductions.
6.9.1.3. Skipping of Study Drugs	Revised recommendations for teclistamab dosing after delays in treatment (Table 49)	To update the recommendations based on emerging data from the teclistamab program.
6.9.4.2. Lenalidomide	Updated CrCL threshold for which lenalidomide dose adjustment should be instituted to CrCl<60 mL/minute.	To align with other teclistamab combination studies.
6.12.2.6. Mitigation Measures in Case of Positive Testing for COVID-19	Added new section describing mitigation measures in case of positive testing for COVID-19.	To provide pertinent information on COVID-19 mitigation measures (to complement the information already provided in Appendix 19) and align

Section Number and Name	Description of Change	Brief Rationale
		with other teclistamab studies.
6.12.3. Prohibited or Restricted Therapies	Moved guidance regarding live attenuated vaccines from 6.12.3.1. to this section and clarified that the guidance on timing of administration of live attenuated vaccines after last dose of study treatment is applicable to teclistamab, talquetamab, and daratumumab.	To clarify guidance on live attenuated vaccines.
7.1.4. Discontinuation of lenalidomide	Added that lenalidomide should be discontinued for confirmed PML.	To align with guidance in the lenalidomide label and other teclistamab combination studies.
7.3. Lost to Follow-Up	Revised to allow site (rather than sponsor) to engage a third party to search public sources for participant vital status information.	Based on feedback from EU health authorities.
8.4.1. Time Period and Frequency for Collecting Adverse Event and Serious Adverse Event Information; 10.3 Appendix 3 subheading: 10.3.5: Adverse Event Reporting Procedures	Updated text regarding reporting of SAEs.	To align with oncology protocol template language, clarify the current safety reporting process, and ensure the protocol is gender-neutral.
8.4.4. Regulatory Reporting Requirements for Serious Adverse Events	Updated text regarding reporting of safety information.	
8.4.5. Pregnancy	Revised to remove gender terminology.	
8.4.6. Disease-Related Events and Disease-Related Outcomes Not Qualifying as Adverse Events or Serious Adverse Events	Revised descriptions of disease-related events and outcomes not qualifying as AEs or SAEs.	To align with oncology protocol template and clarify the disease-related events and outcomes that do not qualify as AEs or SAEs.
8.4.7. Adverse Events of Special Interest	Added Grade ≥3 non-ICANS teclistamab- or talquetamab-related neurotoxicity as an AESI. Updated existing AESIs of CRS and ICANS from Grade ≥2 to Grade ≥3.	To align with teclistamab and talquetamab program requirements in terms of events that are considered AESIs based on emerging data.
9.4.1. General Considerations	Added planned safety analyses for new arms and updated primary and secondary analyses accordingly. Included a statement that the sponsor may decide to modify planned analyses based on emerging data or practical considerations.	To provide pertinent information concerning planned safety analyses for the new arms.
10.2. Appendix 2 subheading: 10.2.4. Recruitment Strategy	Added new section to provide information on study recruitment strategy.	To align with EU CTR requirements.
10.2. Appendix 2 subheading: 10.2.6: Storage, Use, Transfer, and Retention of Data and Samples	Revised text to provide updated guidance on the retention of data and samples.	To align with oncology protocol template language (updated guidance on retention/usage of data

Section Number and Name	Description of Change	Brief Rationale
10.2. Appendix 2 subheading: 10.2.8. Use of Information and Publication	Revised section to provide updated guidance on the use of information and publication.	and retention of samples).
10.2. Appendix 2 subheading: 10.2.14. Record Retention	Added text regarding record retention under EU regulation, per EU CTR requirement.	To align with EU CTR requirements.
10.2. Appendix 2 subheading: 10.2.15. Study and Site Start and Closure	Updated definition of first act of recruitment. Reordered and streamlined text on study/site termination.	To align with oncology protocol template language and update the definition of the first act of recruitment in line with EU CTR.
10.3. Appendix 3 subheading: 10.3.2. Attribution Definitions	Added factors to consider when assessing causality.	To provide additional information to assist in causality assessments for AEs.
10.3 Appendix 3 subheading: 10.3.3 NCI-CTCAE Grading of Adverse Event Severity	NCI-CTCAE Grading of Adverse Event Severity appendix (formerly Appendix 20) moved to a section within the AE appendix (Appendix 3).	To consolidate related information within the same appendix.
10.5. Appendix 5: IMWG Response Criteria (2016)	The following revision was made: In addition to the above criteria, if present at baseline >90% reduction in the **sum of the products of the maximal perpendicular diameters of the measured lesions** ~~sum of the maximal perpendicular diameter~~ (SPD) compared with baseline for soft-tissue plasmacytoma.	To correct an error.
10.9. Appendix 9: Contraceptive Guidance and Lenalidomide Global/Local PPP Programs	Revised to remove gender terminology.	To align with oncology protocol template language: all oncology protocols are to be gender-neutral.
10.13. Appendix 13: Considerations for Outpatient Teclistamab or Talquetamab Administration Through the First Treatment Dose	Revised text to include talquetamab and provide updated guidance for outpatient administration.	To reflect addition of talquetamab-based combination regimens and align with teclistamab and talquetamab program requirements.
10.22. Appendix 22 subheading: 10.22.3 Study Treatment Administered	Added statement to clarify that information in this section is for information only and current dosing schedules are provided in Section 1.2 and Section 6.1.	For clarification purposes.
11. References	Updated list of references.	To reflect addition and removal of references from the protocol body.
Throughout the protocol	Minor clarifications, grammatical, formatting, or spelling changes were made.	Minor errors were noted.

Amendment 3 (06 July 2023)

Overall Rationale for the Amendment: The overall rationale for this amendment is to administer teclistamab per revised dosing regimen (Q4W dosing starting at Cycle 2), to open new treatment arms (Arm A1 and potentially Arm C1) and align with teclistamab program level protocol language.

The changes made to the clinical protocol 64007957MMY2003 as part of Protocol Amendment 3 are listed below, including the rationale of each change and a list of all applicable sections.

**Section Number and Name**	**Description of Change**	**Brief Rationale**
1.1. Synopsis	Added benefit-risk assessment section.	To comply with EU-CTR requirements.
1.1. Synopsis<br>1.2. Schema<br>6.1. Study Treatment Administered	Revised dosing regimen:<br>- teclistamab (Q4W dosing starting at Cycle 2) in all treatment arms.<br>- bortezomib (weekly) for Arm B<br>- dexamethasone (Cycles 1 and 2; Days 1-2, 8-9, 15-16 and 21-22) for induction arms.	Dose reduction of teclistamab, dexamethasone and bortezomib to optimize the safety profile.
Throughout the protocol	Arm A1 added as a new arm (Tec-DRd induction) to started in parallel to Arm B.<br>Arm C1 added as a potential new arm (Tec-DR maintenance), to be opened if deemed necessary, based on evolving data.	To investigate the revised dosing regimen in additional cohorts.
1.2. Schema	All figures in Section 1.2 have been updated to align with the revised study design and dosing schedule.	To clarify study design and conduct.
1.1. Synopsis; 1.2. Schema; 2.3.4. Rationale for Combining Teclistamab with Daratumumab SC and Lenalidomide for Maintenance; 4.1. Overall Design; 4.2.2. Rationale for Maintenance Treatment Duration;<br>4.4. End of Study Definition; 6.1. Study Treatment Administered;<br>9.4.3. Secondary Endpoints	Tec-DR maintenance treatment duration was updated to “a maximum of 18 cycles”.	To clarify study design and conduct.
1.1. Synopsis;<br>1.2. Schema; 4.1. Overall Design; 4.2.2. Rationale for Maintenance Treatment Duration;<br>6.1. Study Treatment Administered	Text added to state that Tec-DR study maintenance therapy can be discontinued when 12 months of sustained MRD negativity has been observed.	To clarify study design and conduct.

Section Number and Name	Description of Change	Brief Rationale
1.1. Synopsis; 1.2. Schema; 1.3. Schedule of Activities; 4.3.1. Treatment Dose and Schedule for Teclistamab; 6.1. Study Treatment Administered; 6.1.1. Study Treatment Administered -Tec-DRd and Tec-DVRd Induction 6.1.2. Study Treatment Administered - All Arms - Tec-DR Maintenance 6.9.1.3. Skipping of Study Drugs; 10.23. Appendix 23 Previous Schedule of Activities and Dose Regimens for Arm A and Arm C	Moved text and tables relevant to previous dosing schedules for Arm A and Arm C from the body to Appendix 23.	To add clarity and streamline the body of the protocol.
1.3. Schedule of Activities	<u>Table 2:</u> Added biomarker blood sampling for immunophenotyping and molecular markers. <u>Table 3, Table 4 and Table 5</u>: Adjusted visit windows to align with revised dosing regimen. Note added for hematology and chemistry assessments: "During the step-up phase, laboratory tests must be performed ≤24 hours prior to the second and third dose."<br><u>Table 3 and Table 4:</u> Note added to for disease evaluations: "Evaluations will also be done after completion of Cycle 3 (before mobilization procedure)" Text revised for Bone marrow aspirate and core biopsy as follows: "Sampling for MRD will be done after completion of Cycle 3 (before mobilization procedure) ~~in participants with a response of VGPR or better~~ and after completion of Cycle 6 in all participants ~~irrespective of response~~."<br><u>Table 5</u>:<br>- Note added to hematology assessments: "Participants who have not previously received lenalidomide as part of induction or consolidation should have a complete blood cell count, including white blood cell count with differential count, platelet count, hemoglobin, and hematocrit performed at baseline and every week for the first 8 weeks of lenalidomide treatment and biweekly thereafter."<br>- Updated number of visits for vital signs.<br><u>Table 6 and Table 7</u>: Revised visits for PK, immunogenicity and biomarker sampling.<br><u>Table 6</u>: Text revised for biomarker blood sampling to be performed in all participants after completion of Cycle 3 (before mobilization procedure) and after Induction Cycle 6.	To clarify study design and conduct.

Section Number and Name	Description of Change	Brief Rationale
2.1.3. B cell Maturation Antigen	Added approval information for teclistamab monotherapy.	To align with latest teclistamab approval information.
2.2. Teclistamab	Streamlined text describing mechanism of action of teclistamab.	To align with teclistamab program level protocol language.
2.2.1.1. Study 64007957MMY1001 (MajesTEC-1)	Revised based on recent available results that are relevant to this study.	To provide recent study results.
2.2.1.2. Study 64407564MMY1002 (TriMM-2);<br>2.2.1.3. Study 64007957MMY1004 (MajesTEC-2)	Updated TriMM-2 and MajesTEC-2 Study results.	To provide current study data for teclistamab treatment regimens that are relevant to this protocol.
2.2.1.4. Study 644007957MMY3005 (MajesTEC-7)	New section added to summarize safety results for Safety Run-in Cohort 1 (Tec-DR) in MajesTEC-7 Study.	To provide recent study results.
2.3.1. Rationale for Combining Teclistamab with Daratumumab SC, Lenalidomide, and Dexamethasone for Induction	Reference added to MajesTEC-7 Safety Run-in.	Clarification.
2.3.3. Rationale for Starting with Tec-DRd and Sequentially Adding a Tec DVRd Induction Arm	Rationale added for revised bortezomib dosing schedule.	To clarify rationale for weekly administration of bortezomib.
2.5.1.1. Risks Associated with Teclistamab	Table 10: Updated mitigation strategies for potential risks associated with teclistamab.	To align with teclistamab program level protocol language.
2.5.1.4. Risks Associated with Bortezomib	Reference added for weekly administration of bortezomib being better tolerated.	To clarify rationale for weekly administration of bortezomib.
2.5.3. Benefit-Risk Assessment for Study Participation	The risk-mitigation measures were updated.	To clarify management strategies for potential toxicities.
4.1.1. Initiation and Expansion Rules for All Arms	Text revised to present the number of study participants to be enrolled in each arm.	To clarify study design and conduct.
4.2.1 Study-Specific Ethical Design Considerations	Revised blood volume.	To reflect updated sampling.
4.2.2. Rationale for Maintenance Treatment Duration	References added to support the early discontinuation of study maintenance treatment after 12 months of sustained MRD negativity.	To clarify rationale for the early discontinuation of maintenance treatment.
4.3.1. Treatment Dose and Schedule for Teclistamab	Added new section justifying the treatment and dose schedule for teclistamab in revised dosing regimen.	To provide data to support the justification of teclistamab dose.
5. Study Population	Text revised.	To align with teclistamab program level protocol language.

Section Number and Name	Description of Change	Brief Rationale
5.1 Inclusion Criteria	Inclusion Criterion 3.1: Removed re-evaluation of clinical laboratory values from the text.	To align with teclistamab program level protocol language.
	Inclusion Criterion 4.1: Updated pregnancy testing requirements.	
	Inclusion Criterion 5.1: Updated contraception methods and increased timing of contraception use from 3 months to 6 months after the last dose of study treatment.	
	Inclusion Criterion 6.1: Increased the timing for egg donation or freezing for future use from 3 months to 6 months after last dose of treatment.	
	Inclusion Criterion 7.1 and 8.1: Clarity added to the use of male condom requirements and for the timing sperm donation.	
	Inclusion Criteria 1A.1 Updated criteria for defining multiple myeloma.	
	Inclusion Criterion 1B.1: Added a note that participants need to have had measurable disease at ≥1 g/dL at diagnosis.	
	Inclusion Criterion 3B.1: Requirement added for participants with plasmacytomas.	
	Inclusion Criterion 4B.1: Timeline of 6 months from transplant to enrollment: 1 additional month for patients receiving consolidation. Added 7 months for participants who received consolidation.	
5.2 Exclusion Criteria	Exclusion Criterion 1.2: Clarified malignancies to be excluded.	To align with teclistamab program level protocol language.
	Exclusion Criterion 3.1: Added transient ischemic attack and timelines to within 6 months of Cycle 1 Day 1.	
	Exclusion Criterion 5.1: Reworded Hepatitis B infection criteria. Note deleted for participants with hepatitis C infection and asthma.	
	Exclusion Criterion 6.1: Removed uncontrolled diabetes and active autoimmune disease.	
	Exclusion Criterion 14.1: Added non-live or non-replicating vaccines authorized for emergency use (eg, COVID-19) by local health authorities are allowed.	
	Arms A, A1 and B - Exclusion Criterion 4.1: Added smoldering multiple myeloma and threshold of 5% for circulating plasma cells in plasma cell leukemia.	
	Arms C and C1 - Exclusion Criterion 6.1: cumulative dose of corticosteroids received was updated to ≥40 mg dexamethasone.	
	Arms C and C1 - Exclusion Criterion 7.1: smoldering multiple myeloma added.	
5.3. Lifestyle Considerations	Increased blood donation restrictions to at least 6 months after last dose of study treatment.	To align with teclistamab program level program language.
5.4. Screen Failures	Text added: "This study will use IWRS. The investigator will not generate screening and enrollment logs directly from IWRS."	To align with late development clinical protocol template.

Section Number and Name	Description of Change	Brief Rationale
6.1. Study Treatment Administered	Table added to describe authorization status of Investigational Medicinal Product and Non-investigational Medicinal Product.	To comply with EU-CTR requirements.
	Updated dose schedule tables for each treatment arm. Text added to state that the lenalidomide regimen may be adjusted at any timepoint by the sponsor.	To clarify study design and conduct.
6.1.1. Study Treatment Administered -Tec-DRd and Tec-DVRd Induction<br>6.1.2. Study Treatment Administered - All Arms - Tec-DR Maintenance	Table 14 and Table 16: Adjusted visit windows to align with revised dosing regimen. Revised text to clarify that on Cycle 1 Day 8, an additional dose of dexamethasone (8 mg) must be given (and should be at least 15 minutes prior to teclistamab dosing) if >4 hours have elapsed since prior administration of dexamethasone (20 mg).<br>Table 14: Clarified that dexamethasone given as part of the treatment regimen will serve as pretreatment medication on Days 1, 2, 8 and 15. For bortezomib administration in Arm B, removed text stating that bortezomib regimen may be adjusted based on the safety data collected in the first 6 participants.<br>Table 16: Text added to state that the lenalidomide regimen may be adjusted at any timepoint by the sponsor.	To clarify study design and conduct.
	Table 15 and Table 17: Dexamethasone dosing instructions revised. For Safety Monitoring Requirement, severity for specified AEs for teclistamab was increased to Grade 3.	To align with teclistamab program level protocol language.
6.2. Pretreatment Medications	Note updated to state that dexamethasone should not be administered as pretreatment medication after Induction or Maintenance Cycle 1 Day 15.	To clarify study design and conduct.
6.4.1.2. Required Hospitalization for Teclistamab Dosing Following Specified Adverse Events	Table 18 revised: Grade 2 CRS or ICANS do not require hospitalization for subsequent administration of teclistamab.	To align with teclistamab program level protocol language.
6.5.1.1. Management Guidelines for CRS	Added daily monitoring of chemistry and hematology assessments. Text revised to state that additional therapy, including chemotherapy, may be considered for high-grade CRS with clinical findings overlapping with HLH/MAS.	To align with teclistamab program level protocol language.
6.5.3 Hypogammaglobulinemia	Added new section on management of hypogammaglobulinemia	To clarify guidance regarding management of hypogammaglobulinemia.
6.5.4. Infection	Added a new section.	To provide guidance for infection prophylaxis and management.
6.5.5.1. Management of sARRs Related to Daratumumab SC	Added guidance regarding the occurrence of ocular symptoms (including choroidal effusion, acute myopia, and acute angle closure glaucoma).	To align with daratumumab IB.
HBV Reactivation (previous Section 6.5.7); HCV Reactivation (previous Section 6.5.8)	Deleted section and moved relevant text to a new section (6.5.4 Infection).	To consolidate guidance for infection.
6.6.2. Preparation /Handling/Storage for	Added SC daratumumab injections will be prepared as described in the site IPPI, SmPC, USPI, local prescribing information, or equivalent documentation.	To align with daratumumab IB.

Section Number and Name	Description of Change	Brief Rationale
Other Combination Agents		
6.7. Measures to Minimize Bias: Randomization and Blinding	Text revised to clarify that participants will be assigned to Arm A1 or Arm B in parallel.	To clarify study design and conduct.
6.9. Dosing Interruption and Dose Reduction	Revised to provide guidance for teclistamab dose reductions.	To align with teclistamab program level protocol language.
6.9.1.3. Skipping of Study Drugs	Revised to provide guidance for teclistamab and daratumumab dose skip rules.	
6.9.2. Guidance for Dose Reduction	Revised to state that dose reductions or changes in frequency of teclistamab could start in Cycle 2.	
6.9.4.1 Teclistamab and Daratumumab SC	Revised teclistamab and daratumumab SC dosing interruption for non-hematologic adverse events	
6.9.4.3. Bortezomib	Removed text regarding change of bortezomib schedule to weekly.	To clarify study design and conduct.
6.11. Treatment of Overdose	Text revised for clarity.	To align with teclistamab program level protocol language.
6.12.1. Permitted Therapies	Revised text for administration of immunoglobulin replacement for hypogammaglobulinemia. Removed text on vaccination.	
6.12.2.1 Infection Prophylaxis	Removed text regarding prophylaxis for treatment for infection and referenced new section (6.5.4. Infection).	To consolidate guidance for infection prophylaxis and management.
6.12.2.3. Drugs Affecting Bone Structure and Mineralization<br>6.12.3. Prohibited or Restricted Therapies	Removed prohibition of using RANK ligand inhibitors.	To align with teclistamab program level protocol language.
6.12.2.5. Prevention of Deep Vein Thrombosis and Pulmonary Embolism	Clarified that the choice of anticoagulants should be guided by international guidelines and institutional practice.<br>.	
6.12.3. Prohibited or Restricted Therapies	Modified the criteria to administer emergency radiotherapy.	To provide flexibility in study conduct.
6.12.3.1. Prohibited and Restricted Therapies Specific to Teclistamab	Revised the time period for which CYP450 substrates with narrow therapeutic index should be administered.	To align with teclistamab program level protocol language.
6.12.3.1. Prohibited and Restricted Therapies Specific to Teclistamab<br>10.17. Appendix 17 Prophylaxis of Venous Thromboembolism	Added use of other vitamin K antagonists as anticoagulant therapy.	
6.13. Subsequent Antimyeloma Therapy	Added that the investigator should obtain approval from the sponsor if they deem it is in the best interest of a participant to start subsequent antimyeloma therapy in the absence of PD.	To align with teclistamab program level protocol language.
8.2.3. Minimal Residual Disease Evaluations<br>8.7.2. Minimal Residual Disease	Text added: "Alternative methods of MRD assessment may be additionally explored."	To clarify study design and conduct.
9.2. Sample Size Determination	Updated the number of participants.	To align with changes in the study conduct.

Section Number and Name	Description of Change	Brief Rationale
	Table 40 was added to present the widest 95% confidence interval for AE rates based on sample size.	
9.3. Populations for Analysis Sets	Text revised. Definition of 2 populations for efficacy analyses added; efficacy analysis population and responsible-evaluable population.	
9.4.1. General Considerations	Optional safety analyses were removed. Added number of participants planned to be enrolled in each arm.	
9.4.3. Secondary Endpoints	Text revised	Clarification.
10.9. Appendix 9 Contraceptive Guidance and Lenalidomide Global/Local PPP Programs	Updated contraceptive and barrier guidance.	To align with late development clinical protocol and teclistamab templates.
10.13. Appendix 13 Considerations for Outpatient Teclistamab Administration Through First Treatment Dose	Removed the following:<br>- No high tumor burden.<br>- No rapidly progressing disease.	To align with teclistamab program level protocol language.
10.20. Appendix 20 NCI-CTCAE Grading of Adverse Event Severity	Revised text regarding reporting of Grade 5 adverse events.	Revised based on feedback from health authority.
11. REFERENCES	Updated references.	Clarification.
Section 8	Section heading numbers were reorganized to avoid Level 6 Heading and above.	Clarification.
Throughout the protocol	Removed 'female' and 'male' except for CrCl calculations (Appendix 7) and descriptions of type of condom (Appendix 11). Added territory(ies) where applicable.	Clarification.
Throughout the protocol	Definitions of abbreviations at first use and underneath tables and figures have been removed.	To align with new protocol template.
Throughout the protocol	Minor grammatical, formatting, or spelling changes were made.	Minor errors were noted.

Amendment 2 (17 October 2022)

Overall Rationale for the Amendment: The overall rationale for this amendment is to provide clarification in response to feedback received from the Ethics Committee (EC): Ethikkommission der Medizinischen Fakultät der Universität Heidelberg.

The changes made to the clinical protocol 64007957MMY2003 as part of Protocol Amendment 2 are listed below, including the rationale of each change and a list of all applicable sections..

Section Number and Name	Description of Change	Brief Rationale
1.2. Schema Figure 2: Schematic Overview of Study Design	The figure has been revised for clarity on study design.	To clarify study design.
4.1.1. Initiation and Expansion Rules for	Arm C was added to section heading. The conditions to initiate and expand treatment arms was described in further detail for clarity.	

Section Number and Name	Description of Change	Brief Rationale
Arm A, Arm B and Arm C		
9.4.1. General Considerations	Text in bold was added for Arm A:<br>• After at least 6 participants have received at least 2 cycles of Tec-DRd induction treatment **with successful stem cell mobilization and collection performed in at least 3 participants after at least 3 cycles**	
10.2.1. Regulatory and Ethical Considerations	The following text was added:<br>**"The study will be performed in compliance with ICH-GCP E6 (R2) guidelines, as well as country-specific regulations, in particular Arzneimittelgesetzes (AMG) and GCP-Verordnung (GCP-V)."**	Country-specific regulations were added per EC request.
10.2.4. Data Protection	Text in bold was added:<br>These data must be collected and processed with adequate precautions to ensure confidentiality and compliance with applicable data privacy protection laws and regulations,<br>**ie, Datenschutz-Grundverordnung (DSGVO) and Bundes-/Landesdatenschutzgesetze (BDSG/LDSG).**	To respond to EC request.

Amendment 1 (07 October 2022)

Overall Rationale for the Amendment: The overall rationale for this amendment is to provide clarification in response to feedback received from the Paul Ehrlich Institute (PEI).

The changes made to the clinical protocol 64007957MMY2003 as part of Protocol Amendment 1 are listed below, including the rationale of each change and a list of all applicable sections.

In the description of change column, modified text is presented as follows: text in strikethrough has been deleted, text in bold has been added.

Section Number and Name	Description of Change	Brief Rationale
1.3. Schedule of Activities	Table 3 and Table 4 revised to include chemistry assessments on Day 1 and Day 15 during induction Cycles 2 to 6.<br>Table 3, Table 4 and Table 5 updated to include "Thyroid stimulating hormone" and "Creatine kinase"	To provide clarity to study conduct and to address concerns raised by the PEI.
5.1. Inclusion Criteria (#10)<br>8.4. Adverse Events, Serious Adverse Events, and Other Safety Reporting	Any reference to the "participant's legally acceptable representative" has been removed.	To align with the requirements of the German Medicinal Products Act § 41 (3) per PEI request.

Section Number and Name	Description of Change	Brief Rationale
5.2. Exclusion Criteria (#1.b)	The exclusion criterion was reworded as follows:<br>"Skin cancer ~~(non-melanoma or melanoma)~~ **(non-melanoma skin cancers treated with curative therapy or localized melanoma treated with curative surgical resection alone)**"	To provide further clarity and detail to this exclusion criterion.
7.1.3. Discontinuation of Daratumumab SC	Text was revised as follows:<br>• **Participants who experience a Grade 4 sARR associated with administration of daratumumab SC.**<br>• Second event of Grade 3 sARR (including during re-starting of daratumumab SC administration; see Section 6.5.3.1) ~~or any event of Grade 4 sARR associated with administration of daratumumab SC.~~<br>• Any Grade ~~≥~~2 **or 3** laryngeal edema or Grade ≥2 **or 3** event of bronchospasm associated with administration of daratumumab SC that does not respond to systemic therapy and does not resolve within 6 hours from onset.	To provide clarity on when treatment with daratumumab SC should be discontinued.
7.1.4. Discontinuation of Lenalidomide	Text was revised as follows:<br>• **Anaphylactic reaction associated with lenalidomide**<br>• Grade 4 rash or **any grade of** exfoliative or bullous rash associated with lenalidomide. **Of note, discontinuation should be considered for Grade 2 or 3 skin rash.**<br>• Stevens-Johnson syndrome/toxic epidermal necrolysis, **drug reaction with eosinophilia and systemic symptoms (DRESS), or other severe dermatologic reaction** associated with lenalidomide.	To align with the SmPC of lenalidomide.
Table 2 and 8.2.2. Bone Marrow Examination	Cross-reference to Table 36 was added for details on bone marrow testing.	To provide clarity and consistency across the protocol.
8.3.7. Neurological Examination	Text was revised for consistency with Schedule of Activity Tables (Table 3, Table 4 and Table 5).	To provide clarity and consistency across the protocol.
10.2.14. Study and Site Start and Closure	Text was restructured for clarity and the following sentence was added:<br>"**A reason for the early closure of the study by the sponsor or investigator may include but is not limited to data revealing a major safety risk for participants**."	To provide a reason for early closure of the study.
Appendix 18: Clinical Laboratory Tests	Creatine kinase and thyroid-stimulating hormone were added to serum chemistry test to be performed. Table footnotes were revised.	To address concerns raised by the PEI.
Throughout the protocol	Minor grammatical, formatting, or spelling changes were made.	Minor errors were noted

11. REFERENCES

Adams HC III, Stevenaert F, Krejcik J, et al. High-parameter mass cytometry evaluation of relapsed/refractory multiple myeloma patients treated with daratumumab demonstrates immune modulation as a novel mechanism of action. Cytometry A. 2019;95(3):279-289.

Alonso R, Cedena MT, Wong S, et al. Prolonged lenalidomide maintenance therapy improves the depth of response in multiple myeloma. Blood Adv. 2020;4(10):2163–2171.

Ariza-Heredia EJ, Chemaly RF. Practical review of immunizations in adult patients with cancer. Hum Vaccin Immunother. 2015;11(11):2606-2614.

Attal M, Lauwers-Cances V, Hulin C, et al. Lenalidomide, bortezomib, and dexamethasone with transplantation for myeloma. N Engl J Med. 2017;376(14):1311-1320.

Bazarbachi AH, Al Hamed R, Malard F, Harousseau JL, Mohty M. Relapsed refractory multiple myeloma: a comprehensive overview. Leukemia. 2019;33(10):2343-2357.

Blade J, Esteve J, Rives S, et al. High-dose therapy autotransplantation/intensification vs continued standard chemotherapy in multiple myeloma in first remission. Results of a non-randomized study from a single institution. Bone Marrow Transplant. 2000;26(8):845-849.Burtis CA. Section IV Pathophysiology. In: Burtis CA, Ashwood ER, eds. Tietz textbook of clinical chemistry. 3rd ed. Philadelphia, PA: WB Saunders; 1999;1400. Available upon request.

CancerMPact® Kantar Health 2018. Western Europe patient metrics: sources and methodology. Health Economics website. www.cancermpact.com. Last updated September 2018. Accessed 30 January 2019.

Cavo M, Gay F, Patriarca F, et al. Double autologous stem cell transplantation significantly prolongs progression-free survival and overall survival in comparison with single autotransplantation in newly diagnosed multiple myeloma: an analysis of phase 3 EMN02/HO95 study. Blood. 2017;130(suppl 1):401.

Cavo M, Gay F, Beksac M, et al. Autologous haematopoietic stem-cell transplantation versus bortezomib-melphalan-prednisone, with or without bortezomib-lenalidomide-dexamethasone consolidation therapy, and lenalidomide maintenance for newly diagnosed multiple myeloma (EMN02/HO05): a multicentre, randomised, open-label, phase 3 study. Lancet Haematol. 2020;7(6):e456-e468.

Chapuy C, Nicholson R, Aguad MD, et al. Resolving the daratumumab interference with blood compatibility testing. Transfusion. 2015;55:1545-1554.

Chari A, Minnema, MC, Berdeja JG, et al. Talquetamab, a T-Cell–Redirecting GPRC5D Bispecific Antibody for Multiple Myeloma. N Engl J Med. 2022;387(24):2232-2244.

Child JA, Morgan GJ, Davies FE, et al. High-dose chemotherapy with hematopoietic stem-cell rescue for multiple myeloma. N Engl J Med. 2003;348(19):1875-1883.Chung C. Role of immunotherapy in targeting the bone marrow microenvironment in multiple myeloma: an evolving therapeutic strategy. Pharmacotherapy. 2017;37(1):129-143.

Cho SF, Lin L, Xing L, et al. The immunomodulatory drugs lenalidomide and pomalidomide enhance the potency of AMG 701 in multiple myeloma preclinical models. Blood Adv. 2020;4(17):4195-4207.

Cockcroft DW, Gault MH. Prediction of creatinine clearance from serum creatinine. Nephron. 1976;16(1):31-41.

Costa LJ, Chhabra S, Medvedova E, et al. Daratumumab, carfilzomib, lenalidomide, and dexamethasone with minimal residual disease response-adapted therapy in newly diagnosed multiple myeloma. J Clin Oncol. 2022;40(25):2901-2912.

Delforge M, Ludwig H. How I manage the toxicities of myeloma drugs. Blood. 2017;129(17):2359-2367.

Derman BA, Kansagra A, Zonder J, et al. Elotuzumab and weekly carfilzomib, lenalidomide, and dexamethasone in patients with newly diagnosed multiple myeloma without transplant intent: a phase 2 measurable residual disease–adapted study. JAMA Oncol. 2022;8(9):1278–1286.

Dimopoulos MA, Moreau P, Terpos E, et al. Multiple myeloma: EHA-ESMO Clinical Practice Guidelines for diagnosis, treatment and follow-up. Ann Oncol. 2021;32(3):309-322 (2021a).

Dimopoulos MA, Oriol A, Nahi H, et al. Daratumumab, lenalidomide, and dexamethasone for multiple myeloma. N Engl J Med. 2016;375(14):1319-1331.

Dimopoulos MA, Richardson PG, Schlag R, et al. VMP (bortezomib, melphalan, and prednisone) is active and well tolerated in newly diagnosed patients with multiple myeloma with moderately impaired renal function, and results in reversal of renal impairment: cohort analysis of the phase III VISTA study. J Clin Oncol. 2009;27(36):6086-6093.

Dimopoulos MA, San-Miguel J, Belch A, et al. Daratumumab plus lenalidomide and dexamethasone versus lenalidomide and dexamethasone in relapsed or refractory multiple myeloma: updated analysis of POLLUX. Haematologica. 2018;103(12):2088-2096.

Dimopoulos MA, Terpos E, Boccadoro M, et al. Daratumumab plus pomalidomide and dexamethasone versus pomalidomide and dexamethasone alone in previously treated multiple myeloma (APOLLO): an open-label, randomised, phase 3 trial. Lancet Oncol. 2021;22(6):801-812 (2021b).

Drayson MT, Bowcock S, Planche T, et al. Levofloxacin prophylaxis in patients with newly diagnosed myeloma (TEAMM): a multicentre, double-blind, placebo-controlled, randomised, phase 3 trial. Lancet Oncol. 2019;20(12):1760-1772.

Durie BGM, Miguel JFS, Blade J, Rajkumar SV. Clarification of the definition of complete response in multiple myeloma. Leukemia. 2015;29(12):2416-2417.

ECIS. European Cancer Information System. Data Explorer: Estimates of incidence and mortality in 2020, All Countries - Multiple Myeloma. Available from: https://ecis.jrc.ec.europa.eu. Accessed 28 September 2020.

CCI [redacted]

Facon T, Kumar S, Plesner T, et al. MAIA Trial Investigators. Daratumumab plus lenalidomide and dexamethasone for untreated myeloma. N Engl J Med. 2019;380(22):2104-2115.

Facon T, Kuman SK, Plesner T, et al. Daratumumab, lenalidomide, and dexamethasone versus lenalidomide and dexamethasone alone in newly diagnosed multiple myeloma (MAIA): overall survival results from a randomized, open-label, phase 3 trial. Lancet Oncol. 2021;22(11):1582-1596.

Farge D, Frere C, Connors JM, et al. 2022 international clinical practice guidelines for the treatment and prophylaxis of venous thromboembolism in patients with cancer, including patients with COVID-19. Lancet Oncol. 2022;(7):e334-e347.

Fernández de Larrea C, Staehr M, Lopez AV, et al. Defining an optimal dual-targeted CAR T-cell therapy approach simultaneously targeting BCMA and GPRC5D to prevent BCMA escape–driven relapse in multiple myeloma. Blood Cancer Discov. 2020;1(2):146 154.

Fernández de Larrea C, Kyle R, Rosiñol L, et al. Primary plasma cell leukemia: consensus definition by the International Myeloma Working Group according to peripheral blood plasma cell percentage. Blood Cancer J. 2021;11(12):192.

Fischbach FT, Dunning MB. *A Manual of Laboratory and Diagnostic Tests*. 7th ed. Philadelphia, PA:Williams & Wilkins; 2004.

Flockhart DA, Thacker D, McDonald C, Desta Z. The Flockhart Cytochrome P450 Drug-Drug Interaction Table. Division of Clinical Pharmacology, Indiana University School of Medicine (Updated 2021). Available from: https://drug-interactions.medicine.iu.edu/. Accessed 29 Dec 2021.

Fonseca R, Facon T, Hashim M, et al. First-line use of daratumumab, lenalidomide, and dexamethasone confers survival benefit compared with second-line use of daratumumab-based regimens in transplant-ineligible patients with multiple myeloma: Analysis of different clinical scenarios. ASH Annual Meeting & Exposition. 2021. Abstract 118. Available from: https://ash.confex.com/ash/2021/webprogram/Paper144914.html. Accessed 10 March 2022.

Fonseca R, Usmani SZ, Mehra M, et al. Frontline treatment patterns and attrition rates by subsequent lines of therapy in patients with newly diagnosed multiple myeloma. BMC Cancer. 2020;20(1):1087.

Frerichs KA, Broekmans MEC, Soto JAM, et al. Preclinical activity of JNJ-7957, a novel BCMAxCD3 bispecific antibody for the treatment of multiple myeloma, is potentiated by daratumumab. Clin Cancer Res. 2020;26(9):2203-2215.

Giralt S, Jolles S, Kerre T, et al. Recommendations for Management of Secondary Antibody Deficiency in Multiple Myeloma. Clin Lymphoma Myeloma Leuk. 2023;23(10):719-732.

Global RxPh The Clinician's Ultimate Reference: Corticosteroid – Glucocorticoid Conversion Based on Potency. Available from: https://globalrph.com/medcalcs/corticosteroid-converter-based-on-anti-inflammatory-potency/. September 2017. Accessed 16 September 2021.

Goldschmidt H, Mai EK, Bertsch U, et al. Addition of isatuximab to lenalidomide, bortezomib and dexamethasone as induction therapy for newly-diagnosed, transplant-eligible patients with multiple myeloma (GMMG-HD7): part 1 of an open-label, multicentre, randomised, active-controlled, phase 3 trial. Lancet Haematol. 2022;9(11):e810-e821.

Gormley N, Fashoyin-Aje L, Locke T, et al. Recommendations on eliminating racial disparities in multiple myeloma therapies: a step toward achieving equity in healthcare. Blood Cancer Discov. 2021;2(2):119-124.

Hamed RA, Bazarbachi AH, Malard F, Harousseau JL, Mohty M. Current status of autologous stem cell transplantation for multiple myeloma. Blood Cancer J. 2019;9(4):44.

Hari P, Pasquini MC, Stadtmauer EA, et al. Long-term follow-up of BMT CTN 0702 (STaMINA) of postautologous hematopoietic cell transplantation (autoHCT) strategies in the upfront treatment of multiple myeloma (MM). J Clin Oncol. 2020;38(15):8506.

Hayden PJ, Roddie C, Bader P, et al. Management of adults and children receiving CAR T-cell therapy: 2021 best practice recommendations of the European Society for Blood and Marrow Transplantation (EBMT) and the Joint Accreditation Committee of ISCT and EBMT (JACIE) and the European Hematology Association. Ann Oncol. 2022;33(3):259-275.Jones GL, Will A, Jackson GH, et al. Guidelines for the management of tumour lysis syndrome in adults and children with haematological malignancies on behalf of the British Committee for Standards in Haematology. Br J Haematol. 2015;169(5):661-671.

Klinger M, Brandl C, Zugmaier G, et al. Immunopharmacologic response of patients with B-lineage acute lymphoblastic leukemia to continuous infusion of T cell-engaging CD19/CD3-bispecific BiTE antibody blinatumomab. Blood. 2012;119(26):6226-6233.

Krishnan A, Hoering A, Hari P, Sexton R, Orlowski RZ. Phase III study of daratumumab/rhuph20 (nsc- 810307) + lenalidomide or lenalidomide as post-autologous stem cell transplant maintenance therapy in patients with multiple myeloma (MM) using minimal residual disease to direct therapy duration (DRAMMATIC study): SWOG s1803. Blood. 2020;136(suppl 1):21-22.

Kumar S, Paiva B, Anderson KC, et al. International Myeloma Working Group consensus criteria for response and minimal residual disease assessment in multiple myeloma. Lancet Oncol. 2016;17(8):e328-e346.

Lee DW, Santomasso BD, Locke FL, et al. ASTCT consensus grading for cytokine release syndrome and neurologic toxicity associated with immune effector cells. Biol Blood Marrow Transplant. 2019;25(4):625-638.

Lonial S, Lee HC, Badros A, et al. Belantamab mafodotin for relapsed or refractory multiple myeloma (DREAMM-2): a two-arm, randomised, open-label, phase 2 study. Lancet Oncol. 2020;21(2):207-221.

Lu G, Middleton RE, Sun H, et al. The myeloma drug lenalidomide promotes the cereblon-dependent destruction of Ikaros proteins. Science. 2014;343(6168):305-309.

Ludwig H, Terpos E, van de Donk N, et al. Prevention and management of adverse events during treatment with bispecific antibodies and CAR T cells in multiple myeloma: a consensus report of the European Myeloma Network. Lancet Oncol. 2023;24(6):e255-e269.

Ma C, Bandukwala S, Burman D, et al. Interconversion of three measures of performance status: an empirical analysis. Eur J Cancer. 2010;46(18):3175-3183.

Mateos MV, Nahi H, Legiec W, et al. Subcutaneous versus intravenous daratumumab in patients with relapsed or refractory multiple myeloma (COLUMBA): a multicentre, open-label, non-inferiority, randomised, phase 3 trial. Lancet Haematol. 2020;7(5):e370-e380.

Mazahreh F, Mazahreh L, Schinke C, et al. Risk of infections associated with the use of bispecific antibodies in multiple myeloma: a pooled analysis. Blood Adv. 2023:bloodadvances.2022009435.

McCarthy PL Holstein SA, Petrucci MT, et al. Lenalidomide maintenance after autologous stem-cell transplantation in newly diagnosed multiple myeloma: a meta-analysis. J Clin Onc. 2017;35(29):3279-3289.

McCudden C, Axel AE, Slaets D, et al. Monitoring multiple myeloma patients treated with daratumumab: teasing out monoclonal antibody interference. Clin Chem Lab Med. 2016;54(6):1095-1104.

Mohan M, Chakraborty R, Bal S, et al. Recommendations on prevention of infections during chimeric antigen receptor T-cell and bispecific antibody therapy in multiple myeloma. Br J Haematol. 2023;203(5):736-746.

Mohyuddin GR, Aziz M, McClune B, Abdallah AO, Qazilbash M. Antibiotic prophylaxis for patients with newly diagnosed multiple myeloma: Systematic review and meta-analysis. Eur J Haematol. 2020;104(5):420-426.

Minnema MC, Krishnan AY, Berdeja JG, et al. Efficacy and safety of talquetamab, a G protein-coupled receptor family C group 5 member D x CD3 bispecific antibody, in patients with relapsed/refractory multiple myeloma (RRMM): updated results from MonumenTAL-1. J Clin Oncol. 2022;40(no. 16_suppl):8015.

Moreau P, San Miguel JS, Ludwig H, et al. Multiple myeloma: ESMO Clinical Practice Guidelines for diagnosis, treatment and follow-up. Ann Oncol. 2013;24(suppl 6):vi133-vi137.

Moreau P, Attal M, Hulin C, et al. Bortezomib, thalidomide, and dexamethasone with or without daratumumab before and after autologous stem-cell transplantation for newly diagnosed multiple myeloma (CASSIOPEIA): a randomised, open-label, phase 3 study. Lancet. 2019;394(10192):29-38.

Moreau P, Sonneveld P. CASSIOPEIA Study Investigators. Daratumumab (DARA) maintenance or observation (OBS) after treatment with bortezomib, thalidomide and dexamethasone (VTd) with or without DARA and autologous stem cell transplant (ASCT) in patients (pts) with newly diagnosed multiple myeloma (NDMM): CASSIOPEIA Part 2. J Clin Oncol. 2021;39(15 suppl):8004.

Moreau P, Garfall AL, van de Donk NWCJ, et al. Teclistamab in Relapsed or Refractory Multiple Myeloma. N Engl J Med, 2022;387:495-505.

Mosteller RD. Simplified calculation of body-surface area. N Engl J Med, 1987;317:1098.

Munshi NC, Avet-Loiseau H, Anderson KC, et al. A large meta-analysis establishes the role of MRD negativity in long-term survival outcomes in patients with multiple myeloma. Blood Adv. 2020;4(23):5988-5999.

National Comprehensive Cancer Network. NCCN Clinical Practice Guidelines in Oncology (NCCN Guidelines®): Multiple Myeloma, version 4.2022 (14 December 2021). Available at: nccn.org. Accessed 28 December 2021.

Niesvizky R, Jayabalan DS, Christos PJ, et al. BiRD (Biaxin [clarithromycin]/Revlimid [lenalidomide]/dexamethasone) combination therapy results in high complete- and overall-response rates in treatment-naive symptomatic multiple myeloma. Blood. 2008;111(3):1101-1109.

Oken MM, Creech RH, Tormey DC, et al. Toxicity and response criteria of the Eastern Cooperative Oncology Group. Am J Clin Oncol. 1982;5(6):649-655.

Orlowski RZ. Why proteasome inhibitors cannot ERADicate multiple myeloma. Cancer Cell. 2013;24(3):275 277.

Pai MP. Estimating the glomerular filtration rate in obese adult patients for drug dosing. Adv Chronic Kidney Dis. 2010;17(5):e53-62.

Palumbo A, Cavallo F, Gay F, et al. Autologous transplantation and maintenance therapy in multiple myeloma. N Engl J Med. 2014;371(10):895-905.

Palumbo A, Rajkumar SV, Dimopoulos MA, et al. Prevention of thalidomide- and lenalidomide-associated thrombosis in myeloma. Leukemia. 2008;22(2):414-423.

Pellom ST Jr, Dudimah DF, Thounaojam MC, et al. Bortezomib augments lymphocyte stimulatory cytokine signaling in the tumor microenvironment to sustain CD8+T cell antitumor function. Oncotarget. 2017;8(5):8604-8621.

Quach H, Ritchie D, Stewart AK, et al. Mechanism of action of immunomodulatory drugs (IMiDS) in multiple myeloma. Leukemia. 2010;24(1):22-32.

Raje NS, Anaissie E, Kumar SK, et al. Consensus guidelines and recommendations for infection prevention in multiple myeloma: a report from the International Myeloma Working Group. Lancet Haematol. 2022;9(2):e143-e161.

Rajkumar SV, Harousseau JL, Durie B, et al. Consensus recommendations for the uniform reporting of clinical trials: report of the International Myeloma Workshop Consensus Panel 1. Blood. 2011;117(18):4691-4695.

CCI [redacted]

Rosinol L, Beksac M, Zamagni E, et al. Expert review on soft-tissue plasmacytomas in multiple myeloma: definition, disease assessment and treatment considerations. Br J Hematol. 2021;194(3):496-507.

Rossi A, Mark T, Jayabalan D, et al. BiRd (clarithromycin, lenalidomide, dexamethasone): an update on long-term lenalidomide therapy in previously untreated patients with multiple myeloma. Blood. 2013;121(11):1982-1985. doi:10.1182/blood-2012-08-448563

SEER 2020. Surveillance, Epidemiology, and End Results Program (www.seer.cancer.gov). SEER*Stat Database: Incidence - SEER Research Data, 9 Registries, Nov 2019 Sub (1975-2017) - Linked To County Attributes - Time Dependent (1990-2017) Income/Rurality, 1969-2017 Counties, National Cancer Institute, DCCPS, Surveillance Research Program, released April 2020, based on the November 2019 submission.

Shah ND, Lutska Y, Pei H, et al. Daratumumab (DARA) plus lenalidomide versus lenalidomide alone as maintenance treatment in patients with newly diagnosed multiple myeloma (NDMM) after frontline autologous stem cell transplant (ASCT): use of minimal residual disease (MRD) as a novel primary endpoint in the phase 3 AURIGA study.Blood. 2019;134(suppl 1):1829.

Sidana S, Narkhede M, Elson P, et al. Neuropathy and efficacy of once weekly subcutaneous bortezomib in multiple myeloma and light chain (AL) amyloidosis. PLoS One. 2017;12(3):e0172996.

Sonneveld P, Goldschmidt H, Rosinol L, et al. Bortezomib-based versus nonbortezomib-based induction treatment before autologous stem-cell transplantation in patients with previously untreated multiple myeloma: A meta-analysis of Phase III randomized, controlled trials. J Clin Oncol. 2013;31(26):3279-3287.

Sonneveld P, Dimopoulos MA, Boccadoro M, et al. Daratumumab, Bortezomib, Lenalidomide, and Dexamethasone for Multiple Myeloma. N Engl J Med. Published online December 12, 2023. Tai YT, Anderson KC. Targeting B-cell maturation antigen in multiple myeloma. Immunotherapy. 2015;7(11):1187-1199.

Terpos E, Kleber M, Engelhardt M, et al. European Myeloma Network guidelines for the management of multiple myeloma-related complications. Haematologica. 2015;100(10):1254 1266.

Thounaojam MC, Dudimah DF, Pellom ST Jr, et al. Bortezomib enhances expression of effector molecules in anti-tumor CD8+ T lymphocytes by promoting Notch-nuclear factor-κB crosstalk. Oncotarget. 2015;6(32):32439-32455.

Turesson I, Bjorkholm M, Blimark CH, Kristinsson S, Velez R, Landgren O. Rapidly changing myeloma epidemiology in the general population: Increased incidence, older patients, and longer survival. Eur J Haematol. 2018;101(2):237-244.

US Department of Health and Human Services. Food and Drug Administration. FDA Guidance for Industry: Pharmacokinetics in Patients with Impaired Renal Function – Study Design, Data Analysis, and Impact on Dosing. https://www.fda.gov/media/78573/download. September 2020. Accessed 12 December 2021.

Usmani S, Ahmadi T, Ng Y, et al. Analysis of real-world data on overall survival in multiple myeloma patients with ≥3 prior lines of therapy including a proteasome inhibitor (PI) and an immunomodulatory drug (IMiD), or double refractory to a PI and an IMiD. Oncologist. 2016;21(11):1355-1361.

Usmani SZ, Garfall AL, van de Donk CJ, et al. Teclistamab, a B-cell maturation antigen xCD3 bispecific antibody, in patients with relapsed or refractory multiple myeloma (MajesTEC-1): a multicentre, open-label, single-arm, phase 1 study. Lancet. 2021;398(10301):665-674.

Usmani SZ, Hoering A, Cavo M, et al. Clinical predictors of long-term survival in newly diagnosed transplant eligible multiple myeloma-an IMWG Research Project. Blood Cancer J. 2018;8:123.

Venner CP, Bahlis NJ, Neri P, et al. In multiple myeloma progression free and overall survival in the relapsed setting remains poor with early exposure to novel agents: experience from a real-world cohort. Blood. 2015;126(23):4261.

Verkleij CPM, Broekmans MEC, van Duin M, et al. Preclinical activity and determinants of response of the GPRC5DxCD3 bispecific antibody talquetamab in multiple myeloma. Blood Adv. 2021;5(8):2196-2215.

Voorhees PM, Kaufman JL, Laubach J, et al. Daratumumab, lenalidomide, bortezomib, and dexamethasone for transplant-eligible newly diagnosed multiple myeloma: the GRIFFIN trial. Blood. 2020;136(8):936-945.

Wang X, Walter M, Urak R, et al. Lenalidomide enhances the function of CS1 chimeric antigen receptor-redirected T cells against multiple myeloma. Clin Cancer Res. 2018;24(1):106-119.

CCI [redacted]

[redacted]

Works M, Soni N, Hauskins C, et al. Anti-B cell maturation antigen chimeric antigen receptor t cell function against multiple myeloma is enhanced in the presence of lenalidomide. Mol Cancer Ther. 2019;18(12):2246-2257.

Yong K, Delforge M, Driessen C, et al. Multiple myeloma: patient outcomes in real-world practice. Br J Haematol. 2016;175(2):252-264.

INVESTIGATOR AGREEMENT

JNJ-64007957; JNJ-64407564 (teclistamab; talquetamab)

Clinical Protocol GMMG-HD10/DSMM XX/64007957MMY2003 Amendment 5

INVESTIGATOR AGREEMENT

I have read this protocol and agree that it contains all necessary details for carrying out this study. I will conduct the study as outlined herein and will complete the study within the time designated.

I will provide copies of the protocol and all pertinent information to all individuals responsible to me who assist in the conduct of this study. I will discuss this material with them to ensure that they are fully informed regarding the study intervention, the conduct of the study, and the obligations of confidentiality.

Coordinating Investigator (where required):

Name (typed or printed): ______________________

Institution and Address: ______________________

____

____

____

Signature: ______________________ Date: ______________________

(Day Month Year)

Principal (Site) Investigator:

Name (typed or printed): ______________________

Institution and Address: ______________________

____

____

____

Telephone Number: ______________________

Signature: ______________________ Date: ______________________

(Day Month Year)

Sponsor's Responsible Medical Officer:

Name (type PPD [redacted]

Institution: [redacted]

Signature: [redacted] Date: PPD [redacted]

(Day Month Year)

Note: If the address or telephone number of the investigator changes during the study, written notification will be provided by the investigator to the sponsor, and a protocol amendment will not be required.

CONFIDENTIAL – FOIA Exemptions Apply in U.S. 265

Status: Approved, Date: 19 December 2024

Statistical Analysis Plan (SAP)

Version 2.0

JNJ-64007957 (teclistamab); JNJ-64407564(talquetamab)

Study GMMG-HD10/DSMM XX/64007957MMY2003

A Phase 2 Study to Evaluate Safety and Efficacy of Teclistamab- and Talquetamab-based Combination Regimens in Participants with Newly Diagnosed Transplant Eligible Multiple Myeloma

Revision History

Version	Date	Author(s)	Summary of Changes/Comments
1.0	04-20-2023	PPD	First version based on protocol amendment 2
2.0	03-28-2024	PPD	Second version based on protocol amendment 4 issued on 13/03/2024

TABLE OF CONTENTS

TABLE OF CONTENTS 3

LIST OF ABBREVIATIONS 5

1. Introduction 7

1.1. Amendments from Previous Version(s) 7
1.2. Study Objectives 8
1.3. Study Design 8
1.4. Study Population 11
1.5. Study Period and Visit Window Definitions 11
1.6. Hypotheses and Decision Rules 12
1.7. Sample Size Justification 12
1.8. Randomization and Blinding 13
1.9. Deviations from Protocol 13

2. Populations of Analysis 13

2.1. Safety Analysis Population 13
2.2. Efficacy Analysis Populations 14
2.3. Per Protocol Population 15
2.4. Other Populations 15
2.5. Subgroup Definitions 16
2.6. Protocol Deviations 16

3. Study Endpoints 16

3.1. Efficacy Endpoints 16
3.2. Safety Endpoints 17
3.3. Other Endpoints 19

4. Statistical AnalySes 21

4.1. Timing of the primary analyses 21
4.2. Study Participants and Data Sets Analyzed 21
4.3. Demographic and Other Baseline Characteristics 22
4.4. Evaluation of Treatment Compliance and Exposure 23
4.5. Prior and Concomitant Medication 24
4.6. Analysis of Efficacy Endpoints 24
4.7. Analysis of Safety Endpoints 26
4.8. Analyses of Other Endpoints 28
4.9. Interim Analyses and Safety Monitoring Analyses 29

REFERENCES 31

APPENDIX 1 Further Definition of Endpoints 32
APPENDIX 2 Immune-mediated/autoimmune disorders 34

LIST OF ABBREVIATIONS

ADAs	Antidrug antibodies
AE	Adverse event
ALT	Alanine aminotransferase
ANC	Absolute neutrophil count
ASCT	Autologous stem cell transplantation
ATC	Anatomical therapeutic chemical
BCMA	B cell maturation antigen
BSA	Body surface area
C1D1	Cycle 1 Day 1
CAR-T	Chimeric antigen receptor T cells
CI	Confidence Intervals
CR	Complete response
CRF	Case report form
CRS	Cytokine Release Syndrome
CSR	Clinical study report
DOR	Duration of response
ECG	Electrocardiogram
ECOG	Eastern Cooperative Oncology Group
eGFR	Estimated glomerular filtration rate
EOT	End of treatment
FAS	Full Analysis Set
FLC	Serum free light chain
HDT	High-dose (chemo-)therapy
HRQoL	Health-related quality of life
ICANS	Immune effector cell-associated neurotoxicity syndrome
ICH	International Conference on Harmonisation
IEC	Independent ethics committee
IMWG	International Myeloma Working Group
IRB	Institutional review board
ISS	International Staging System
ITT	Intent-To-Treat
MedDRA	Medical Dictionary for Regulatory Activities
MI	Multiple Imputation
MRD	Minimal residual disease
NCI-CTCAE	National Cancer Institute Common Terminology Criteria for Adverse Events

ND-TEMM	Newly Diagnosed Transplant Eligible Multiple Myeloma
NGF	Next-generation flow
ORR	Overall response ratio
OS	Overall survival
PA	Protocol amendment
PD	Progressive disease
PFS	Progression-free survival
PK	Pharmacokinetic
PP	Per Protocol
PR	Partial response
PRO	Patient-reported outcome
PT	Preferred term
SAE	Serious adverse event
SAP	Statistical Analysis Plan
SD	Standard deviation
SE	Standard error
SC	Subcutaneous
SoC	Standard of Care
SOC	System organ class
SST	Subsequent therapy
Tal	Talquetamab
CCI [redacted]	[redacted]
TEAE	Treatment-emergent adverse events
Tec	Teclistamab
Tec-D	Teclistamab in combination with Daratumumab (D)
Tec-DR	Teclistamab in combination with Daratumumab (D) and Lenalidomide (R)
Tec-DRd	Teclistamab in combination with Daratumumab (D), Lenalidomide (R) and Dexamethasone (d)
Tec-DVRd	Teclistamab in combination with Daratumumab (D), Bortezomib (V), Lenalidomide (R) and Dexamethasone (d)
VGPR	Very good partial response
WHO-DD	World health organization drug dictionnary

1. INTRODUCTION

This statistical analysis plan (SAP) contains definitions of the analysis sets, derived variables and statistical methods for the planned analysis for the clinical study report (CSR) of the Phase 2, open label, Study to Evaluate Safety and Efficacy of Teclistamab- and Talquetamab-based Combination Regimens in Participants with Newly Diagnosed Transplant Eligible Multiple Myeloma (ND-TEMM).

1.1. Amendments from Previous Version(s)

This is the first amendment of the SAP and is based on protocol amendment 4.

The purpose of amendment 1 of the SAP is to update the SAP with protocol amendment 3 (PA3; 06 July 2023) and protocol amendment 4 (PA4; 13 March 2024) of the protocol. The major changes are as follows:

- Incorporate the new treatment arms added in the protocol (PA3: A1, C1; PA4: CCI [redacted]
- Align maintenance regimen for arms A, B, and C with the protocol (PA4);
- Align the wording of the primary objective and the hypothesis with the protocol;
- Align the wording of the timing of the primary analyses with the protocol;
- Add pooling of the treatment arms for safety and efficacy analyses;
- Add the analyses of the patient-related outcome (PRO) questionnaires (added by PA4).

**Applicable Section(s)**	**Description of Change(s)**
Rationale: Updates made according to latest protocol amendments 4 and 3 and analyses added accordingly	
Title page, Introduction	Update study title
1.2	Update study objectives and clinical hypotheses as described in the protocol
1.3	Update study design as described in the protocol
1.4	Update study population as described in the protocol
1.5	Add study period and visit window as described in the protocol
1.7	Update sample size justification as described in the protocol
1.8	Add treatment allocation as described in the protocol
2.1, 2.2, 2.4	Update populations of analysis and adding pooling of the treatment arms for safety and efficacy analyses
3.1, 3.2, 3.3	Update study endpoints and adding patient-related outcome as endpoints
4.1	Adding the timing of the primary analyses
4.6, 4.7, 4.8	Update study endpoints analyses and adding the analyses of the patient-related outcome
4.9	Update the timing of interim analyses and safety monitoring analyses
References	Adding some references to the references section
Rationale: Minor errors were noted	
Throughout the document	Minor grammatical, formatting, or spelling changes were made

1.2. Study Objectives

The primary objective is to evaluate the safety and tolerability of teclistamab- and talquetamab-based combination regimens over the entire treatment phase for each arm, in participants with ND-TEMM.

The clinical hypothesis of this study is that teclistamab- and talquetamab-based combination regimens are safe and well tolerated treatments in participants with ND-TEMM.

1.3. Study Design

This is an open-label multicenter study in participants with ND-TEMM.

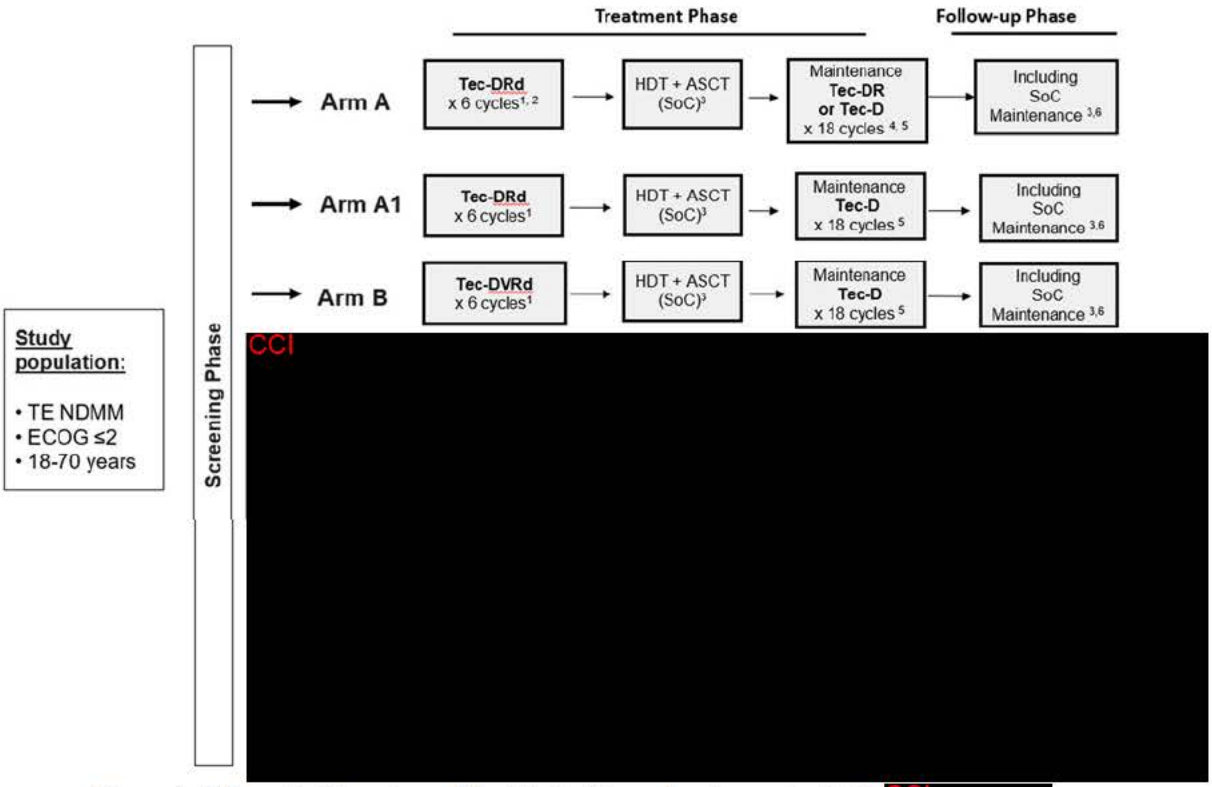

Figure 1 Schematic Overview of the Study Phases for Arms A, A1, B, [redacted]

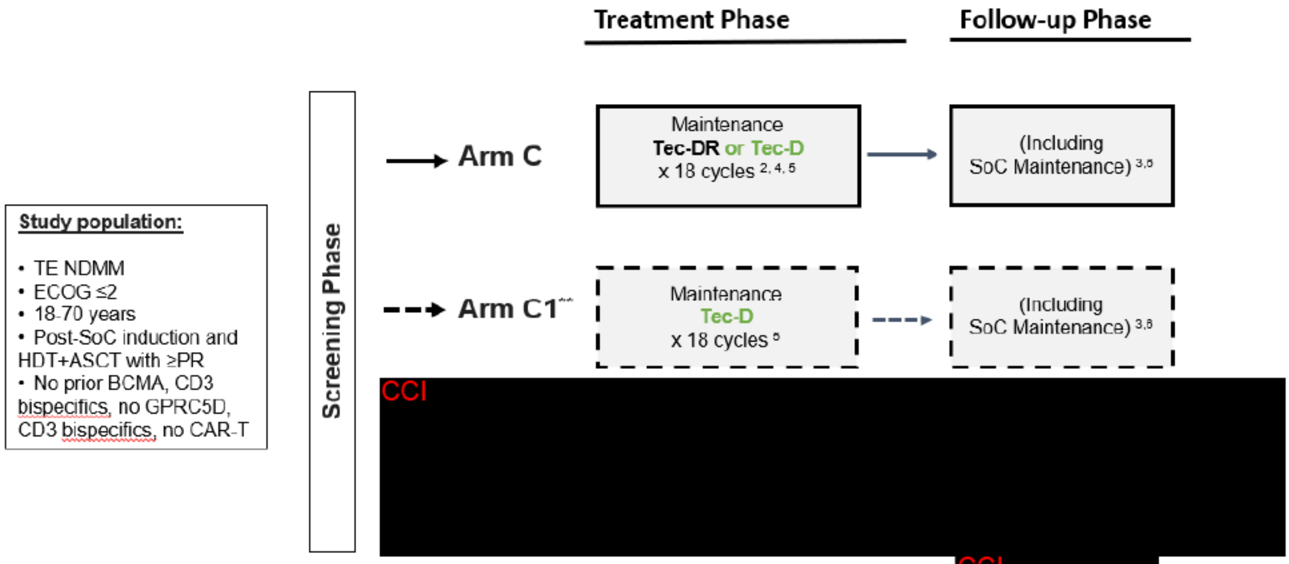

Figure 2 Schematic Overview of the Study Phases for Arms C, C1, [redacted]

1. Including stem cell collection.
2. Arm A and Arm C participants initially treated with previous teclistamab dosing schedules have transitioned to teclistamab monthly dosing.
3. SoC treatments are not considered study treatments.
4. Per Amendment 4, participants in Arm A and Arm C initially assigned to receive Tec-DR maintenance may receive Tec-D maintenance per investigator's choice (participants who have started Tec-DR may discontinue lenalidomide to receive Tec-D per investigator's choice).
5. [redacted] therapy following induction, or study maintenance therapy with Tec-DR, Tec-D, [redacted] or [redacted], to be administered for a maximum of 18 cycles.
6. Disease evaluation before progressive disease every 12 weeks (±1 week) until confirmed PD or start of SST (whichever occurs earlier).

* Based on emerging data from Arms A1, B, [redacted], the sponsor may open Arms [redacted]

** Based on evolving data, the sponsor may also open Arm C1 where participants receive Tec-DR maintenance treatment with teclistamab administered monthly as of Cycle 1.

The study will be conducted in 3 phases: Screening (28 days), Treatment, and Follow-up. For Arms A, A1, B, [redacted] the Treatment Phase includes induction treatment and maintenance treatment. [redacted] For Arms C, C1, [redacted] the Treatment Phase includes maintenance treatment (Figure 2).

During the Screening Phase, all participants will provide written consent for study participation and will be screened for study eligibility within 28 days. All eligibility criteria must be met prior to enrollment and eligibility should be confirmed at the time of first dose of study treatment. The sponsor will strive to enroll a participant population that adequately represents the multiple myeloma population generally (Gormley 2021).

Given the limited data examining teclistamab or talquetamab in combination with other medications in participants with NDMM eligible for ASCT, the study will include early safety evaluations. To facilitate these evaluations, a Study Safety Committee which includes external experts, and a Study Steering Committee will be commissioned for the study.

Treatment Phase

Arms A, A1, B, [redacted]

After Screening, the Treatment Phase begins with the administration of study treatment (induction with Tec-DRd [Arms A, A1, [redacted] Tec-DVRd [Arm B], or [redacted] [Arms [redacted] and continues until the completion of the EOT Visit. Based on emerging data from Arms A1, B, [redacted], the sponsor may also open Arms [redacted] where participants receive [redacted] induction. Periodic safety evaluations will be conducted to ensure that treatment is safe and tolerable.

Participants will receive six 28-day cycles of Tec-DRd, Tec-DVRd, [redacted] or [redacted] induction therapy followed by HDT and a single ASCT according to local Standard of Care (SoC) (Arms A, A1, B, [redacted] and [redacted] or [redacted] treatment for a maximum of 18 cycles or until confirmed progressive disease, death, intolerable toxicity, loss to follow-up, or consent withdrawal, whichever comes first (Arm [redacted] Stem cell collection is recommended to be done after Induction Cycle 3 according to local SoC.

After ASCT, per PA4, participants in Arm A initially assigned to receive Tec-DR maintenance treatment may receive Tec-D maintenance per investigator's choice. Participants in Arms A1, B, [redacted] will receive study maintenance treatment with Tec-D, and participants in Arms [redacted] will receive study maintenance treatment with [redacted]. Study maintenance treatment will be for a maximum of 18 cycles or until confirmed progressive disease, death, intolerable toxicity, loss to follow-up, or consent withdrawal, whichever comes first.

[redacted] treatment should be started within 28 days of induction and at least 14 days after the last dose of lenalidomide. Maintenance therapy should commence within 180 days of transplant when engraftment is complete (defined as ANC ≥1.0 x 10^9/L and platelet count ≥75 x 10^9/L) and when in the opinion of the investigator the participant is fit enough to tolerate maintenance therapy.

Arms C, C1, [redacted]

Participants enter the Screening Phase after induction, HDT, and ASCT according to local SoC (outside of the study). The Treatment Phase begins with the administration of study treatment (study maintenance treatment with Tec-DR or Tec-D per investigator's choice in Arm C, or [redacted] in Arm [redacted] and continues until the completion of the EOT Visit. Based on evolving data, the sponsor may also open Arm C1 where participants receive Tec-D maintenance treatment with teclistamab administered monthly as of Cycle 1. Participants will receive study maintenance treatment with Tec-DR, Tec-D, or [redacted] for a maximum of 18 cycles or until confirmed progressive disease, death, intolerable toxicity, loss to follow-up or consent withdrawal, whichever comes first. Periodic safety evaluations will be conducted to ensure that treatment is safe and tolerable.

<u>Follow up Phase</u>

All Arms

Upon treatment discontinuation, an EOT Visit will be conducted. Thereafter, participants will continue in the Follow up Phase until death, withdrawal of consent, loss to follow-up, or end of the study, whichever occurs first.

Following study maintenance therapy, additional SoC maintenance treatment per institutional standard and local investigator decision, is permitted. This additional maintenance therapy, without intercurrent progressive disease, is not considered subsequent therapy and is outside of the study. If decided to be in the best interest of the participant by the local investigator, the participant can also be observed only after the end of study treatment.

In the Follow-up Phase, participants who discontinued treatment before disease progression must continue to have disease evaluations and should not initiate any subsequent antimyeloma treatment until confirmed disease progression. After disease progression is documented, follow up will continue and subsequent antimyeloma treatment, disease progression data (per investigator assessment) on the first line of subsequent therapy, second primary malignancies, and survival status will also be recorded.

Participants who have a response of Complete Response (CR) or better can stop Tec-DR, Tec-D, [redacted], or [redacted] maintenance after sustained MRD negativity assessed by central labs (at or below the threshold of 10^{-5}) for 12 months, after sponsor approval. Initial MRD negativity may occur when in minimum response of Very Good Partial Response (VGPR), however the criteria of CR or better must be met at the time of decision to stop maintenance Tec-DR, Tec-D, [redacted] or [redacted] therapy. Qualified participants can subsequently continue standard of care maintenance treatment per institutional standard and local investigator decision.

In addition to efficacy and safety assessments, samples for pharmacokinetic (PK) and immunogenicity and samples for pharmacodynamics and exploratory biomarkers will be collected. All study evaluations will be conducted according to the Schedule of Activities. Arms [redacted] and C1 are optional per protocol. If one of these arms does not open, the analysis related to that specific arm will not be carried out; and the pooling (Section 2) will simply proceed without that specific arm.

1.4. Study Population

The study population includes participants with ND-TEMM, per study inclusion/exclusion criteria. More specifically,

- Arms A, A1, B, [redacted] include newly diagnosed participants for whom HDT and ASCT is part of the intended treatment plan;
- Arm C, C1, [redacted] include newly diagnosed participants who received 4 to 6 cycles of induction therapy and HDT and ASCT.

1.5. Study Period and Visit Window Definitions

See details in study design section 1.3 and schematic overview of the study phases.

Unless otherwise specified, safety will be summarized overall (and for selected analyses, by phase). For the definition of Treatment-Emergent Adverse Events (TEAEs) TEAE's (for overall summaries), see Section 3.2; for the definition of study phases for reporting TEAEs, see Section 4.7. The same definition will be used for reporting deaths and laboratory values.

Unless otherwise specified, data to be analyzed or listed over time will be presented by day and time point (as appropriate) that are recorded in the eCRF.

For analyses of data by cycle, if data are collected by date (e.g., AE onset), the corresponding study evaluations will be assigned to actual sequential cycles, which are derived from the study treatment administration data. The start date of a particular cycle is defined as the date of the first scheduled dose of any component of the study treatment, and the end date of a cycle is the start date of the next cycle minus 1. For the last cycle, the end date is defined as the end of treatment visit date or the minimum of last study treatment date plus 30 days and subsequent antimyeloma therapy minus 1 day if the end of treatment visit date is not available.

In general, if data (e.g., laboratory and vital sign etc.) are collected by cycle, the nominal cycle will be used to summarize data. However, due to possible cycle delays, assessment performed in the same cycle may not be well aligned in time scale for different participants. To address this, by week windowing rules may be applied in the overtime data summaries by study week.

Study Day 1 refers to the start of the first study treatment administration. All efficacy and safety assessments at all visits will be assigned a day relative to this date.
Study day or relative day for a visit is defined as:

- Visit date - (date of Study Day 1) +1, if visit date is ≥ date of Study Day 1
- Visit date - date of Day 1, if visit date < date of Day 1

There is no 'Day 0'.

1.6. Hypotheses and Decision Rules

No statistical hypothesis testing will be performed in this study.

1.7. Sample Size Justification

This study anticipates enrolling up to a maximum of approximately 320 participants. Arm A will enroll approximately 10 participants. Arms A1 and B will initially enroll approximately 20 participants each and may be expanded up to a total of 80 participants combined (Arms A1 and B combined). Arm C will enroll approximately 10 participants, and based on evolving data Arm C1 may also be opened to enroll approximately 10 participants. Arm [redacted] will initially enroll approximately 10 participants and may enroll up to 30 participants. Arm [redacted] will initially enroll approximately 20 participants and may enroll up to 60 participants. Arms [redacted] will enroll up to 30 participants each. Based on emerging data from Arms A1 and B, Arms [redacted] may also be opened to enroll up to 30 participants each.

Table 1 presents the length of the widest 95% confidence interval, given the possible sample sizes per arm, for an AE rate of 50% based on Clopper-Pearson 95% confidence interval.

Table 1: Length of Widest 95% Confidence Interval Around AE Rates

Sample Size in Treatment Arm [a]	Length of Widest 95% CI [b]
10	62.6%
20	45.6%
30	37.4%
40	32.4%
50	28.9%
60	26.4%

a. Arm A1 and Arm B will initially enroll approximately 20 participants per cohort. Based on the safety snapshot and the totality of data, Arm A1 *and/or* Arm B may be expanded up to a total of 80 participants (Arms A1 and B combined); i.e., a given treatment arm may have up to a maximum of 60 participants. Arms A, C and (if opened) C1 will have approximately 10 participants each. Arm [redacted] may have up to 30 participants. Arm [redacted] may have up to 60 participants. Arms [redacted] and [redacted] and (if opened) [redacted] and [redacted] will have up to 30 participants each.
b. Based on Clopper and Pearson 95% confidence interval, assuming an AE rate of 50%.

1.8. Randomization and Blinding

Randomization will not be used in this study.

Treatment Allocation

Participants will be assigned to either Arm A1 or Arm B in parallel based on a schedule prepared before the study, by or under the supervision of the sponsor.

Participants will be assigned to Arm [redacted] or Arm [redacted] in parallel, and to Arm [redacted] or Arm [redacted] in parallel based on a schedule prepared before the study, by or under the supervision of the sponsor.

(If it is decided that other combinations of arms are to be assigned in parallel, for example due to the flexibility on opening or expanding an arm, then allocation will be based on a schedule prepared before the study, by or under the supervision of the sponsor.)

Blinding

As this is an open study, blinding procedures are not applicable.

1.9. Deviations from Protocol

In general, the following list of major protocol deviations may have the potential to impact participants' rights, safety or well-being, or the integrity and/or result of the clinical study. Participants with major protocol deviations will be identified prior to database lock.

- Developed withdrawal criteria but not withdrawn
- Entered but did not satisfy criteria
- Received a disallowed concomitant treatment
- Received wrong treatment or incorrect dose
- Other

2. POPULATIONS OF ANALYSIS

2.1. Safety Analysis Population

All safety analyses will be performed on the all-treated population, which consists of participants who received at least one dose of study treatment. Safety will be summarized by treatment arms (including primary analysis).
In Arm A, up until PA4, participants were assigned to receive Tec-DR maintenance treatment (where lenalidomide is given as of Cycle 2). As described in Section 1.3, per PA4, participants in Arm A initially assigned to receive Tec-DR maintenance treatment may receive Tec-D maintenance per investigator's choice; participants who have not yet received their first Tec-

DR dose by the time of PA4 approval, may receive only Tec-D maintenance. Thus, within Arm A, for the purposes of the maintenance safety analyses, there are 2 safety populations:

- **Tec-DR Arm A patients:** Participants who received at least one Tec-DR dose as maintenance or they discontinued study treatment before the second maintenance dose (this will include all participants who are on maintenance treatment before PA4);
- **Tec-D Arm A patients:** Participants who never received Tec-DR as maintenance and they received at least 2 Tec-D maintenance doses (this will include all participants who are on maintenance schedule specified in PA4; note that, dependent on the actual dosing schedules and the time of the PA4 approval, this group may not have any participants).

Similarly, per PA4, participants in Arm C initially assigned to receive Tec-DR maintenance treatment may receive Tec-D maintenance per investigator's choice. However, at the time of PA4 approval all participants in Arm C will have received their first Tec-DR dose and so equivalent maintenance safety analyses will not be defined for Arm C.

Selected safety analyses (TEAE, SAE; see Section 4.7 for more details) will be conducted on pooled arms, as specified in Table 2 (Modifications are possible, e.g., due to data availability, as arms open for enrolment at different times; additional combinations may also be added for exploratory purpose).

Table 2 Combining Arms for Additional Pooled Safety Analyses, by Treatment Phase

Treatment Phase	Regimen	Combination	Arms
Induction	teclistamab-based regimens	Tec-DRd	A + A1 [redacted]
	[redacted]	[redacted]	[redacted]
Maintenance	teclistamab-based regimens	Tec-DR	A (Tec-DR patients) + C
		Tec-D	A (Tec-D patients) + A1 + B +C1 + [redacted]
	[redacted]	[redacted]	[redacted]

+ sign stands for combining arms

2.2. Efficacy Analysis Populations

There will be two analysis populations: efficacy analysis population and response-evaluable population. The primary analysis of all efficacy endpoints will be based on the efficacy analysis population. The International Myeloma Working Group (IMWG) response rates will also be performed using the response-evaluable population.

The efficacy analysis population consists of enrolled participants who received at least one dose of the study treatment.

The response-evaluable population includes all enrolled participants who received at least one disease evaluation and have measurable disease at baseline (or at diagnostic baseline for Arm C, C1, [redacted],or who do not have measurable disease at baseline or screening but are assessed as CR or better.

A measurable disease at baseline is defined as follows:

- Serum monoclonal paraprotein (M-protein) level ≥1.0 g/dL or urine M-protein level ≥200 mg/24 hours; or
- Serum immunoglobulin free light chain (FLC) ≥10 mg/dL and abnormal serum immunoglobulin kappa lambda FLC ratio.

Efficacy will be summarized by treatment arms. In addition, selected efficacy analyses will be conducted on pooled arms, as specified in Table 3 (Modifications are possible, e.g., due to data availability, as arms open for enrolment at different times; additional combinations may also be added for exploratory purpose).

Table 3 Combining Arms for Additional Efficacy Analyses by Treatment Phase and Overall

Endpoint and Treatment Phase	Regimen	Combination	Arms
Endpoints based on MRD and response, at post-induction	teclistamab-based regimens	Tec-DRd	A + A1 [redacted]
		Tec-DRd or Tec-DVRd	A + A1 + B [redacted]
	[redacted]	[redacted]	[redacted]
Endpoints based on MRD and response, at post-transplant	teclistamab-based regimens	Tec-DRd	A + A1
		Tec-DRd or Tec-DVRd	A + A1 + B
	[redacted]	[redacted]	[redacted]

+ sign stand for combining arms.

2.3. Per Protocol Population

NA.

2.4. Other Populations

Teclistamab PK-evaluable set: All participants who received at least one dose of teclistamab and have at least one evaluable PK sample after the first dose of treatment.

Talquetamab PK-evaluable set: All participants who received at least one dose of talquetamab and have at least one evaluable PK sample after the first dose of treatment.

Daratumumab PK-evaluable set: All participants who received at least one dose of Daratumumab and have at least one evaluable PK sample after the first dose of treatment.

Teclistamab immunogenicity-evaluable set: All participants who received at least one of teclistamab and have at least one immunogenicity sample after the first dose of treatment.

Talquetamab immunogenicity-evaluable set: All participants who received at least one of Talquetamab and have at least one immunogenicity sample after the first dose of treatment.

Daratumumab immunogenicity-evaluable set: All participants who received at least one of Daratumumab and have at least one immunogenicity sample after the first dose of treatment.

2.5. Subgroup Definitions

Subgroup analyses may be performed as needed.

2.6. Protocol Deviations

No protocol deviations that will lead to exclusion from the safety analysis.

3. STUDY ENDPOINTS

The primary endpoints are incidence and severity of Adverse Events (AEs) and Serious Adverse Events (SAEs) (see details in Section 3.2). The secondary endpoints include the efficacy endpoints and endpoints for stem cell harvest.

3.1. Efficacy Endpoints

Efficacy endpoints include:

Minimal residual disease (MRD) negative complete response (CR), defined as achieving MRD negative status as determined by next-generation flow (NGF) with a sensitivity of 10^{-5}, and achieve CR or better response at any time after the date of first dose of the study medication and prior to progressive disease, subsequent therapy, or both.

Sustained MRD negative CR, achieving MRD negative CR, confirmed for a minimum of 12 months apart and without any examinations in between showing MRD-positive status, occurrence of PD, or start of subsequent anti-myeloma therapy.

MRD negative CR conversion during maintenance (all arms except Arm [redacted] or during [redacted] treatment (Arm [redacted] defined as participants who had a response worse than CR or were assessed as MRD positive (10^{-5} by NGF) prior to the first dose of the maintenance treatment (all arms except Arm [redacted] or prior to first dose of [redacted] treatment (Arm [redacted] and who later achieved MRD negative CR status.

MRD negative deepening during maintenance (all arms except Arm [redacted] or during [redacted] treatment (Arm [redacted] defined as the change in the proportion of participants with MRD-negativity prior to the first dose of the maintenance treatment (all arms except Arm [redacted] or prior to first dose of [redacted] treatment (Arm [redacted] to the end of the study.

MRD negativity defined regardless of response per IMWG, as achieving MRD negative status as determined by next-generation flow (NGF) with a sensitivity of 10^{-5}, after the date of first dose of the study medication and prior to progressive disease, subsequent therapy, or both.

Response rates (Arms A, A1, B, [redacted]):

Overall Response Rate (ORR) defined as the proportion of participants who achieve Partial Response (PR) or better, according to the IMWG criteria, by the respective time point.

CR or better rate, defined as the proportion of participants who achieve CR or better, according to the IMWG criteria, by the respective time point.

VGPR or better rate is defined as the proportion of participants achieving VGPR or better, according to the IMWG criteria, by the respective time point.

Response rates (Arms C, C1, [redacted]

CR or better rate.

Duration of response (DOR)

- For Arms A, A1, [redacted] the duration of response is defined as the duration from the date of initial documentation of a response (PR or better) according to the IMWG criteria to the date of first documented evidence of progressive disease according to the IMWG criteria, or death due to any cause, whichever occurs first. Participants who have not progressed and are alive will be censored at the last disease evaluation before the start of subsequent anti-myeloma therapy.

- For Arms C, C1, [redacted] the duration of response is defined as the duration from the date from the first maintenance dose to the date of first documented evidence of progressive disease according to the IMWG criteria, or death due to any cause, whichever occurs first. Participants who have not progressed and are alive will be censored at the last disease evaluation before the start of subsequent anti-myeloma therapy.

Progression-free survival (PFS) defined as the duration from the date of first dosing to the date of first documented evidence of progressive disease or death, whichever comes first. Disease progression will be determined according to the IMWG criteria. For participants who have not progressed and are alive, the PFS time will be censored at the last disease assessment before the start of any subsequent anti-myeloma therapy. Participants who withdrew consent from the study before disease progression will be censored at the last disease assessment before withdrawal of consent to study. Participants who are lost to follow-up will be censored at the last disease assessment before the participants were lost to follow-up. Participants who have not progressed and are still alive at the clinical cut-off date (data cut-off date) for analysis will be censored at the last disease assessment. Participants without any post-baseline disease assessment will be censored at the date of first dosing.

Overall survival (OS) defined as the time from the date of first dosing to the date of death from any cause. Participants who are lost to follow-up will be censored at the time of lost to follow-up. Participants who died after consent withdrawal but with death data collected as allowed by applicable regulations will be considered as having an OS event. If the participant is alive at the clinical cut-off date for the analysis or the survival status is unknown, then the participant's data will be censored at the date the participant was last known to be alive. The date of last known alive will be determined by the maximum collection/assessment date from among selected data domains within the clinical database.

3.2. Safety Endpoints

Safety endpoints include:

- AEs, TEAEs
- SAEs

- Deaths
- AEs of Clinical Interest
- Clinical Laboratory Tests
- Electrocardiograms (ECGs)
- Measures of Stem Cell Harvest
- Vital Signs
- Cardiac Function Assessments
- Eastern Cooperative Oncology Group (ECOG) Performance Scores

Adverse events

AEs and SAEs will be monitored throughout the study. AEs will be recorded in standard medical terminology and graded according to the National Cancer Institute Common Terminology Criteria for Adverse Events (NCI-CTCAE), Version 5.0, with the exception of (1) Cytokine Release Syndrome (CRS) and Immune Effector Cell-associated Neurotoxicity Syndrome [ICANS], which will be evaluated according to the American Society for Transplantation and Cellular Therapy (ASTCT) consensus grading system. The verbatim terms used in the eCRF by investigators to identify AEs will be coded using the latest Medical Dictionary for Regulatory Activities (MedDRA).

TEAEs are defined as any AE that occurs at or after the start of study treatment (e.g., Teclistamab, Talquetamab, Daratumumab, Lenalidomide, Bortezomib, or Dexamethasone) until 30 days after the last study treatment administration, or the day prior to start of subsequent anti-myeloma therapy, whichever is earlier; or an AE linked to an existing TEAE with onset date and time beyond 30 days after the last dose of study intervention but prior to the start of subsequent therapy; or any AE that is considered related to study drug (very likely, probably, or possibly related) regardless of the start date of the event; or any. If the event occurs on the day of the initiation of study treatment and either event time or time of the initiation of study treatment are missing, then the event will be assumed to be treatment emergent. If the event date is recorded as partial or completely missing, then the event will be considered as treatment-emergent unless it is known to be prior to the initiation of study treatment based on partial onset date or resolution date.

Deaths

All deaths occurring during the study will be included in the analysis.

AEs of Clinical Interest

AEs of Clinical Interest are described in Appendix 1.

Clinical Laboratory Tests

The following selected clinical laboratory tests will be analyzed:

- Hematology parameters include the following:
    - Hemoglobin
    - Platelets

  - Absolute lymphocyte counts
  - White blood cell count
  - Absolute neutrophil count
- Biochemistry parameters include the following:
  - AST
  - ALT
  - Alkaline phosphatase
  - Creatinine
  - Total bilirubin
  - Calcium
  - Phosphate
  - Uric acid
  - eGFR
- Coagulation parameters include the following:
  - Prothrombin time/INR
  - Activated partial thromboplastin time
  - Fibrinogen

Measures of stem cell harvest

The following selected data of stem cell harvest will be analyzed:

- Number of participants who underwent stem cell mobilization and who underwent transplant.
- Stem cell yield (10^6 CD34 cells/kg),
- The number of CD34+ cells transplanted (10^6/kg),
- Days to engraftment for neutrophils, assessed by investigators as the time to absolute neutrophil count (ANC) recovery, defined as the date of the first of 3 consecutive laboratory values (obtained on different days) where the ANC is >0.5x10^9/L.
- Days to engraftment for platelets, assessed by investigators as the time to platelet count recovery, defined as the date of first laboratory value where the platelet count is >20 x 10^9/L and at least 7 days after the most recent prior platelet transfusion.
- Number and proportion of participants with clonal plasma cell-negative autograft after 3 cycles of induction with Tec-DRd, Tec-DVRd, [redacted] and (if applicable) [redacted]

3.3. Other Endpoints

Pharmacokinetic endpoints

The following selected pharmacokinetic endpoints will be analyzed:

- PK parameters for teclistamab
- PK parameters for talquetamab
- PK parameters for daratumumab

Immunogenicity endpoints

The following selected immunogenicity endpoints will be analyzed:

- Incidence of anti-teclistamab antibodies
- Incidence of anti- talquetamab antibodies
- Incidence of anti- daratumumab antibodies

PRO endpoints

PRO data will be captured to assess the changes in symptoms, functioning, and overall Health Related Quality of Life (HRQoL) with treatment regimens using the following standard PRO instruments:

[redacted]

During the Treatment Phase, standard PRO instruments should be completed by the participant within 2 days prior to dosing before any clinical tests, procedures, or other consultations that would influence their perceptions of their current health state. Additionally:

- The PRO instruments will be provided in the local language in accordance with local guidelines.
- The PRO instruments will be available for regulators and for IRB/IEC submissions and will be provided separately in a companion manual with the instruments that will be submitted with the protocol.
- The PRO and AE data will not be reconciled with one another.

For PRO assessments conducted beyond disease progression or subsequent antimyeloma therapy, if no site visits are scheduled for additional disease evaluations, participants may complete the PRO assessments via telephone.

[redacted]

[redacted]

[redacted]

[redacted]

[redacted]

[redacted]

4. STATISTICAL ANALYSES

Descriptive summaries for categorical variables will include numbers and percentages; and for continuous variables will include mean, standard deviation, median, and range.

4.1. Timing of the primary analyses

The primary endpoints are AE and SAE rate and severity during treatment with teclistamab- and talquetamab-based combination regimens for each arm. The primary analysis for each regimen will be performed after all treated participants have completed the maintenance phase or (for Arm [redacted] treatment phase, or have been discontinued from study treatment by this time point. The final data cutoff and analysis, to potentially update secondary endpoints and safety, will occur at the end of study.

Additional safety analyses are planned to be performed as described in Section 4.7.

4.2. Study Participants and Data Sets Analyzed

Participant disposition, demographic and baseline disease characteristics will be summarized on the all-treated analysis set for each of the Arms.

The incidence of major protocol deviations will be summarized by category, based on the all-treated analysis set. A listing of all major protocol deviations will be provided, including participant ID, type of deviation, and reasons for deviations. A similar listing will be presented for all COVID-19 related minor protocol deviations.

4.3. Demographic and Other Baseline Characteristics

Table 3Table 4 presents a list of the demographic variables that will be summarized by arm for the all-treated analysis set.

Table 4 Demographic Variables

Continuous Variables:	Summary Type
Age (years)	Descriptive statistics (N, mean, standard deviation [SD], median and range [minimum and maximum]
Weight (kg)	
Height (cm)	
Body surface area (BSA) (m^2)	
**Categorical Variables**	
Age (<50, ≥50, <65 years, and ≥65 years)	Frequency distribution with the number and percentage of participants in each category.
Sex (male, female, undifferentiated, unknown)	
Ethnicity (Caucasian, of African descent, and Other)	
Baseline ECOG performance status (0, 1, 2)	

Table 5 presents a list of the baseline disease characteristics that will be summarized by arm for the all-treated analysis set.

Table 5 Disease Characteristics

Continuous Variables	Summary type
Time since initial multiple myeloma diagnosis (Months)	Descriptive statistics (N, mean, standard deviation [SD], median and range [minimum and maximum]).
Selected hematology laboratory analytes (hemoglobin, absolute lymphocyte count, white blood cells, absolute neutrophil count, platelet count)	
Selected chemistry laboratory analytes (sodium, potassium, AST, ALT, Alkaline phosphatase, phosphate, eGFR, total bilirubin, corrected serum calcium, uric acid, glucose, gamma-glutamyl transferase, creatinine) (baseline at randomization)	
Coagulation laboratory analytes (prothrombin time/international normalized ratio, activated partial thromboplastin time, fibrinogen)	
Vital sign parameters (pulse, systolic blood pressure, diastolic blood pressure, temperature, respiratory rate, oxygen saturation)	
**Categorical variables**	
Type of myeloma by immunofixation or serum FLC assay, n (%):<br>IgG<br>IgA<br>IgM<br>IgD<br>IgE<br>Free light chain only:<br>-Kappa<br>-Lambda<br>Biclonal<br>Negative immunofixation<br>NE	Frequency distribution with the number and percentage of participants in each category.
Type of measurable disease, n (%):<br>Serum and urine<br>Free Light Chain<br>Serum only	

Urine only<br>Other<br>NE	
ISS Staging at diagnosis, n (%):<br>I<br>II<br>III<br>Missing	
Revised ISS Staging at diagnosis, n (%):<br>I<br>II<br>III<br>Missing	
Number of lytic bone lesions:<br>None<br>1-3<br>4-10<br>More than 10	
Presence of diffuse myeloma-related osteopenia, n (%):<br>Yes<br>No<br>Unknown	
Number of extramedullary plasmacytomas, n (%) (0, 1, 2, 3, and 4)<br>Number of soft-tissue plasmacytomas [0, >=1]<br>Number of extramedullary soft-tissue plasmacytomas [0, >=1]<br>Number of paraskeletal soft-tissue plasmacytomas [0, >=1]	
% Plasma cells, bone marrow biopsy/aspirate(<10, 10 – 59, >=60, Missing)	
Cellularity, bone marrow biopsy/aspirate, n (%):<br>Hypocellular<br>Normocellular<br>Hypercellular<br>Indeterminate	
Cytogenetic risk c, n (%):<br>Standard risk<br>High risk (del(17p), t(4;14), t(14;16))	
For Arm C, C1[redacted]	
anti-CD38 antibody-based induction/consolidation (yes/no)	
>=CR at baseline (yes/no)	

4.4. Evaluation of Treatment Compliance and Exposure

Extent of exposure to study treatments will be summarized based on the safety analysis set including the following:

- Number and percentage of participants treated within each cycle,
- Maximum number of treatment cycles received by treatment phase (induction, [redacted] treatment, and maintenance),
- Total number of treatment cycles received,
- Duration of study treatment, defined as the number of months from the date of the first administration of study treatment to the date of the last dose,

- Total dose administered overall and by treatment cycle for Teclistamab SC (mg/kg), Talquetamab SC (mg/kg), Daratumumab (mg), Lenalidomide (mg), Bortezomib (mg/m^2), or Dexamethasone (mg),
- Dose intensity for each treatment, which is calculated as the sum of total doses (mg/kg, or mg/m^2, or mg) received in all cycles divided by the number of treatment cycles,
- Relative dose intensity (%), which is defined as the ratio of the total actually received dose and total planned dose. Total planned dose is calculated as the sum of planned dose level over the number of recorded infusions or dose administrations (zero & non-zero dosing records included). The planned dose for each dose administration is described in Section 6.1 of the protocol.
- Number of participants with cycle delay, dose skipped (not administered), adjusted, stopped and interrupted for Teclistamab SC, Talquetamab SC, Daratumumab SC, Lenalidomide, Bortezomib and Dexamethasone as well as the respective reasons.

4.5. Prior and Concomitant Medication

Prior and Concomitant medications will be coded using the World Health Organization (WHO) Drug Dictionary (WHO-DD). Prior medications are defined as any therapy used during screening before the day of first dose (partial or complete) of study intervention. Concomitant medications are defined as any therapy used on or after the same day as the first dose of study intervention, including those that started before and continue after the first dose of study intervention. Pre-dose study medications will not be considered concomitant medications.

Prior Multiple Myeloma Therapies

A summary of types of induction/consolidation/ASCT treatment will be provided for Arm C, Arm C1 and Arm[redacted]

Concomitant Medications

Concomitant medications collected on the eCRF page during the study will be summarized by anatomical therapeutic chemical (ATC) class and drug name. The proportion of participants who receive each concomitant medication will be summarized as well as the proportion of participants who receive at least one concomitant medication. Concomitant medications will be coded using the latest version of WHO Drug Dictionary (WHO-DD).

The number and proportion of participants who receive transfusions and immunoglobulins will be summarized. Colony stimulating factors, antivirals, antiviral prophylactic use for herpes infection by therapy class and drug will be summarized.

Systemic steroids and tocilizumab/anti-cytokine therapies as concomitant medication use for CRS and teclistamab neurotoxicity during the study will be summarized. Oxygen supplementation will be summarized as well.

Pre-treatment medications by preferred ATC class and drug will be summarized.

4.6. Analysis of Efficacy Endpoints

Responses or progression will be evaluated by the investigators and use of a validated algorithm according to the IMWG criteria (2016). No inferential analyses will be performed.

Unless otherwise specified, efficacy analyses will be conducted on the efficacy analysis population, by treatment arms. (Response rates will be provided on response-evaluable population as well; selected MRD- and response-based endpoint will be provided on pooled treatment arms as well, as specified in Section 2.2.) Note, in below, post-ASCT includes induction and ASCT, defined as the period from C1D1 of induction to prior to C1D1 maintenance treatment. Post-maintenance (for arm [redacted] post [redacted]) includes induction, ASCT, and maintenance (for arm [redacted] post [redacted]) period, defined as the period from C1D1 of induction to end of study treatment.

Minimal residual disease (MRD) negative complete response (CR) rate:

The rate of MRD negative CR post-induction (Arms A, A1, B, [redacted] and [redacted] post-ASCT (Arms A, A1, B, [redacted] and [redacted] and best overall (all arms) will be calculated within each treatment arm. The 95% Clopper-Pearson confidence intervals will be provided as well. In addition, this analysis, for post-induction and post-ASCT, will be carried out by the pooled arms as well (as specified in Section 2.2).

Sustained MRD negative CR rate:

The rate of sustained MRD negative CR will be calculated along with the Clopper-Pearson 95% confidence interval.

MRD negative CR conversion rate:

The rate of MRD negative CR conversion is defined as the proportion of participants who converted, calculated with the number of participants with response worse than CR or MRD positive status at the first maintenance dosing as the denominator (all arms except Arm [redacted] or at first dosing of [redacted] as the denominator (Arm [redacted] Participants with missing baseline MRD measurement will be considered as MRD positive. The conversion rate and the Clopper-Pearson 95% confidence interval will be calculated.

MRD negativity rate:

The rate of MRD negative and the Clopper-Pearson 95% confidence intervals will be calculated within each treatment arm. Participants with missing MRD measurement will be considered as MRD positive. Analysis will be carried out by treatment arms and, for post-induction and post-ASCT, by the pooled arms as well (as specified in Section 2.2).

Response rate (ORR, CR or better, and VGPR):

Response rates post-induction treatment, post-ASCT, post-maintenance, and best overall (Arms A, A1, B, [redacted] [redacted] [redacted] and post-induction, post-[redacted] treatment, and best overall (Arm [redacted] will be calculated, along with the Clopper-Pearson 95% confidence intervals. Analysis will be carried out by treatment arms and, for post-induction and post-ASCT, by the pooled arms as well (as specified in Section 2.2). Analysis by treatment arm will be repeated on the response-evaluable population as well.

Shift tables will be provided to present the change in response category from the first dose of the maintenance phase (all arms except Arm [redacted] of the [redacted] treatment (Arm [redacted] to the end of the treatment phase among participants with evaluations available at both timepoints.

Duration of response (DoR):

For each arm, the Kaplan-Meier DoR curve will be plotted. Median DoR (25% quantile, 75% quantile), and the respective 95% confidence intervals will be calculated.

PFS and **OS:**

For each arm, the Kaplan-Meier PFS and OS curve will be plotted. Median PFS and OS (25% quantile, 75% quantile), and the respective 95% confidence intervals will be calculated.

4.7. Analysis of Safety Endpoints

Unless otherwise specified, safety analyses will be conducted on the safety analysis population, for the entire treatment phase, by treatment arms. (Selected AE summaries will be provided on pooled treatment arms as well, as specified in Section 2.2. Selected safety analyses will be provided by treatment phase as well.)

Adverse Events

Unless otherwise specified, at each level (e.g., system organ class [SOC] and/or preferred term [PT]) of participant summarization in reporting the incidence of the AE, a participant is counted once if one or more events were recorded. For summarizing new onset events, all event records of the same preferred term from the same participant are to be linked by the onset date and the end date. If an event is followed by another event of the same preferred term with an onset date (or date/time) the same as or 1 day (or 1 minute if applicable) after the end date (or date/time) of the previous record and any features of the adverse event (i.e.: toxicity grades/seriousness/action taken) are different between these two records, these 2 records should be linked together and considered as one event. A Grade 5 event will be linked to previous event of the same preferred term if the onset date of grade 5 record is the same or one day after the end date of previous record. All summaries of AEs will be based on TEAEs.

Primary Safety Endpoints

Summary of TEAE will include the number of participants for each treatment cohort, in whom the event occurred and the rate of occurrence of:

• TEAE by SOC and PT, by worst grade

• TESAE by SOC and PT, by worst grade

Other Safety Endpoints

Incidence of AE during induction is based on AE onset during the induction treatment cycles (1 to 6, and within 30 days of the last induction dose).

Incidence of AE during maintenance (for arm [redacted] during [redacted] treatment) is based on AE onset during maintenance (for arm [redacted] treatment cycles (1 to 18, and AE within 30 days of the last dose).

During the immediate post-transplant period (defined as the first day of high-dose melphalan administration to the first dose of maintenance therapy; arms A, A1, [redacted]), only the following AEs, and concomitant medications and procedures associated with these AEs, have to be recorded in the eCRF:

• Any evolution of an ongoing AE

• Any new AE related to the induction therapy

The same approach also applies to AEs during SoC maintenance (all arms).

Summary of TEAE will include the number of participants, by treatment phase for each treatment cohort, in whom the event occurred and the rate of occurrence of:

• An overview of the TEAE, including participants with TEAE, Treatment-emergent Serious Adverse Event (TESAE), serious AE (SAE), TEAE related to study drug, TEAE of maximum grade 1 to 5, TEAE with outcome of death

• TEAE by SOC, PT and toxicity grade 3/4*

• TESAE by SOC and PT*

• Toxicity grade 3 or 4 TEAE by SOC, PT, and relationship to study drug

• TEAE with outcome death by SOC, PT and relationship to study drug*

• Treatment-emergent infusion related reactions

Analyses denoted by * will be repeated by treatment phase, for the pooled treatment arms, as specified in Section 2.1.

Incidence treatment emergent AE will also be summarized by treatment cycle.

In addition to the summary tables, listings will be provided for participants who:

• Had Serious TEAEs

• Had TEAEs leading to discontinuation of study treatment.

Analyses of AEs of clinical interest are described in Appendix 1.

Death

The number and corresponding percentage of participants who died during the study and the primary cause of death will be summarized for the safety analysis set. In addition, all deaths within 30 days after the last dose of treatment will also be summarized respectively.

A listing will be generated for participants from the safety set who died during the study.

ECOG Performance Scores

ECOG performance status evaluates the effect of the disease status on the activities of daily living. Descriptive statistics will be used to summarize ECOG performance status at baseline,

scheduled post-baseline timepoints (including change from baseline), worst score during post injection period (including change from baseline) for each treatment group. Shift table from baseline to worst score during the post injection period may be provided.

Vital signs

Descriptive statistics will be used to summarize the vital signs (systolic and diastolic blood pressure, pulse/heart rate, respiratory rate, oxygen saturation, and temperature) values at baseline. Weight loss from baseline during treatment will be summarized.

Electrocardiogram

The interpretation (normal, abnormal clinically insignificant, abnormal clinically significant, not evaluable) of the ECGs as determined by a qualified physician (investigator or qualified designee) will be displayed by the number and percentage of participants meeting the normality criteria. A shift table of baseline versus worse ECG result post-baseline will be provided as well as a listing of participants with clinically significant abnormal ECG results.

Clinical Laboratory Evaluation

Descriptive statistics will be used to summarize observed laboratory analyte at baseline and for observed values and change from baseline in observed value at each scheduled timepoint for each study treatment. Line plots of the mean with standard error for each laboratory analyte over time will be displayed for the selected laboratory analytes (see Section 3.2).

Stem cell harvest:

The number (%) of participants who undergone stem cell mobilization and who undergone transplant will be summarized.

Stem cell yield (10^6 CD34 cells/kg), the number of CD34+ cells transplanted (10^6/kg), days to engraftment for neutrophils (0.5×10^9/L) and days to engraftment for platelets (20×10^9/L) will be summarized by mean, standard deviation, median, minimum and maximum.

When participants' neutrophils or platelets did not drop below the specified threshold, some investigators captured engraftment days as 0. As such, 0 engraftment day will not be included in the summary of data. The ASCT related information will be listed.

In addition, medications received for stem cell mobilization will also be summarized.

4.8. Analyses of Other Endpoints

Pharmacokinetic endpoints:

Pharmacokinetic data from participants in the pharmacokinetic analysis set will be presented at each sampling timepoint. All serum concentrations below the lowest quantifiable concentration or missing data will be labeled as such in the concentration data presentation. Concentrations below the lowest quantifiable concentration will be treated as zero in the summary statistics. Data from participants outside the prespecified visit window will be reported, but not included in the summary statistics. All participants and samples excluded from the analysis will be clearly documented in the data presentation specification.

Descriptive statistics will be used to summarize Tec serum concentrations at each sampling time point. Line plot of mean (±SD) serum trough concentrations over time will be provided.

Immunogenicity endpoints:

The incidence of antibodies to teclistamab, talquetamab, and daratumumab will be summarized separately for all participants who receive ≥1 dose of teclistamab, talquetamab, or daratumumab and have appropriate samples for detection of ADAs. A listing of any participants who are positive for ADAs to teclistamab, talquetamab or daratumumab will also be presented.

PRO endpoints:

The [redacted], [redacted] and [redacted] will be summarized descriptively.
.

For the [redacted] graph will display the distribution of response options for each item, by study visit.

For [redacted], [redacted] line plot of mean (±SE) over time will be plotted by treatment arm for each individual scale.

4.9. Interim Analyses and Safety Monitoring Analyses

The following safety analyses are planned to be performed and data will be reviewed by the Study Safety Committee and the Study Steering Committee:

- Arm A: After at least 6 participants have received at least 2 cycles of Tec-DRd induction treatment with successful stem cell mobilization and collection performed in at least 3 participants after at least 3 cycles. As of Amendment 4, Arm A has enrolled 10 participants.
- Arms A1 and B: After at least 10 participants in each arm have received at least 3 cycles of Tec-DR (Arm A1) or Tec-DVRd (Arm B) induction treatment. It is expected that 20 participants will be enrolled in each arm (Arm A1 and Arm B) at the time of this safety snapshot analysis.
- Arm [redacted] After approximately 6 participants have received at least 3 cycles of [redacted].
- Arms [redacted] After approximately 6 participants in each arm have received at least 3 cycles of [redacted] induction treatment.
- Arms [redacted] After approximately 6 participants in each arm have received at least 3 cycles of [redacted] induction treatment.
- Arms C, C1, [redacted] After approximately 6 participants have received at least 3 cycles of Tec-DR, Tec-D, or [redacted] maintenance treatment. It is expected that 10 participants will be enrolled in each arm at the time of the respective safety snapshot analyses.

The sponsor may take the decision that based on emerging data from other arms or from external data or based on practical considerations (e.g., to reasonably align safety snapshots

from different arms), these planned safety analyses may be modified. In addition, safety data from all arms will be reviewed on an ongoing basis and additional safety analyses may be triggered at any time during the study.

REFERENCES

1. Kumar S, Paiva B, Anderson KC, et al. International Myeloma Working Group consensus criteria for response and minimal residual disease assessment in multiple myeloma. Lancet Oncol. 2016;17(8):e328-e346.
2. Rajkumar SV, Harousseau JL, Durie B, et al. Consensus recommendations for the uniform reporting of clinical trials: report of the International Myeloma Workshop Consensus Panel 1. Blood. 2011;117(18):4691-4695.
3. Lee DW, Santomasso BD, Locke FI, et al. ASTCT consensus grading for cytokine release syndrome and neurological toxicity associated with immune effector cells. Biol Blood Marrow Transplant. 2019; 25 (4):625-638
4. ICH Guidelines E3 "Structure and Content of Clinical Study Reports"
5. ICH Guidelines E9 "Statistical Principles for Clinical Trials"
6. CCI [redacted]
7. CCI [redacted].
8. CCI [redacted]
9. CCI [redacted]

APPENDIX 1 Further Definition of Endpoints

Adverse Events of Clinical Interest

Adverse events of clinical interest include tumor lysis syndrome (TLS), cytopenias (anemia, neutropenia, thrombocytopenia, lymphopenia), infections (including opportunistic infections and viral infections), systemic administration-related reactions (sARRs) related to teclistamab or daratumumab, local injection site reactions (ISRs) related to teclistamab or daratumumab, hypogammaglobulinemia, immune-mediated/auto immune disorders, hepatotoxicity, and second primary malignancies. CRS, ICANS, and neurologic adverse events are also considered adverse events of clinical interest.

Cytokine Release Syndrome

Participants with any CRS will be summarized by the maximum toxicity grades (according to ASTCT[2] consensus grading system). In addition, the time from the most recent dose to the first onset of CRS, the time of CRS occurrence, CRS leading to discontinuation of study drug, the duration of CRS in days, the outcome of CRS, and the treatment for CRS will be summarized as well.

Additionally, participants with any symptom of CRS will be summarized by MedDRA SOC, PT and maximum toxicity grade (according to NCI-CTCAE version 5.0).

Listings will be provided respectively for participants who reported any CRS.

Immune Effector Cell-associated Neurotoxicity Syndrome

Participants with any ICANS will be summarized by the maximum toxicity grades (according to ASTCT[2] consensus grading system). In addition, the time from the most recent dose of teclistamab to new onset of ICANS, the time of ICANS occurrence, ICANS leading to discontinuation of study drug, the duration of ICANS, the outcome of ICANS, the treatment of ICANS, and concurrent/non-concurrent CRS will be summarized as well. Additionally, participants with any symptom of ICANS will be summarized by SOC, PT and maximum toxicity grade (according to NCI-CTCAE version 5.0).

Listings will be provided for participants who reported any ICANS.

Tumor Lysis Syndrome

Participants with any TLS will be summarized by MedDRA SOC, PT and maximum toxicity grade (according to NCI-CTCAE version 5.0). A listing of participants who reported any treatment-emergent TLSs during the study will be provided.

Second Primary Malignancies

A listing of participants who reported second primary malignancies during the study will be provided. This listing will include diagnosis, study day of diagnosis, stage of disease, recurrence of a prior existing malignancy (yes, no) and pathology diagnosis (biopsy, aspirate, etc.) information whenever a second primary malignancy is observed. In addition, prior

exposure to multiple myeloma therapies, cumulative study treatment exposure, whether or not participants received subsequent anticancer therapy (yes, no), the treatment for second primary malignancy and the outcome information will also be presented in the listing. Second primary malignancies will be clinically reviewed and categorized as cutaneous/non-invasive, non-cutaneous/invasive, or hematologic malignancies, which will be summarized accordingly.

Other adverse events of clinical interest

Neurologic adverse events are defined as adverse events in the Nervous System Disorders SOC or Psychiatric Disorders SOC regardless of relationship with study treatment. Participants with any neurologic adverse event reported after the first dose of study treatment will be summarized by MedDRA SOC, PT, and grade 3 or 4. Listings will be provided for participants who reported any neurologic adverse events.

Participants with any cytopenias reported after the first dose of study treatment will be summarized by MedDRA SOC, PT, and worst toxicity grade of 3 or higher, as well as by treatment cycle. Hemorrhage will be summarized by MedDRA preferred term and maximum toxicity grade. Hemorrhage events will be based on the Standardized MedDRA Queries (SMQ) with the first subcategory SMQ of hemorrhage terms (excluding laboratory terms).

A summary of number of participants with 1 or more treatment-emergent infections and infestations by MedDRA preferred term and worst toxicity grade of 3 or higher will be provided. In addition, treatment-emergent infections and infestations will be summarized by MedDRA preferred term and treatment cycle. Opportunistic infections will be summarized by MedDRA preferred term. Opportunistic infections will include preferred terms from the Standardized MedDRA Queries (SMQ) of narrow scope and some additional terms identified by sponsor.

Participants with local injection site reactions (ISR) related to teclistamab or daratumumab will be summarized by MedDRA preferred term and maximum toxicity grade. A summary of participants with ISRs by maximum toxicity grade, the time from the most recent dose of treatment to new onset of ISR, the duration of ISR, the outcome of ISR, and the treatment of ISR will be provided as well. Systemic administration-related reactions (sARRs) related to daratumumab will be summarized by MedDRA preferred term and maximum toxicity grade. Listings will be provided for participants with sARRs related to teclistamab. In addition, time to onset of sARRs will be summarized.

Hypogammaglobulinemia will be assessed by TEAEs and by laboratory IgG values. Participants with hypogammaglobulinemia will be summarized by PT and maximum toxicity grade.

Participants with immune-mediated/autoimmune disorders will be summarized by MedDRA PT, and maximum toxicity grade. The list of preferred terms that will be used for the assessment of immune-mediated/autoimmune disorders is provided in Appendix 2.

Hepatotoxic TEAEs and liver laboratory parameters will be summarized.

APPENDIX 2 Immune-mediated/autoimmune disorders

Immune-mediated/autoimmune disorders defined by MedDRA Preferred Terms:

· Immune-mediated adverse reaction · Noninfective encephalitis · Autoimmune pancytopenia · Autoimmune encephalopathy · Autoimmune neutropenia · Immune-mediated encephalopathy · Immune thrombocytopenia · Autoimmune enteropathy · Immune-mediated cytopenia · Toxic epidermal necrolysis · Autoimmune aplastic anaemia · Immune-mediated hepatic disorder · Autoimmune haemolytic anaemia · Autoimmune hepatitis · Addison's disease · Immune-mediated hepatitis · Adrenal insufficiency · Hyperthyroidism · Primary adrenal insufficiency · Immune-mediated hyperthyroidism · Immune-mediated adrenal insufficiency · Primary hyperthyroidism · Adrenocortical insufficiency acute · Hypophysitis · Arthritis · Immune-mediated hypophysitis · Polyarthritis · Autoimmune hypothyroidism · Acute aseptic arthritis · Immune-mediated hypothyroidism · Autoimmune arthritis · Primary hypothyroidism · Immune-mediated arthritis · Autoimmune thyroiditis · Polymyalgia rheumatica · Immune-mediated thyroiditis · Autoimmune blistering disease · Thyroiditis · Autoimmune demyelinating disease · Thyroiditis acute · Autoimmune cholangitis · Hypopituitarism · Immune-mediated cholangitis · Immune-mediated lung disease · Immune-mediated cholestasis · Interstitial lung disease · Chronic inflammatory response syndrome · Pneumonitis · Immune-mediated cystitis · Myasthenia gravis · Autoimmune colitis · Myasthenia gravis crisis · Colitis · Myasthenic syndrome · Colitis ulcerative · Immune-mediated myasthenia gravis · Enterocolitis · Autoimmune myocarditis · Immune-mediated enterocolitis · Giant cell myocarditis · Immune-mediated gastritis · Immune-mediated myocarditis · Enterocolitis haemorrhagic · Myocarditis · Enteritis · Immune-mediated pericarditis · Immune-mediated endocrinopathy · Autoimmune myositis · Autoimmune dermatitis · Immune-mediated myositis · Dermatitis exfoliative · Polymyositis · Dermatitis exfoliative generalized · Myositis · Immune-mediated dermatitis · Necrotising myositis · Fulminant type 1 diabetes mellitus · Autoimmune nephritis · Type 1 diabetes mellitus · Immune-mediated nephritis · Immune-mediated encephalitis · Nephritis · Tubulointerstitial nephritis · Autoimmune neuropathy · Immune-mediated neuropathy · Immune-mediated neurologic disorder · Immune-mediated oesophagitis · Autoimmune pancreatitis · Immune-mediated pancreatitis · Pancreatitis · Pancreatitis acute · Immune-mediated renal disorder · Rhabdomyolysis · Severe cutaneous adverse reaction · Stevens-Johnson syndrome · Autoimmune uveitis · Immune-mediated uveitis · Uveitis · Immune-mediated scleritis · Vasculitis · Vasculitis necrotising · Vogt-Koyanagi-Harada disease
