# Supplementary material for: Teclistamab-based induction treatment in transplant-eligible, newly diagnosed multiple myeloma: a phase 2 trial
Source: Nat Med. 2026 Jun 25;32(7):2440–8. doi: 10.1038/s41591-026-04471-x (PMC13375549; doi:10.1038/s41591-026-04471-x)
Supplement: Supplementary file 2 — Reporting Summary [file 41591_2026_4471_MOESM2_ESM.pdf]

nature portfolio

Corresponding author(s): Marc S. Raab

Last updated by author(s): May 12, 2026

Reporting Summary

Nature Portfolio wishes to improve the reproducibility of the work that we publish. This form provides structure for consistency and transparency in reporting. For further information on Nature Portfolio policies, see our Editorial Policies and the Editorial Policy Checklist.

Statistics

For all statistical analyses, confirm that the following items are present in the figure legend, table legend, main text, or Methods section.

n/a	Confirmed	
☐	☒	The exact sample size (*n*) for each experimental group/condition, given as a discrete number and unit of measurement
☐	☒	A statement on whether measurements were taken from distinct samples or whether the same sample was measured repeatedly
☒	☐	The statistical test(s) used AND whether they are one- or two-sided<br>*Only common tests should be described solely by name; describe more complex techniques in the Methods section.*
☒	☐	A description of all covariates tested
☒	☐	A description of any assumptions or corrections, such as tests of normality and adjustment for multiple comparisons
☐	☒	A full description of the statistical parameters including central tendency (e.g. means) or other basic estimates (e.g. regression coefficient) AND variation (e.g. standard deviation) or associated estimates of uncertainty (e.g. confidence intervals)
☒	☐	For null hypothesis testing, the test statistic (e.g. *F*, *t*, *r*) with confidence intervals, effect sizes, degrees of freedom and *P* value noted<br>*Give P values as exact values whenever suitable.*
☒	☐	For Bayesian analysis, information on the choice of priors and Markov chain Monte Carlo settings
☒	☐	For hierarchical and complex designs, identification of the appropriate level for tests and full reporting of outcomes
☒	☐	Estimates of effect sizes (e.g. Cohen's *d*, Pearson's *r*), indicating how they were calculated

Our web collection on statistics for biologists contains articles on many of the points above.

Software and code

Policy information about availability of computer code

Data collection	MajesTEC-5 used electronic Case Report Forms (eCRFs) to collect patient data.
Data analysis	No formal statistical hypothesis testing is planned in this study; all data are presented descriptively. Response was assessed by Investigators per IMWG criteria (requiring >=2 consecutive identical response assessments). Minimal residual disease (MRD) at the 1x10-5 threshold was assessed by next-generation flow (NGF) using EuroFlow. Additionally, aspirates collected after cycle 6 were assessed for MRD at the 1x10-6 threshold by next-generation sequencing (NGS) using ClonoSEQ assay (Version 2.0; Adaptive Biotechnologies). SAS software (Version 9.4) was used for all outputs.

For manuscripts utilizing custom algorithms or software that are central to the research but not yet described in published literature, software must be made available to editors and reviewers. We strongly encourage code deposition in a community repository (e.g. GitHub). See the Nature Portfolio guidelines for submitting code & software for further information.

Data

Policy information about availability of data

All manuscripts must include a data availability statement. This statement should provide the following information, where applicable:

- Accession codes, unique identifiers, or web links for publicly available datasets
- A description of any restrictions on data availability
- For clinical datasets or third party data, please ensure that the statement adheres to our policy

A data availability statement that conforms with that required by the Sponsor can be found in the "Data Availability" section of this manuscript. Of note, data found and presented within this manuscript is considered the minimum dataset required to interpret, verify and extend research in the article. The data availability statement is as follows:"The Heidelberg University Hospital in partnership with the German-Speaking Myeloma Multicenter Group (GMMG), Deutsche Studiengruppe Multiples Myelom (DSMM), and Johnson & Johnson will make the data available according to the data sharing policy of Johnson & Johnson, which is available at https://innovativemedicine.jnj.com/our-innovation/clinical-trials/transparency. As noted on this site, requests for access to the study data can be submitted through the Yale Open Data Access (YODA) Project site at http://yoda.yale.edu." As noted, readers may make a request for data per the Yale Open Data Access site, and data will be shared/made available per sponsor policy.

Research involving human participants, their data, or biological material

Policy information about studies with human participants or human data. See also policy information about sex, gender (identity/presentation), and sexual orientation and race, ethnicity and racism.

Reporting on sex and gender	Sex was collected and reported in the study; sex was reported by the patient. Total number and percent of male and female patients recruited across arms are provided. A disaggregated analysis by sex was not pre-specified in the trial protocol or statistical analysis plan, and was not performed as sex was not considered a major confounder in the newly diagnosed multiple myeloma field.
Reporting on race, ethnicity, or other socially relevant groupings	Ethnicity of patients is reported in Table 1 of the manuscript in a descriptive manner (ie, n/N [%])
Population characteristics	Enrolled patients were 18 to 70 years of age, had documented newly diagnosed multiple myeloma per IMWG diagnostic criteria, were eligible for high-dose therapy and ASCT, and had an ECOG performance status score of 0 to 2. Patients had a hemoglobin level of ≥7.5 g/dL (≥4.65 mmol/L; without prior red blood cell transfusion within 7 days before the laboratory test), a platelet count of ≥75×10^9/L (if <50% of bone marrow-nucleated cells were plasma cells; otherwise, the platelet count was ≥50×10^9/L), an absolute neutrophil count of ≥1.0×10^9/L (prior growth factor support was permitted, but must have been without granulocyte–colony stimulating factor [G-CSF] or granulocyte–macrophage colony stimulating factor support for 7 days, or without pegylated-G-CSF for 14 days). In addition, patients had aspartate and alanine aminotransferase levels of ≤2.5 times the upper limit of normal, an estimated glomerular filtration rate of ≥30 mL/min (based on Cockroft-Gault formular or 24-hour urine collection), a total bilirubin level ≤2.0 times the upper limit of normal, and a corrected serum calcium level of ≤14 mg/dL (≤3.5 mmol/L) or free ionized calcium of ≤6.5 mg/dL (≤1.6 mmol/L). Excluded were patients with prior or current systemic therapy or stem cell transplant (exception of a short course of corticosteroids), prior radiotherapy or focal radiation (within 14 or 7 days, respectively, of enrollment), prior plasmapheresis (within 28 days of enrollment), prior or concurrent invasive malignancy (other than multiple myeloma), central nervous system involvement or clinical signs of meningeal involvement of multiple myeloma, seropositive for human immunodeficiency virus, hepatitis B or C infection (or received a live, attenuated vaccine within 28 days of first study dose), chronic obstructive pulmonary disease with a forced expiratory volume in 1 s <50% of predicted normal, moderate or severe persistent asthma within the past 2 years or currently uncontrolled asthma, grade ≥2 peripheral neuropathy or neuropathic pain, concurrent medical or psychiatric condition or disease (likely to interfere with study procedures), cardiac conditions, contraindications or life-threatening allergies, hypersensitivity, or intolerance to any study drug or its excipients, prior traumatic injury or surgery (within 14 days of enrollment), received an investigational drug or investigational medical device, or gastrointestinal disease (impacting absorption of oral drugs). A complete list of inclusion/exclusion criteria are provided in the redact protocol provided as part of the Supplementary Information.<br><br>Complete patient characteristics of those patients included in Cohorts A, A1 and B (presented herein) are summarized in Table 1.
Recruitment	This is an ongoing, multicenter, open-label, non-randomized, multi-cohort, phase 2 study (ClinicalTrials.gov number, NCT05695508). Those patients presented herein were enrolled from 11 sites across Germany. Patients were enrolled between 13 December, 2022 and 6 March, 2023, in Arm A, and between the 17/24 October, 2023 and 14 March, 2024 in Arm A1/Arm B, respectively.
Ethics oversight	The study was conducted in accordance with the principles of the Declaration of Helsinki, the International Council for Harmonisation guidelines for Good Clinical Practice, and any country-specific regulations. The study protocol and relevant documents were approved at each site by an independent ethics committee (Ethikkommission der Medizinischen Fakultät Heidelberg [Ethics Committee of the Medical Faculty Heidelberg]) and institutional review board (Paul-Ehrlich-Institut, Bundesinstitut für Impfstoffe und biomedizinische Arzneimittel [Federal Institute for Vaccines and Biomedicinal Drugs]). All protocol amendments were similarly approved by the independent ethics committee and/or institutional review board at each study site, and are outlined in the Supplementary Protocol available with the Supplemental Information. All patients provided written informed consent. A Study Safety Committee was established to monitor safety, inclusive of 2 independent

external medical experts in the relevant therapeutic area.

Note that full information on the approval of the study protocol must also be provided in the manuscript.

Field-specific reporting

Please select the one below that is the best fit for your research. If you are not sure, read the appropriate sections before making your selection.

☒ Life sciences ☐ Behavioural & social sciences ☐ Ecological, evolutionary & environmental sciences

For a reference copy of the document with all sections, see nature.com/documents/nr-reporting-summary-flat.pdf

Life sciences study design

All studies must disclose on these points even when the disclosure is negative.

Sample size	Sample size determination was guided by precision for estimation of adverse event incidence. The most conservative scenario for a binomial proportion occurs at an event rate of 50%; therefore, precision was assessed using a 2-sided exact (Clopper-Pearson) 95% confidence interval assuming a true incidence of 50%.<br><br>For Arm A, approximately 10 patients were planned to be enrolled. For Arms A1 and B, an initial enrollment of approximately 20 patients in each arm was planned, with the potential for expansion up to a total of 80 patients (Arms A1 and B combined)<br><br>This pooled data analysis of Arms A, A1 and B was conducted to characterize safety and efficacy prior to the maintenance treatment period, since all patients have completed both the induction treatment phase and ASCT, specifically. Pooled arm reporting was permitted by the statistical analysis plan with modifications permitted to make reporting more informative (ie, for interpreting adverse event patterns), to account for data availability and, arms open for enrolment at different times, and exploration of additional combinations for exploratory purposes.
Data exclusions	No data exclusion was performed
Replication	N/A (clinical trial)
Randomization	N/A (unblinded and non-randomized as mentioned in the Methods)
Blinding	N/A (unblinded and non-randomized as mentioned in the Methods)

Reporting for specific materials, systems and methods

We require information from authors about some types of materials, experimental systems and methods used in many studies. Here, indicate whether each material, system or method listed is relevant to your study. If you are not sure if a list item applies to your research, read the appropriate section before selecting a response.

Materials & experimental systems

n/a	Involved in the study	
☒	☐	Antibodies
☒	☐	Eukaryotic cell lines
☒	☐	Palaeontology and archaeology
☒	☐	Animals and other organisms
☐	☒	Clinical data
☒	☐	Dual use research of concern
☒	☐	Plants

Methods

n/a	Involved in the study	
☒	☐	ChIP-seq
☒	☐	Flow cytometry
☒	☐	MRI-based neuroimaging

Clinical data

Policy information about clinical studies

All manuscripts should comply with the ICMJE guidelines for publication of clinical research and a completed CONSORT checklist must be included with all submissions.

Clinical trial registration	This trial was registered with ClinicalTrials.gov (NCT05695508)
Study protocol	The redacted Study Protocol and redacted Statistical Analysis Plan (SAP) are included in the Supplementary Information file
Data collection	This is an ongoing, multicenter, open-label, non-randomized, multi-cohort, phase 2 study (ClinicalTrials.gov number, NCT05695508). Those patients presented herein were enrolled from 11 sites across Germany. Patients were enrolled between 13 December, 2022 and 6 March, 2023, in Arm A, and between the 17/24 October, 2023 and 14 March, 2024 in Arm A1/Arm B, respectively.

Outcomes

The analysis period reported in this manuscript is from the first dose of study treatment to the last visit prior to the start of maintenance (pre-maintenance treatment period).

The primary endpoint was the incidence and severity of adverse events and serious adverse events over the entire treatment phase (pre-maintenance treatment period). Pre-specified secondary efficacy endpoints reported herein, include MRD-negative CR rate, MRD-negativity rates (at 1×10−5 and 1×10−6 sensitivity thresholds), overall response rate (partial response or better), ≥VGPR, ≥CR, and stem-cell yield and days to engraftment. MRD negativity was defined, regardless of response, as achieving MRD-negative status by next-generation flow cytometry (NGF) with a sensitivity of 1×10−5 after the date of first dose of the study medication and prior to progressive disease, subsequent therapy, or both. MRD-negative status was also assessed by next-generation sequencing (NGS) with a sensitivity of 1×10−6. MRD-negative CR was defined as achieving MRD negativity (per NGF at 1×10-5) and ≥CR at any time after the date of first dose of the study medication and prior to progressive disease, subsequent therapy, or both. Overall response rate, ≥CR and ≥VGPR were defined as the proportion of patients who achieved the respective response per IMWG criteria, by the pre-maintenance treatment period.

Additional secondary endpoints that are not reported at this time include duration of response, sustained (≥12 month) MRD-negative CR rate (1×10−5), progression-free survival, pharmacokinetic parameters, and immunogenicity. As previously mentioned, a disaggregated analysis by sex was not pre-specified in the trial protocol or statistical analysis plan, and was not performed as sex was not considered a major confounder in the newly diagnosed multiple myeloma field.

Both the safety and efficacy analysis set consisted of all patients who received ≥1 dose of study treatment. The MRD-evaluable analysis set consisted of all treated patients with an available sample per testing modality (NGF or NGS) that was either positive or negative, excluding those who were not tested, tested but sample is indeterminate, had no baseline clone detected (as it pertains to NGS), or with a missing sample.

Plants

Seed stocks

N/A

Novel plant genotypes

N/A

Authentication

N/A
